# B‐N Fused Anthracene as Functional Linker for π‐Extended Viologens: Near‐IR Emission and Electrochromism

**DOI:** 10.1002/anie.202521634

**Published:** 2025-12-10

**Authors:** Rajendra Prasad Nandi, Jingyao Zuo, Abhishek Shibu, Frieder Jäkle

**Affiliations:** ^1^ Department of Chemistry Rutgers University‐Newark 73 Warren Street Newark NJ 07102 USA; ^2^ HORIBA Instruments Incorporated 20 Knightsbridge Road Piscataway New Jersey 08854 USA

**Keywords:** Anthracene, Boron, Electrochromism, NIR emission, Viologen

## Abstract

As prototypical organic electrochromic materials viologens have been extensively studied in display technologies, smart materials, and energy storage applications. Their properties can be fine‐tuned by introducing different substituents on the pyridine rings, fusion with heteroatoms, or insertion of π–conjugated linkers. In this article we study the effect of B‐N fused dipyridylanthracene (BDPA) as a novel linker unit in viologens on the electronic structure, optical properties, and electrochromic characteristics. Quaternization of pyridyl‐functionalized BDPA (**1Py**) by N‐methylation or complexation with B(C_6_F_5_)_3_ as a powerful Lewis acid gives rise to two fundamentally different π‐extended viologens, dicationic [**1Py‐Me**](PF_6_)_2_, and the neutral complex **1Py‐BCF**. We investigate the effect of these different quaternization methods on the LUMO energy, band gaps, absorption and emission, and the self‐sensitized reactivity toward oxygen. We also demonstrate facile electrochemical reduction to singly and multiply reduced species. Spectroelectrochemical and computational studies reveal formation of strongly colored doubly reduced species with a closed shell electronic configuration and prominent quinoidal delocalization. The corresponding radical anions give rise to absorptions in the near‐IR. A prototype electrochromic device with **1Py‐BCF** as the redox‐active material is also presented.

## Graphical abstract

We introduce π–extended viologens with B‐N fused dipyridylanthracene (BDPA) as conduit for delocalization. Quaternization of pyridyl‐substituted BDPA by methylation or Lewis pair formation with B(C_6_F_5_)_3_ produces strongly colored compounds that are emissive in the near‐IR. Spectroelectrochemical studies reveal reversible formation of doubly reduced species that adopt a closed shell electronic configuration with prominent quinoidal delocalization.

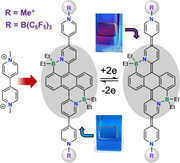

## Introduction

**Figure 1:**
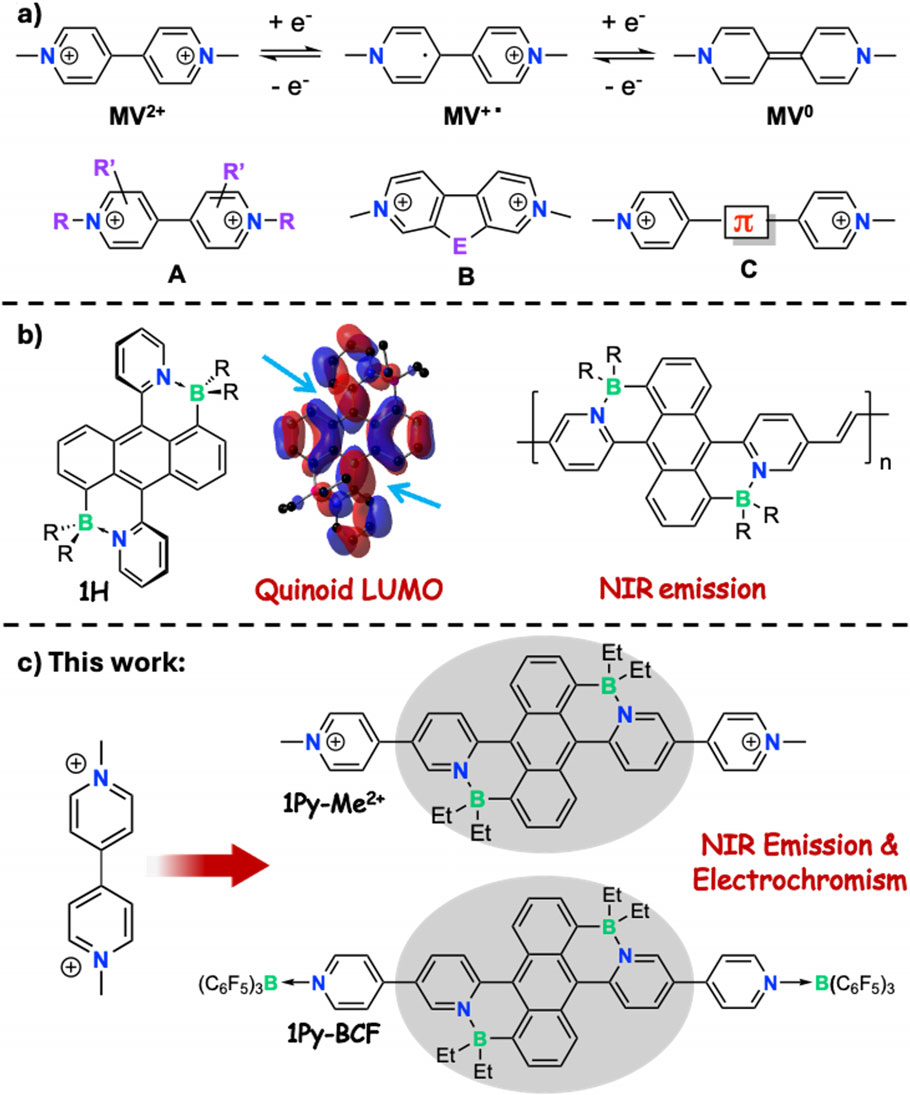
a) Examples of viologens and their redox properties (counterions omitted). b) Development of NIR emitting BDPA polymer through LUMO extension (the blue arrows highlight π‐interactions between anthracene and pyridine in the LUMO). c) Viologen‐type compounds with a BDPA linker showing NIR emission and electrochromic behavior.

Electrochromism (EC) refers to the alteration of the color of molecular species in response to an electrical stimulus by means of electron transfer between two different redox states. Electrochromic materials are useful in display technologies, molecular switches, and energy storage applications.^[^ ^1^, ^2^ ^]^ Most commercially available EC materials are metal‐based which is unfavorable in terms of production cost, toxicity, and tunability, making research advances on organic EC materials extremely important.^[^ ^3^, ^4^ ^]^ Among the most thoroughly investigated organic EC materials are viologens (*N*,*N*’‐disubstituted‐4,4'‐bipyridinium salts). As illustrated in Figure 1a, methyl viologen (MV^2+^) undergoes reversible 2‐step reductions accompanied by dramatic color changes from colorless to blue‐violet and brownish.^[^ ^5^, ^6^ ^]^ The properties of viologens can be tuned by structural modifications^[^ ^7^, ^8^, ^9^, ^10^ ^]^ primarily through (a) variation of substituents on the pyridine rings and counterions,^[^ ^11^ ^]^ (b) fusion of the pyridine rings with heteroatoms (E),^[^ ^12^, ^13^ ^]^ and (c) use of π‐conjugated linkers between pyridine rings (**A‐C**, Figure 1a).^[^ ^14^, ^15^, ^16^, ^17^, ^18^, ^19^ ^]^ Polycyclic aromatic hydrocarbons (PAHs) are particularly attractive as linkers because the large planar π‐systems not only provide pathways for extended delocalization between pyridinium moieties but also offer complementary absorption and emission characteristics and promote intermolecular interactions, for example with DNA.^[^ ^20^, ^21^, ^22^, ^23^ ^]^

The modification of PAHs with heteroatoms has attracted enormous attention in recent times owing to the dramatic effects on the photophysical and electronic properties that can be achieved.^[^ ^24^, ^25^, ^26^ ^]^ The concept of BN/CC isosterism where a nonpolar C─C bond is replaced with a dipolar B─N bond has been utilized extensively.^[^ ^27^, ^28^, ^29^, ^30^, ^31^, ^32^ ^]^ Although boron is usually incorporated in its tricoordinate form, recent studies have very effectively utilized tetracoordinate motifs with B←N dative bonding to alter the electronic structure of conjugated materials.^[^ ^33^, ^34^, ^35^, ^36^, ^37^ ^]^ In earlier work, Yamaguchi and coworkers reported dimesitylboryl‐substituted thienylthiazoles dimers as novel electron‐transporting materials,^[^ ^38^ ^]^ Ingleson, Turner, and coworkers developed benzothiadiazole‐based B‐N systems for OLED applications,^[^ ^39^ ^]^ Wakamiya, Murata, and coworkers introduced a solvato‐, thermo‐, and mechanochromic probe based on the reversibility of the intramolecular B←N coordination,^[^ ^40^ ^]^ and Thilagar and coworkers explored B←N fused Ni(II) porphyrins as potential photosensitizers.^[^ ^41^ ^]^ Many recent studies have focused on the development of luminescent and chiroptical materials, acceptors for photovoltaics, and molecular switches based on B←N modified PAHs.^[^ ^42^, ^43^, ^44^, ^45^, ^46^, ^47^, ^48^, ^49^ ^]^ As part of our program on B←N Lewis pair‐fused PAHs, we have pursued the N‐directed borylation of dipyridyl derivatives of fluorene,^[^ ^50^, ^51^, ^52^ ^]^ pyrene,^[^ ^53^ ^]^ and anthracene.^[^ ^54^, ^55^, ^56^, ^57^, ^58^, ^59^ ^]^ Modification of anthracene with B←N Lewis pairs in particular serves as a highly effective tool to modify the electronic structure with important ramifications on the emissive properties and the generation of and reactivity toward singlet oxygen. Our investigations on B‐N fused 9,10‐dipyridylanthracenes (BDPAs) such as **1H** (Figure 1b) and its derivatives revealed that the LUMO energy is greatly reduced because of the quinoidal character and extensive delocalization over the central anthracene ring and pyridine moieties.^[^ ^54^, ^55^, ^56^ ^]^ The LUMO level was further lowered by extension of conjugation (“LUMO extension”)^[^ ^60^ ^]^ at the pyridyl sites, resulting in polymers that exhibit strong NIR emission.^[^ ^59^ ^]^

NIR materials are highly desirable for applications in both optoelectronics and biomedical fields due to the effective penetration of tissues by NIR light.^[^ ^61^, ^62^, ^63^, ^64^, ^65^ ^]^ Moreover, while still in early stages of development, electrochromic NIR materials are attracting tremendous current interest given their strong potential for advanced devices that operate in the NIR region.^[^ ^66^, ^67^, ^68^, ^69^, ^70^, ^71^ ^]^ We hypothesized that attaching additional electron deficient 4‐pyridyl heterocycles on the BDPA framework, followed by quaternization of the pendant pyridyl nitrogens, would bring the LUMO even further down, resulting in new low band gap material. In addition, we expected the quaternized derivatives to behave as viologen‐type materials with BDPA as a unique π‐conjugated linker unit. With its highly delocalized LUMO, BDPA was deemed to be especially effective in stabilizing the quinoidal doubly‐reduced form of viologens, providing a conduit for electronic delocalization.

Methylation of the pyridyl groups in **1Py** is shown to give rise to a dicationic species, [**1Py‐Me**](PF_6_)_2_ (Figure 1c). As an alternative quaternization strategy, we also explore the formation of a strong Lewis acid‐base adduct of **1Py** with B(C_6_F_5_)_3_ (BCF) to give the neutral complex **1Py‐BCF**. We demonstrate that quaternization very effectively reduces the LUMO energy, resulting in further narrowed band gaps and strong luminescence in the NIR region, and provide insights into the effect on the self‐sensitized reactivity toward oxygen. Stabilization of the LUMO promotes electrochemical transformation to reduced species. These processes are studied by spectroelectrochemical and computational methods, revealing the formation of doubly‐reduced quinoidal species [**1Py‐BCF]^2−^** and [**1Py‐Me**]^0^ with a closed shell electronic configuration. Prominent color changes are seen upon reduction of **1Py‐BCF** which prompted assembly of a prototype electrochromic device with **1Py‐BCF** as the redox‐active material.

## Results and Discussion

### Synthesis and Characterization

**Scheme 1:**
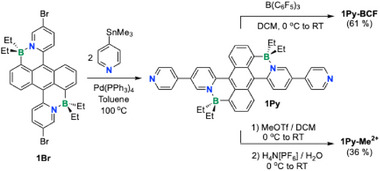
Synthesis of **1Py**, **1Py‐BCF**, and **1Py‐Me^2+^**.

The dibrominated BDPA derivative **1Br** was synthesized from 9,10‐bis(5‐bromopyridyl)anthracene following a previously reported procedure.^[^ ^59^ ^]^ **1Br** was then subjected to Stille coupling with 4‐(trimethylstannyl)pyridine to afford pyridyl‐functionalized **1Py**, which was purified by silica gel column chromatography and isolated by crystallization from chloroform/pentane in 60% yield (Scheme 1). Treatment of **1Py** with B(C_6_F_5_)_3_ in dichloromethane afforded the Lewis acid‐base complex **1Py‐BCF**, and quaternization with methyl triflate, followed by anion exchange with NH_4_PF_6_, generated the dicationic viologen [**1Py‐Me**](PF_6_)_2_ which will be referred to simply as **1Py‐Me^2+^**. The progress of these reactions was readily monitored by a change in color from purple to blue. All the newly synthesized compounds were characterized by multinuclear and 2D NMR, including 2D H,H‐COSY and H,H‐NOESY, as well as high‐resolution mass spectrometry. Compounds **1Py** and **1Py‐Me^2+^** showed a single peak around 0 ppm in the ^11^B NMR spectra corresponding to the tetracoordinate boron atoms of the BDPA moiety, whereas **1Py‐BCF** showed two peaks, one at 0.2 ppm corresponding to the boron atoms of the BDPA moiety and another at –3.2 ppm corresponding to the boron atoms of BCF.

**Figure 2:**
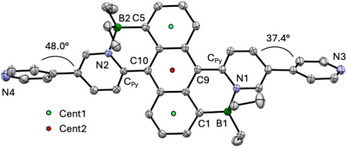
ORTEP plot of the X‐ray crystal structure of **1Py** viewed from above the anthracene backbone (50% thermal ellipsoids).

**1Py** was further characterized by single crystal X‐ray diffraction (SC‐XRD). Crystals were grown from chloroform solution upon slow diffusion of pentane vapor, however, suitable crystals for **1Py‐BCF** and **1Py‐Me^2+^** could not be obtained despite numerous attempts. The molecular structure of **1Py** is illustrated in Figure 2, and important geometric parameters are summarized in Table S1. The B─N bond distances of 1.634(2) and 1.645(2) Å are in the typical range and indicative of strong Lewis acid‐base interactions.

Similar to other BDPA derivatives,^[^ ^54^, ^55^, ^58^, ^59^ ^]^ **1Py** shows a curved anthracene backbone with an interplanar angle between the outer benzene rings of *ε* = 20.2°. The angle *ε* is somewhat smaller than that for the parent compound **1H** ^[^ ^58^ ^]^ (*ε* = 23.6°) and the methylated derivative **1Me** ^[^ ^59^ ^]^ (*ε* = 22.7°) but similar to that for vinyl‐substituted **1Vi** ^[^ ^59^ ^]^ (*ε* = 20.6°). A more detailed comparison of the structure of **1Py** with those of other BDPA derivatives reveals that the angles Cent1‐C_An_‐B (*β* = 173.1°, 174.1°) are relatively closer to 180° than in **1H** (*β* = 169.1°), **1Me** (*β* = 167.9°, 168.7°), and **1Vi** (*β* = 171.2°). Conversely, the angles Cent2‐C_An_‐C_Py_ (*γ* = 164.0°, 166.1°) are smaller than in **1H** (*γ* = 168.6°), **1Me** (*γ* = 166.2°, 166.3°), and **1Vi** (*γ* = 169.2°). This shows that for **1Py** the steric strain in the system is compensated by stronger bending of the pyridyl groups away from the anthracene backbone, whereas dislocation of the boron atoms relative to anthracene and bending of the anthracene itself is less pronounced. The rotation of the pyridyl groups relative to the core, expressed by the interplanar angle *ϕ* between the inner anthracene ring and the pendent pyridyl groups, is smaller for **1Py** (34.4, 36.4°) than **1H** (36.8°) and **1Me** (38.8, 39.4°), but larger than for **1Vi** (30.1°). Overall, the X‐ray crystal structure shows that **1Py** adopts a more planar molecular conformation than **1H** and **1Me**, similar to that of the π‐extended derivative **1Vi**, which should promote electronic delocalization. The interplanar angles between the terminal pyridyls (Py_t_) and the BDPA‐bound pyridyls (Py_B_) are 37.4° and 48.0°, which demonstrates the possibility of further delocalization into the terminal pyridyl moieties.

### Photophysical Properties and Computational Studies

**Figure 3:**
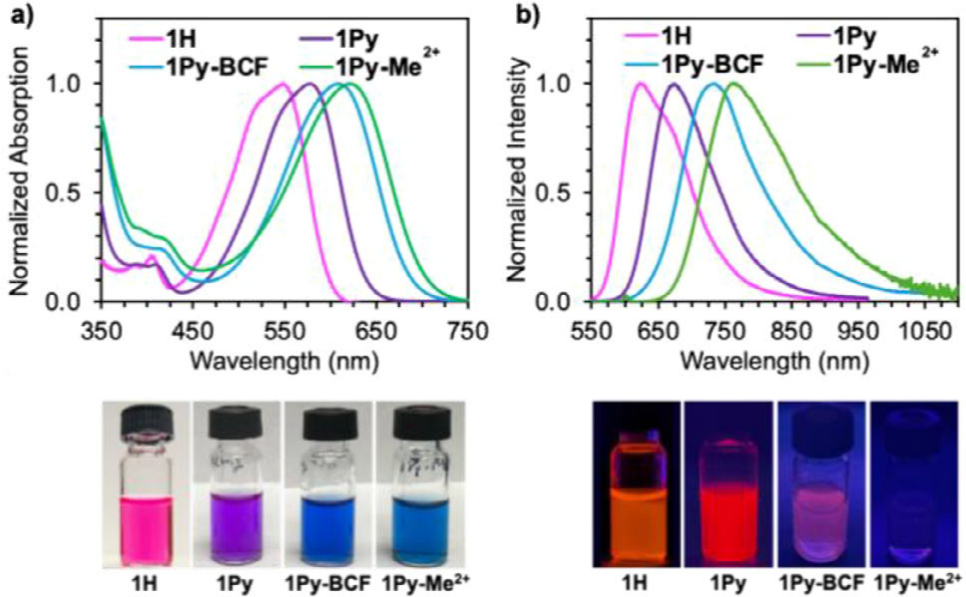
a) Absorption and b) emission spectra of **1Py**, **1Py‐BCF**, and **1Py‐Me^2+^** in comparison to parent compound **1H** in acetonitrile solution; photographs illustrate the solutions a) under ambient light and b) upon irradiation at 365 nm.

The absorption and fluorescence spectra of **1Py**, **1Py‐BCF**, and **1Py‐Me^2+^** in acetonitrile solution are illustrated in Figure 3 along with those of the parent compound **1H**, and the detailed data in acetonitrile as a highly polar and DCM as a less polar solvent are summarized in Table 1. Whereas **1H** is red colored in acetonitrile with an absorption maximum (*λ*_abs_) at 547 nm, that of **1Py** is bathochromically shifted to *λ*_abs_ = 577 nm. Further bathochromic shifts are seen upon borane complexation in **1Py‐BCF** (609 nm) and quaternization in **1Py‐Me^2+^** (619 nm). All the compounds are fluorescent, and the wavelength of maximum emission in acetonitrile is dramatically shifted from 623 nm for **1H** to 669 nm for **1Py**, 732 nm for **1Py‐BCF**, and 770 nm for **1Py‐Me^2+^**. These observations are in line with the hypothesis that attachment of electron‐withdrawing pyridine heterocycles to the BDPA framework lowers the LUMO energy and reduces the HOMO‐LUMO gap, resulting in red‐shifted absorptions and emissions. The effect is further enhanced by borane complexation and even more so by methylation, ultimately resulting in the observation of near‐IR emission for **1Py‐BCF** and **1Py‐Me^2+^**. A negative solvatochromic effect is seen in the absorption spectra of these compounds, indicating higher polarity in the ground in comparison to the excited state (Figures S48, S50, and S52). Conversely, only a very minor solvatochromic effect on the emission is observed. In accordance with the energy gap law, the fluorescence lifetime gradually decreases from 7.5 ns for **1Py** to 1.8 ns for **1Py‐Me^2+^**, and so does the quantum yield from 75.2% to 11.7%. Similar or slightly higher quantum yields are achieved for the neutral complexes in CH_2_Cl_2_ as a less polar solvent, in which **1Py‐Me^2+^** is poorly soluble (Table 1).

**Table 1:** Comparison of photophysical data in acetonitrile and CH_2_Cl_2_ solution.

Compd	Solvent	*λ*_abs,TDDFT_ ^a)^[nm]	*λ*_abs_ [nm]	*λ*_Fl,_ [nm]^b)^	Stokes Shift [cm^−1^]	*τ*_Fl,_ [ns]^c)^	*Φ*_Fl_ [%]^d)^	*k*_r_ ^e)^ [10^7^ s^−1^]	*k*_nr_ ^e)^ [10^7^ s^−1^]
**1H** ^f)^	CH_2_Cl_2_	529	560	629	1960	11.1	79.9	7.2	1.8
**1Py**	CH_2_Cl_2_	561	595	676	2014	7.5	77.5	10.3	3.0
**1Py‐BCF**	CH_2_Cl_2_	599	638	751	2358	3.1	24.6	7.9	24.3
**1H**	CH_3_CN	523	547	623	2230	9.8	71.0	7.2	3.0
**1Py**	CH_3_CN	554	577	669	2383	7.5	75.2	10.0	3.3
**1Py‐BCF**	CH_3_CN	589	609	732	2759	3.2	24.9	7.8	23.5
**1Py‐Me^2+^**	CH_3_CN	605	619	770	3168	1.8	11.7	6.5	49.1

^a)^ Computed at B3LYP/6–31G(d,p)//cam‐B3LYP/6–31G(d,p) level of theory with acetonitrile/dichloromethane solvation (counterions included for **1Py‐Me^2+^**).

^b)^ Excited at longest wavelength absorption maximum.

^c)^ Fluorescence lifetime.

^d)^ Fluorescence quantum yield determined using an integrating sphere.

^e)^ Radiative (*k*_r_) and non‐radiative (*k*_nr_) decay rate constants calculated using the equations *k*_r_ = *Φ* / *τ*, *k*_nr_ = (1 – *Φ*) / *τ*).

^f)^ Ref.[58]

**Figure 4:**
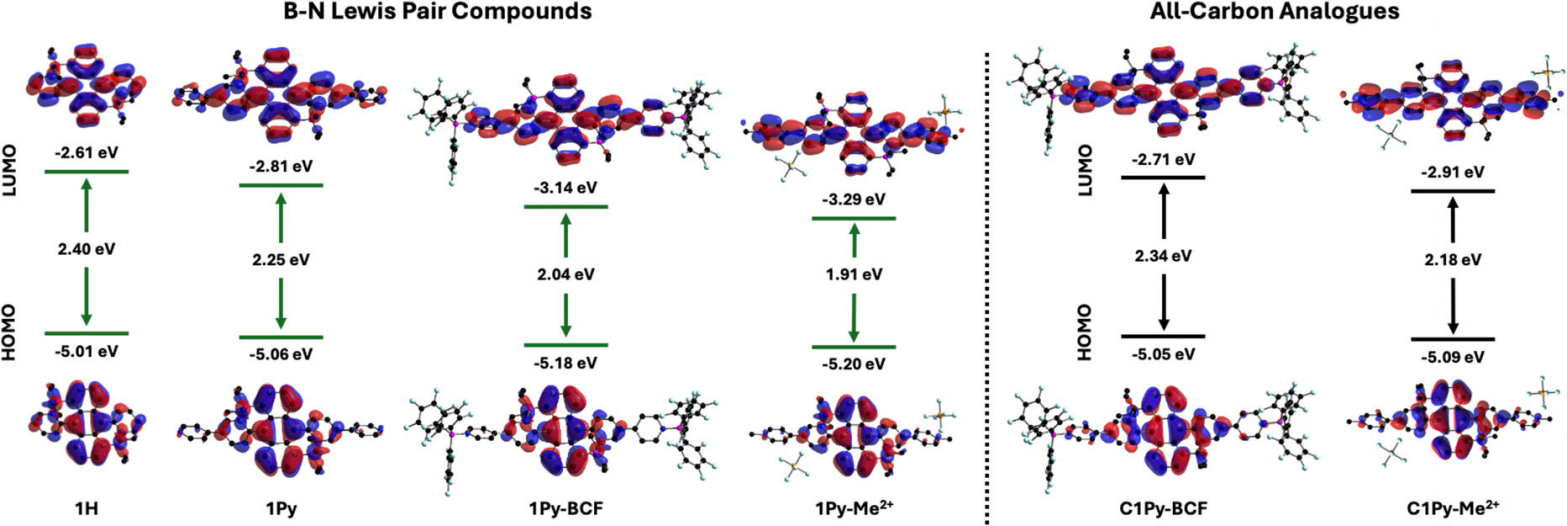
Computed frontier molecular orbital plots and energy levels of **1H**, **1Py**, **1Py‐BCF**, and **1Py‐Me^2+^** (with counterions) and the all‐carbon analogues **C1Py‐BCF** and **C1Py‐Me^2+^** (with counterions) with C─C in place of B─N units; theoretical calculations performed at the DFT/B3LYP/6–31G(d,p) (SCRF = acetonitrile) level.

To gain further insights into the nature of the absorption and emission bands, and the origin of the spectral shifts upon quaternization, density functional theory (DFT) and time‐dependent DFT (TD‐DFT) calculations were carried out. In addition, to assess the effect of B–N incorporation, analogous studies were conducted for the all‐carbon counterparts of the BN‐fused compounds. Consistent with the experimental observation of intense long wavelength absorptions, calculations at the B3LYP/6–31G(d,p)//cam‐B3LYP/6–31G(d,p) level of theory revealed strong S_0_→S_1_ transitions that are dominated by HOMO→LUMO excitation and increase in oscillator strength in the order of **1H **< **1Py** < **1Py‐Me^2+^  **≅ **1Py‐BCF** (Table 1 and Tables S12–S15). Frontier molecular orbital (FMO) analysis (Figure 4) revealed that the HOMO is centered primarily on the anthracene moiety with only small contributions from the boron‐bound pyridyls and virtually no contributions from the terminal pyridyls. Consequently, the functionalization at the peripheral pyridyls had only a modest effect on the HOMO energy level, which slightly decreased upon quaternization by BCF coordination and methylation. In contrast, for all BN fused compounds, the LUMO is distributed over both the anthracene and boron‐bound pyridyl moieties, reflecting a pronounced quinoidal character. Importantly, for **1Py**, **1Py‐BCF**, and **1Py‐Me^2+^** the LUMO extends into the pendent pyridyl moieties. The contributions from the peripheral pyridyl groups are most prominent for **1Py‐Me^2+^**. Accordingly, the LUMO energy levels greatly decrease, following the trend **1H **> **1Py** > **1Py‐BCF** > **1Py‐Me^2+^**. Overall, for the BN fused compounds the HOMO‐LUMO gap follows the trend **1H **> **1Py** > **1Py‐BCF** > **1Py‐Me^2+^**, thus corroborating the observed absorption and emission properties. Similar trends are deduced from computations on the all‐carbon analogues (Figure 4, Figures S77–S78). However, while the HOMO levels are generally shifted to slightly lower energy for the BN‐fused species compared to the all‐carbon analogs, the LUMO levels are markedly lower in energy by 0.38–0.50 eV, which should make their reduction much more facile (vide infra). Furthermore, replacement of CC with BN units leads to a significant lowering of the HOMO–LUMO gap, which is slightly more pronounced for **1Py‐BCF** (0.30 eV) than **1Py‐Me^2+^** (0.27 eV), explaining their low energy absorption and NIR emission profiles.

### Singlet Oxygen Sensitization

**Figure 5:**
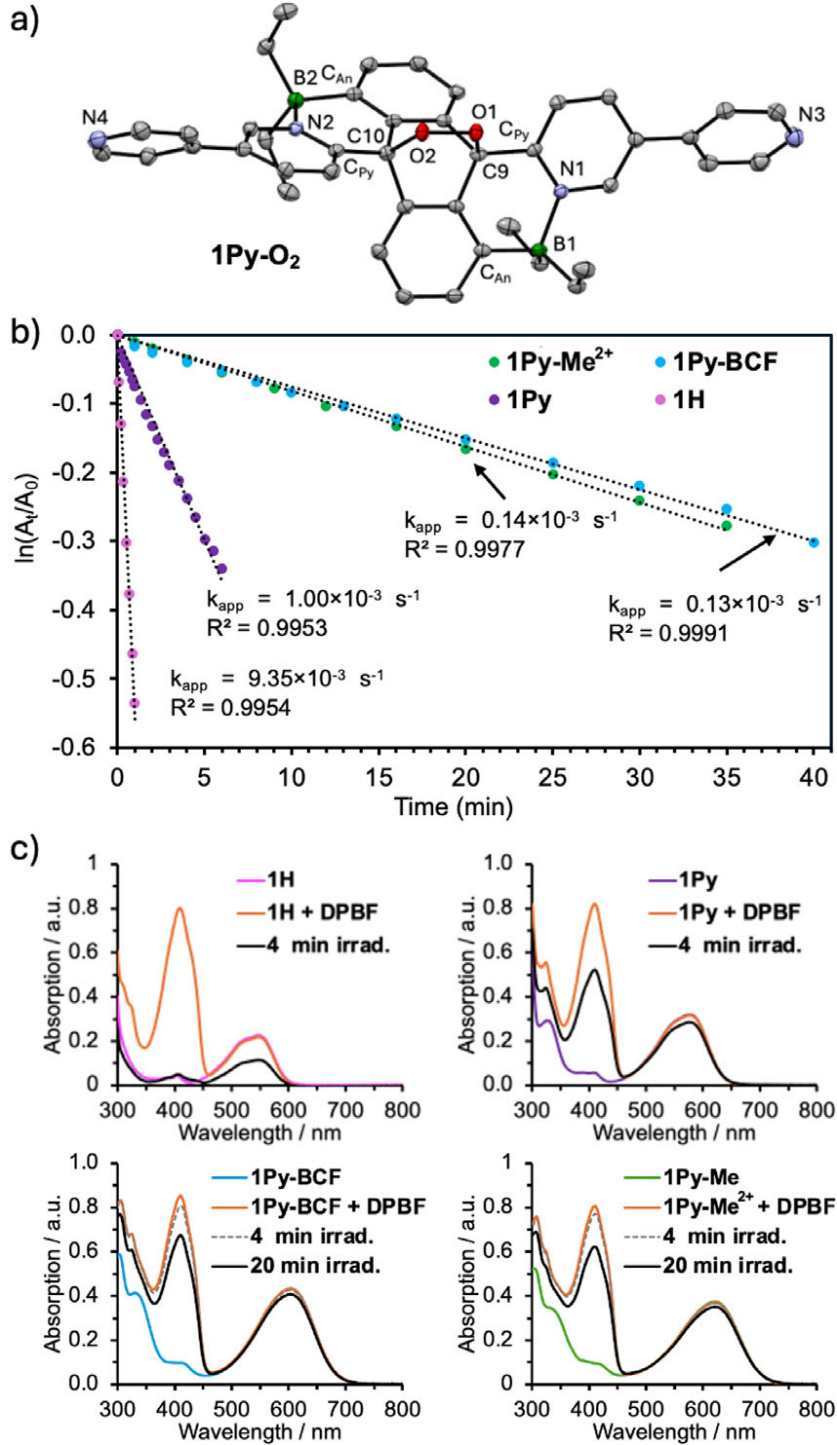
a) Plot of the X‐ray crystal structures of **1Py‐O_2_** (50% thermal ellipsoids, H atoms, and two cocrystallized CHCl_3_ solvent molecule omitted for clarity). b) Pseudo first‐order kinetics plot for the reaction of **1H**, **1Py**, **1Py‐BCF**, and **1Py‐Me^2+^** with oxygen upon photoirradiation with a Xe lamp to give the respective endoperoxides in acetonitrile (455 nm cut‐off filter was used, absorbance *A* at *λ*_max_). c) UV–vis spectral data taken before and after photoirradiation of solutions of **1H**, **1Py**, **1Py‐BCF**, and **1Py‐Me^2+^** (10 µM in ACN) by a Xe lamp at room temperature (34 Watt, 495 nm cutoff filter) in the presence of 1,3‐diphenylisobenzofuran (**DPBF**) (30 µM in ACN) as a singlet oxygen scavenger.

Our earlier investigations established that upon photoirradiation B–N fused anthracenes generate singlet O_2_ and form endoperoxides through 4 + 2 cycloaddition reaction with the generated singlet O_2_.^[^ ^54^, ^55^, ^57^, ^59^ ^]^ The endoperoxides in turn release singlet O_2_ at elevated temperature.^[^ ^72^, ^73^ ^]^ Singlet oxygen is an important reactive oxygen species seeing extensive use in organic synthesis, photodynamic therapy (PDT) as a cancer treatment, and materials science.^[^ ^74^, ^75^, ^76^, ^77^, ^78^, ^79^, ^80^ ^]^ Linker and coworkers have demonstrated the triggered release of singlet oxygen from pyridyl‐substituted anthracenes upon quaternization.^[^ ^73^, ^81^, ^82^ ^]^ This prompted us to further investigate the effect of attaching electron‐withdrawing pyridine and pyridinium functional groups at the periphery of BDPAs on their self‐sensitized reactivity toward singlet O_2_. Solutions in oxygen‐saturated acetonitrile were irradiated with a Xe lamp at room temperature, and the reaction progress to form the respective endoperoxides was monitored by UV–vis absorption spectral measurements. The formation of the corresponding endoperoxides was substantiated by 2D and multinuclear NMR and HRMS analyses of the isolated products and an X‐ray crystal structure analysis of **1Py‐O_2_** (Figure 5a). The ^1^H NMR spectra of the endoperoxides revealed characteristic doublet signals for the peri‐H atoms in the range of 6.47–6.58 ppm that are upfield shifted because of enhanced shielding in the strongly bent oxygenated species (Figures S23, S30, S37).^[^ ^54^, ^55^ ^]^

Kinetic studies revealed a trend where the reactivity decreases in the order of **1H **> **1Py** > **1Py‐Me^2+^** ≅ **1Py‐BCF** (Figure 5b). This trend can be explained by considering the position of the triplet energy level of these compounds. For rapid oxygenation to occur, the S_0_‐T_1_ energy gap should be higher than that of O_2_ at ca. 95 kJ mol^−1^, and the spin orbit coupling promoting intersystem crossing (ISC) has to be very efficient.^[^ ^83^, ^84^, ^85^ ^]^ The computed S_0_‐T_1_ energy gaps for **1Py‐BCF** (84.2 kJ mol^−1^) and **1Py‐Me^2+^** (80.6 kJ mol^−1^) are below this threshold and significantly lower than those of **1H** (97.2 kJ mol^−1^) and **1Py** (91.1 kJ mol^−1^) (Table S26). Although the HOMO energy may also affect the oxygenation efficiency,^[^ ^86^ ^]^ the computational data show a relatively small decrease from –5.01 eV for **1H** to –5.20 eV for **1Py‐Me^2+^**.

To further assess the generation of singlet oxygen and reactivity toward the B–N Lewis pair fused acenes, we performed competition experiments using 1,3‐diphenylisobenzofuran (**DPBF**) as a highly effective singlet oxygen trapping agent in 3‐fold excess (Figure 5c). When selectively irradiating reference compound **1H** over a period of 4 min at wavelengths >495 nm using a cut‐off filter, almost quantitative oxygenation of **DPBF** and ca. 50% oxygenation of **1H** was observed. The reactivity was greatly attenuated for **1Py** and further decreased for **1Py‐BCF** and **1Py‐Me^2+^** which required longer irradiation times of 20 mins for significant consumption of **DPBF** to be observed. These results are consistent with the reduced S_0_‐T_1_ energy gaps of these compounds. The residual reactivity may be an indication of (very limited) population of higher triplet states or other reaction pathways coming to bear.

The thermal release of oxygen was examined at 100 °C in DMF as the solvent. A more rapid reaction was seen for **1Py‐O_2_** in comparison to **1H‐O_2_**, which is consistent with its more electron‐deficient character (Figure S63). **1Py** could be recovered almost quantitatively (Figure S64), but similar experiments for **1Py‐BCF** and **1Py‐Me^2+^** led to poor recovery, indicating the possible involvement of other competing reaction pathways.

### Electrochemical Properties

**Figure 6:**
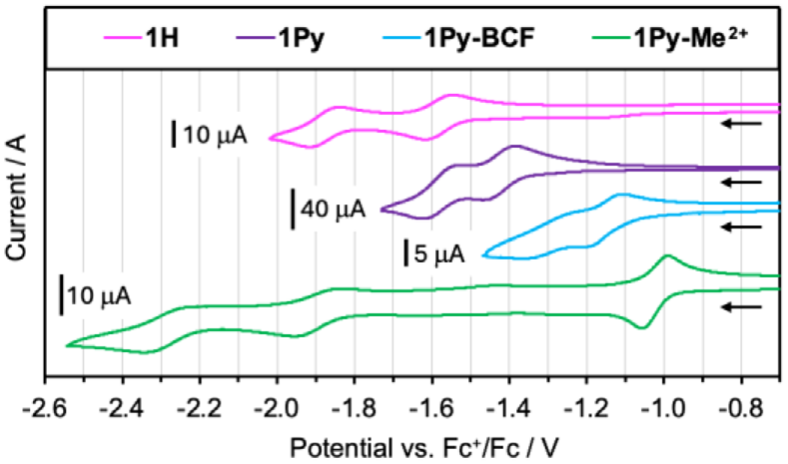
Cyclic voltammograms of **1H**, **1Py**, **1Py‐BCF**, and **1Py‐Me^2+^** at 200 mV/s in acetonitrile containing 0.1 M Bu_4_N[PF_6_].

Cyclic and square wave voltammetry studies offer additional insights into the electronic structure of the reported compounds. The data are summarized in Table 2, and the reduction waves obtained by cyclic voltammetry are illustrated in Figure 6. The overall trend for the 1st reduction potential of **1H **< **1Py** < **1Py‐BCF** < **1Py‐Me^2+^** is in good agreement with the computed LUMO levels, providing further evidence that π‐extension of BDPA with pyridine‐borane or pyridinium units stabilizes the LUMO. Whereas **1H** shows two reversible 1‐electron reduction waves at –1.58 and –1.88 V corresponding to the formation of the radical anion followed by the dianion, the corresponding reduction waves for **1Py** are shifted to less negative potentials at –1.42 and –1.58 V. Complexation with the strong Lewis acid BCF in **1Py‐BCF** further shifts the potentials to –1.16 and –1.32 V. Interestingly, the redox splitting between the first and second reduction decreases from 0.30 V for **1H** to 0.15 V for **1Py** and 0.16 V **1Py‐BCF**. This might be because the reduction processes for **1H** are more localized on the central anthracene unit but involve stronger orbital contributions from the pendent dipyridyl units in **1Py** and **1Py‐BCF**. In contrast, three reduction waves were observed for **1Py‐Me^2+^**. A reversible 2‐electron reduction wave (or 2 closely spaced 1‐electron reductions that cannot be resolved) at –1.02 V, which is even less negative than the first reduction of **1Py‐BCF**, is followed by two quasi‐reversible one‐electron reductions at much more negative potentials. Similar spectral features with an initial single‐step two‐electron reduction have been seen for other π–extended viologens containing two pyridinium moieties separated by *para*‐phenylene (–1.29 V in MeCN/Et_4_N[ClO_4_]), *para*‐biphenylene (–1.48 V in THF/Bu_4_N[PF_6_] versus Fc/Fc^+^), 2,2′‐bithiophene (the 2,5‐thiophene‐bridged derivative gives 2 waves), or 2,7‐disubstituted pyrene (–1.47 V in THF/Bu_4_N[PF_6_] versus Fc/Fc^+^), albeit at significantly more negative potentials.^[^ ^14^, ^20^, ^87^, ^88^, ^89^ ^]^ Although strong electronic communication may be expected to promote splitting of this band into separate waves, an alternative explanation may be that after initial reduction to generate the radical species, formation of a closed shell doubly reduced quinoidal species upon injection of a second electron is energetically favorable. The 2nd and 3rd reduction for **1Py‐Me^2+^** occur at much more negative potentials of –1.90 and −2.29 V, indicating that further reduction is considerably less favorable. However, they are less negative than those of other viologens with *para*‐biphenylene^[^ ^14^ ^]^ (−2.49 and −2.86 V) or 9,10‐diethynylanthracene^[^ ^21^ ^]^ (–2.16 and –2.69 V) as the bridge, reflecting the more electron‐deficient character of the borylated BDPA linker. As such, the 2nd and 3rd reduction plausibly correspond to the generation of a monoanionic and ultimately dianionic species by further reduction of the BDPA moiety. This is substantiated by the fact that the reductions of the parent reference compound **1H** occur at slightly less negative potentials and show a similar redox splitting.

**Table 2:** Cyclic voltammetry data and comparison of electrochemical results from DFT calculations and UV/Vis absorption spectroscopy.

Compound	E_red1,2,3_ [V] ^a)^	Δ*E*_red1‐2_ [V] ^a)^	*E* ^CV^_LUMO_ [eV] ^b)^	*E* ^DFT^_LUMO_ [eV] ^c)^	*E* ^DFT^_HOMO_ [eV] ^c)^	Δ*E* ^DFT^ [eV] ^c)^	Δ*E*_exp,_^UV^ [eV] ^d)^
**1H**	−1.58, −1.88	0.30	−3.22	−2.61	−5.01	2.40	2.27
**1Py**	−1.43, −1.58	0.15	−3.37	−2.81	−5.06	2.25	2.15
**1Py‐BCF**	−1.16, −1.32	0.16	−3.65	−3.14	−5.18	2.04	2.04
**1Py‐Me^2+^**	−1.02 (2 e), −1.90, −2.29	n. o. 0.39 (Δ*E*_red2‐3_)	−3.78	−3.29	−5.20	1.91	1.99

^a)^ Derived from CV data, quasi‐reversible or reversible, *E*_red_ = 0.5 (*E*_pc_ + *E*_pa_).

^b)^ *E*_LUMO_ = −(4.8 + *E*_red_).

^c)^ From DFT calculations (B3LYP/6–31G(d,p); solvated (SCRF = acetonitrile) model.

^d)^ Estimated from absorption maxima.

### Spectroelectrochemistry and Electrochromic Properties

**Figure 7:**
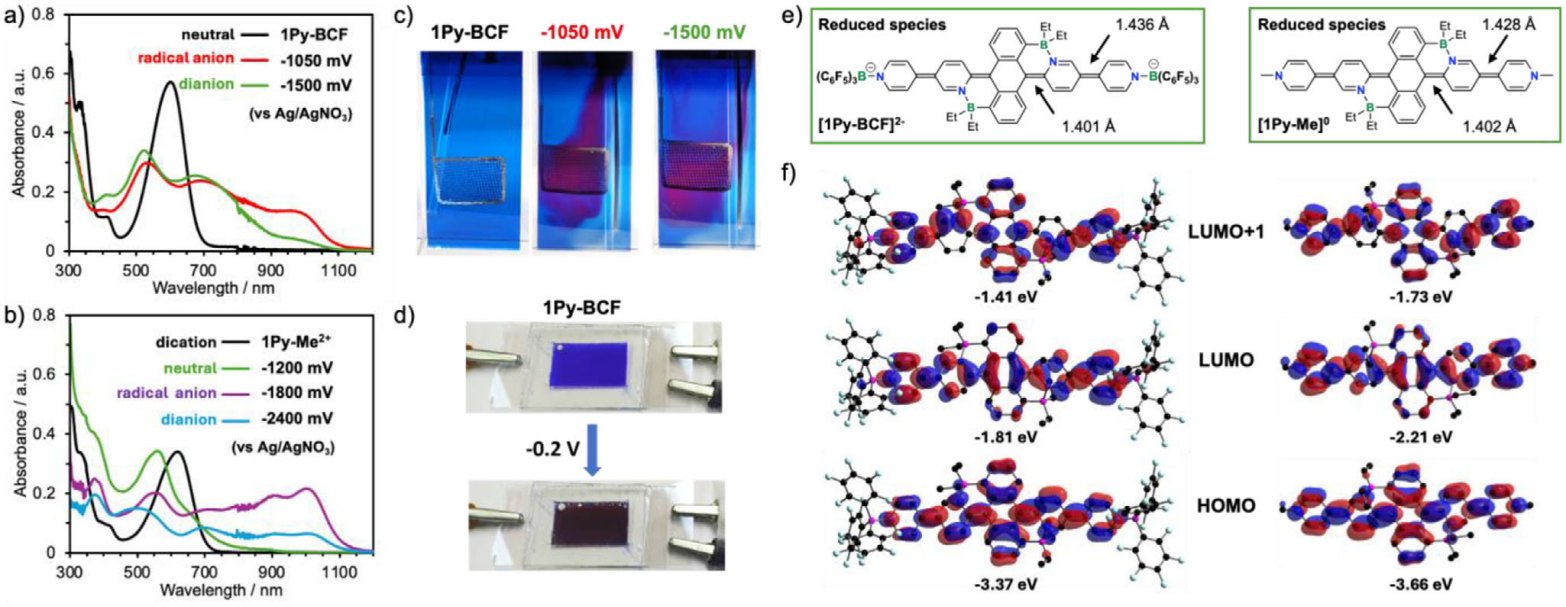
a), b) Spectroelectrochemical data for **1Py‐BCF** and **1Py‐Me^2+^**. c) Photographs illustrating the color changes upon sequential reduction of **1Py‐BCF** in DMF solution. d) Photograph of a prototype electrochromic device based on **1Py‐BCF** in acetonitrile solution, employing two ITO‐coated glass electrodes and tetrabutylammonium hexafluorophosphate as the supporting electrolyte. e) Quinoid resonance structures of doubly reduced **[1Py‐BCF]** ^2^ **^−^** and **[1Py‐Me]^0^** with selected computed bond lengths. f) Computed frontier molecular orbital plots and energy levels of doubly reduced species **[1Py‐BCF]^2−^** (left) and **[1Py‐Me]^0^** (right) from DFT calculations (B3LYP/6–31g(d,p), SCRF = acetonitrile).

The facile first reversible reduction of **1Py‐BCF** and **1Py‐Me^2+^** at potentials similar to that of the directly linked viologen paraquat (‐1.09 V, second reduction at –1.52 V versus Fc^+^/Fc),^[^ ^6^ ^]^ inspired us to explore the electrochromic properties by spectroelectrochemical UV–vis/NIR studies. Drastic changes were seen in the absorption spectra of **1Py‐BCF** upon applying a potential of –1050 mV (versus Ag/AgNO_3_), which is just beyond the 1st electrochemical reduction (Figure 7a). The band centered at 609 nm for the neutral complex vanished and several new broad bands appeared spanning the entire visible range and into the near‐IR with maxima at 401, 531, 688, and a shoulder at ca. 1000 nm (Table S18). The large red shift into the near‐IR region is a strong indication of the formation of a radical species, **1Py‐BCF^−^**. Upon further increasing the applied potential to –1500 mV, corresponding to the 2nd reduction, the intensity of the near‐IR band at 1000 nm band diminished indicating a change in redox state, i.e., formation of the dianionic species **1Py‐BCF^2−^**. When a Pt‐mesh working electrode was used, a distinct color change from deep blue to purple was visually observed upon the 1st reduction (Figure 7c). As the 2nd reduction primarily involves disappearance of the near‐IR absorption band significant changes in color were not visually observed at the higher potential. In case of the dicationic viologen‐type **1Py‐Me^2+^**, the band at 619 nm gradually disappeared upon applying a potential of –1200 mV corresponding to the single step 2‐electron reduction and a new band emerged at 558 nm with a shoulder at ca. 654 nm that is attributed to formation of the neutral species **1Py‐Me^0^** (Figure 7b, Table S24). Although two distinct regimes were identified with an initial decrease in intensity for the band at 619 nm and only later emergence of the new band at 558 nm, an intermediate singly reduced radical species (**1Py‐Me^+.^**) could not be confidently identified as near‐IR absorption bands could not be detected (Figure S74d). When the applied potential was increased to –1800 mV, corresponding to the 2nd reduction, multiple red shifted absorption bands appeared at 557, 696, 906, and 1002 nm, indicating formation of a radical anion, **1Py‐Me^−^**. Further reduction at –2400 mV led to a modest decrease in absorbance of the longest wavelength bands with only slight wavelength shifts. The visible color changes upon sequential reduction of **1Py‐Me^2+^** when using a Pt‐mesh electrode were not as striking as for **1Py‐BCF** because the shifts in the visible absorptions were more modest and masked by the appearance of overlapping blue and red‐shifted bands (Figure S74c).

The more pronounced color changes in the visible region observed upon electrochemical reduction of **1Py‐BCF** prompted us to further investigate this complex as an electrochromic material. A proof‐of‐concept device for **1Py‐BCF** using ITO‐coated glass surface was fabricated following literature reported techniques.^[^ ^90^ ^]^ As illustrated in Figure 7d, upon applying a negative potential a distinct color change from blue to deep purple was apparent, consistent with formation of the dianionic species **1Py‐BCF^2−^**. However, unlike in the spectroelectrochemical studies, the electrochromic switching was not fully reversible, and quantitative performance parameters could not be reliably obtained.

Computational studies suggested that the doubly reduced species **1Py‐BCF^2−^** and **1Py‐Me^0^** adopt a closed‐shell singlet configuration, which is consistent with their description as quinoid species (Figure 7e). A comparison of the computed bond lengths between the central anthracene and internal pyridyl ring (C(An)‐C(Py_B_)) as well as the internal and terminal pyridyls (C(Py_B_)‐C(Py_t_)) before and after reduction supports this interpretation (Table S7). For the doubly reduced species **1Py‐BCF^2−^** these bonds are significantly shortened (average of 1.401 versus 1.466 Å for C(An)‐C(Py_B_) and 1.436 versus 1.475 Å for C(Py_B_)‐C(Py_t_) in the parent compound). The bond lengths for **1Py‐Me^2+^** similarly decrease upon reduction (average of 1.402 versus 1.468 Å for C(An)‐C(Py_B_) and 1.428 versus 1.473 Å for C(Py_B_)‐C(Py_t_) in the parent compound). At the same time, the average angles between internal (Py_B_) and terminal (Py_t_) pyridyl rings dramatically decrease from 31.0° to 7.4° for **1Py‐BCF^2−^** and from 29.4° to 5.8° **1Py‐Me^0^**. Furthermore, the bending of the central anthracene unit becomes more prominent with angles between the outer phenyl ring planes increasing from 22.2° to 30.2° for **1Py‐BCF^2−^** and 22.2° to 30.1° for **1Py‐Me^0^**. Collectively these parameters are consistent with adoption of an extended quinoidal structure with significant double bond character between the *ipso*‐carbons of anthracene and the internal pyridine, and between the internal and terminal pyridine rings. We also note that further reduction of **1Py‐Me^0^** to **1Py‐Me^2−^** reverses some of these trends as the anthracene bending becomes less prominent and the C─C bonds between anthracene and the internal pyridyl rings become longer again; however, the quinoidal character of the bis(pyridyl) units is further strengthened as indicated by even shorter average C(Py_B_)‐C(Py_t_) bonds of 1.410 Å and smaller angles between the pyridyls of 3.6° (Table S7). This suggests that the bis(pyridyl) units upon further reduction become increasingly decoupled from the anthracene core. The B─N bond lengths for the BDPA units slightly decrease upon reduction, indicating that the strong intramolecular Lewis acid‐base interactions are retained. A more dramatic effect is seen for the B─N distances to the B(C_6_F_5_)_3_ moieties in **1Py‐BCF**. They become significantly shorter with each reduction step from 1.650 to 1.623 and 1.605 Å, suggesting that the reduction is strengthening the B─N interaction because the Lewis basicity of the terminal pyridyl moieties is enhanced.

TD‐DFT calculations on **1Py‐BCF^2−^** predict a lowest energy transition at 701 nm that is primarily H→L in nature, which is accompanied by a slightly higher energy transition at 559 nm that is primarily H→L + 1 in nature (Figure 7f, left side; Figure S81, Table S17). The positions of these transitions are consistent with those of the experimentally observed bands at 522 and 674 nm deduced from the spectroelectrochemical studies. Similar low energy transitions are also predicted by TD‐DFT calculations for **1Py‐Me^0^** at 736 (H→L) and 569 nm (H→L + 1) (Figure 7f, right side; Figure S82, Table S20), and again these are in reasonably good agreement with the bands that are detected by spectroelectrochemistry at 558 and 654 nm (shoulder).

## Conclusion

We demonstrate that attachment of pyridine, pyridine‐borane, and N‐methylpyridinium groups to B─N fused dipyridylanthracene (BDPA) results in increasingly large reductions in the LUMO without significantly affecting the HOMO energy levels. Consequently, pronounced bathochromic shifts of the absorption bands are seen, reflected in color changes from purple to blue and blue‐green, and emissions in the near‐IR region for the quaternized species. At the same time, quaternization of **1Py** leads to decreases in the triplet state energies, reducing the propensity for formation of the respective endoperoxides when exposed to visible light.

The dramatic decreases in the LUMO levels also influence the electrochemical properties. Upon quaternization through Lewis pair formation with BCF the reduction waves shift to less negative potentials in **1Py‐BCF** while retaining the 2‐step process with separate 1‐electron reduction waves seen for **1Py**. Methylation in **1Py‐Me^2+^** leads to a further anodic shift, but now the individual reduction processes cannot be resolved. For **1Py‐Me^2+^** two additional 1‐electron reduction waves are seen at more negative potentials indicating that even the corresponding triply reduced radical anion and quadruply reduced dianion can be accessed. All these reduction processes are accompanied by distinct color changes. For the doubly reduced species, **1Py‐BCF^2−^** and **1Py‐Me** ^0^, strong absorption bands are seen in the visible spectral region. Taking advantage of the striking color change from blue to deep purple for **1Py‐BCF,** a prototype electrochromic device was fabricated. For the doubly reduced species, closed‐shell configurations were confirmed by computational studies, which revealed a pronounced quinoidal character with extensive delocalization indicated by short C─C bonds between anthracene and the boron‐bound pyridyls, as well as between pyridyl groups. These findings further indicate that the facile reduction of **1Py‐BCF** and **1Py‐Me** ^2+^ is promoted by strong electronic delocalization in the doubly reduced species. For the radical anion species, the absorptions reach far into the near‐IR spectral region, suggesting potential utility as sought after electrochromic device materials that operate in the near‐IR region. Future work will focus on strategies to further stabilize the reduced species, optimize the device architecture, and explore the near‐IR characteristics.

## Supporting Information

The authors have cited additional references within the Supporting Information and deposited X‐ray data with the joint Cambridge Crystallographic Data Centre and Fachinformationszentrum Karlsruhe.^[^ ^91^, ^92^, ^93^, ^94^, ^95^, ^96^ ^]^

## Conflict of Interests

The authors declare no conflict of interest.

## Supporting information



Supporting Information

Supporting Information

## Data Availability

The data that support the findings of this study are available in the Supporting Information of this article.
