# Supplementary material for: B‐N Fused Anthracene as Functional Linker for π‐Extended Viologens: Near‐IR Emission and Electrochromism
Source: Angew Chem Int Ed Engl. 2025 Dec 10;65(5):e21634. doi: 10.1002/anie.202521634 (PMC12851006; doi:10.1002/anie.202521634)
Supplement: Supplementary file 1 — Supporting Information [file ANIE-65-e21634-s002.pdf]

Supporting Information

for

B-N Fused Anthracene as Functional Linker for π-Extended Viologens: Near-IR Emission and Electrochromism

Rajendra Prasad Nandi,[a] Jingyao Zuo,[a] Abhishek Shibu,[b] and Frieder Jäkle[a]*

[a] Department of Chemistry, Rutgers University-Newark,

73 Warren Street, Newark, NJ 07102, USA

[b] HORIBA Instruments Incorporated

20 Knightsbridge Road, Piscataway, New Jersey, USA 08854

* Corresponding Author: Frieder Jäkle (email: fjaekle@rutgers.edu)

Table of Contents

General Experimental Procedures	S3-S4
Synthetic Procedures and Spectral Data	S4-S6
NMR and HRMS Characterization Data	S7-S32
Single Crystal XRD Data	S33
Photophysical Properties	S34-S42
Kinetics of Photo-Induced Endoperoxide Formation	S43-S44
Kinetics of the Endoperoxide Thermolysis	S45
Experiments with 1,3-Diphenylisobenzofuran (DPBF) as Singlet Oxygen Scavenger	S46-S48
Electrochemical Studies	S49-S50
Spectroelectrochemistry Studies	S51-S52
Computational Studies	S53-S110
References	S111

General Experimental Procedures

All the reactions were carried out using standard Schlenk techniques under positive pressure of pre-purified nitrogen gas. Dichloromethane was dried over CaH_2 and degassed by freeze-pump-thaw method. Precursor **1Br** and 4-(trimethylstannyl)pyridine were synthesized according to the previously published procedures.[1-2] All other reactants, reagents and solvents were purchased from commercial sources and used without further purification. 500.2 MHz ^{1}H NMR, 125.8 MHz ^{13}C NMR, 160.3 MHz ^{11}B NMR, 470.2 MHz ^{19}F NMR, 202.5 MHz ^{31}P NMR ^{1}H-^{1}H gCOSY, ^{1}H-^{1}H NOESY, and ^{1}H-^{13}C HSQC spectra were recorded on a Bruker Advance 500 MHz NMR spectrometer. ^{11}B NMR spectra were acquired with boron-free quartz NMR tubes using a boron-free 5 mm PH SEX 500S1 ^{11}B-H/F-D probe. 150.8 MHz ^{13}C NMR spectra were recorded on a Varian Inova 600 MHz NMR spectrometer. ^{1}H NMR and ^{13}C NMR spectra were referenced internally to the residual solvent signals, whereas ^{11}B signals were referenced to external $BF_3{\cdot}OEt_2$. Chemical shift multiplicities are reported as singlet (s), doublet (d), triplet (t), heptet (h), multiplet (m), and broad (br), and the abbreviations Py_t (terminal pyridyl), Py_B (boron-bound pyridyl), and An (anthracene) are used for signal assignments. High resolution mass spectrometry (HRMS) data were obtained by electrospray ionization (ESI) in positive or negative mode on an Apex Ultra 7.0 Hybrid FTMS or an Orbitrap Exploris 240 instrument.

Single crystals of **1Py** suitable for X-ray analysis were grown from $CHCl_3$/pentane solvent using vapour diffusion method at room temperature. Single crystals of **1Py-O$_2$** were obtained by slow evaporation of a chloroform solution at room temperature. X-ray diffraction intensities were collected at 100 K using a Rigaku XtaLAB Synergy-S Dual Source diffractometer equipped with a PhotonJet Cu-microfocus (λ = 1.54178 Å) and a HyPix-6000HE detector. The structure was solved by the intrinsic phasing method with SHELXT and refined by full-matrix least-squares techniques against F^2 (SHELXL) in the Olex2 graphical user interface.[3-4] Non-hydrogen atoms were refined with anisotropic displacement coefficients, and hydrogen atoms were treated as idealized contribution. Crystallographic data has been deposited with the Cambridge Crystallographic Data Center as supplementary publications with CCDC 2485272 (**1Py**) and 2505729 (**1Py-O$_2$**). Copies of the data can be obtained free of charge on application to CCDC, 12 Union Road, Cambridge CB2 1EZ, UK (fax: (+44) 1223-336-033; email: deposit@ccdc.cam.ac.uk).

A Varian Cary 5000 UV-Vis/NIR spectrophotometer was used for absorption measurements. Steady-state fluorescence emission spectra were collected using either a Horiba Fluorolog-3 (measurements to 850 nm) or a HORIBA Nanolog spectrofluorometer (measurements to 1100 nm). In both cases, a 450 W Xenon lamp was used for excitation and passed through double-grating monochromators for high stray-light rejection. On the Fluorolog, the fluorescence signal was collected at right angle by a Hamamatsu R928 PMT detector and on the Nanolog using a HORIBA iHR320 double-grating monochromator by a Synapse Plus CCD detector which was TE-cooled to -80 °C. For fluorescence quantum yield measurements, a HORIBA QuantaPhi-2 integrating sphere was coupled to the Nanolog and a neutral density filter of OD=1 was utilized on the excitation side to attenuate the Rayleigh scatter to match the detector's linear dynamic range. The experimental set-up was validated by testing a Rhodamine-101 quantum yield reference standard obtained from Starna Scientific. TCSPC measurements were carried out using the HORIBA Fluorolog-3 spectrofluorometer equipped with a 590 nm HORIBA NanoLED pulsed excitation source. Photon counting electronics was controlled by a HORIBA FluoroHub and photons were detected using a Hamamatsu R928 PMT detector.

Cyclic voltammetric measurements were performed using a three electrode CV cell with a CV-50 W analyzer from BASi. An Au disk electrode was used as working electrode, a Pt wire as

counter electrode, and an Ag wire as reference electrode. Cyclic voltammograms were recorded in acetonitrile solution with $[Bu_4N][PF_6]$ (0.1 M) as supporting electrolyte. The potential scale was referenced to the ferrocene/ferrocenium couple. Spectroelectrochemical studies were conducted using a Varian Cary 5000 UV-Vis/NIR spectrophotometer combined with a Honeycomb Spectroelectrochemical Electrode – Gold (Au) (Pine Research Instrumentation) and a $Ag/AgNO_3$ (in acetonitrile) reference electrode inside a Quartz Cuvette (Pine Research Instrumentation). All the measurements were performed in acetonitrile solution with $[Bu_4N][PF_6]$ (0.1 M) as supporting electrolyte.

Density functional theory (DFT/TD-DFT) calculations were performed using the B3LYP functional with a 6-31G(d,p) basis set as incorporated in the Gaussian 16 package for all the atoms, mixing the exact Hartree-Fock-type exchange with Becke's exchange functional and that proposed by Lee-Yang-Parr for the correlation contribution.[5] The optimized structures and the frontier molecular orbitals (FMOs) and energy levels were analysed using Gaussview 6.0.[6] Frequency calculations were performed to confirm the presence of local minima (only positive frequencies). Vertical excitations were calculated by TD-DFT methods at the cam-B3LYP/6-31G(d,p) level.

Synthetic Procedures and Spectral Data

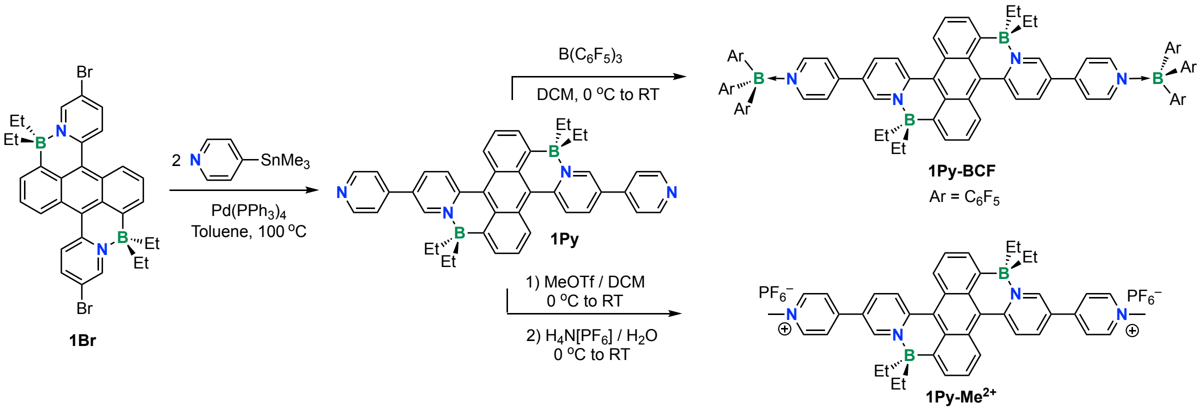

Scheme S1. Synthesis scheme for compounds **1Py**, **1Py-BCF**, and **1Py-Me^{2+}**.

Synthesis of **1Py**

Compound **1Br** (0.80 g, 1.28 mmol), 4-(trimethylstannyl)pyridine (1.24 g, 5.13 mmol, 4.0 equiv) and $Pd(PPh_3)_4$ (0.22 g, 0.19 mmol, 0.15 equiv) were added to 50 mL of dry degassed toluene in a Schlenk flask and the mixture heated at 100 °C for 48 hrs. The solution was concentrated under reduced pressure. The crude product was purified by recrystallization using chloroform/pentane to give the pure product as a dark purple crystalline solid. Yield: 0.48 g (60 %). **^{1}H NMR** (500.2 MHz, $CDCl_3$, 25 °C): δ (ppm) = 9.09 (s, 2H, Py_B), 8.84 (d, J = 4.2 Hz, 4H, Py_t), 8.48 (d, J = 8.5 Hz, 2H, Py_B), 8.16 (m, 4H, Py_B & An), 7.67 (d, J = 6.4 Hz, 2H, An), 7.63 (d, J = 4.2 Hz, 4H, Py_t), 7.56 (t, J = 7.5 Hz, 2H, An), 1.43 (br, 2H, B-CH_2), 1.14 (br, 2H, B-CH_2), 0.82 (br, 6H, CH_3), 0.52 (br, 10H, CH_3 & B-CH_2). **^{13}C{^{1}H} NMR** (125.8 MHz, $CDCl_3$, 25 °C): δ (ppm) = 153.0, 151.2, 149.8 (br, B-C), 143.5, 143.1, 135.6, 133.0, 132.4, 130.1, 128.9, 128.8, 128.7, 127.9, 121.2, 120.6, 23.8 (br), 13.8 (br), 10.4 (br), 9.3 (br). **^{11}B NMR** (160.3 MHz, $CDCl_3$, 25 °C): δ (ppm) = 0.5.

Synthesis of **1Py-BCF**

Compound **1Py** (0.10 g, 0.16 mmol) was dissolved in 20 mL of dry degassed dichloromethane and the solution cooled to 0 °C using an ice bath. Tris(pentafluorophenyl)borane (0.21 g, 0.41 mmol, 2.6 equiv) was dissolved in 10 mL of dichloromethane and the solution added dropwise to the reaction mixture, warmed to room temperature and stirred for 24 hrs. The solvent was removed under reduced pressure, and the residue was washed with hexanes (10 mL x 3). The crude product was purified by repeated precipitation at -20 °C using dichloromethane/hexane to obtain the pure product as a dark blue powder. Yield: 0.17 g (61 %). **^{1}H NMR** (500.2 MHz, $CDCl_3$, 25 °C): δ (ppm) = 9.20 (s, 2H, Py_B), 8.78 (d, *J* = 4.8 Hz, 4H, Py_t), 8.59 (d, *J* = 8.3 Hz, 2H, An), 8.24 (d, *J* = 8.8 Hz, 2H, Py_B), 8.14 (d, *J* = 8.3 Hz, 2H, Py_B), 7.98 (d, *J* = 5.4 Hz, 4H, Py_t), 7.70 (d, *J* = 6.6 Hz, 2H, An), 7.61 (t, *J* = 7.4 Hz, 2H, An), 1.2-0.8 (br, 10H, CH_3 & B-CH_2 overlapped with residual hexanes), 0.52 (br, 10H, CH_3 & B-CH_2). **^{13}C{^{1}H} NMR** (150.8 MHz, $CDCl_3$, 25 °C): δ (ppm) = 155.1, 149.6 (br, B-C), 149.2, 148.0 (d, *J*(^{19}F,^{13}C) = 255 Hz), 147.9, 144.2, 140.6 (d, *J*(^{19}F,^{13}C) = 270 Hz), 137.5 (d, *J*(^{19}F,^{13}C) = 272 Hz), 135.6, 133.3, 130.6, 129.6, 129.3 (two carbons), 128.7, 122.6, 117.7 (br, B-C), 29.9, 14.0, 9.9, 8.3, one quaternary carbon not observed due to overlap. **^{11}B NMR** (160.3 MHz, $CDCl_3$, 25 °C): δ (ppm) = 0.2, -3.2 (overlapped). **^{19}F NMR** (470.2 MHz, $CDCl_3$, 25 °C): δ (ppm) = -131.23 (br d, *J* (^{19}F, ^{19}F) = 16.0 Hz), -155.54 (t, *J* (^{19}F, ^{19}F) = 20.5 Hz), -162.34 (br t, *J* (^{19}F, ^{19}F) = 18.3 Hz).

Synthesis of **1Py-Me^{2+}**

Compound **1Py** (0.20 g, 0.32 mmol) was dissolved in 20 mL of dichloromethane under nitrogen atmosphere and the solution cooled to 0 °C using an ice bath. Methyl triflate (0.26 g, 1.58 mmol, 4.9 equiv) was added to the solution dropwise, the reaction mixture warmed to room temperature and stirred for 24 hrs. A dark blue precipitate formed. The supernatant was decanted, and the residue was washed with dichloromethane (2 x 5 mL) and dried under vacuum. The crude product was redissolved in 10 mL of methanol and added dropwise to a cooled (0 °C) solution of ammonium hexafluorophosphate (1.05 g, 6.44 mmol, 20 equiv) in water (500 mL) with stirring. After stirring for 1 hour, the mixture was filtered and the solid collected, which was washed with water and dried in air. The crude product was purified by repeated reprecipitation using acetone/water. Yield: 0.11 g (36 %). **^{1}H NMR** (500.2 MHz, CD_3CN, 25 °C): δ (ppm) = 9.25 (d, *J* = 2.0 Hz, 2H, Py_B), 8.76 (d, *J* = 7.0 Hz, 4H, Py_t), 8.55 (d, *J* = 8.6 Hz, 2H, Py_B), 8.51 (dd, *J* = 8.6 Hz, 2.1 Hz, 2H, Py_B), 8.39 (d, *J* = 6.9 Hz, 4H, Py_t), 8.23 (dd, *J* = 8.5 Hz, 0.8 Hz, 2H, An), 7.69 (d, *J* = 6.0 Hz, 2H, An), 7.63 (dd, *J* = 6.8 Hz, 6.7 Hz, 2H, An), 4.37 (s, 6H, N-Me), 1.39 (br, 2H, B-CH_2), 1.26 (br, 2H, B-CH_2), 0.74-0.48 (br, 16H, CH_3 & B-CH_2). **^{13}C{^{1}H} NMR** (125.8 MHz, CD_3CN, 25 °C): δ (ppm) = 155.0, 152.0, 150.3 (br, B-C), 146.8, 145.5, 138.7, 133.9, 131.6, 130.4, 130.2, 129.8, 129.1, 126.4, 121.6, 49.0, 24.1, 14.4, 10.7, 9.7, one quaternary carbon not observed due to overlap. **^{11}B NMR** (160.5 MHz, CD_3CN, 25 °C): δ (ppm) = 0.6. **^{19}F NMR** (470.2 MHz, CD_3CN, 25 °C): δ (ppm) = -73.0 (d, ^{1}*J*(^{19}F, ^{31}P) = 706.5 Hz), **^{31}P NMR** (202.5 MHz, CD_3CN, 25 °C): δ (ppm) = -144.6 (h, ^{1}*J*(^{31}P, ^{19}F) = 706.3 Hz).

Synthesis of **1Py-O_2**

Compound **1Py** (10.0 mg, 0.016 mmol) was dissolved in 5 mL of anhydrous oxygen-saturated dichloromethane in a vial. The solution was irradiated by sunlight at room temperature, resulting in complete decoloration within ca. 0.5 hours. After removal of the solvent, the crude product was redissolved in a minimum amount of dichloromethane and carefully layered with hexanes (v/v = 1:1) at room temperature. Diffusion and slow partial evaporation of the solvents gave **1Py-O_2** as pale yellow solid. Yield: 9.0 mg (86%). **^{1}H NMR** (500.2 MHz, $CDCl_3$, 25 °C): δ (ppm) = 9.17 (s, 2H, Py_B), 8.89 (d, *J* = 5.0 Hz, 4H, Py_t), 8.39-8.41 (m, 4H, Py_B), 7.65 (d, *J* = 5.0 Hz, 4H, Py_t), 7.34 (d, *J* = 7.5 Hz, 2H, An), 7.14 (t, *J* = 7.5 Hz, 2H, An), 6.53 (d, *J* = 7.0 Hz,

2H, An), 1.31-1.25 (m, 2H, B-CH_2), 1.06 (m, 2H, B-CH_2), 0.76-0.62 (m, 4H, B-CH_2), 0.47 (t, *J* = 7.5 Hz, 6H, CH_3), 0.29 (t, *J* = 7.5 Hz, 6H, CH_3). **$^{13}C\{^1H\}$ NMR** (125.8 MHz, $CDCl_3$, 25 °C): δ (ppm) = 151.4, 151.1, 146.9 (br, B-C), 143.9, 142.7, 138.1, 137.3, 136.7, 135.3, 130.1, 130.0, 126.5, 121.7, 118.7, 82.1, 22.6, 19.5, 10.4, 9.6. **^{11}B NMR** (160.3 MHz, $CDCl_3$, 25 °C): δ (ppm) = 2.2.

Synthesis of **1Py-BCF-O_2**

Compound **1Py-BCF** (5.0 mg, 7.6 μmol) was dissolved in 0.6 mL of anhydrous oxygen saturated $CDCl_3$, and the solution was irradiated for 30 min with a Xe lamp (34 Watt) resulting in a stark colour change from dark blue to light pink. The solution was used for characterization directly without further purification. **^1H NMR** (500.2 MHz, $CDCl_3$, 25 °C): δ (ppm) = 9.27 (s, 2H, Py_B), 8.86 (br, 4H, Py_t), 8.50 (br, 4H, Py_B), 8.03 (br, 4H, Py_t), 7.34 (d, *J* = 7.5 Hz, 2H, An), 7.15 (t, *J* = 7.3 Hz, 2H, An), 6.48 (d, *J* = 7.0 Hz, 2H, An), 1.12-1.08 (m, 2H, B-CH_2), 0.87 (m, 2H, B-CH_2), 0.74 (m, 2H, B-CH_2), 0.66 (m, 2H, B-CH_2), 0.44 (t, *J* = 7.3 Hz, 6H, CH_3), 0.27 (t, *J* = 7.3 Hz, 6H, CH_3). **$^{13}C\{^1H\}$ NMR** (150.8 MHz, $CDCl_3$, 25 °C): δ (ppm) = 153.4, 148.8, 148.1, 147.8 (d, $J(^{19}F,^{13}C)$ = 244 Hz), 147.4 (br, B-C) 146.5, 144.2, 140.6 (d, $J(^{19}F,^{13}C)$ = 250 Hz), 137.8, 137.6, 137.4 (d, $J(^{19}F,^{13}C)$ = 248 Hz), 134.7, 133.4, 130.5, 130.4, 126.8, 123.6, 118.6, 117.4 (br, B-C), 82.1, 22.7, 19.6, 10.4, 9.9. **^{11}B NMR** (160.3 MHz, $CDCl_3$, 25 °C): δ (ppm) = 2.8, -2.5 (overlapped). **^{19}F NMR** (470.7 MHz, $CDCl_3$, 25 °C): δ (ppm) = -131.1 (br d, $J(^{19}F,^{19}F)$ = 14.1 Hz), -155.2 (t, $J(^{19}F,^{19}F)$ = 20.2 Hz), -162.1 (br t, $J(^{19}F,^{19}F)$ = 18.4 Hz).

Synthesis of **1Py-Me^{2+}-O_2**

Compound **1Py-Me^{2+}** (5.0 mg, 5.3 μmol) was dissolved in 3 mL of anhydrous oxygen-saturated acetonitrile in a vial. The solution was irradiated by sunlight at room temperature, resulting in complete decoloration within ca. 1 hour. Removal of the solvent under vacuum at room temperature gave **1Py-Me^{2+}-O_2** as pale yellow solid. **^1H NMR** (500.2 MHz, CD_3CN, 25 °C): δ (ppm) = 9.30 (d, *J* = 1.5 Hz, 2H, Py_B), 8.82 (d, *J* = 6.5 Hz, 4H, Py_t), 8.74 (dd, *J* = 8.3, 2.2 Hz, 2H, Py_B), 8.37 (d, *J* = 8.5 Hz, 2H, Py_B), 7.35 (d, *J* = 7.5 Hz, 4H, Py_t), 7.20 (t, *J* = 7.5 Hz, 2H, An), 6.64 (d, *J* = 7.5 Hz, 2H, An), 4.40 (s, 6H, N-Me), 1.02-0.98 (m, 4H, B-CH_2), 0.73 (m, 2H, B-CH_2), 0.68 (m, 2H, B-CH_2), 0.39 (t, *J* = 7.5 Hz, 6H, CH_3), 0.24 (t, *J* = 7.5 Hz, 6H, CH_3). **$^{13}C\{^1H\}$ NMR** (125.8 MHz, CD_3CN, 25 °C): δ (ppm) = 152.7, 151.6, 146.8, 145.7, 140.8, 139.0, 136.1, 134.9, 130.8, 130.7, 127.5, 127.4, 119.7, 49.1, 23.6, 19.5,10.4, 9.9, one signal for carbons attached to boron and one signal for carbons attached to oxygen not observed. **^{11}B NMR** (160.5 MHz, CD_3CN, 25 °C): δ (ppm) = 3.4. **^{19}F NMR** (470.6 MHz, CD_3CN, 25 °C): δ (ppm) = -73.0 (d, $^1J(^{19}F, ^{31}P)$ = 706.4 Hz), **^{31}P NMR** (202.5 MHz, CD_3CN, 25 °C): δ (ppm) = -144.6 (h, $^1J(^{31}P, ^{19}F)$ = 706.6 Hz).

NMR and HRMS Characterization Data

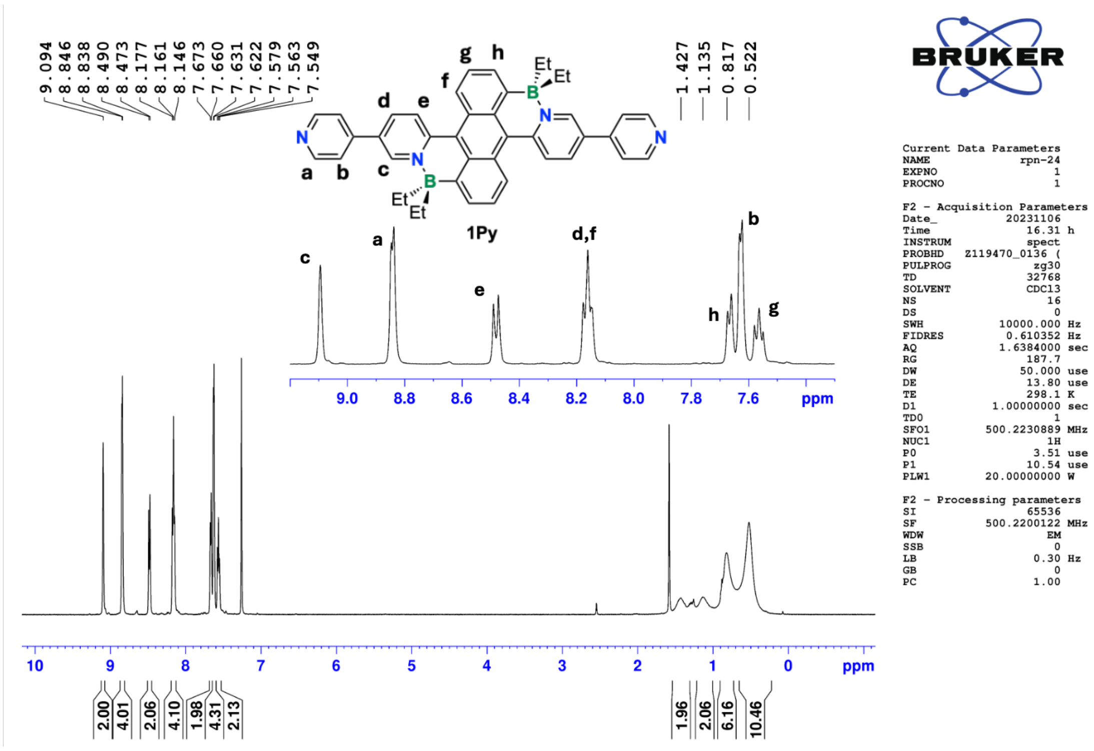

Figure S1. ^{1}H NMR spectrum of **1Py** in $CDCl_3$ at 25 °C.

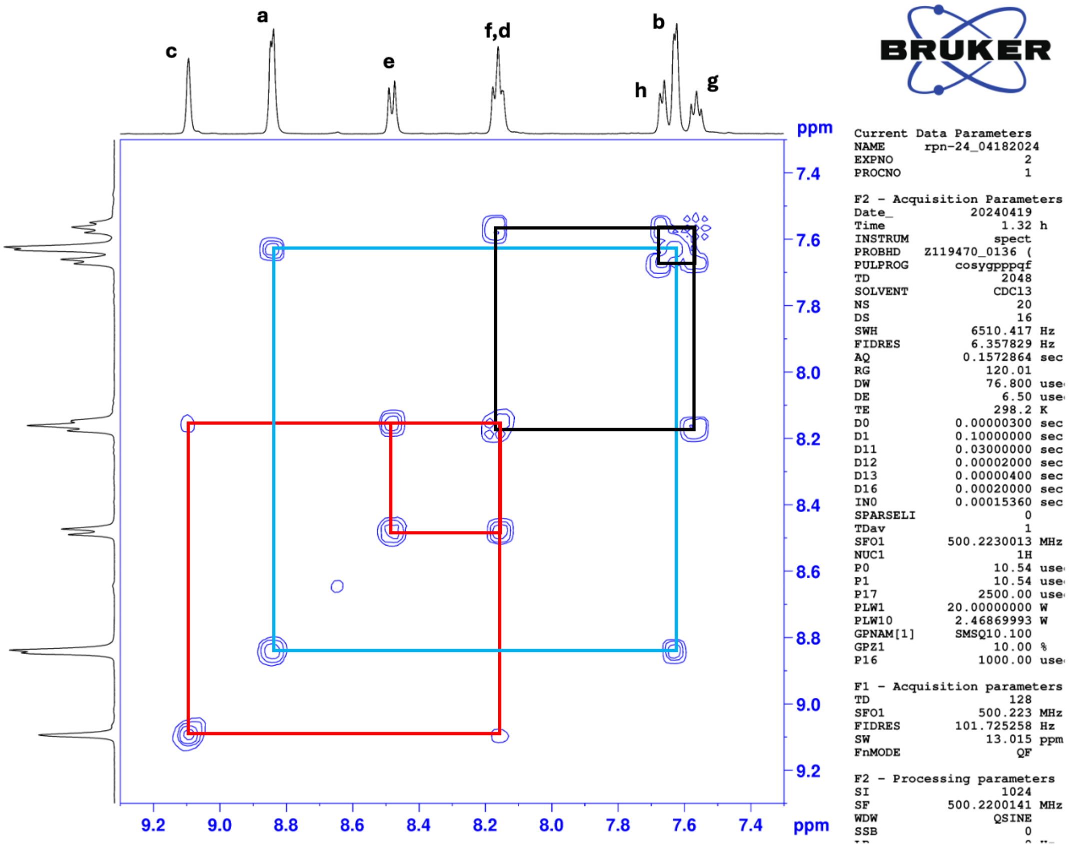

Figure S2. Aromatic region of the gCOSY NMR spectrum of **1Py** in $CDCl_3$ at 25 °C.

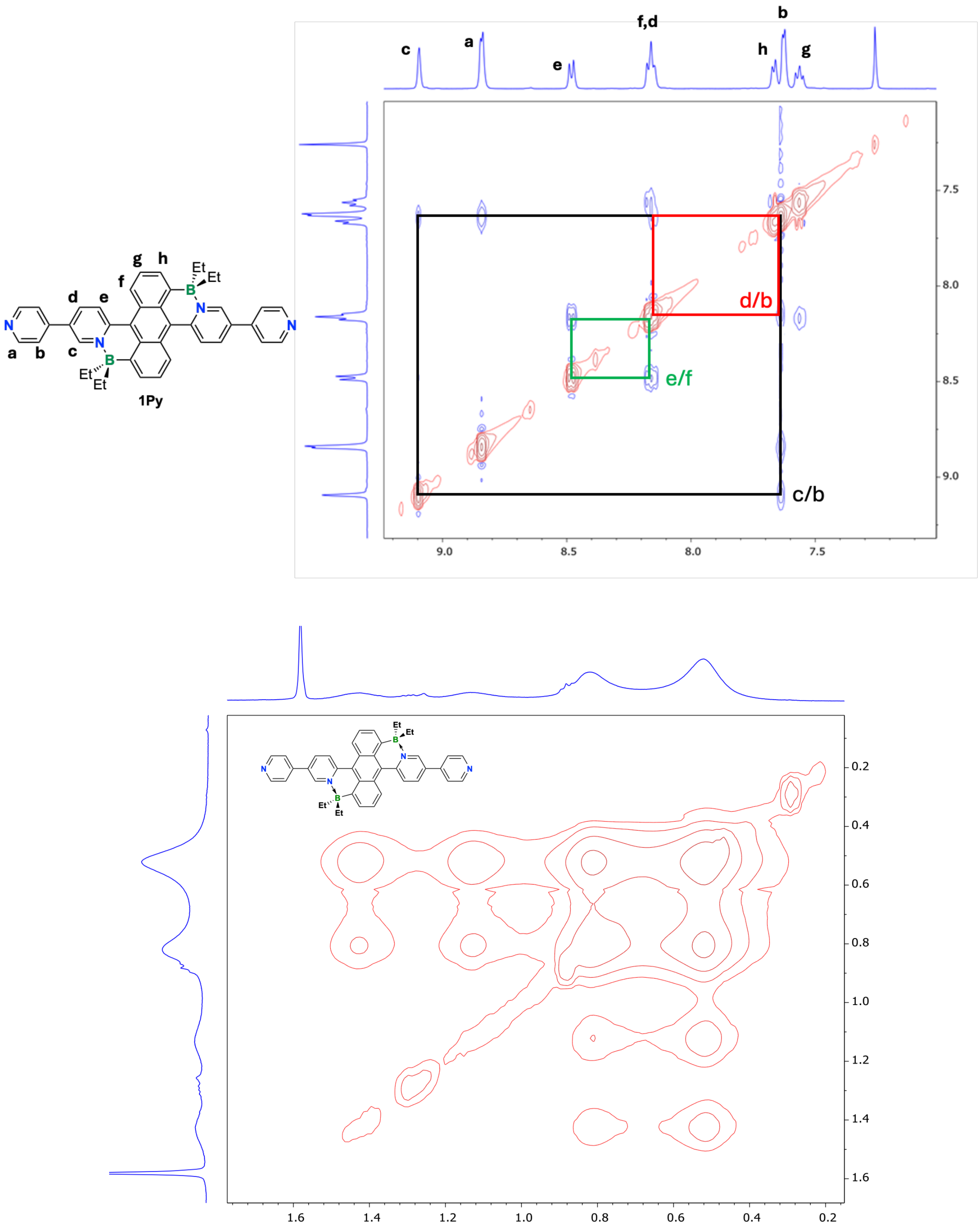

Figure S3. Expansions of the HH-NOESY NMR spectrum **1Py** in $CDCl_3$ at 25 °C; (top) NOE peaks between Py_B-H(e) and anthracene-H(f) protons indicated with green lines; NOE peaks between Py_B-H(c) and ligand Py_t-H(b) protons indicated with black lines, and NOE peaks between Py_B-H(d) and ligand Py_t-H(b) protons indicated with red lines; bottom: the aliphatic region shows exchange peaks due to interconversion of B-Et substituents.

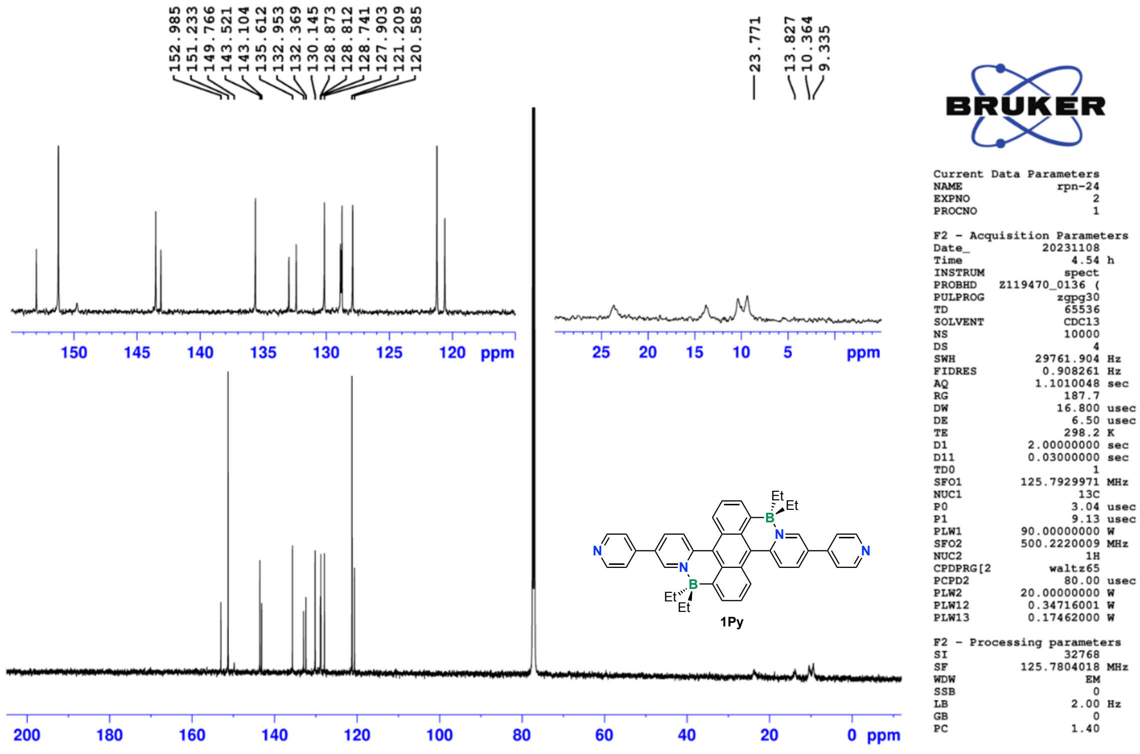

Figure S4. $^{13}C\{^1H\}$ NMR spectrum of **1Py** in $CDCl_3$ at 25 °C.

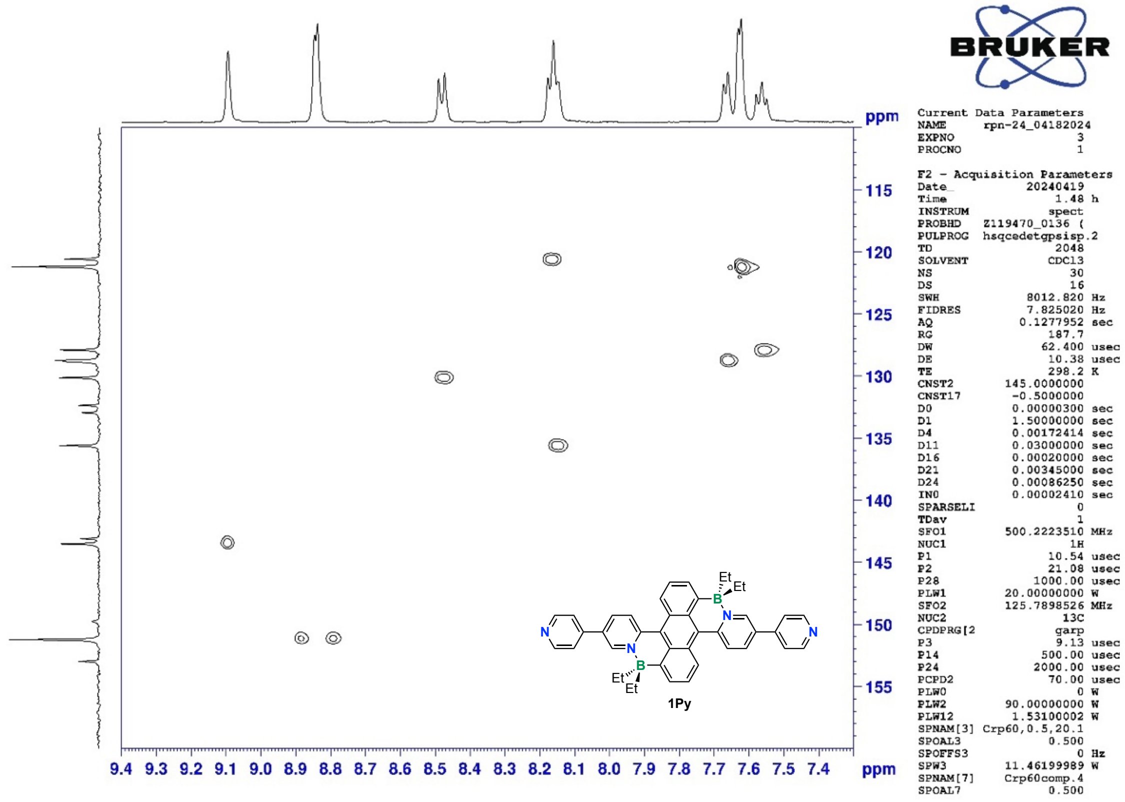

Figure S5. Aromatic region of the HSQC NMR spectrum of **1Py** in $CDCl_3$ at 25 °C

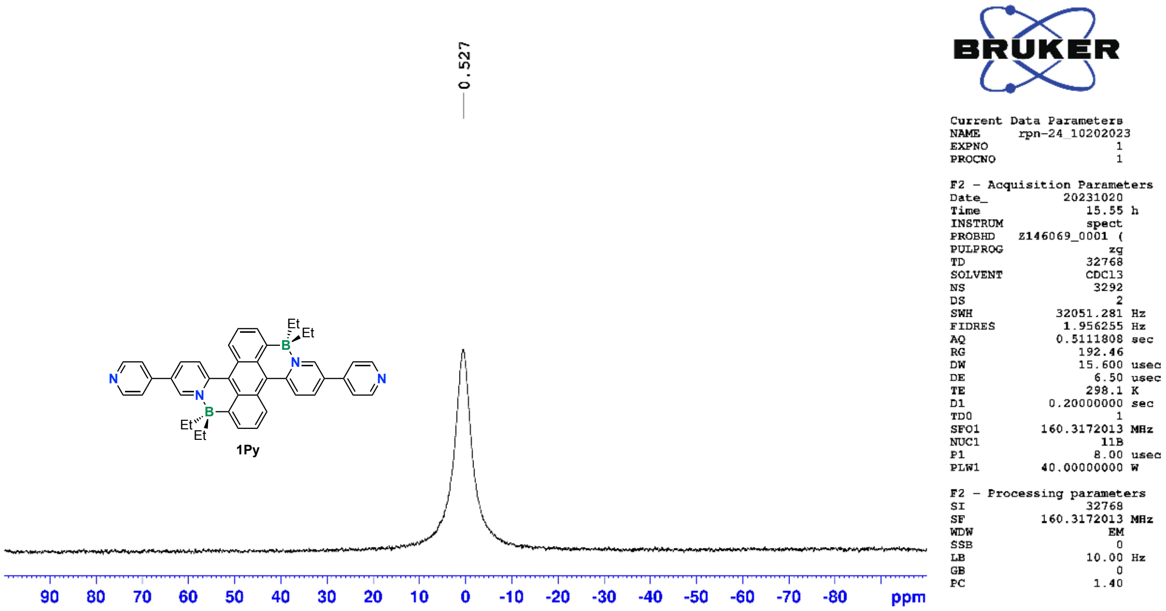

Figure S6. ^{11}B NMR spectrum of **1Py** in $CDCl_3$ at 25 °C.

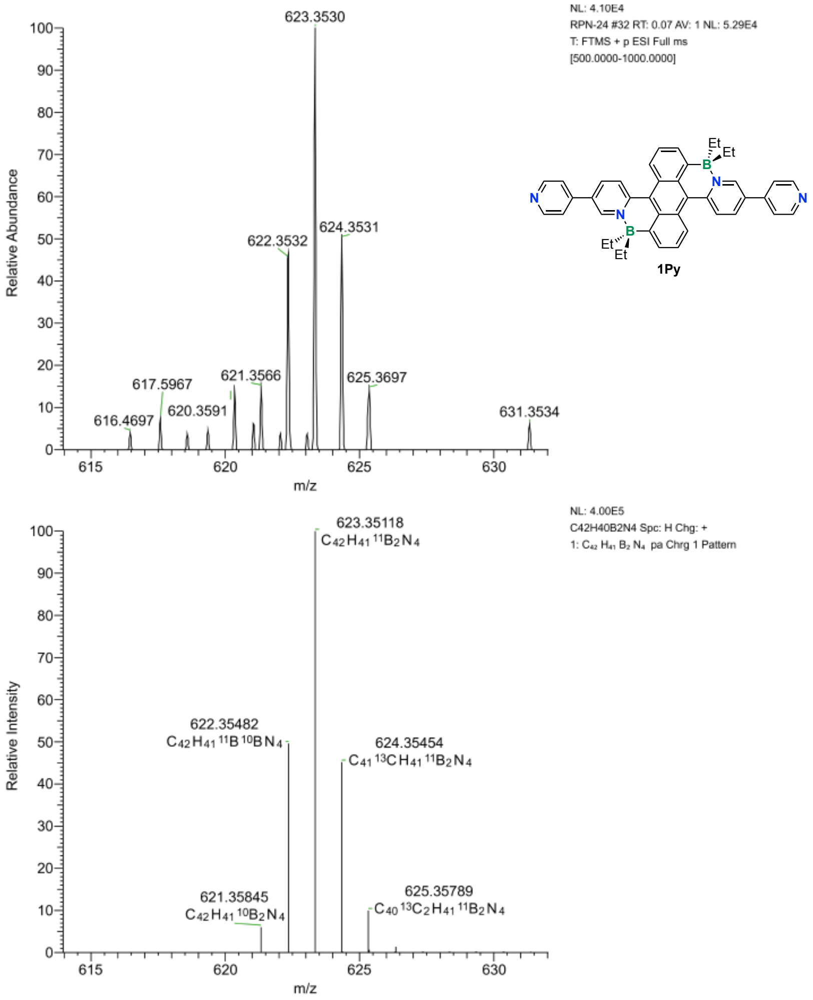

Figure S7. HRMS (ESI, positive mode) data for **1Py** in acetonitrile.

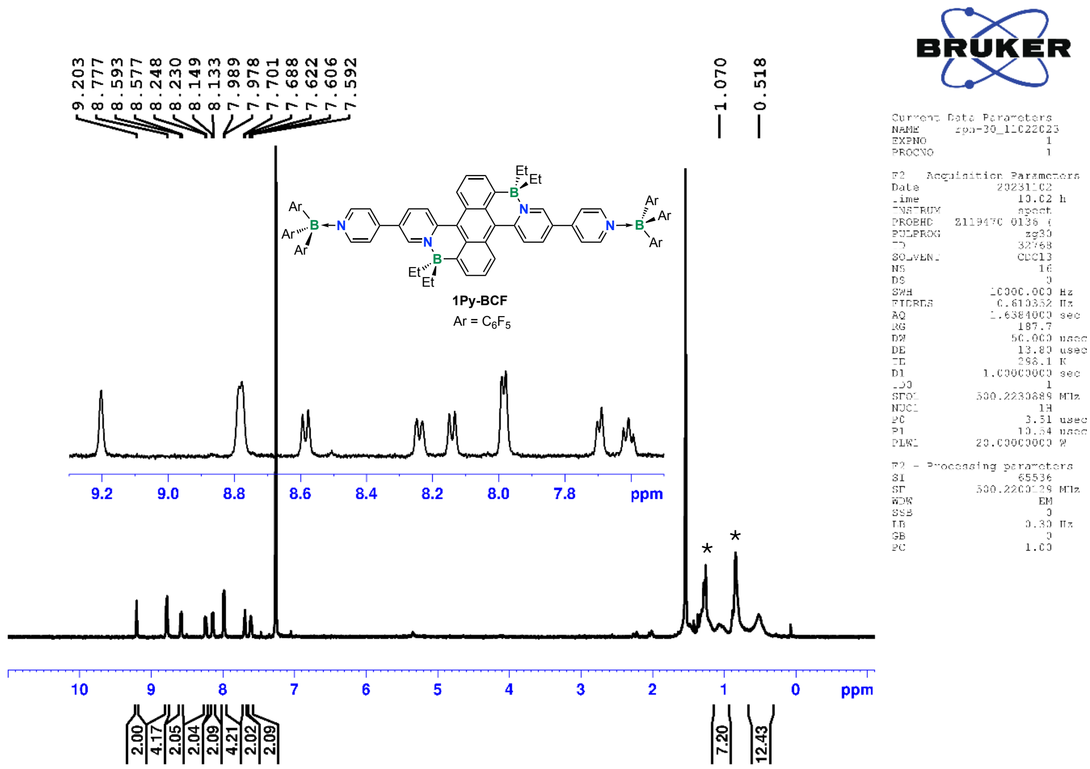

Figure S8. ^{1}H NMR spectrum of **1Py-BCF** in $CDCl_3$ at 25 °C (* residual hexanes).

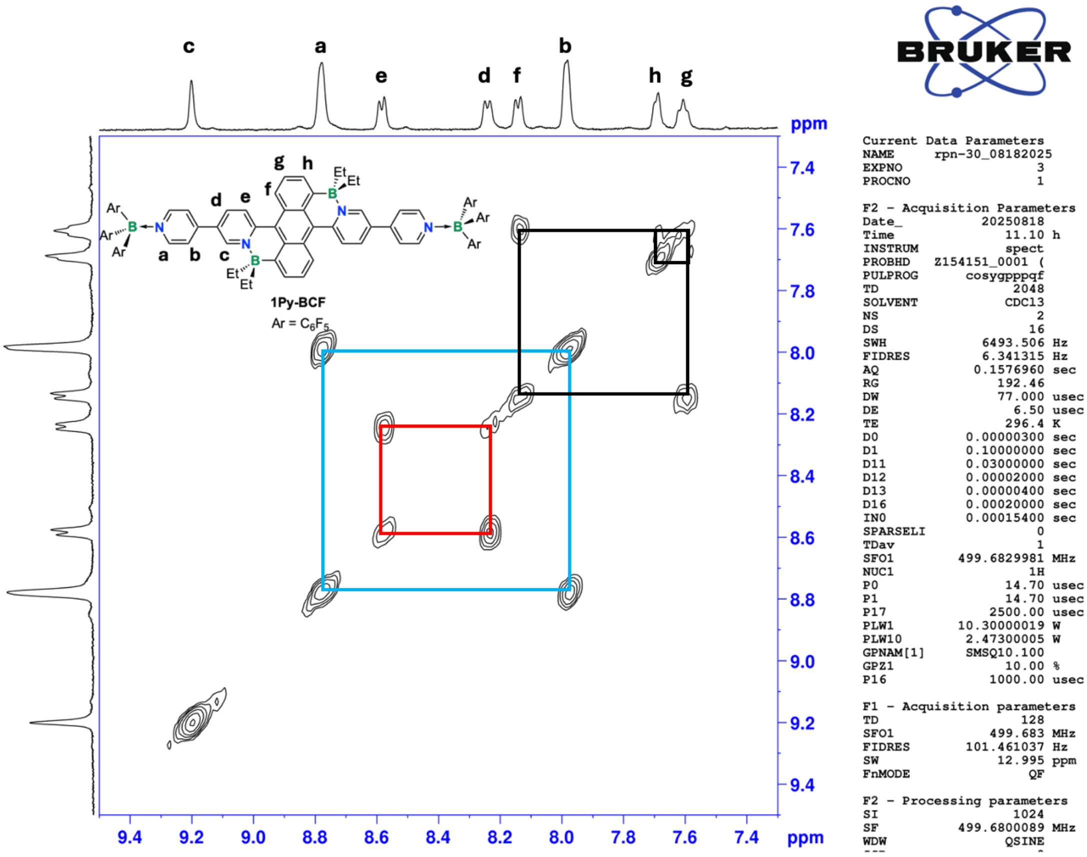

Figure S9. Aromatic region of the gCOSY NMR spectrum of **1Py-BCF** in $CDCl_3$ at 25 °C.

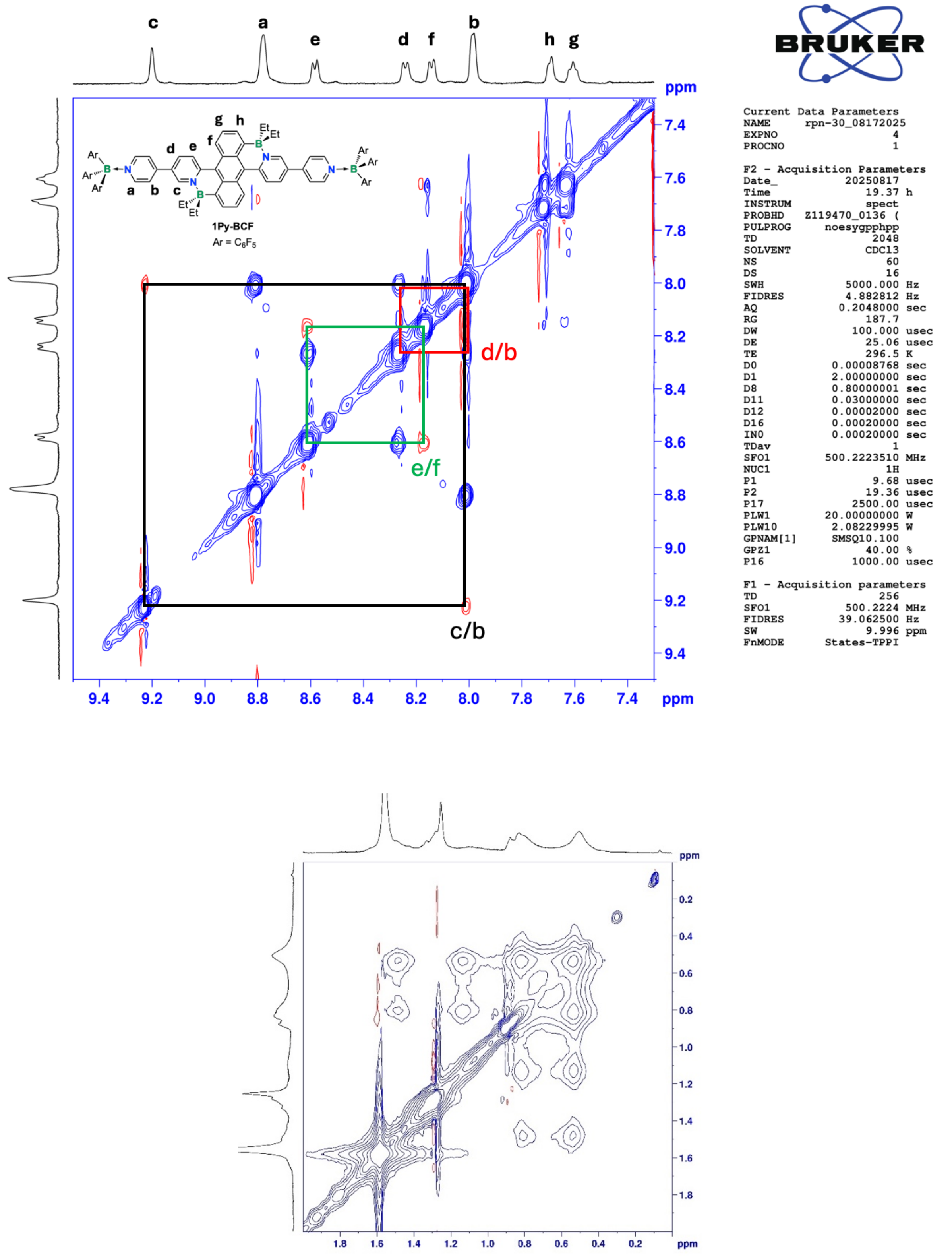

Figure S10. Expansions of the HH-NOESY NMR spectrum **1Py-BCF** in $CDCl_3$ at 25 °C; (top) NOE peaks between Py_B-H(e) and anthracene-H(f) protons indicated with green lines; NOE peaks between Py_B-H(c) and ligand Py_t-H(b) protons indicated with black lines, and NOE peaks between Py_B-H(d) and ligand Py_t-H(b) protons indicated with red lines; (bottom) the aliphatic region shows exchange peaks due to interconversion of B-Et substituents.

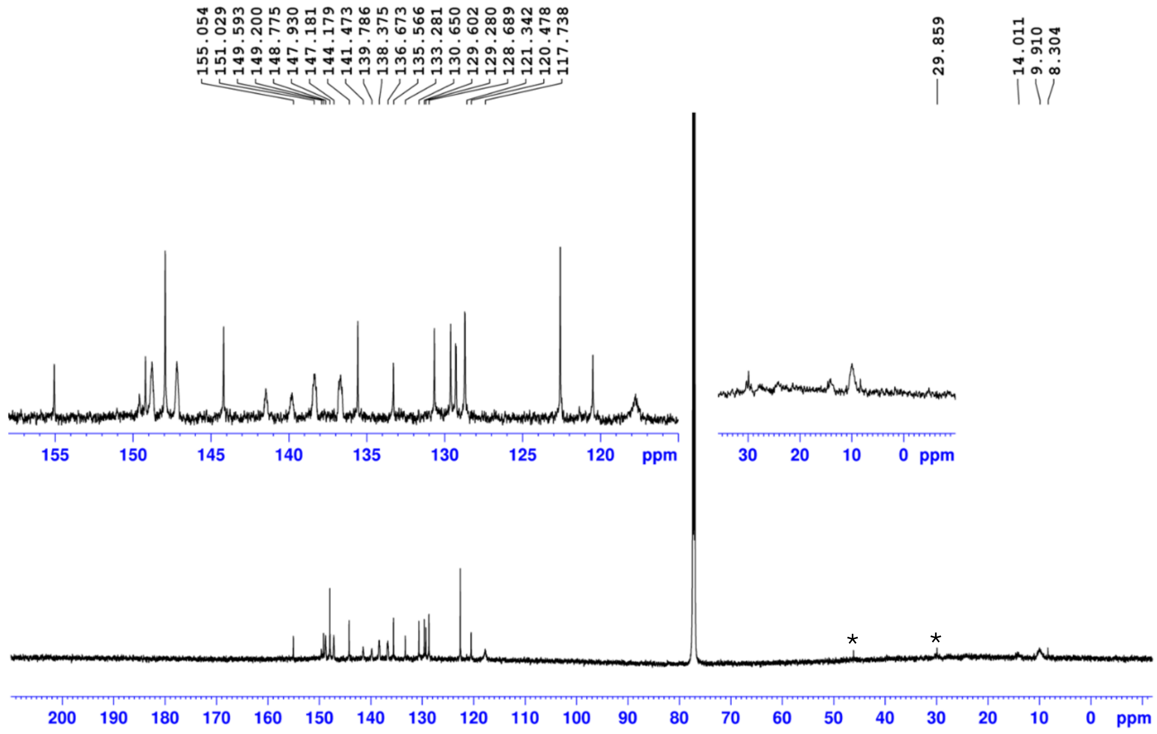

Figure S11. $^{13}C\{^{1}H\}$ NMR spectrum of **1Py-BCF** in $CDCl_3$ at 25 °C (* residual hexanes).

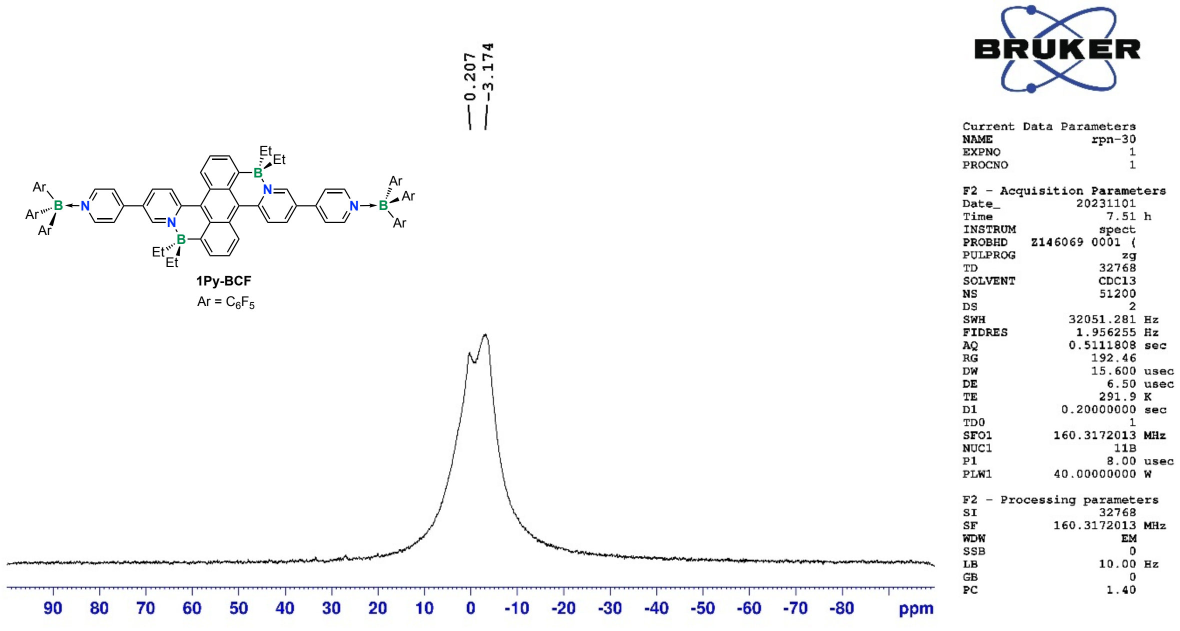

Figure S12. ^{11}B NMR spectrum of **1Py-BCF** in $CDCl_3$ at 25 °C.

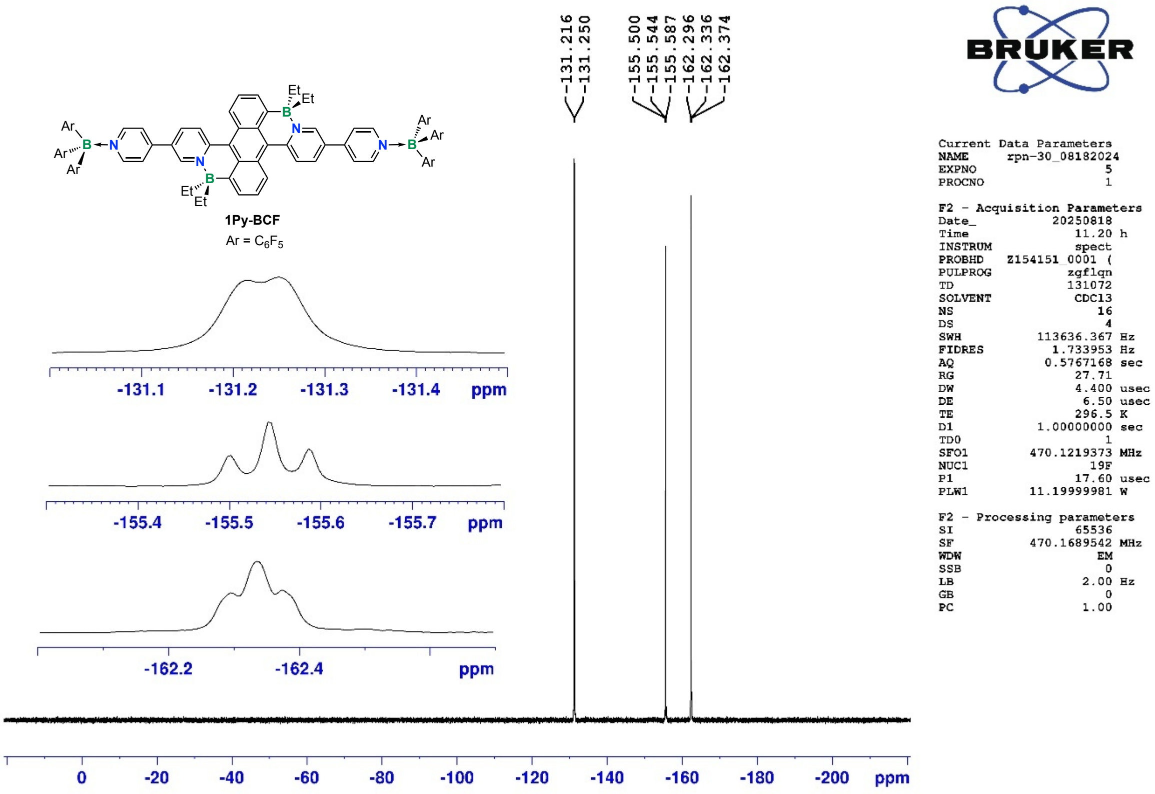

Figure S13. ^{19}F NMR spectrum of **1Py-BCF** in $CDCl_3$ at 25 °C.

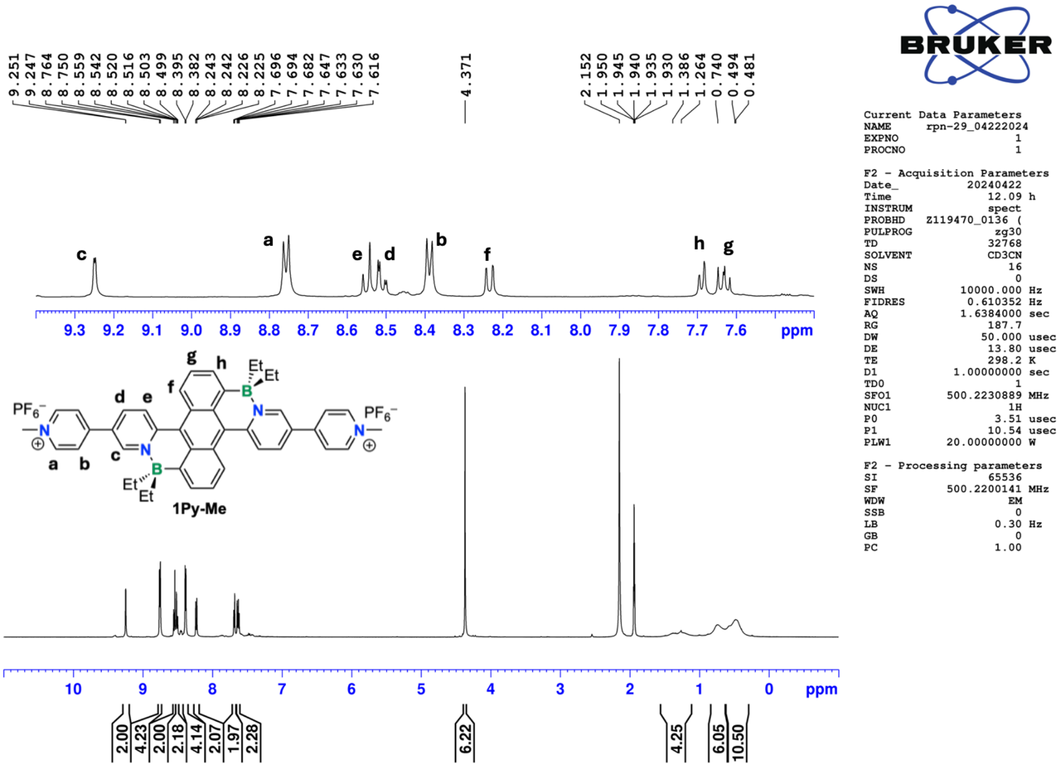

Figure S14. ^{1}H NMR spectrum of **1Py-Me^{2+}** in CD_3CN at 25 °C.

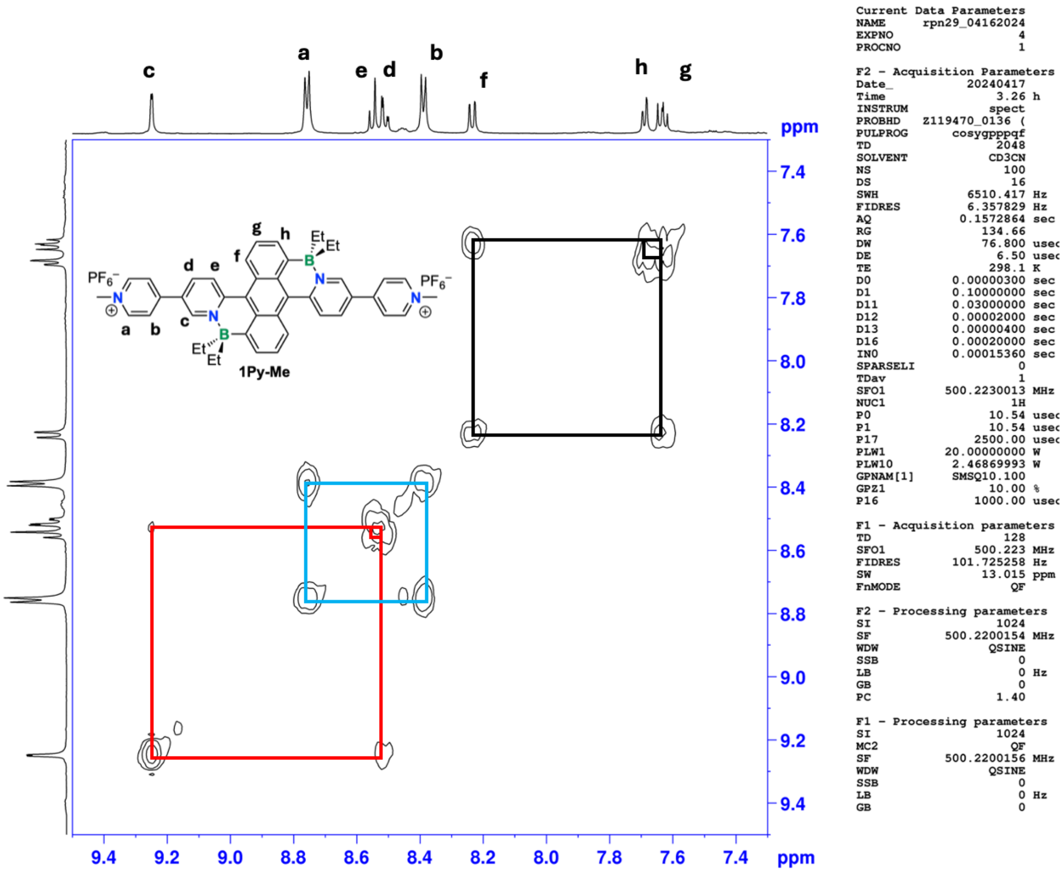

Figure S15. Aromatic region of the gCOSY NMR spectrum of **1Py-Me^{2+}** in CD_3CN at 25 °C.

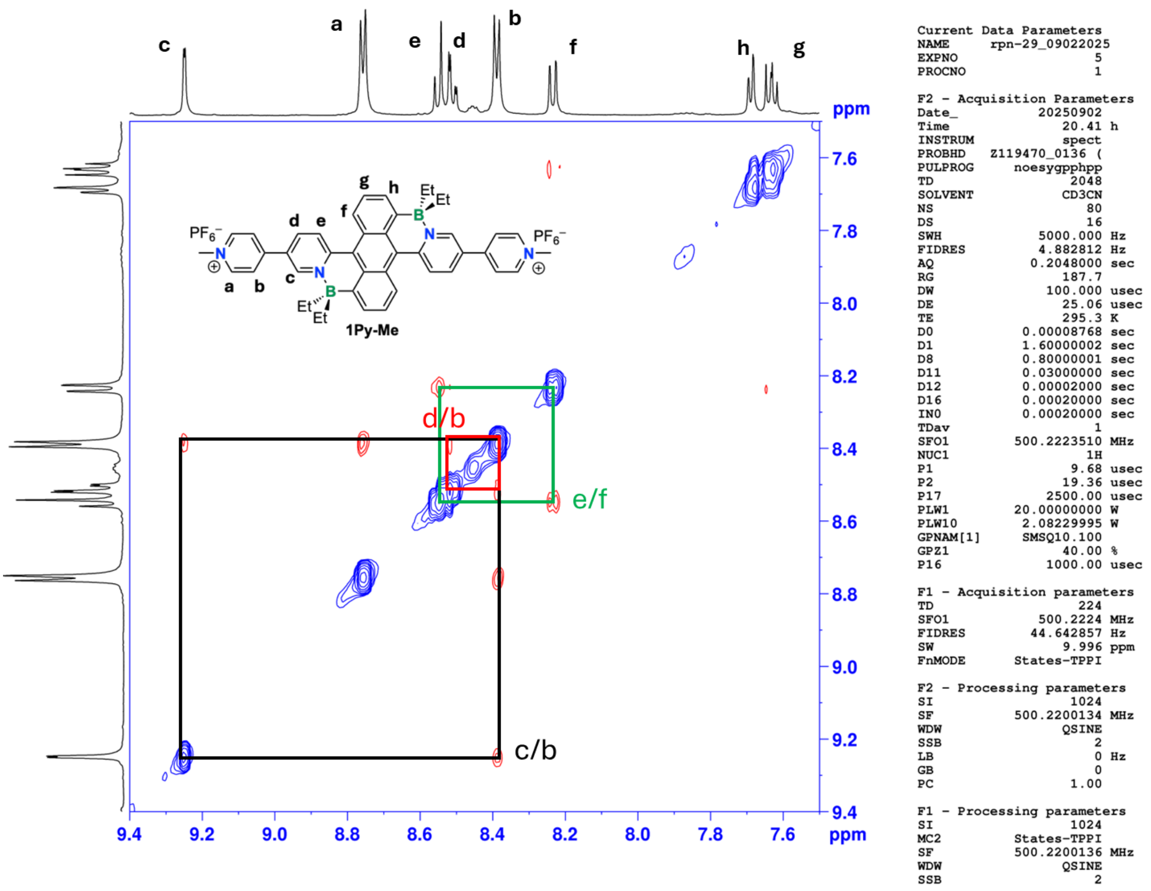

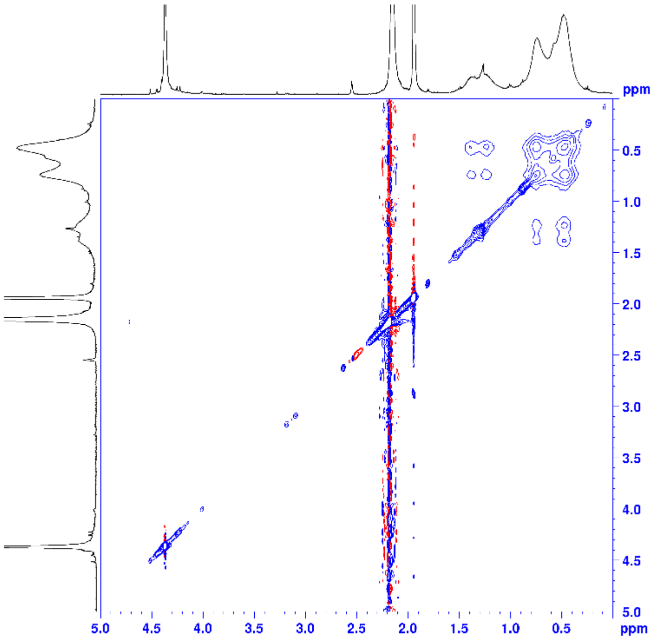

Figure S16. Expansions of the HH-NOESY NMR spectrum **1Py-Me^{2+}** in CD_3CN at 25 °C; (top) NOE peaks between Py_B-H(e) and anthracene-H(f) protons indicated with green lines; NOE peaks between Py_B-H(c) and ligand Py_t-H(b) protons indicated with black lines, and Py_B-H(d) and ligand Py_t-H(b) protons indicated with red lines; (bottom) the aliphatic region shows exchange peaks due to interconversion of B-Et substituents.

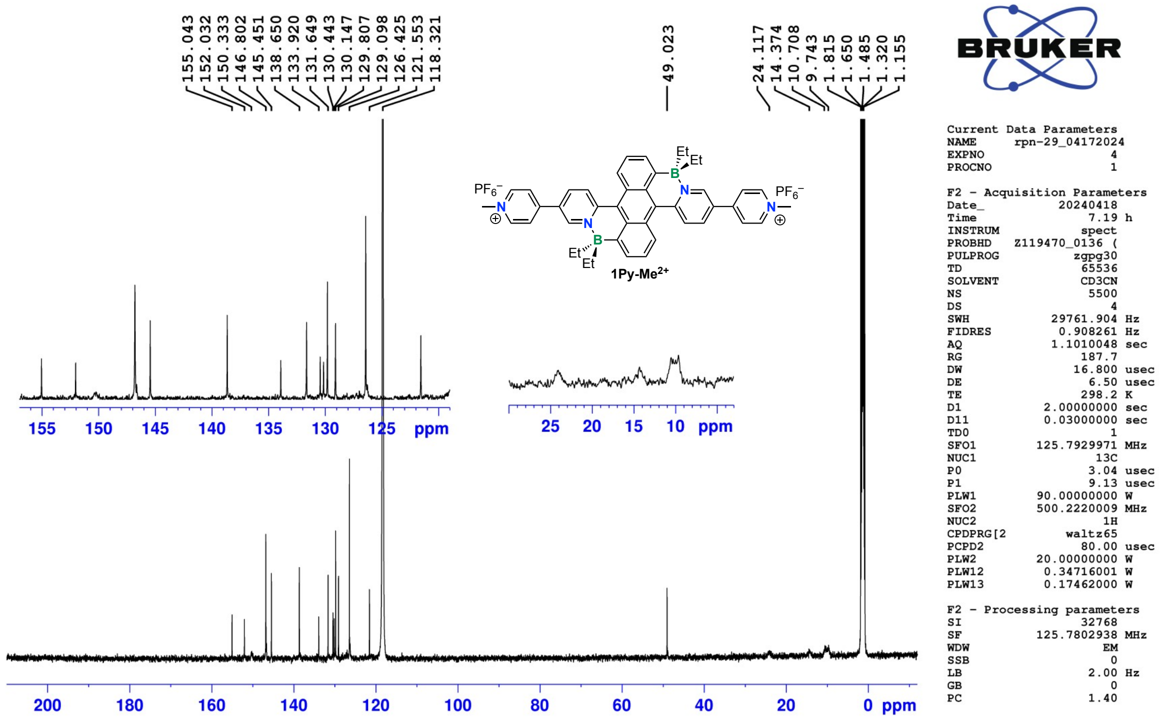

Figure S17. $^{13}C\{^{1}H\}$ NMR spectrum of **1Py-Me^{2+}** in CD_3CN at 25 °C.

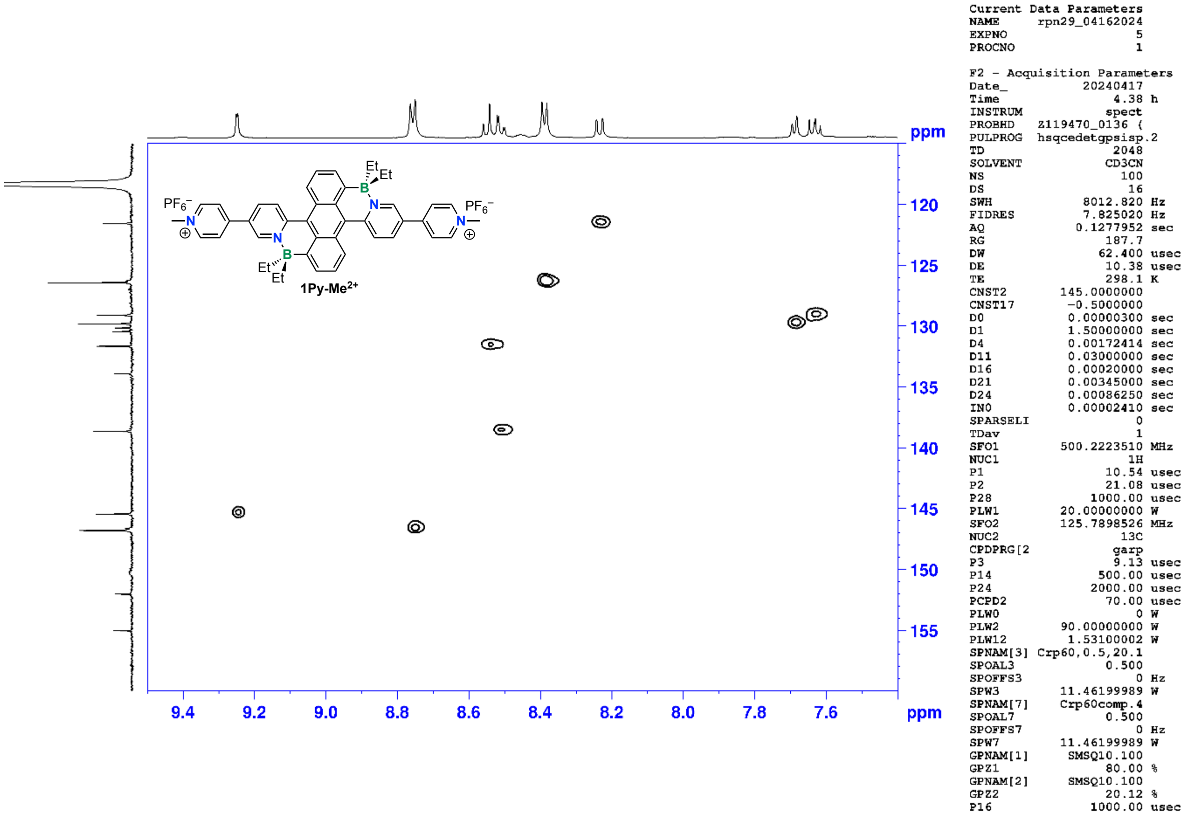

Figure S18. Aromatic region of the HSQC NMR spectrum of **1Py-Me^{2+}** in $CDCl_3$ at 25 °C

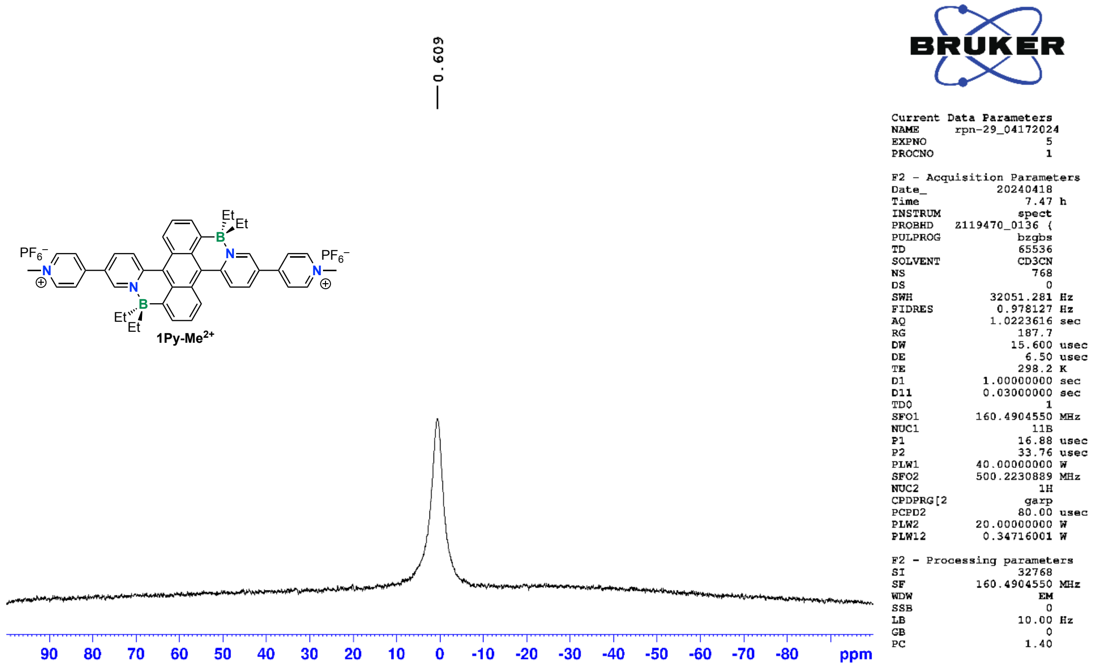

Figure S19. ^{11}B NMR spectrum of **1Py-Me^{2+}** in CD_3CN at 25 °C.

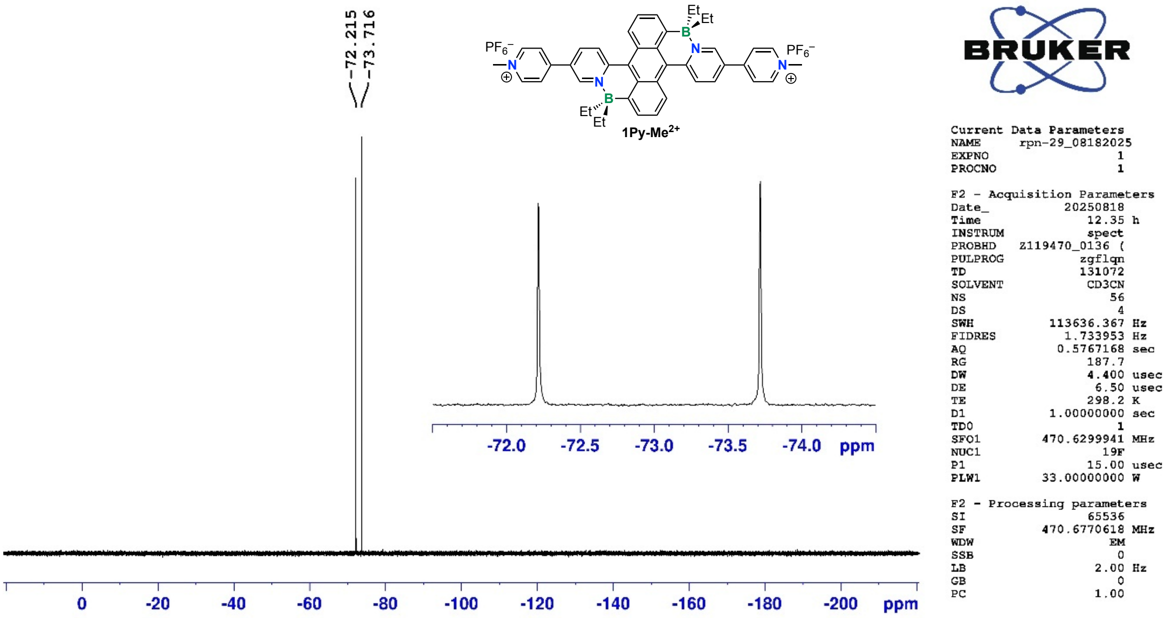

Figure S20. ^{19}F NMR spectrum of **1Py-Me^{2+}** in CD_3CN at 25 °C.

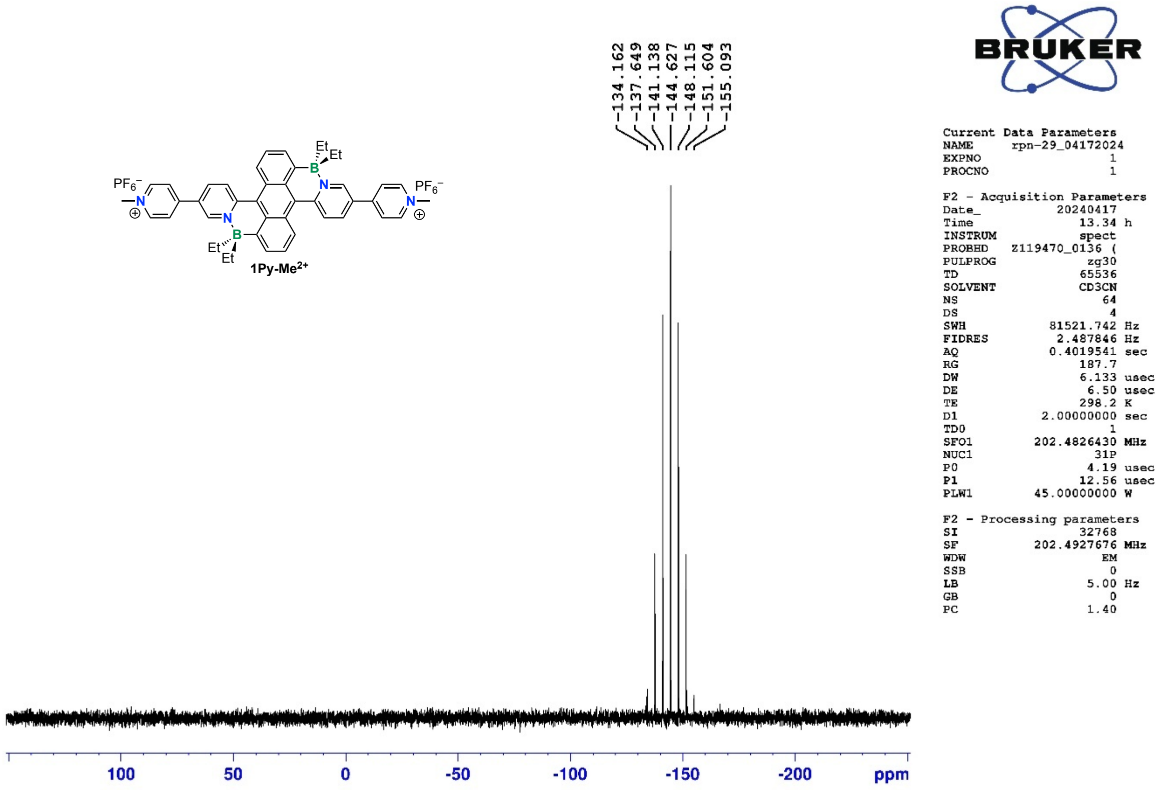

Figure S21. ^{31}P NMR spectrum of **1Py-Me^{2+}** in CD_3CN at 25 °C.

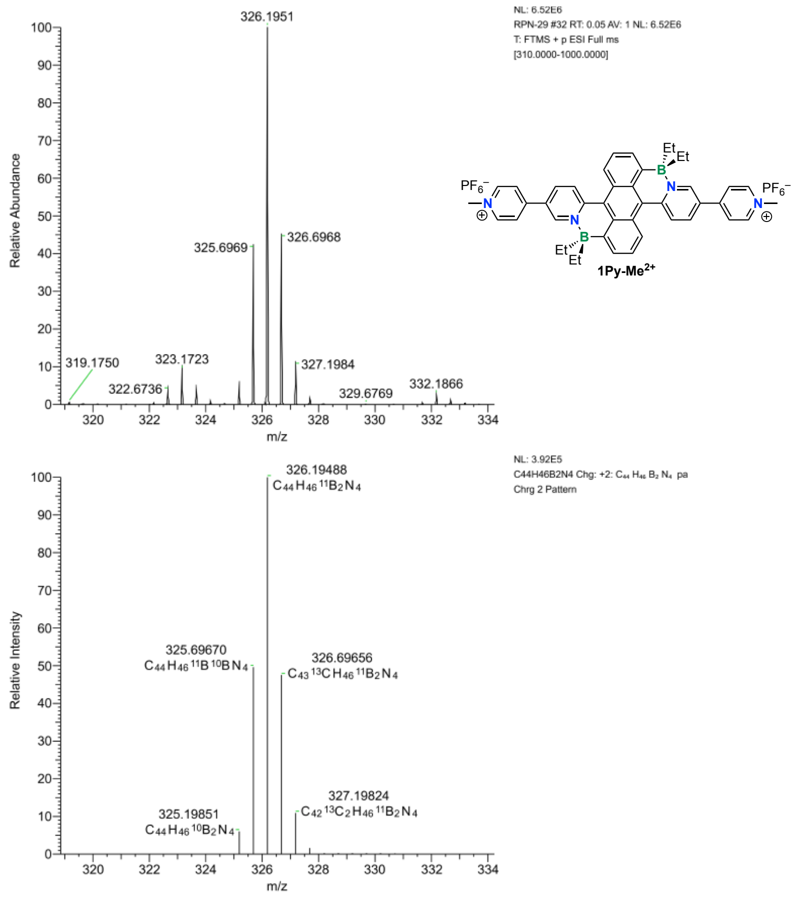

Figure S22. HRMS (ESI, positive mode) data for dicationic **1Py-Me^{2+}** in acetonitrile.

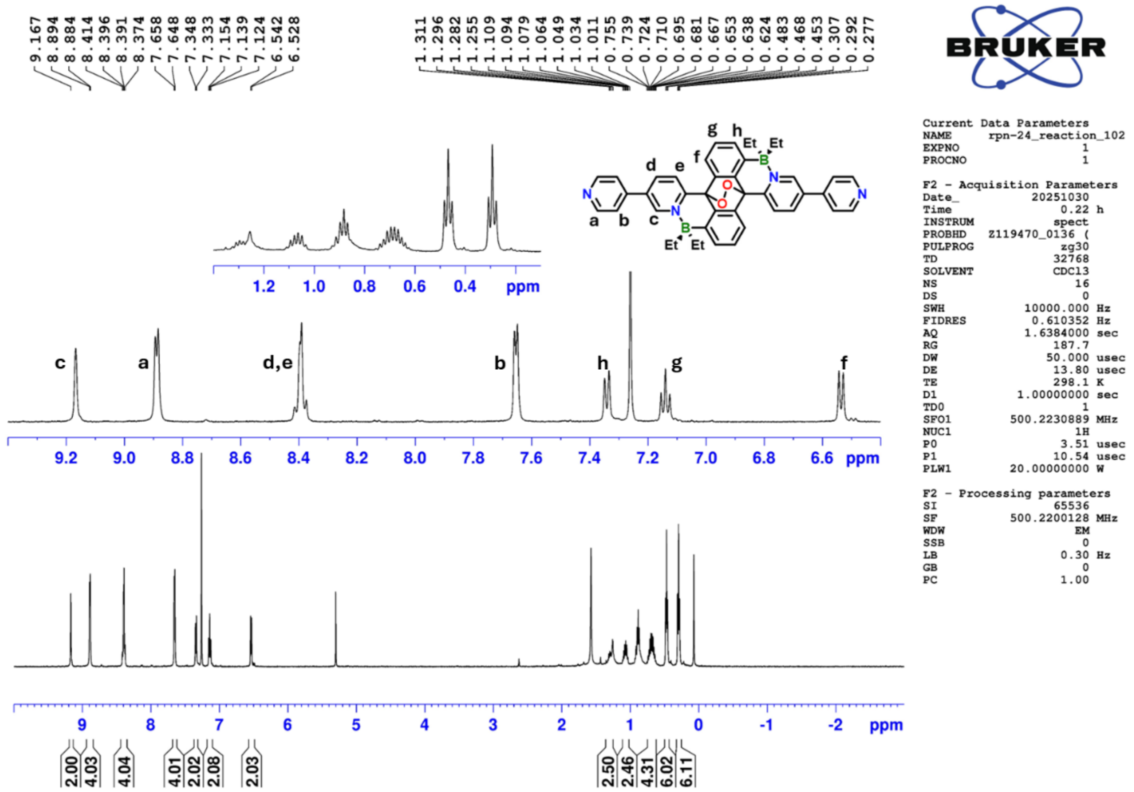

Figure S23. ^{1}H NMR spectrum of **1Py-O$_2$** in $CDCl_3$ at 25 °C.

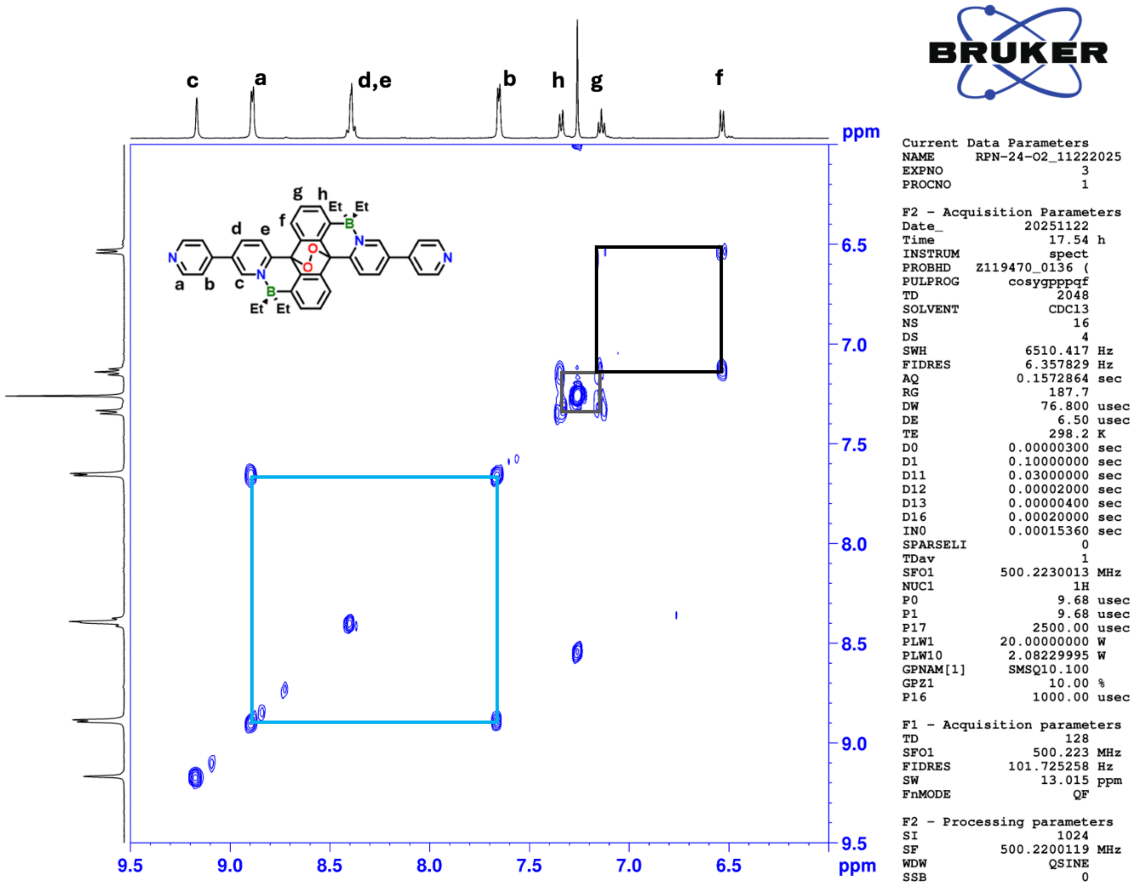

Figure S24. Aromatic region of the gCOSY NMR spectrum of **1Py-O$_2$** in $CDCl_3$ at 25 °C.

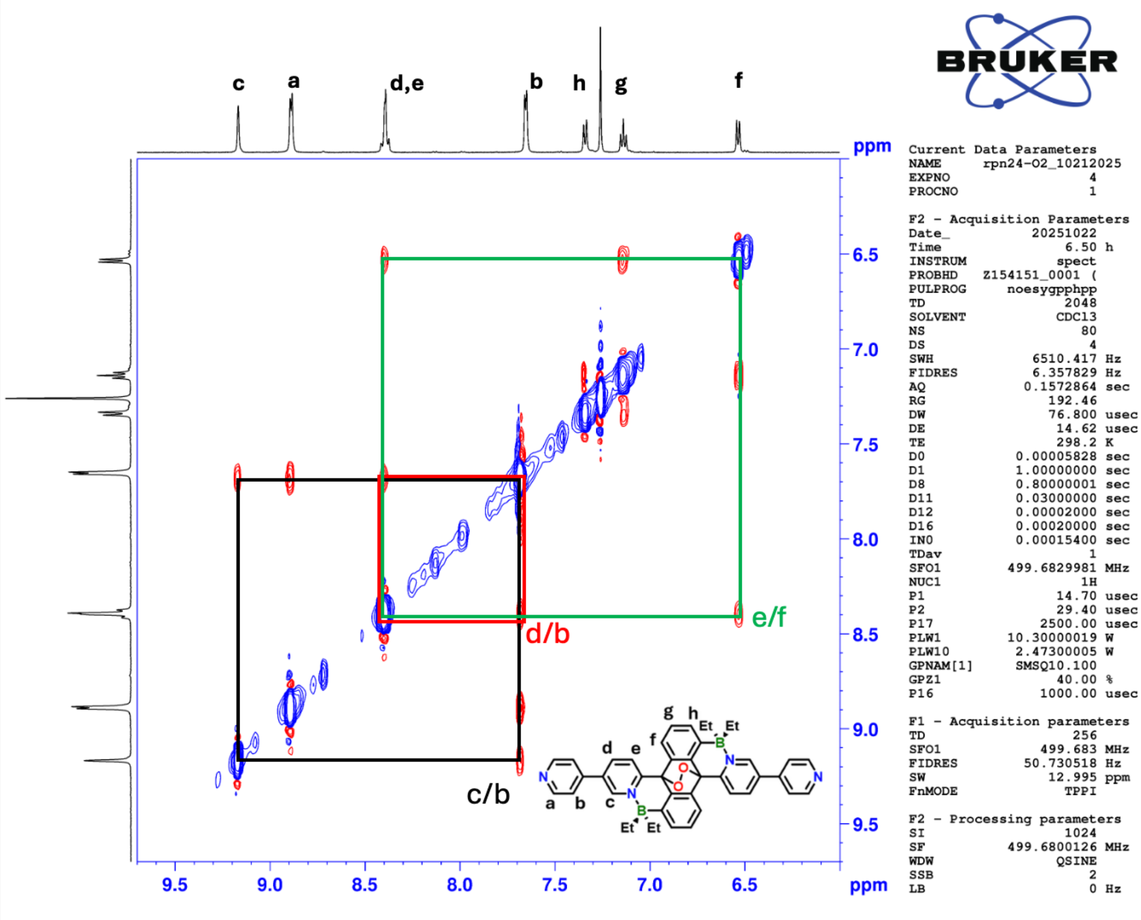

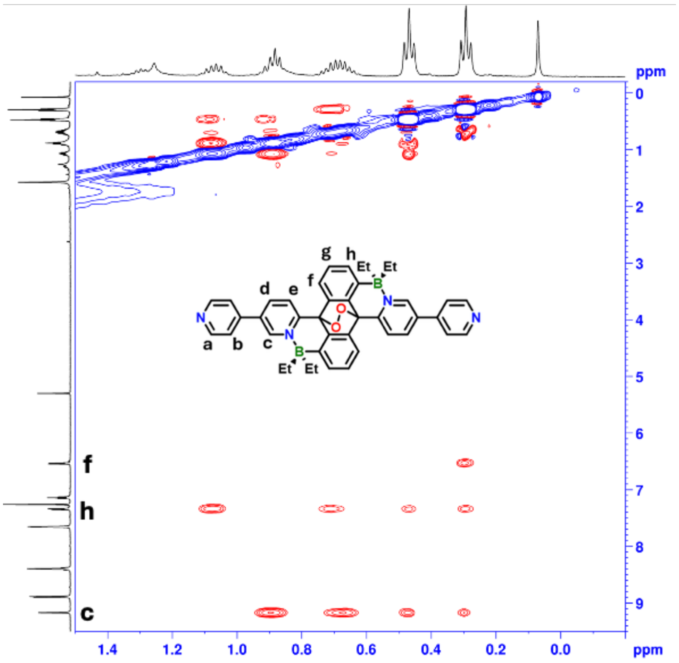

Figure S25. Expansions of the HH-NOESY NMR spectrum **1Py-O$_2$** in $CDCl_3$ at 25 °C; (top) NOE peaks between Py$_B$-H(e) and anthracene-H(f) protons indicated with green lines; NOE peaks between Py$_B$-H(c) and ligand Py$_t$-H(b) protons indicated with black lines, and NOE peaks between Py$_B$-H(d) and ligand Py$_t$-H(b) protons indicated with red lines; (bottom) the aliphatic region shows exchange peaks due to interconversion of B-Et substituents. NOE peaks between aliphatic protons and Py$_B$-H(c) / anthracene-H(h) are also observed.

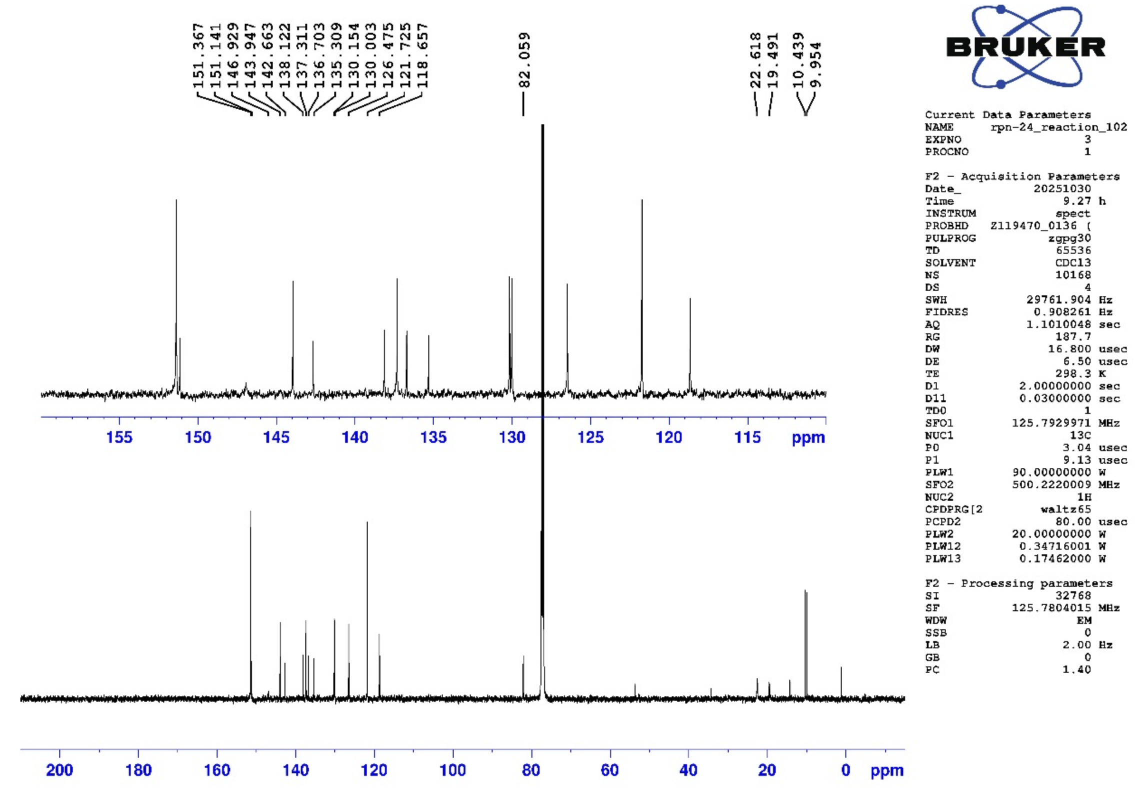

Figure S26. $^{13}C\{^1H\}$ NMR spectrum of **1Py-O$_2$** in $CDCl_3$ at 25 °C.

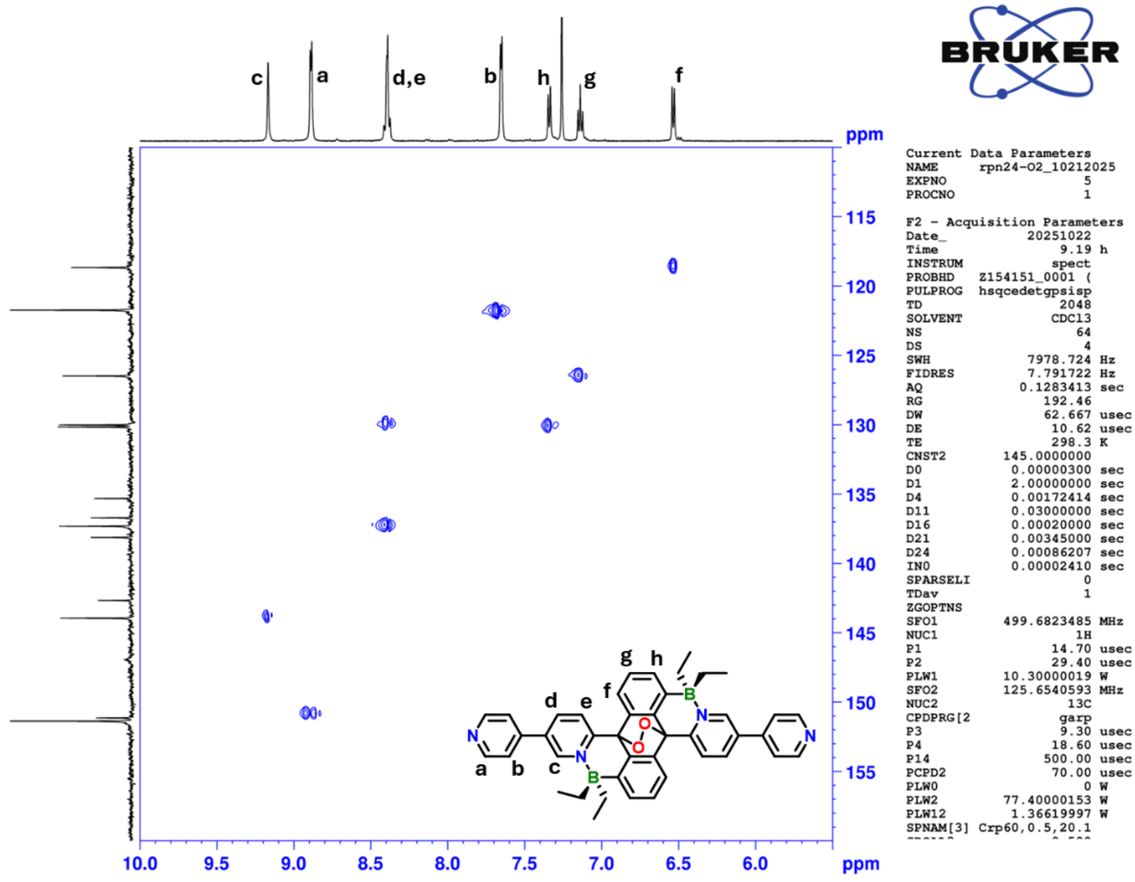

Figure S27. Aromatic region of the HSQC NMR spectrum of **1Py-O$_2$** in $CDCl_3$ at 25 °C.

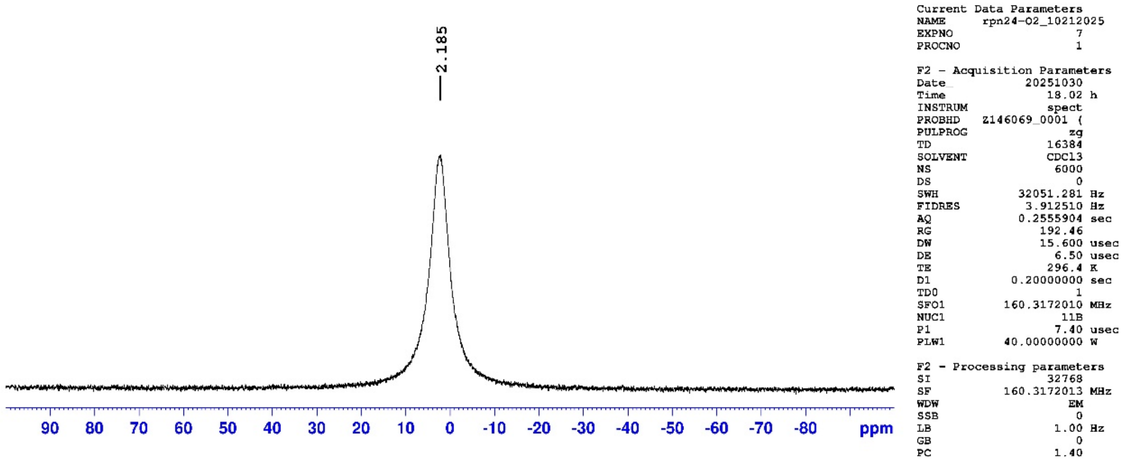

Figure S28. ^{11}B NMR spectrum of **1Py-O$_2$** in $CDCl_3$ at 25 °C.

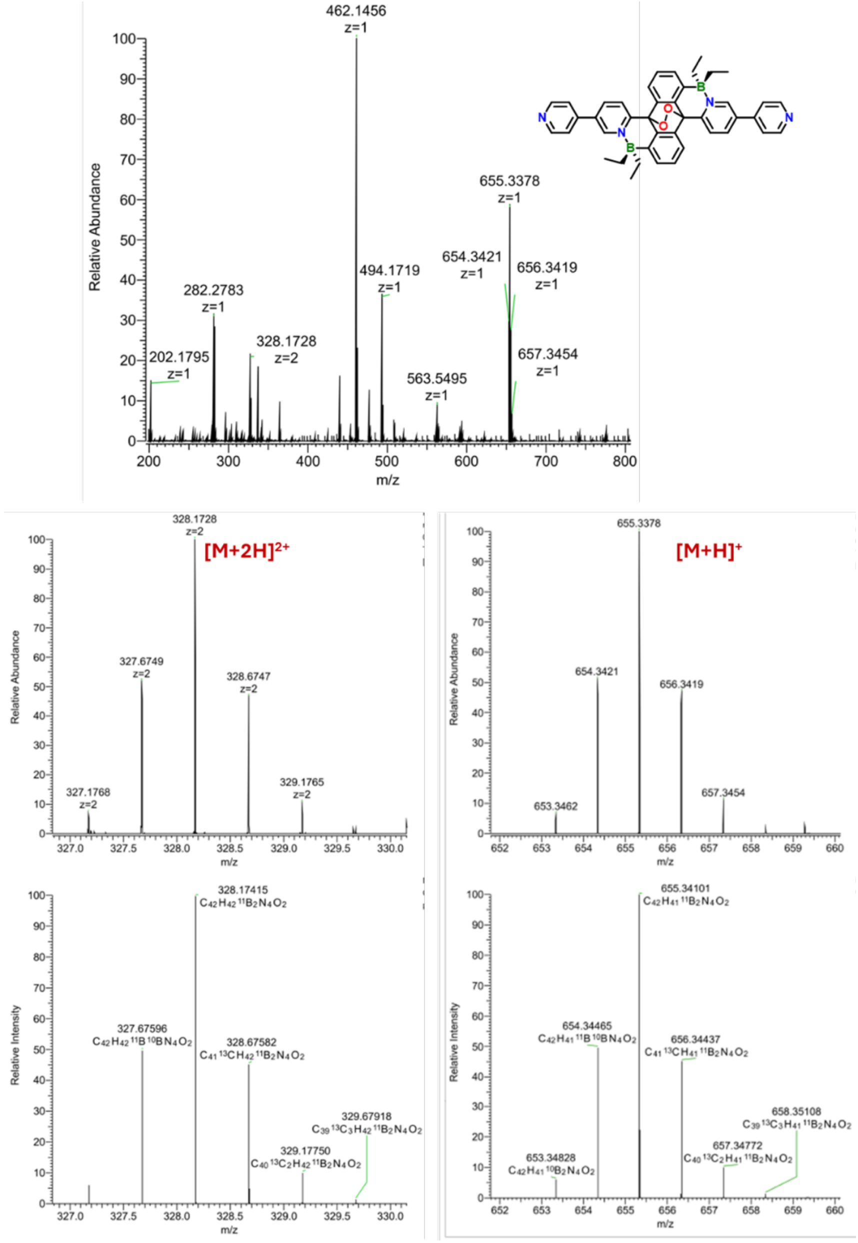

Figure S29. HRMS (ESI, positive mode) data for **1Py-O$_2$** in acetonitrile.

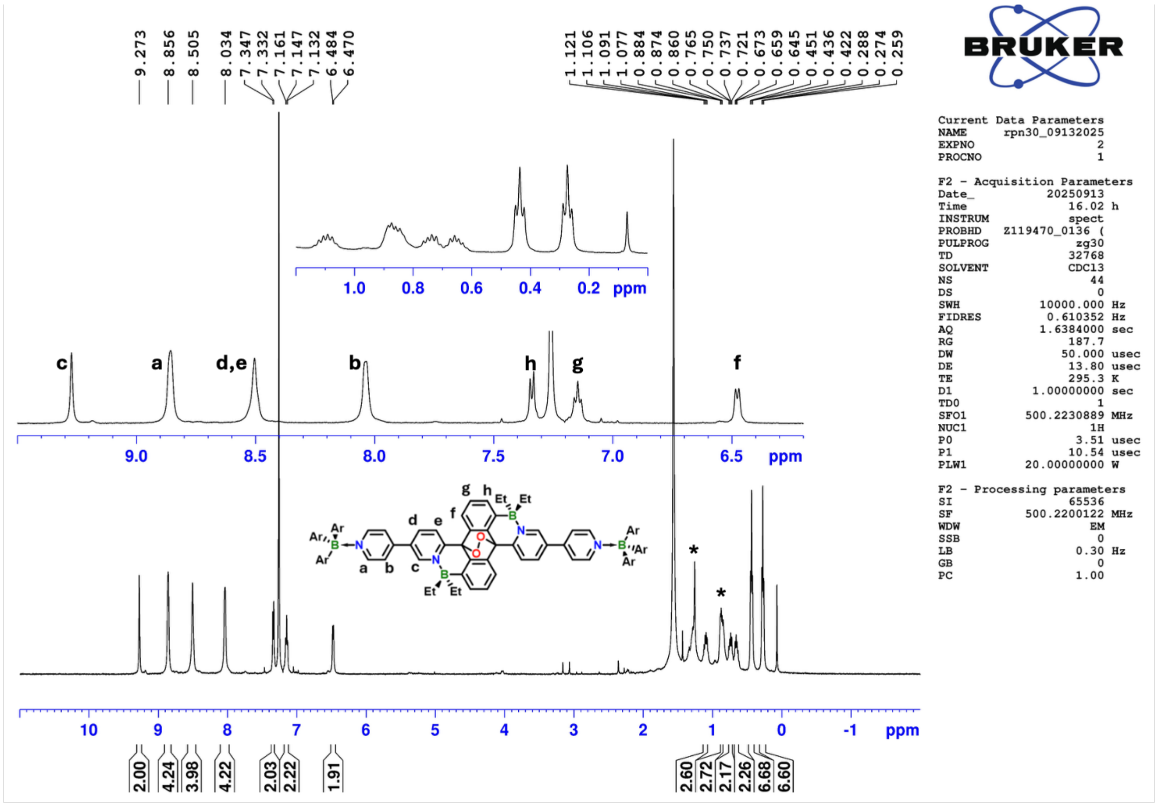

Figure S30. ^{1}H NMR spectrum of **1Py-BCF-O$_2$** in $CDCl_3$ at 25 °C (* residual hexanes).

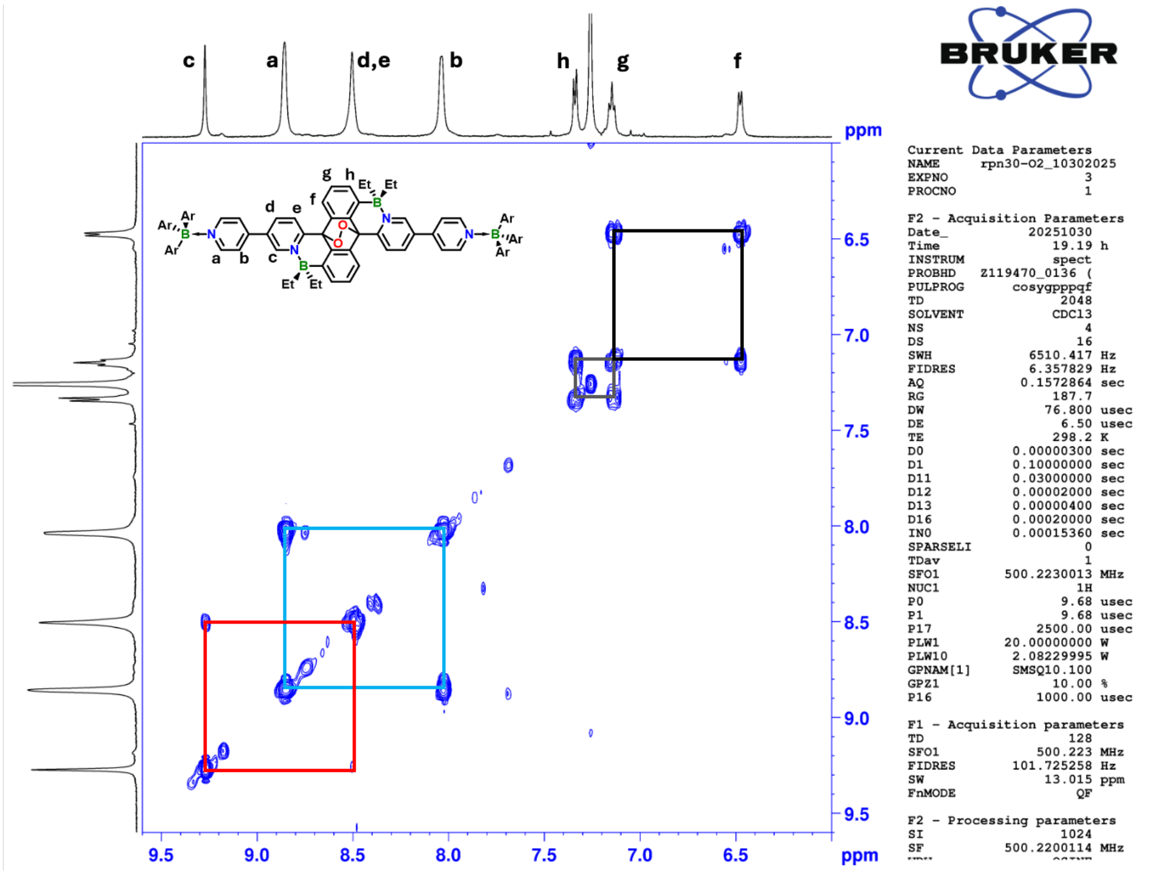

Figure S31. Aromatic region of the gCOSY NMR spectrum of **1Py-BCF-O$_2$** in $CDCl_3$ at 25 °C.

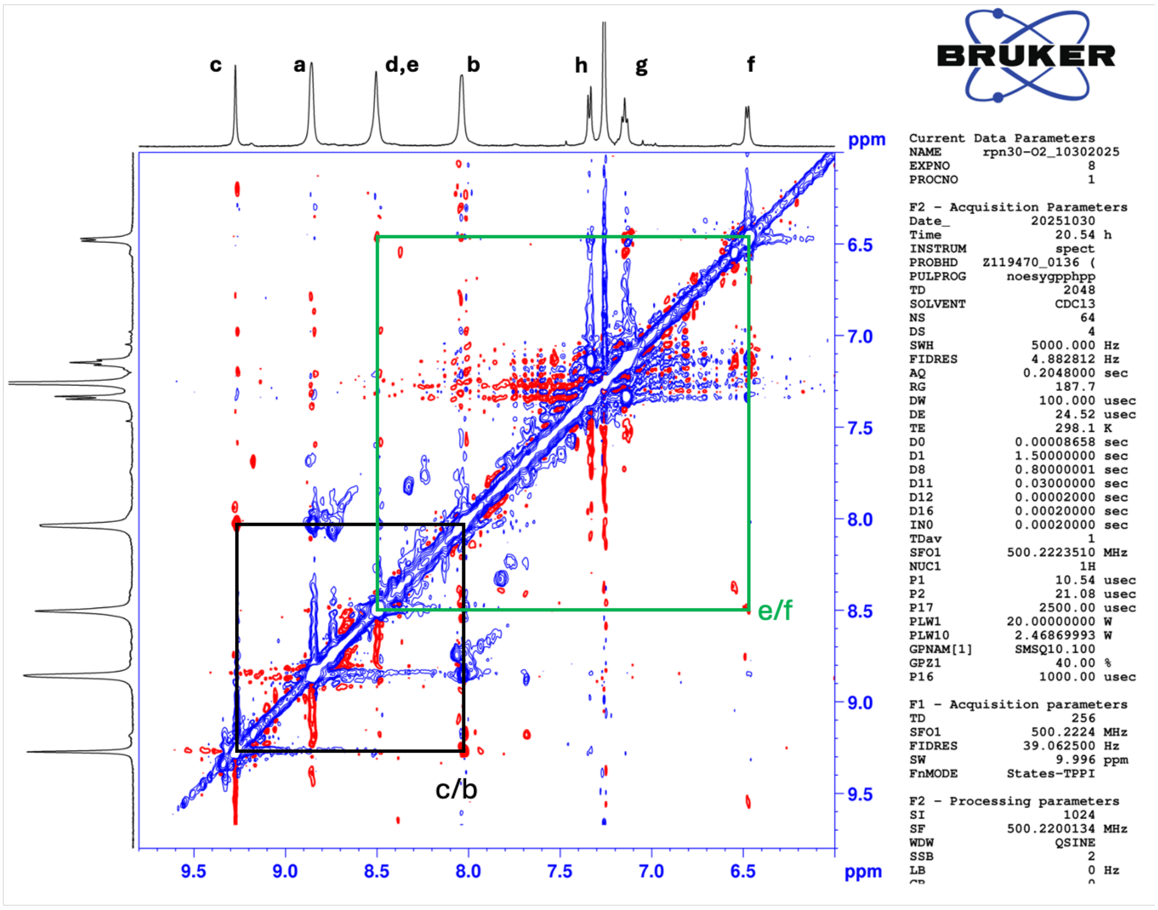

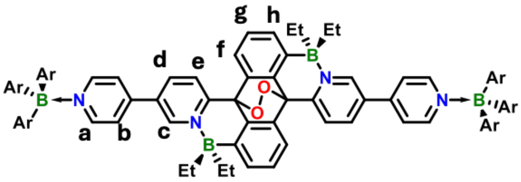

Figure S32. Expansion of the HH-NOESY NMR spectrum **1Py-BCF-O$_2$** in $CDCl_3$ at 25 °C; NOE peaks between Py$_B$-H(e) and anthracene-H(f) protons indicated with green lines; NOE peaks between Py$_B$-H(c) and ligand Py$_t$-H(b) protons indicated with black lines.

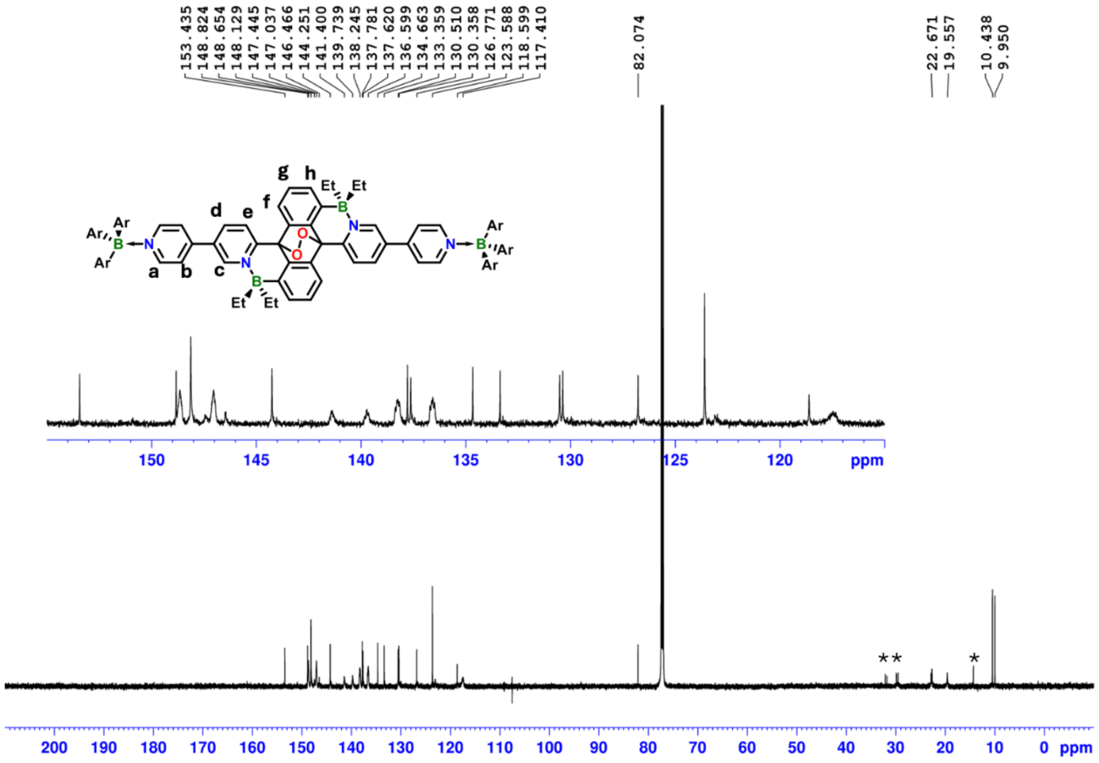

Figure S33. $^{13}C\{^{1}H\}$ NMR spectrum of **1Py-BCF-O$_2$** in $CDCl_3$ at 25 °C (* residual hexanes).

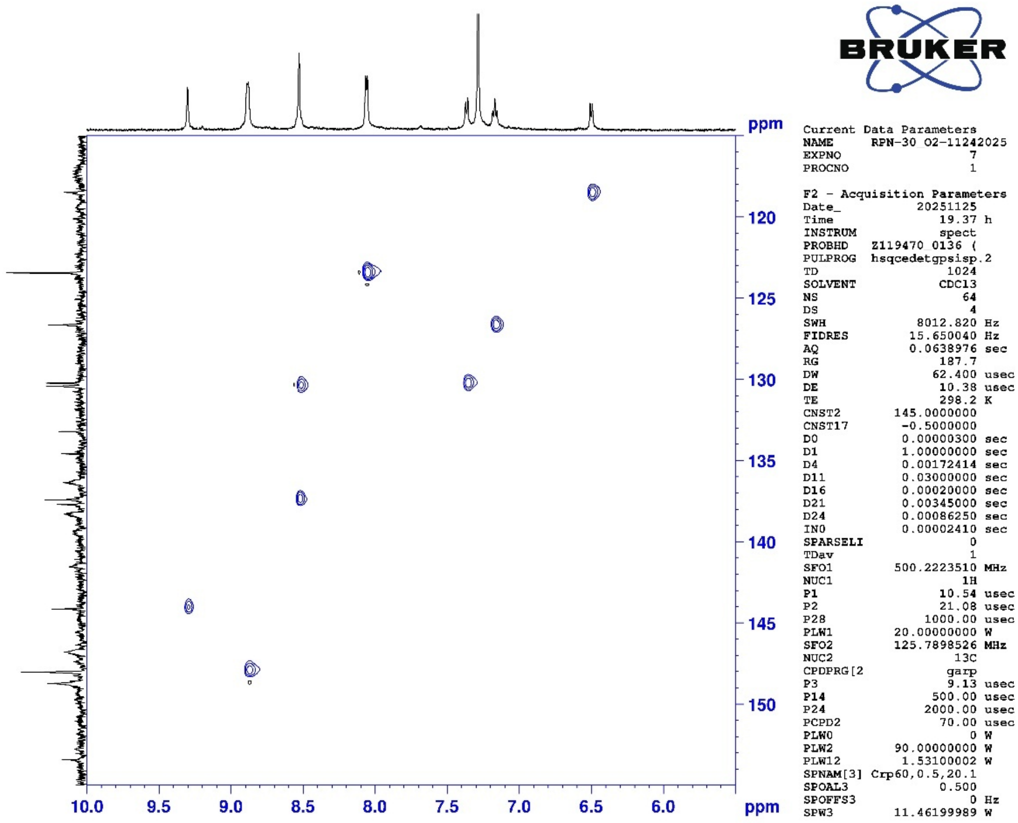

Figure S34. Aromatic region of the HSQC NMR spectrum of **1Py-BCF-O$_2$** in $CDCl_3$ at 25 °C.

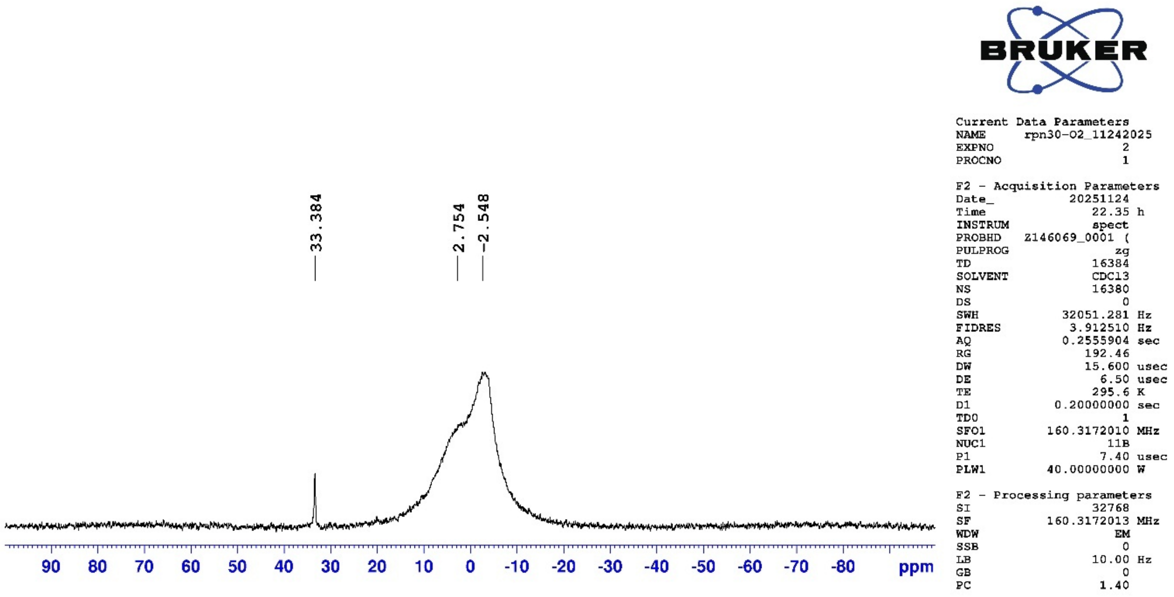

Figure S35. ^{11}B NMR spectrum of **1Py-BCF-O$_2$** in $CDCl_3$ at 25 °C.

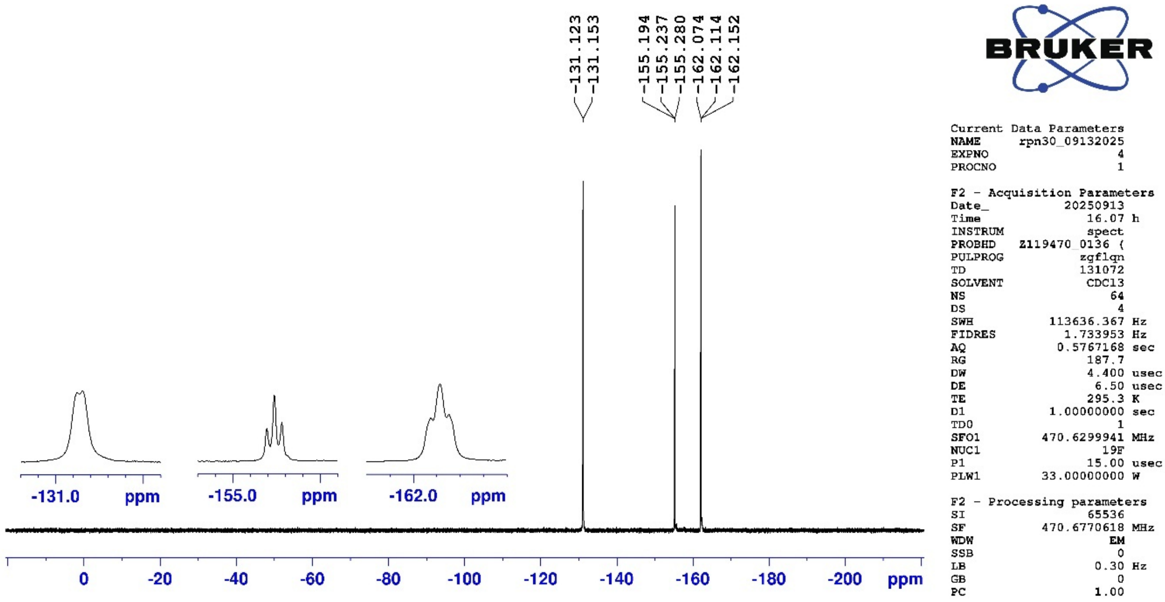

Figure S36. ^{19}F NMR spectrum of **1Py-BCF-O$_2$** in $CDCl_3$ at 25 °C.

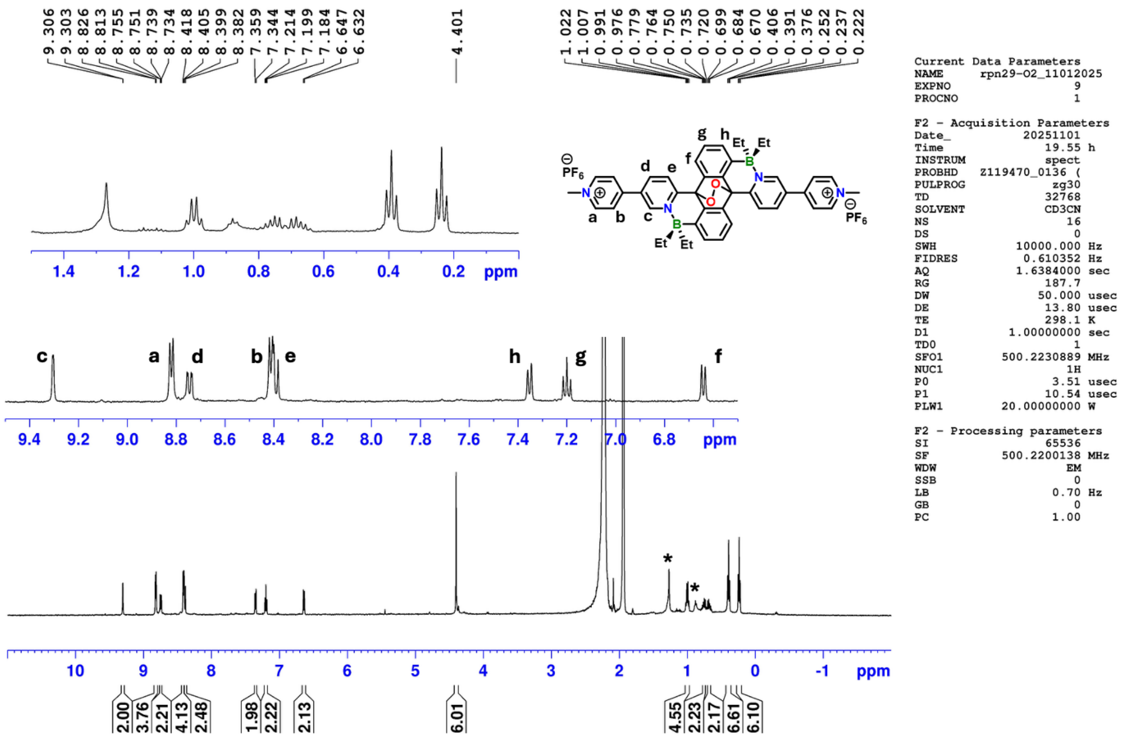

Figure S37. ^{1}H NMR spectrum of **1Py-Me^{2+}-O$_2$** in CD_3CN at 25 °C (* residual hexanes).

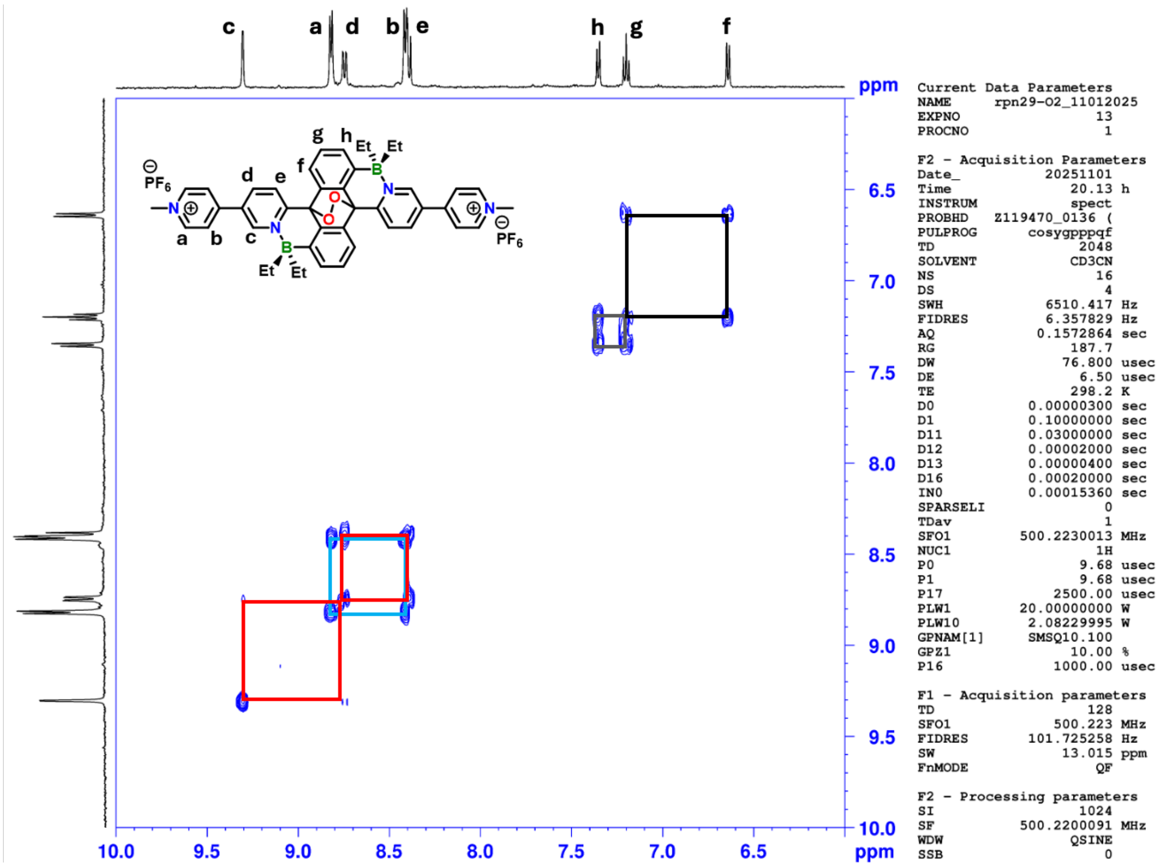

Figure S38. Aromatic region of the gCOSY NMR spectrum of **1Py-Me^{2+}-O$_2$** in CD_3CN at 25 °C.

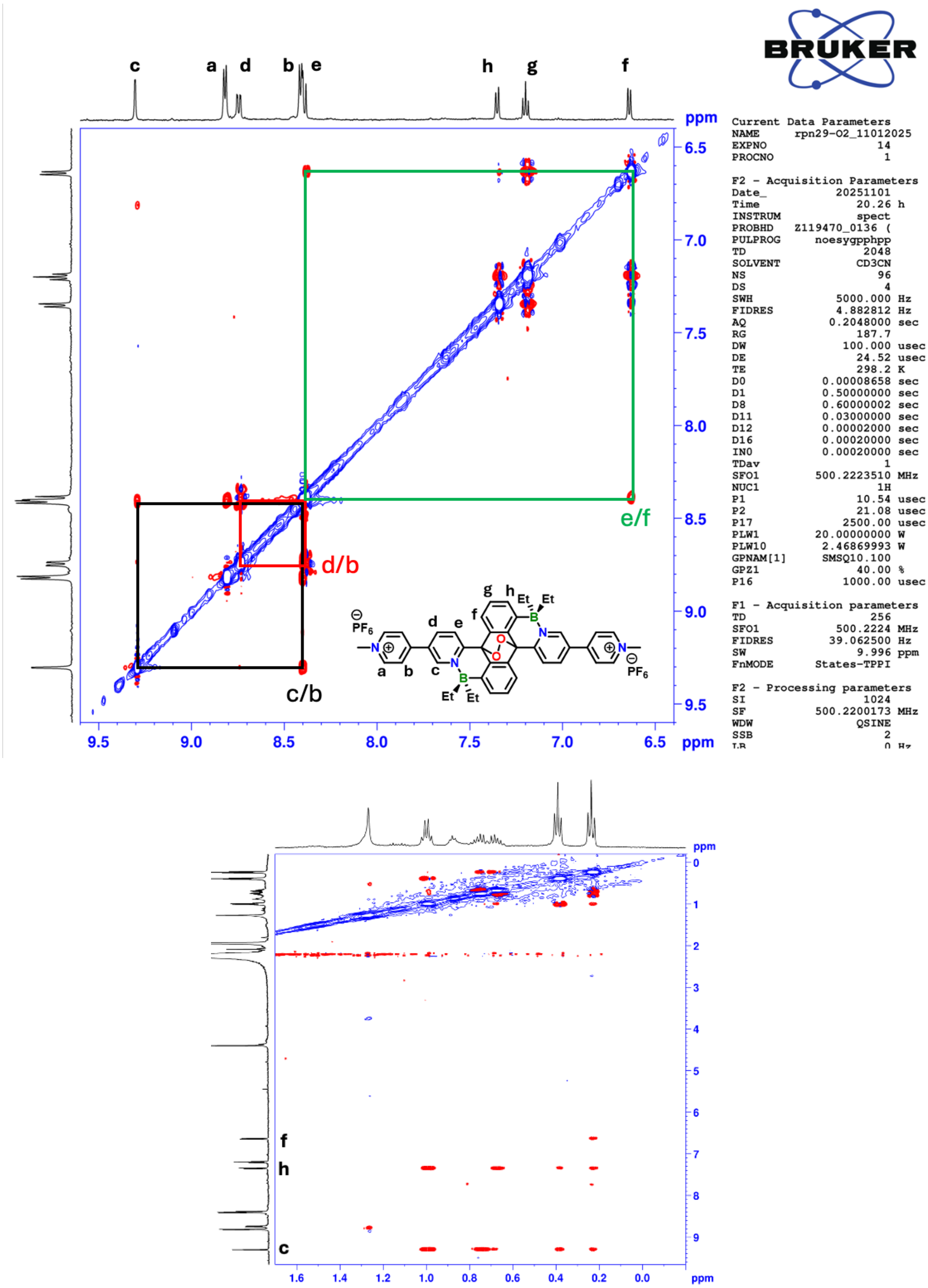

Figure S39. Expansions of the HH-NOESY NMR spectrum **1Py-Me^{2+}-O$_2$** in CD_3CN at 25 °C; (top) NOE peaks between Py$_B$-H(e) and anthracene-H(f) protons indicated with green lines; NOE peaks between Py$_B$-H(c) and ligand Py$_t$-H(b) protons indicated with black lines, and Py$_B$-H(d) and ligand Py$_t$-H(b) protons indicated with red lines; (bottom) the aliphatic region shows exchange peaks due to interconversion of B-Et substituents. NOE peaks between aliphatic protons and Py$_B$-H(c) / anthracene-H(h) are also observed.

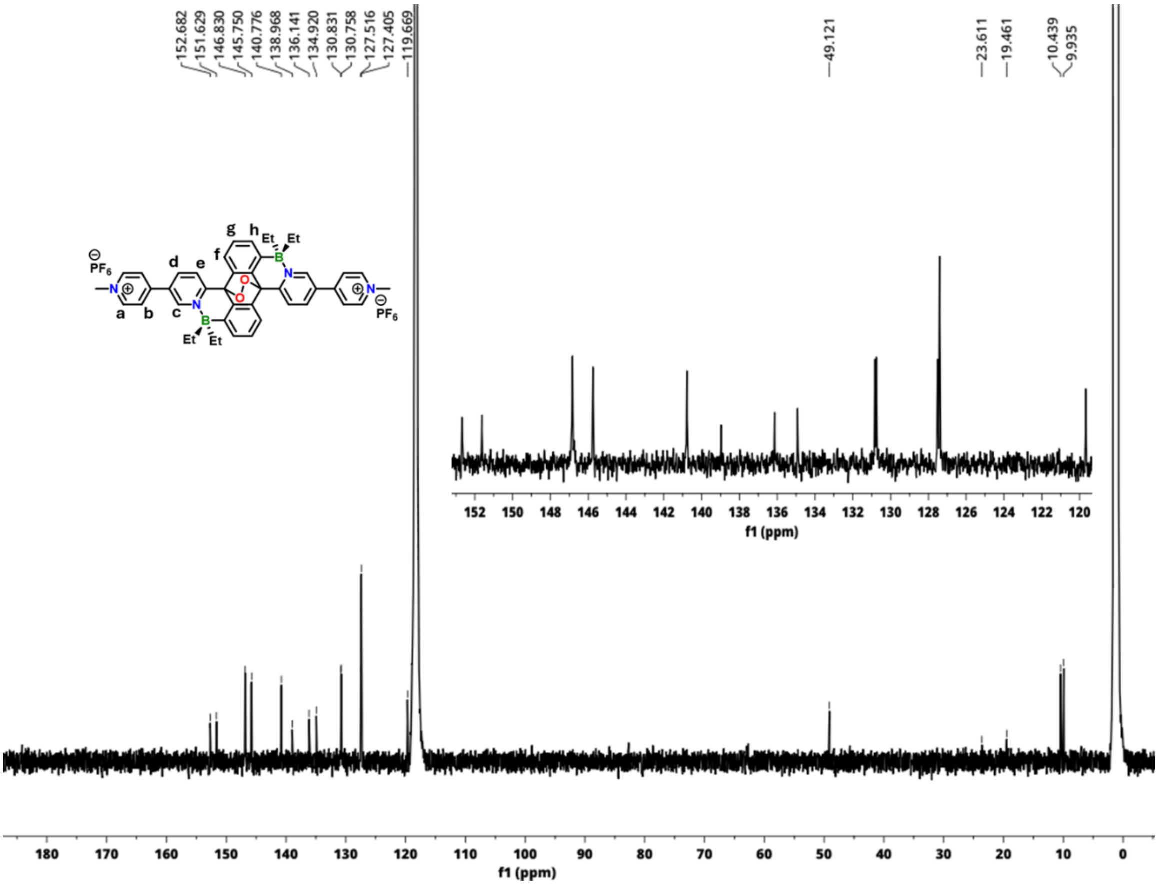

Figure S40. $^{13}C\{^1H\}$ NMR spectrum of **1Py-Me^{2+}-O$_2$** in CD_3CN at 25 °C.

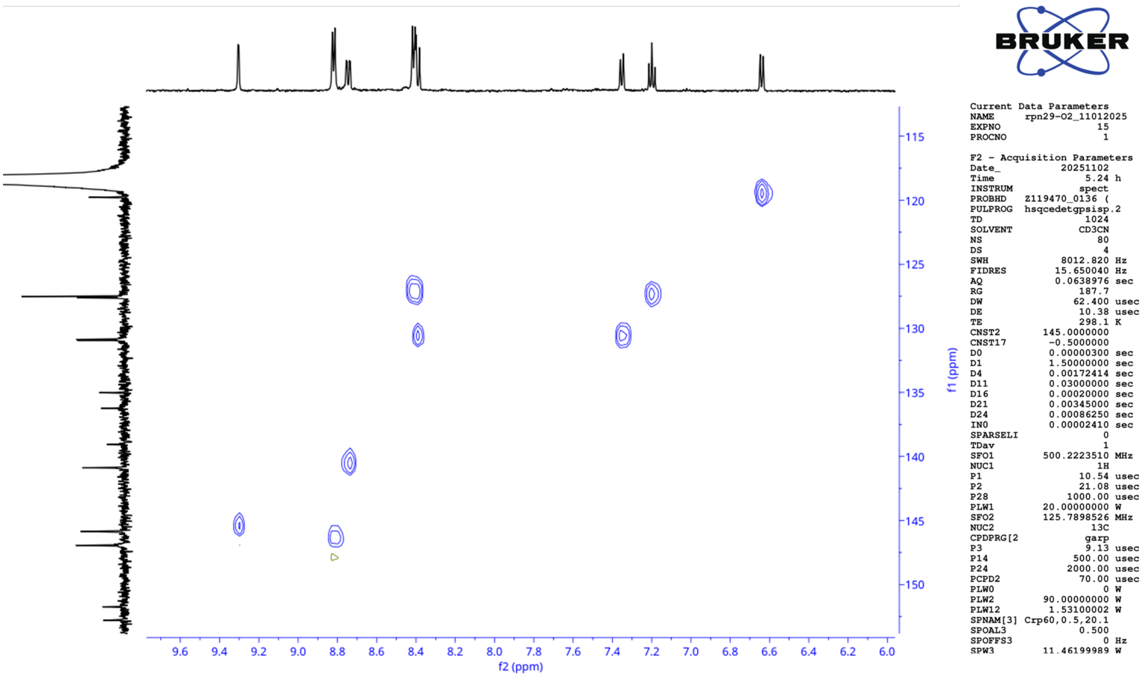

Figure S41. Aromatic region of the HSQC NMR spectrum of **1Py-Me^{2+}-O$_2$** in CD_3CN at 25 °C.

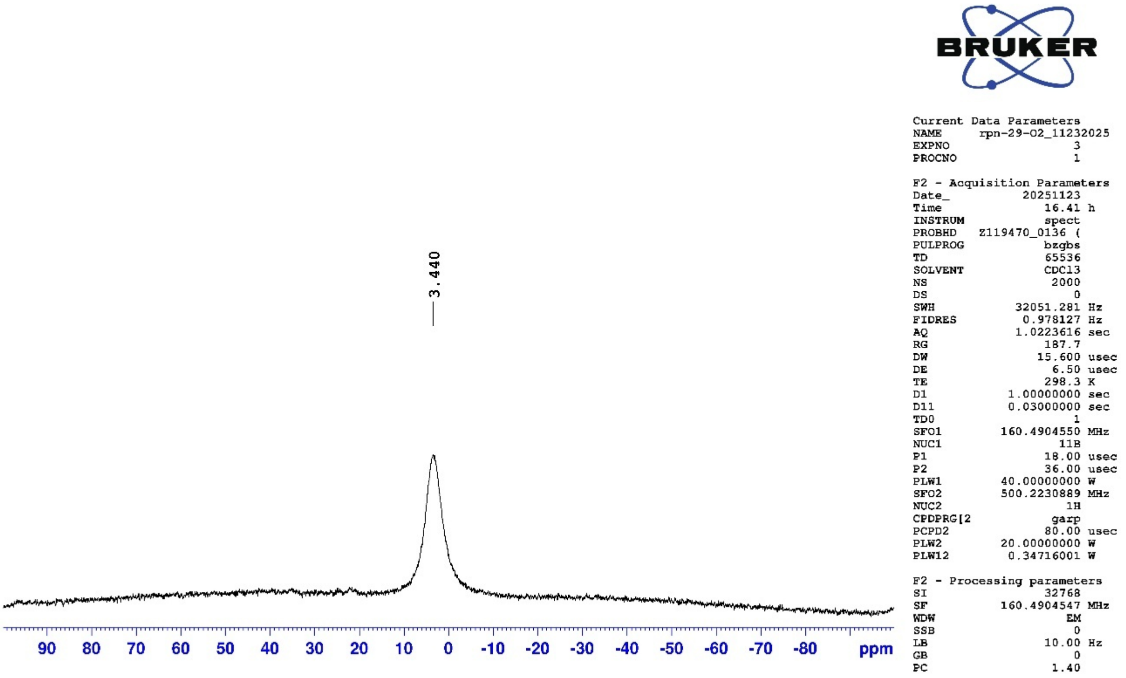

Figure S42. ^{11}B NMR spectrum of **1Py-Me^{2+}-O$_2$** in CD_3CN at 25 °C.

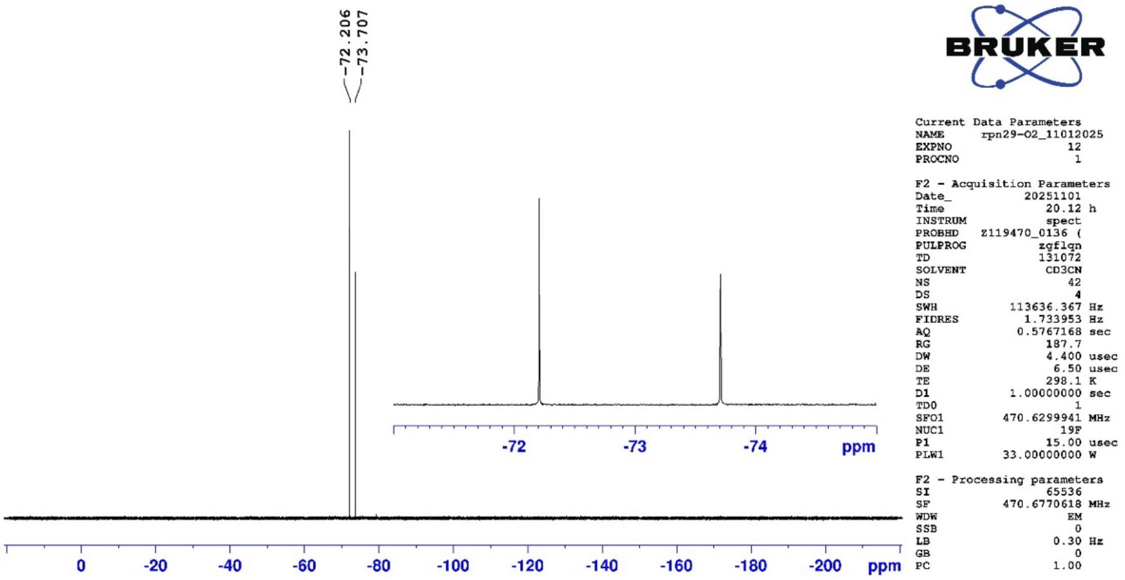

Figure S43. ^{19}F NMR spectrum of **1Py-Me^{2+}-O$_2$** in CD_3CN at 25 °C.

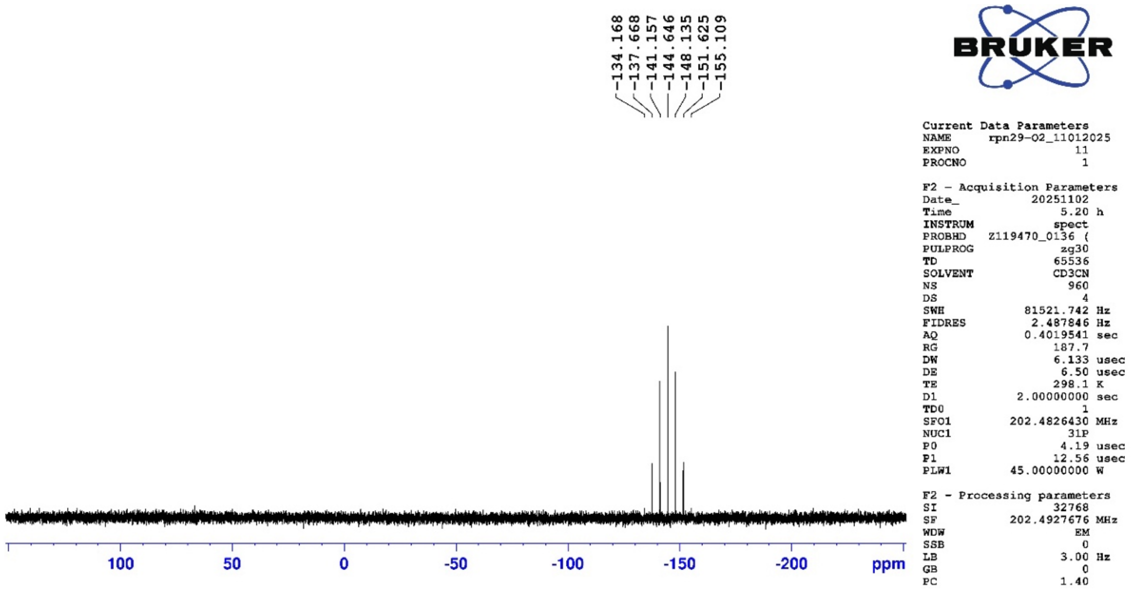

Figure S44. ^{31}P NMR spectrum of **1Py-Me^{2+}-O$_2$** in CD_3CN at 25 °C.

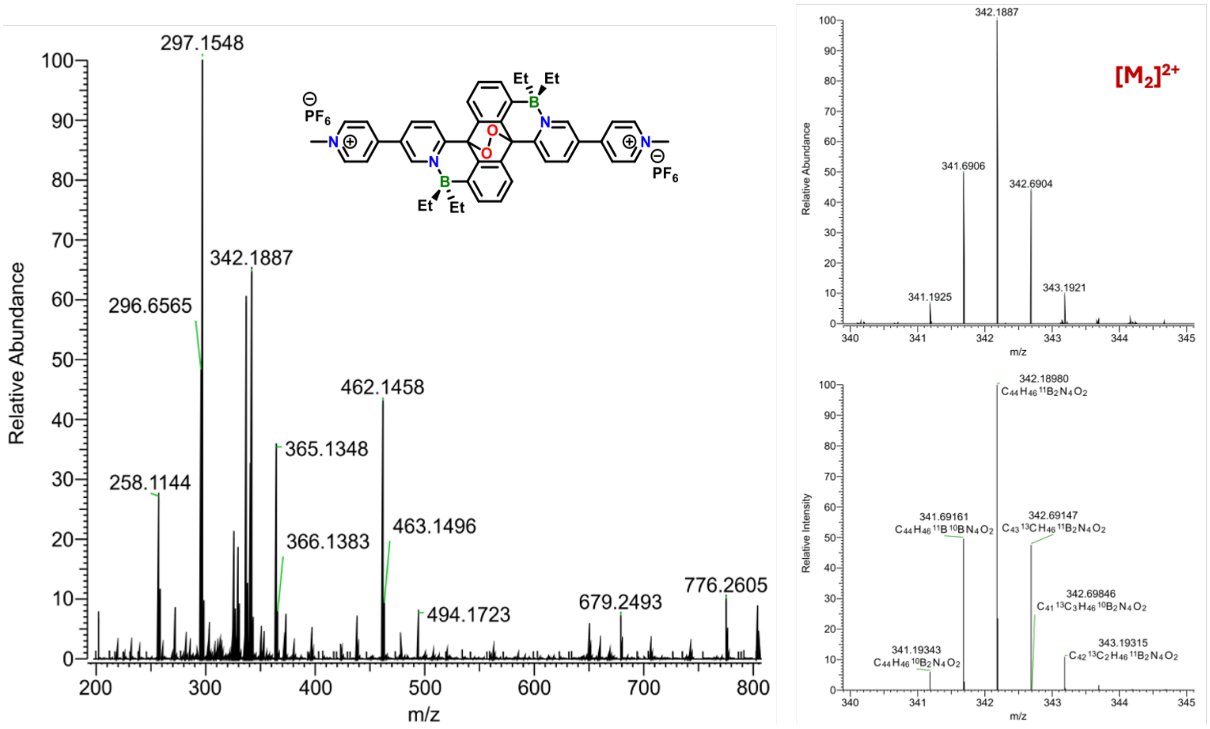

Figure S45. HRMS (ESI, positive mode) data for dicationic **1Py-Me^{2+}-O$_2$** in acetonitrile.

Single Crystal XRD Data

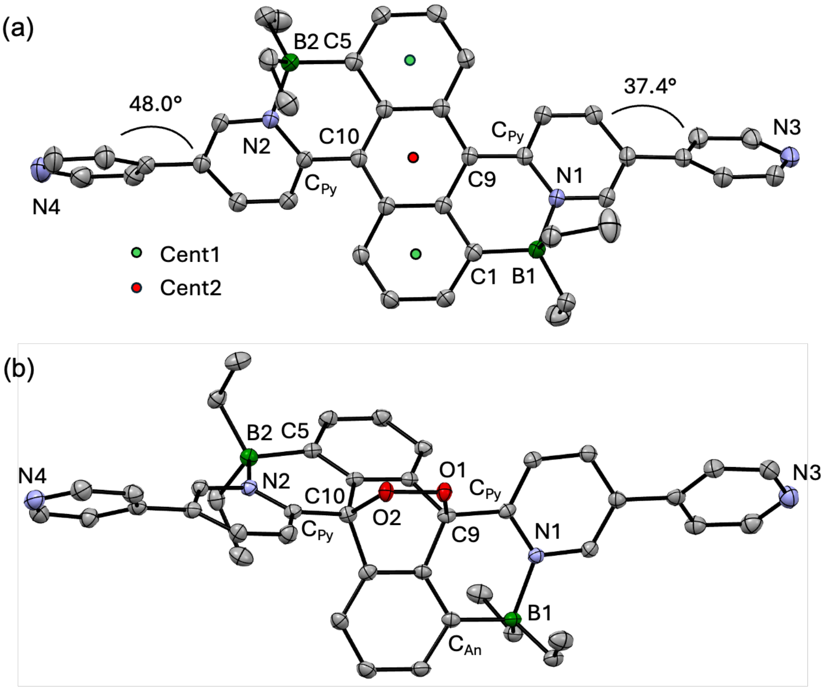

Figure S46. Plots of the X-ray crystal structures of (a) **1Py** (CCDC 2485272) and (b) **1Py-O_2** (CCDC 2505729); 50% thermal ellipsoids, H atoms and a cocrystallized $CHCl_3$ solvent molecule for **1Py-O_2** omitted for clarity.

Table S1. Geometric parameters (distances in Å, angles in °) obtained from X-ray crystal structure analysis of **1Py** and comparison with data for **1H**.[7]

	B-N	B-C_{An}	C_{An}-C_{Py}	α[a]	β[b]	γ[c]	δ[d]	ε[e]	ϕ[f]
**1H** [7]	1.632(2)	1.618(2)	1.480(2)	106.5(1)	169.1	168.6	16.5	23.6	36.8
	1.632(2)	1.618(2)	1.480(2)	106.5(1)	169.1	168.6			36.8
**1Py**	1.634(2)	1.613(3)	1.472(2)	106.3(1)	173.1	164.0	17.7	20.2	34.4
	1.645(2)	1.612(3)	1.467(2)	107.1(1)	174.1	166.1			36.4
**1Py-O_2**	1.655(4)	1.594(4)	1.507(4)	107.5(2)	178.0		56.9	56.8	
	1.664(4)	1.606(4)	1.507(4)	106.9(2)	174.2				

[a] α = C_{An}-B-N angle; [b] β = Cent1-C1-B; [c] γ = Cent2-C11-C_{Py}; [d] δ = internal bending of central anthracene ring; [e] ε = $Ph_{An,out}$ // $Ph_{An,out}$, dihedral angle between outer anthracene rings; [f] ϕ = $Ph_{An,in}$ // Py, interplanar angle between inner anthracene ring and pendent pyridyl ring.

Photophysical Properties

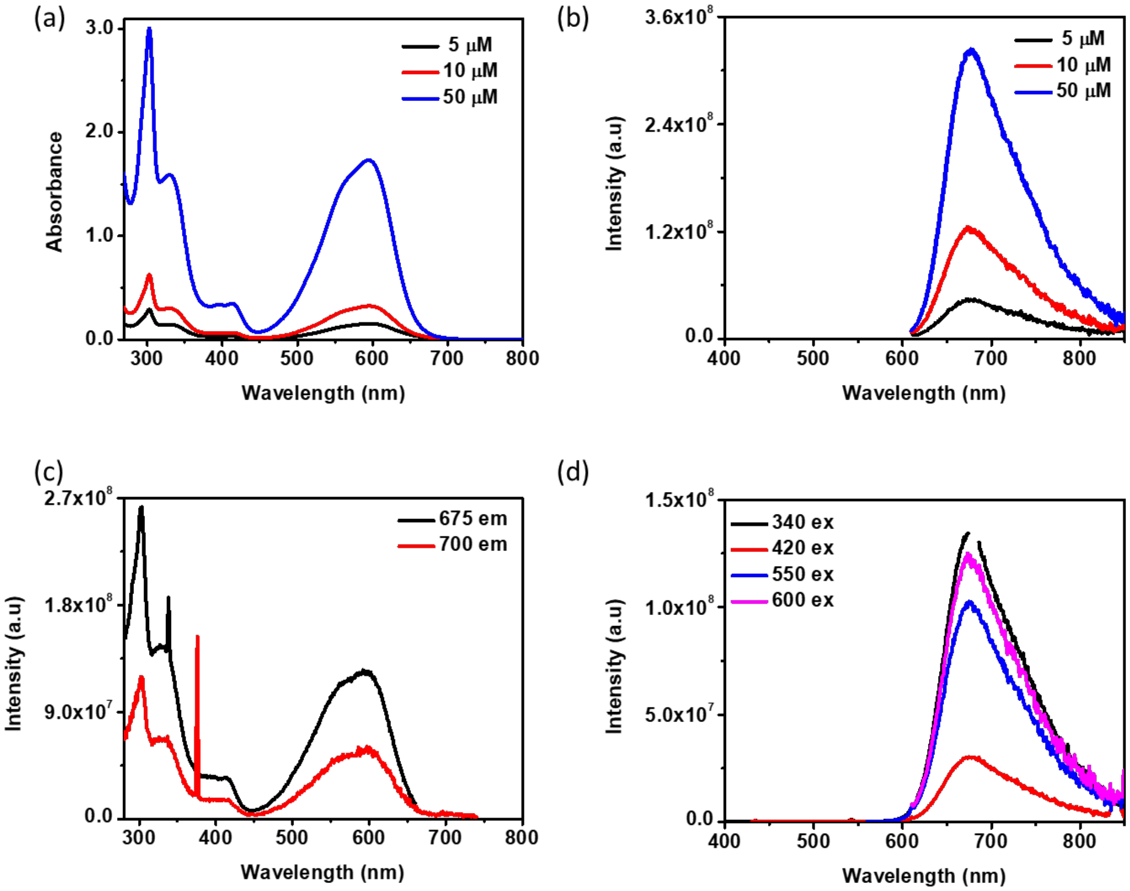

Figure S47. (a) Absorption and (b) emission (λ_{ex} = 600 nm) spectra of **1Py** in dichloromethane at different concentrations. (c) Excitation spectra of **1Py** (conc. = 10 μM) in dichloromethane collected at different emission wavelengths. (d) Emission spectra of **1Py** (conc. = 10 μM) in dichloromethane collected at different excitation wavelengths.

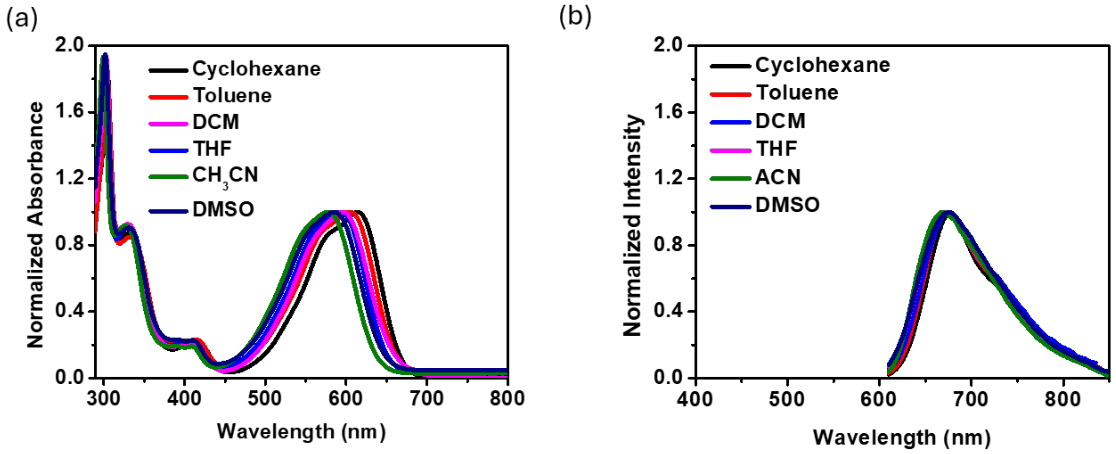

Figure S48. (a) Normalized absorption spectra and (b) emission spectra (λ_{ex} = 600 nm) of **1Py** in different solvents (conc. = 10 μM).

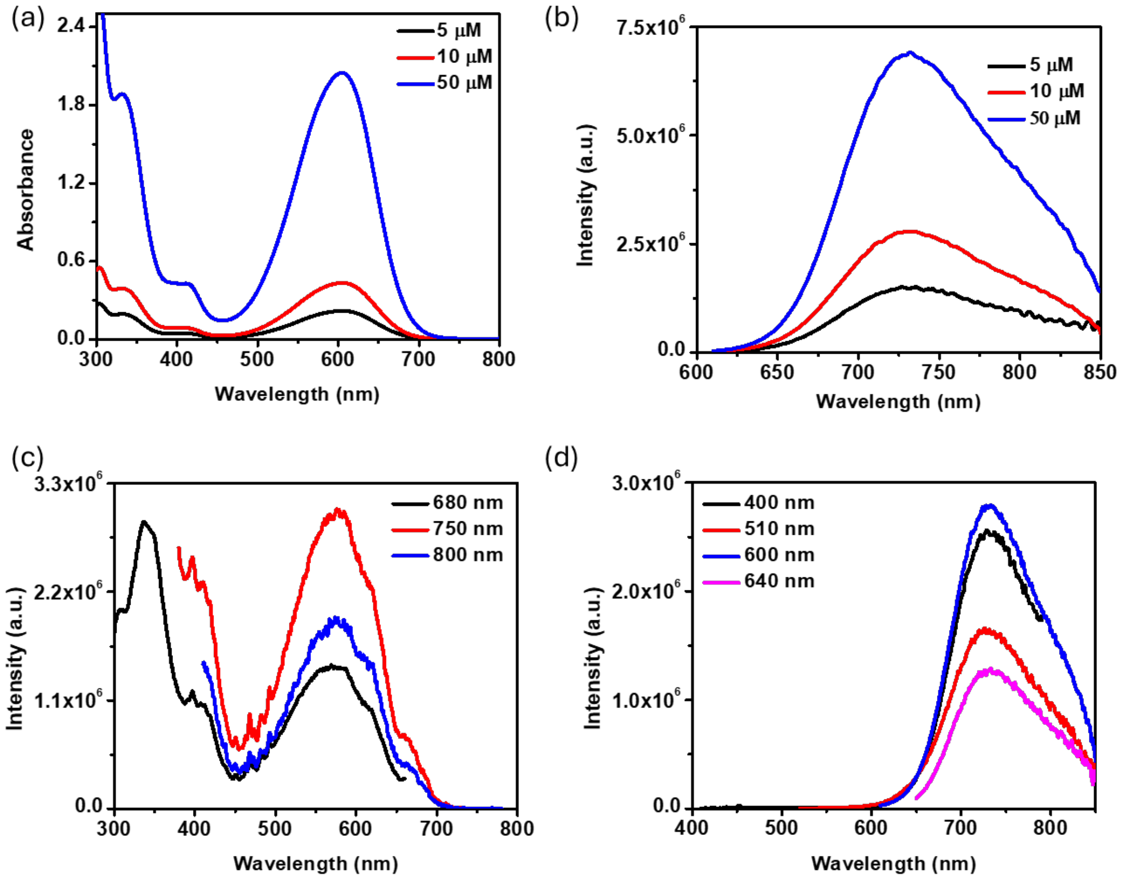

Figure S49. (a) Absorption and (b) emission (λ_{ex} = 600 nm) spectra of **1Py-BCF** in acetonitrile at different concentrations. (c) Excitation spectra of **1Py-BCF** (conc. = 10 μM) in acetonitrile collected at different emission wavelengths. (d) Emission spectra of **1Py-BCF** (conc. = 10 μM) in acetonitrile collected at different excitation wavelengths.

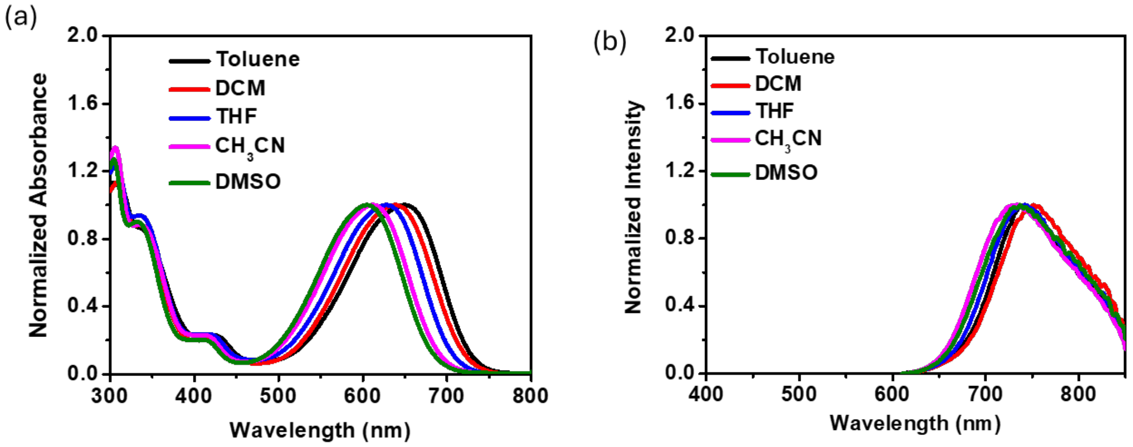

Figure S50. (a) Normalized absorption spectra and (b) emission spectra (λ_{ex} = 600 nm) of **1Py-BCF** in different solvents (conc. = 10 μM).

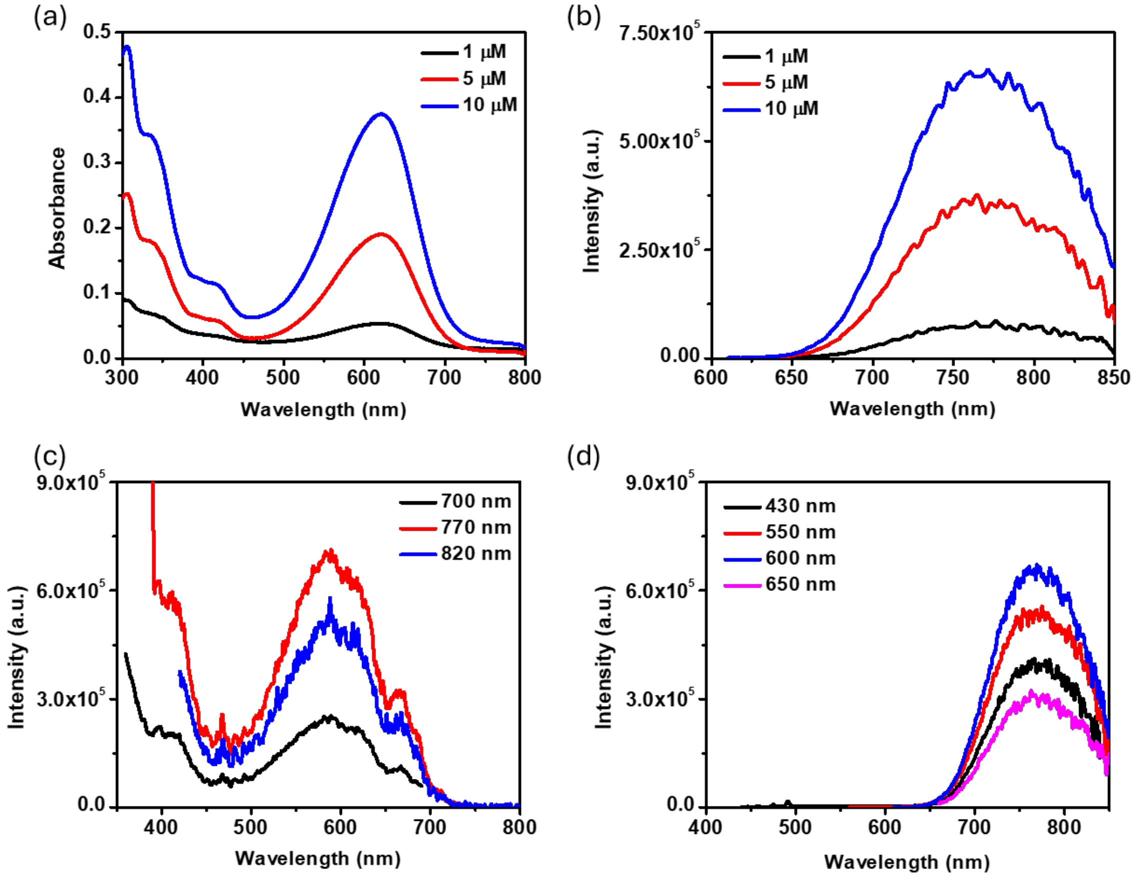

Figure S51. (a) Absorption and (b) emission (λ_{ex} = 600 nm) spectra of **1Py-Me^{2+}** in acetonitrile at different concentrations. (c) Excitation spectra of **1Py-Me^{2+}** (conc. = 10 μM) in acetonitrile collected at different emission wavelengths. (d) Emission spectra of **1Py-Me^{2+}** (conc. = 10 μM) in acetonitrile collected at different excitation wavelengths.

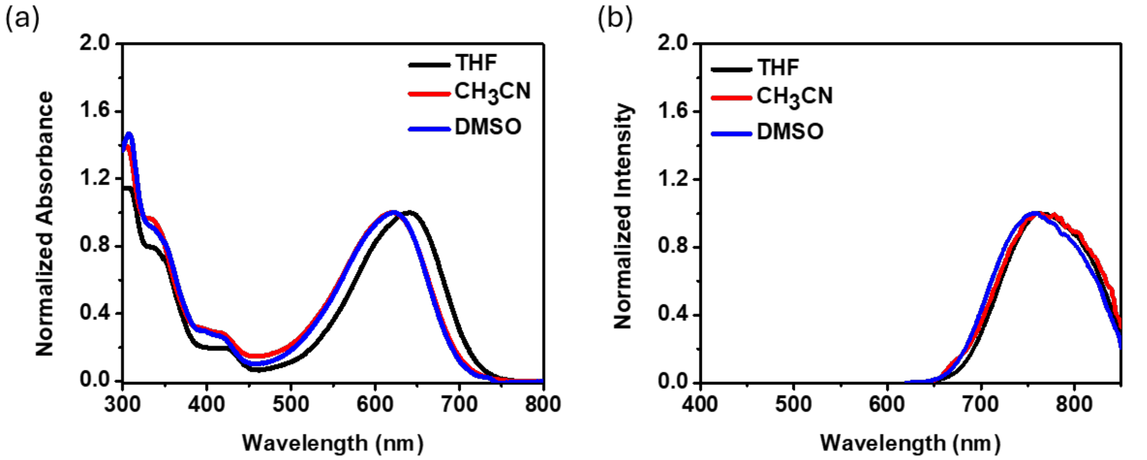

Figure S52. (a) Normalized absorption spectra and (b) emission spectra (λ_{ex} = 600 nm) of **1Py-Me^{2+}** in different solvents (conc. = 10 μM). Note that THF, acetonitrile and DMSO were chosen because of the low solubility of **1Py-Me^{2+}** in less polar solvents.

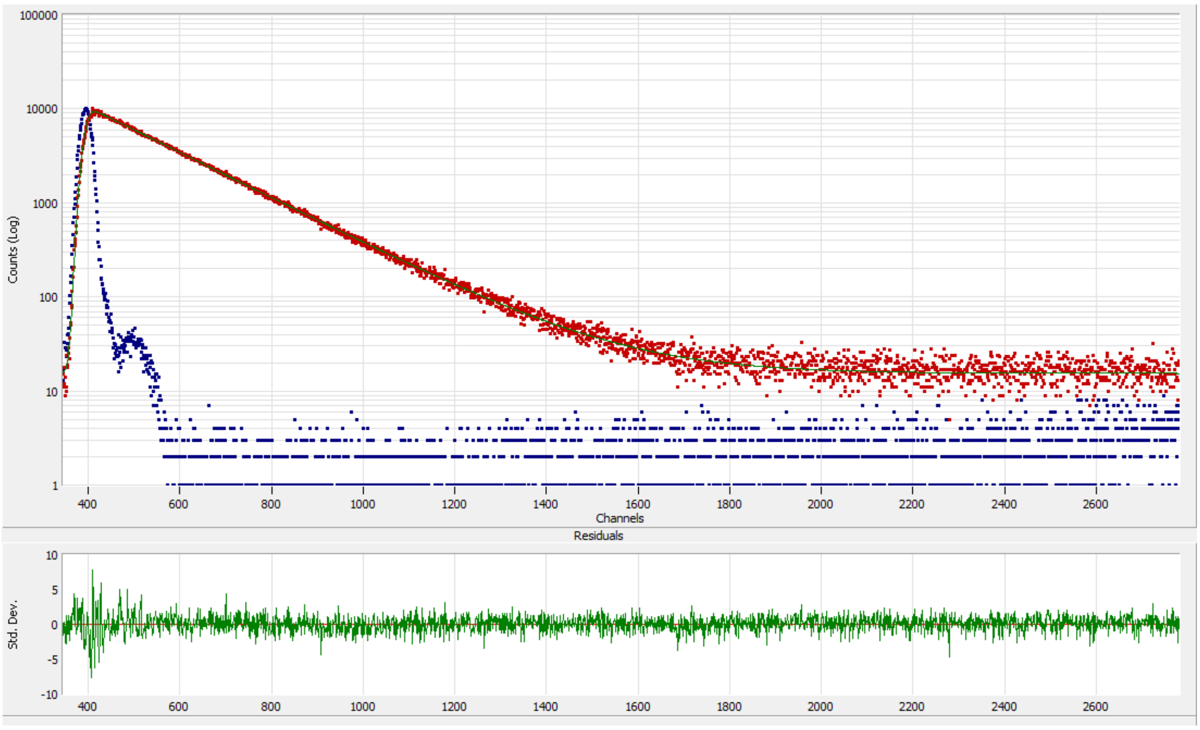

Figure S53. Single-exponential fit of fluorescence decay of **1H** in acetonitrile excited with a 390 nm nanoLED (data points shown in red, prompt shown in blue, fit and residuals from fit shown in green); mono-exponential fit with τ = 9.8 ns, χ^2 = 1.40.

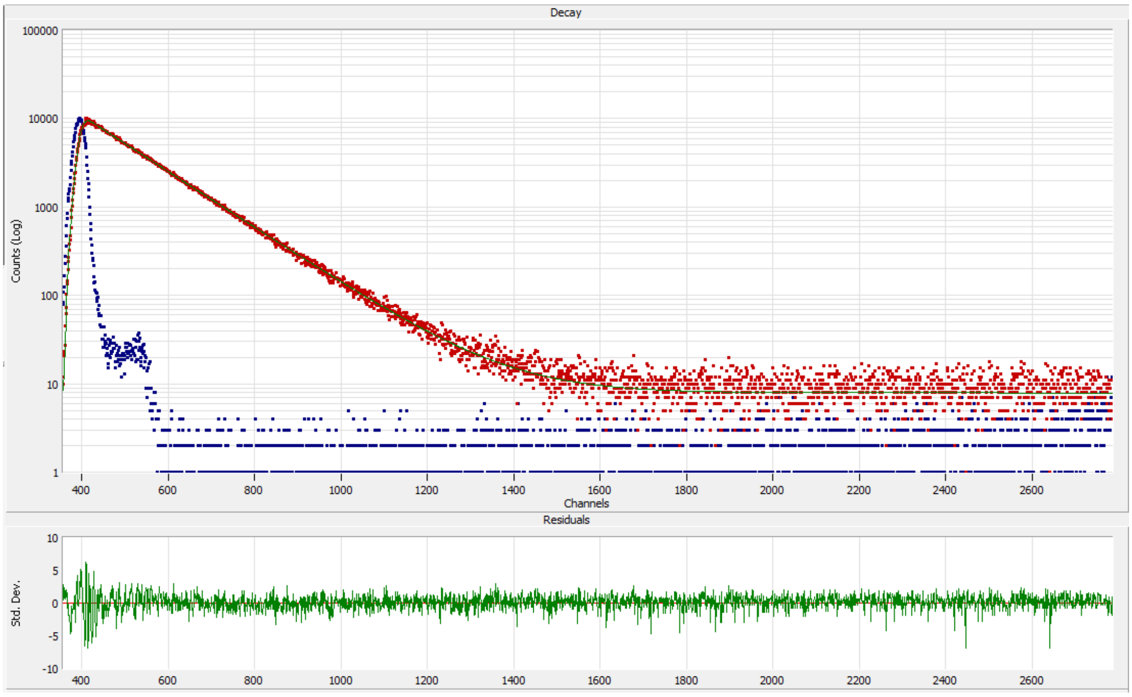

Figure S54. Single-exponential fit of fluorescence decay of **1Py** in acetonitrile excited with a 390 nm nanoLED (data points shown in red, prompt shown in blue, fit and residuals from fit shown in green); mono-exponential fit with τ = 7.5 ns, χ^2 = 1.47.

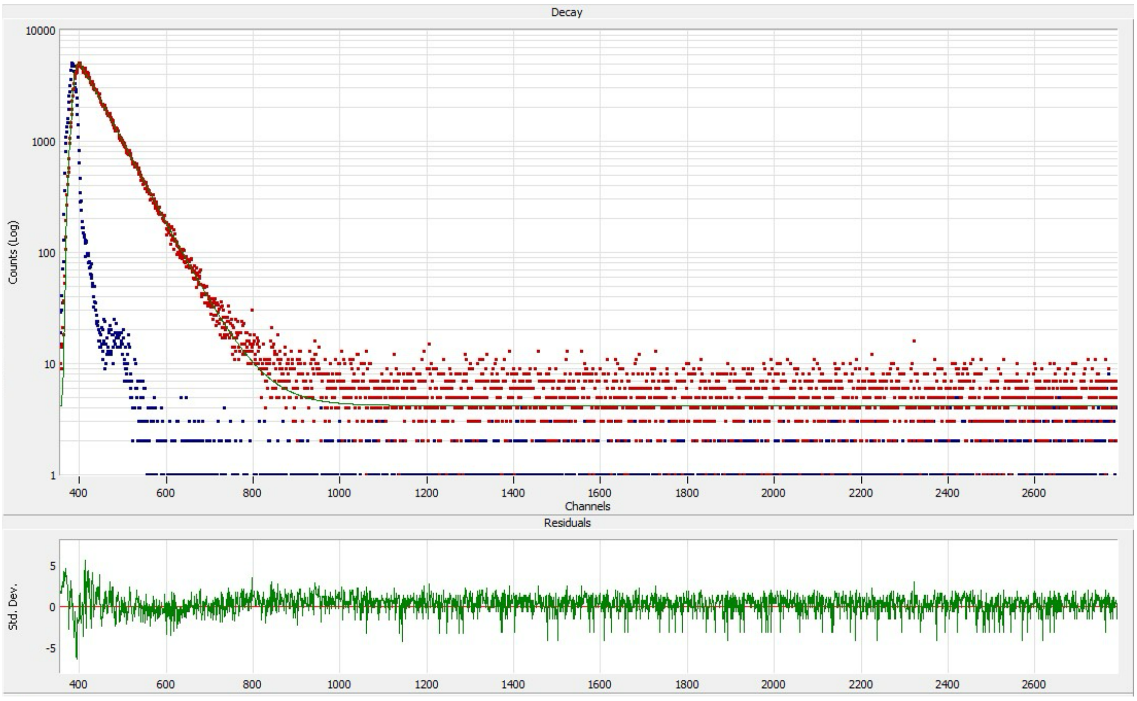

Figure S55. Single-exponential fit of fluorescence decay of **1Py-BCF** in acetonitrile excited with a 670 nm nanoLED (data points shown in red, prompt shown in blue, fit and residuals from fit shown in green); mono-exponential fit with τ = 3.2 ns, χ^2 = 1.48.

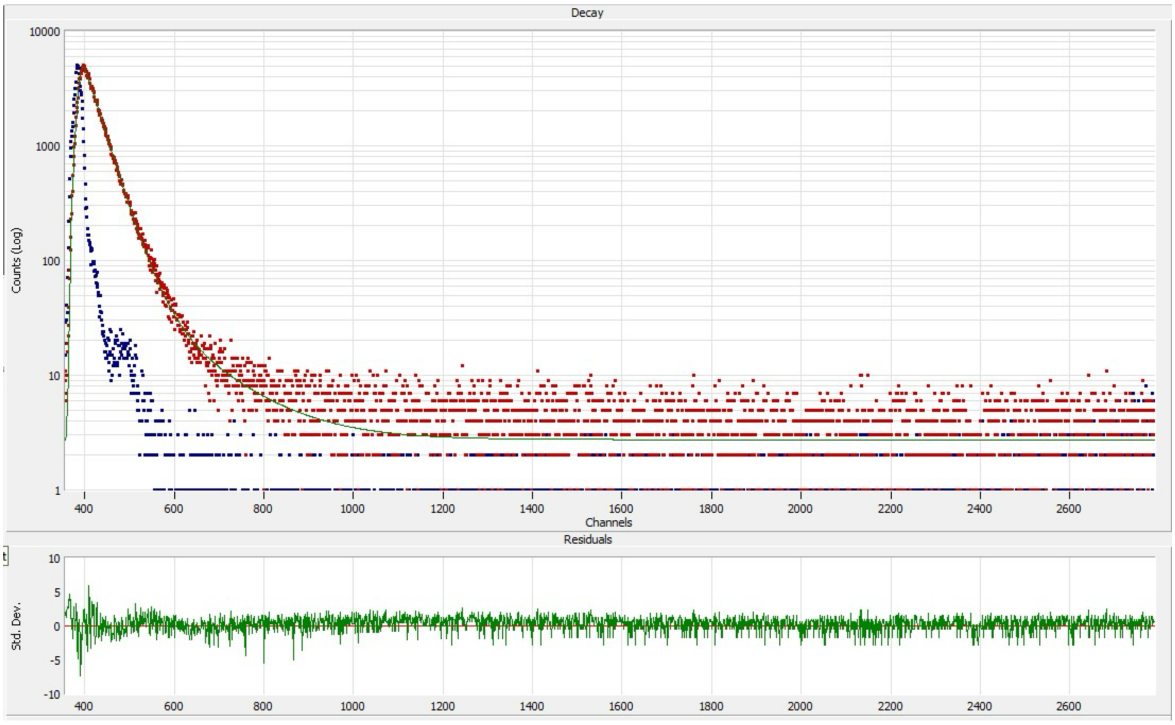

Figure S56. Biexponential fit of fluorescence decay of **1Py-Me^{2+}** in acetonitrile excited with a 670 nm nanoLED (data points shown in red, prompt shown in blue, fit and residuals from fit shown in green); biexponential fit with τ_1 = 1.8 ns (95.0), τ_2 = 6.8 ns (5.0), χ^2 = 1.40.

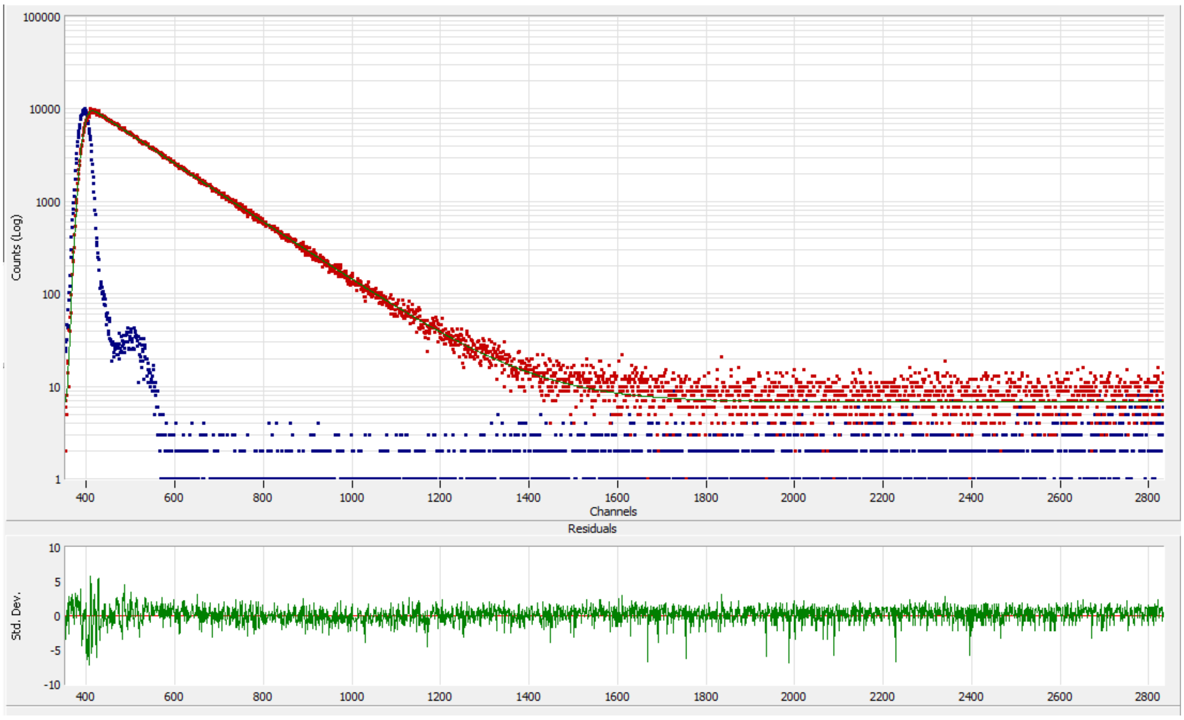

Figure . Single-exponential fit of fluorescence decay of **1Py** in dichloromethane excited with a 390 nm nanoLED (data points shown in red, prompt shown in blue, fit and residuals from fit shown in green); mono-exponential fit with τ = 7.5 ns, χ^2 = 1.52.

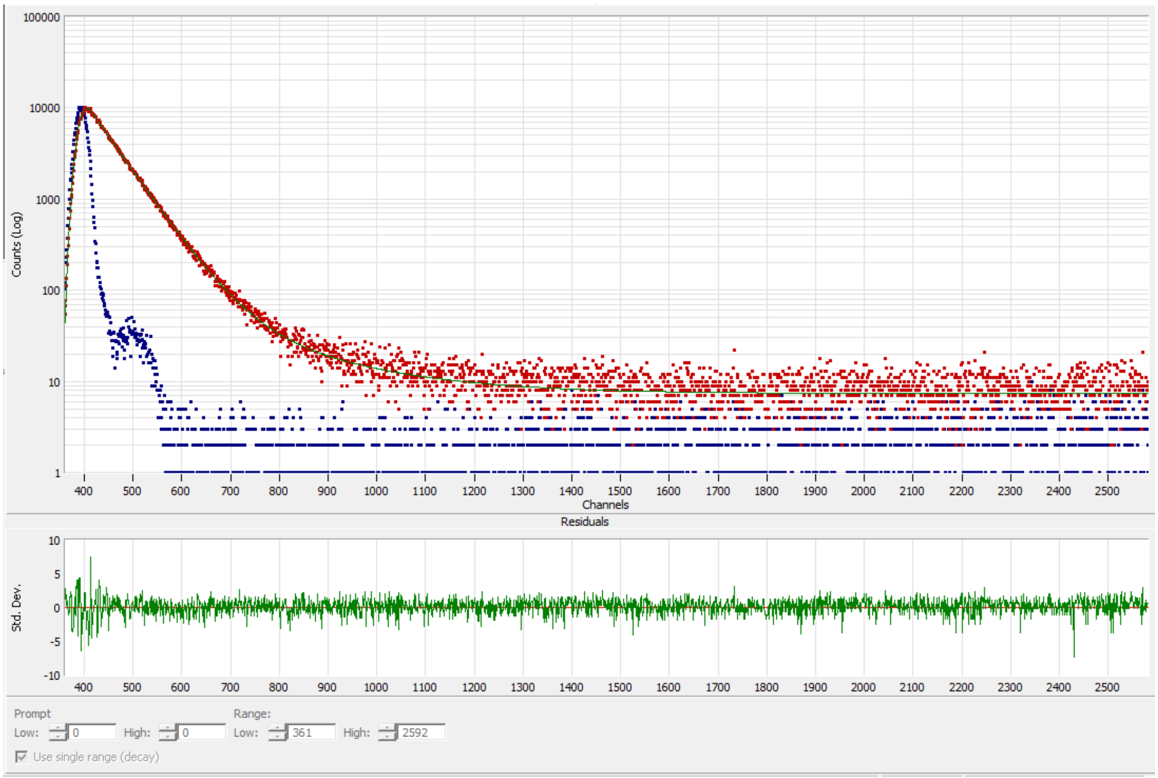

Figure S58. Single-exponential fit of fluorescence decay of **1Py-BCF** in dichloromethane excited with a 390 nm nanoLED (data points shown in red, prompt shown in blue, fit and residuals from fit shown in green); mono-exponential fit with τ = 3.1 ns, χ^2 = 1.40.

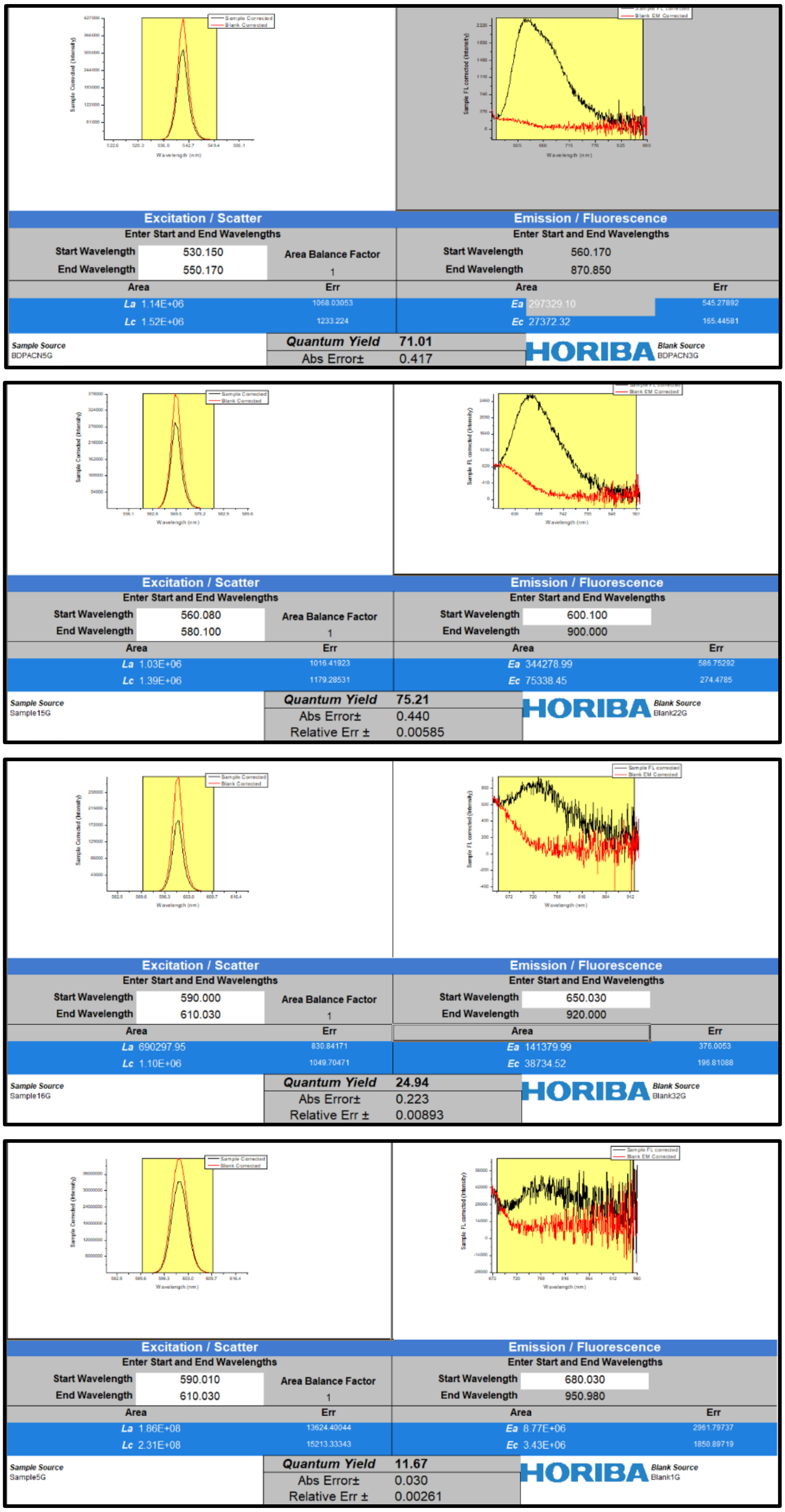

Figure S59. Absolute PLQY measurement of **1H**, **1Py**, **1Py-BCF**, and **1Py-Me**$^{2+}$ (from top to bottom) in acetonitrile.

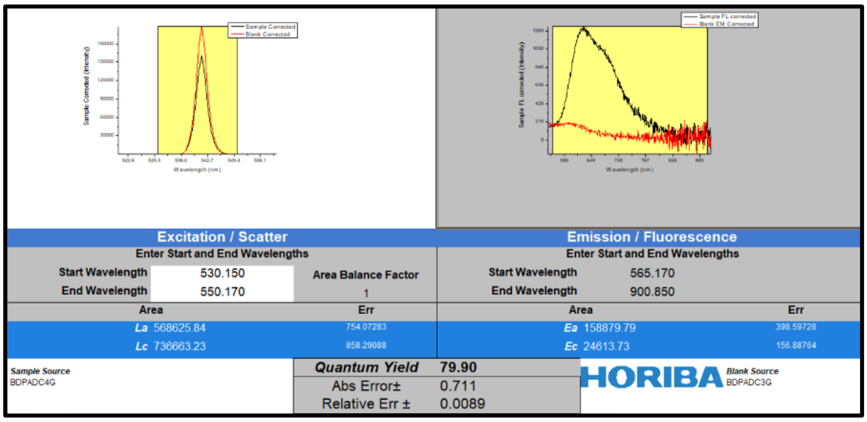

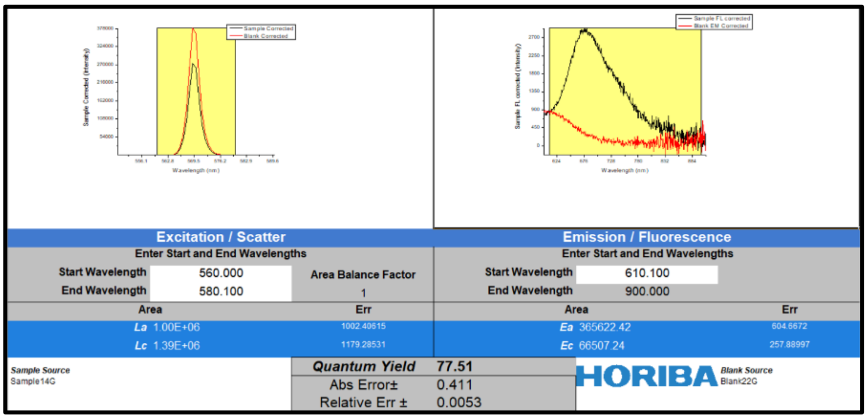

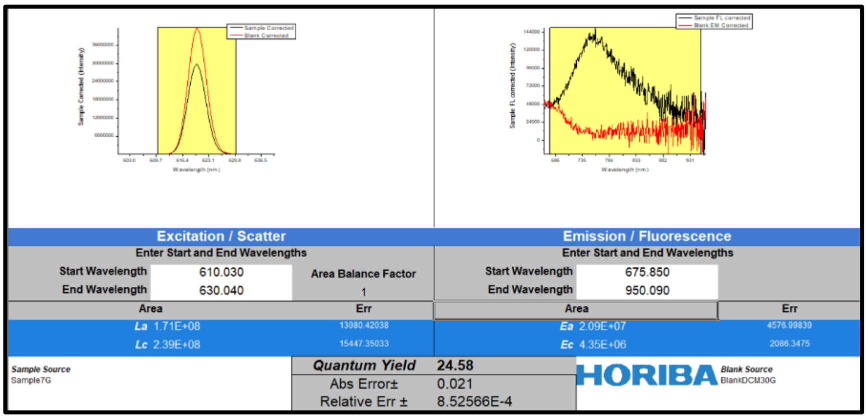

Figure S60. Absolute PLQY measurement of **1H**, **1Py**, and **1Py-BCF**(from top to bottom) in dichloromethane; **1Py-Me**$^{2+}$ is insoluble in dichloromethane.

Table S2. Comparison of photophysical properties in acetonitrile solution.

	$\lambda_{abs,TD\text{-}DFT, AcN}$ [nm]	$\lambda_{abs, ACN}$ (ϵ) [nm] ([10^4 M^{-1} cm^{-1}])	$\lambda_{Fl, ACN}$ [nm][a]	Stokes shift [cm^{-1}]	$\tau_{Fl, ACN}$ [ns][b]	$\Phi_{Fl, ACN}$ [%][c]	k_r[d] / 10^7 s^{-1}	k_{nr}[d] / 10^7 s^{-1}
**1H**	523	547 (2.2)	623	2230	9.8	71.0	7.2	3.0
**1Py**	554	577 (3.1)	669	2383	7.5	75.2	10.0	3.3
**1Py-BCF**	589	609 (4.3)	732	2759	3.2	24.9	7.8	23.5
**1Py-Me^{2+}**	605	619 (3.7)	770	3168	1.8	11.7	6.5	49.1

[a] Excited at absorption maximum. [b] Fluorescence lifetime; excited at 390/670 nm, detected at emission maximum. [c] Absolute photoluminescence quantum yield. [d] Radiative (k_r) and non-radiative (k_{nr}) decay rate constants calculated using the equations $k_r = \Phi / \tau$, $k_{nr} = (1-\Phi) / \tau$.

Table S3. Comparison of photophysical properties in DCM solution.

	$\lambda_{abs,TD\text{-}DFT, DCM}$ [nm]	$\lambda_{abs, DCM}$ (ϵ) [nm] ([10^4 M^{-1} cm^{-1}])	$\lambda_{Fl, DCM}$ [nm][a]	Stokes shift [cm^{-1}]	$\tau_{Fl, DCM}$ [ns][b]	$\Phi_{Fl, DCM}$ [%][c]	k_r[d] / 10^7 s^{-1}	k_{nr}[d] / 10^7 s^{-1}
**1H** [7]	529	560	629	1960	11.1	79.9	7.2	1.8
**1Py**	561	595	676	2014	7.5	77.5	10.3	3.0
**1Py-BCF**	599	638	751	2358	3.1	24.6	7.9	24.3

[a] Excited at absorption maximum. [b] Fluorescence lifetime; excited at 390 nm, detected at emission maximum. [c] Absolute photoluminescence quantum yield. [d] Radiative (k_r) and non-radiative (k_{nr}) decay rate constants calculated using the equations $k_r = \Phi / \tau$, $k_{nr} = (1-\Phi) / \tau$.

Kinetics of Photo-Induced Endoperoxide Formation

Solutions of the BN Lewis pair-functionalized anthracenes (~1×10^{-5} M in 2.5 mL oxygen-saturated acetonitrile) were prepared. The solutions were irradiated by a Xe lamp at room temperature (34 Watt, the distance to the light source was kept constant at 10 cm). A cutoff filter with a wavelength of 455 nm was used to ensure selective excitation at the lowest energy absorption of the BN compounds. The decrease in the acene concentration during irradiation was measured by UV-Vis spectroscopy (short range, fast scan rate) at predetermined time intervals. The wavelengths for recording the absorbances *A* were as follows: **1H** (547 nm), **1Py** (577 nm), **1Py-BCF** (609 nm), and **1Py-Me^{2+}** (619 nm). The corresponding endoperoxides do not absorb at these wavelengths.

The kinetics were determined three times for each substance and the absorbance averaged at each time point. The bimolecular rate constants (k_{app}) were derived using the equation $\ln A = \ln A_0 - k_{app}t$, where a plot of $\ln[A/A_0]$ vs t will be linear with a slope of $-k_{app}$.

Table S4. Comparison of the slope for the plot of $\ln[A/A_0]$ vs t and the pseudo first-order rate constants for the early stages of the reaction of BN Lewis pair-functionalized anthracenes with oxygen upon photoirradiation to give the respective endoperoxides in acetonitrile.

	Slope	k_{app} (10^3 s^{-1})
**1H**	-0.5612	9.353
**1Py**	-0.0598	0.997
**1Py-BCF**	-0.0075	0.125
**1Py-Me^{2+}**	-0.0081	0.135

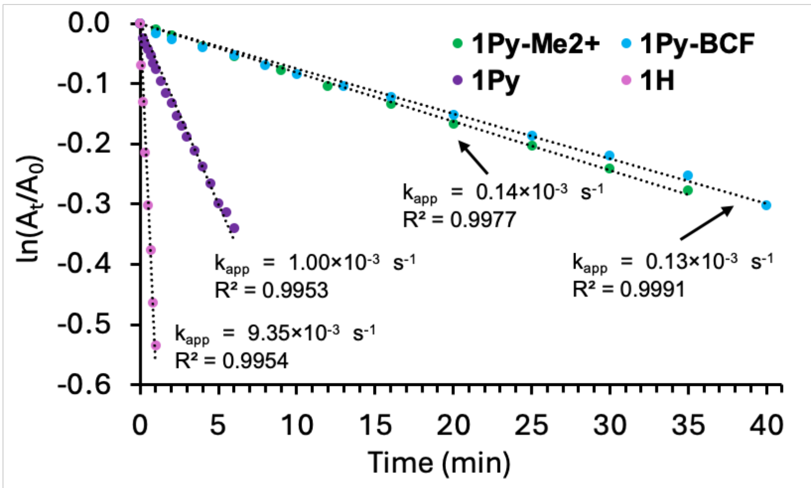

Figure S61. Pseudo first-order kinetics for the early stages of the reaction of BN Lewis pair-functionalized anthracenes with oxygen upon photoirradiation to give the respective endoperoxides in acetonitrile.

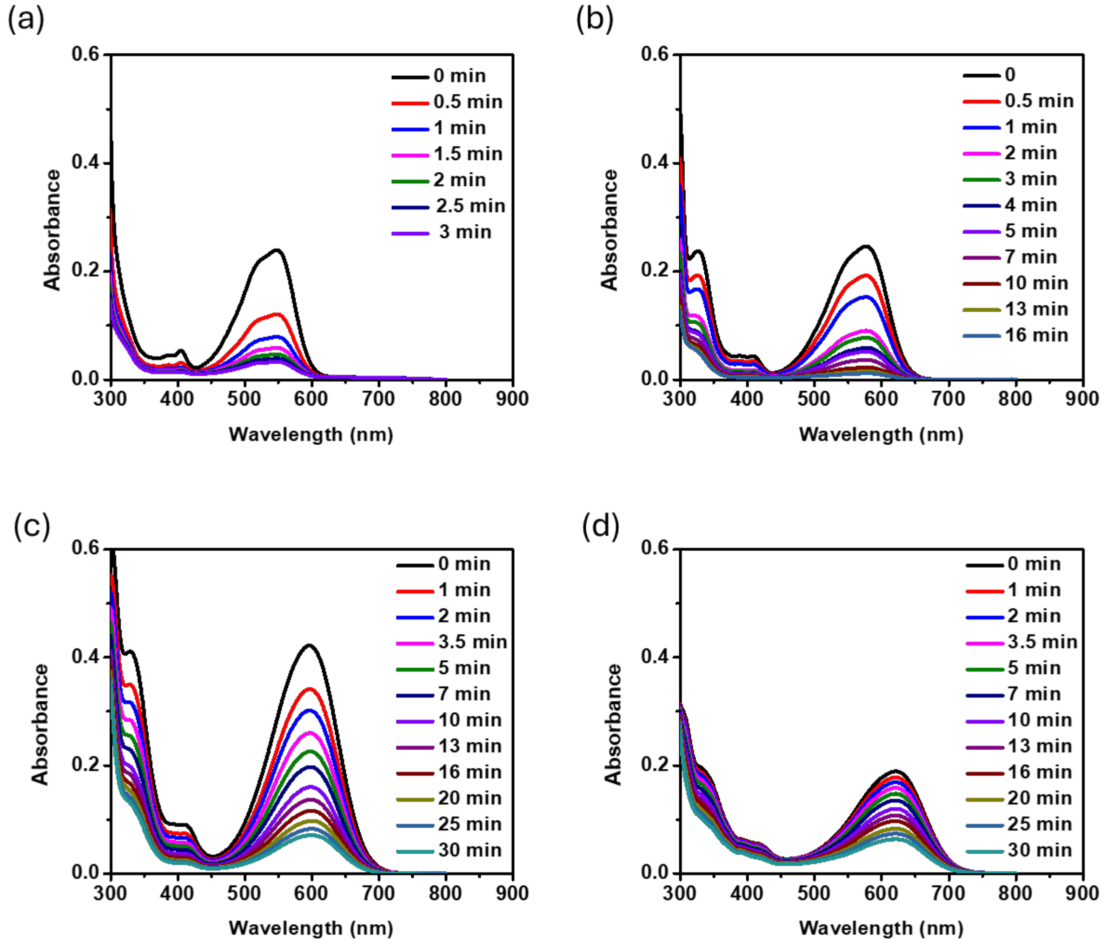

Figure S62. UV-vis spectra of (a) **1H**, (b) **1Py**, (c) **1Py-BCF**, and (d) **1Py-Me^{2+}** at different times for the reaction of BN Lewis pair-functionalized anthracenes (~1×10^{-5} M in oxygen-saturated acetonitrile) with oxygen upon photoirradiation by a Xe lamp (34 Watt) at room temperature to give the respective endoperoxides.

Kinetics of the Endoperoxide Thermolysis

For measurement of thermal release of oxygen (k_{-1} values), solutions of the endoperoxides in anhydrous and deoxygenated dimethylformamide (10 μM anthracene units) were heated to 100 °C. The appearance of the oxygen-free acene species was recorded by UV-vis spectroscopy. The wavelengths for recording the absorbances *A* were as follows: **1H** (554 nm), **1Py** (584 nm). The initial rate constant k_{-1} was determined from the plot of $\ln(A_{end} - A_t)/A_{end}$ versus time.

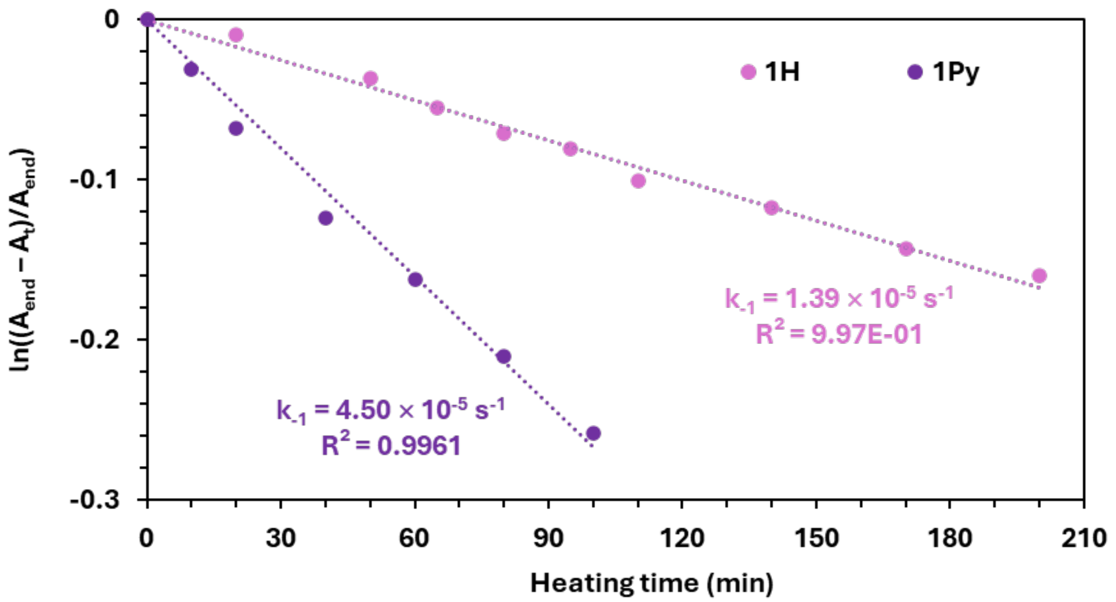

Figure S63. Thermolysis of the endoperoxides in DMF at 100 °C; A_{end}: expected final absorbance of deoxygenated acene based on initial [acene]^0, A_t: absorbance of deoxygenated acene at a given time.

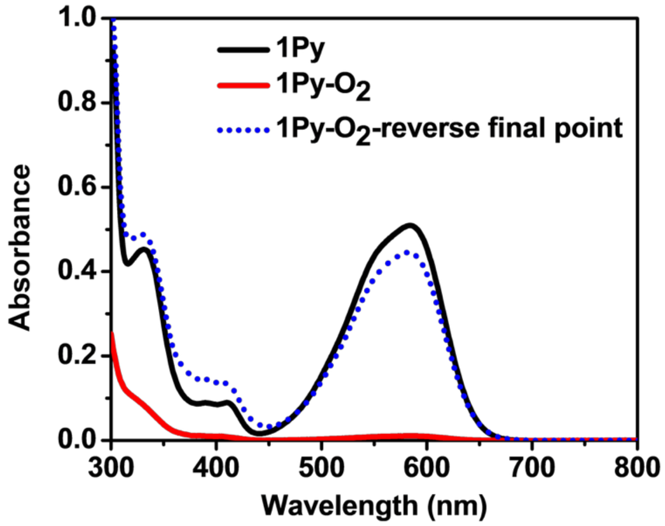

Figure S64. UV-vis spectra of **1Py** in DMF taken before and after thermolysis of the endoperoxides at 100 °C, compared with corresponding deoxygenated acenes prior to endoperoxide formation.

Experiments with 1,3-Diphenylisobenzofuran (DPBF) as Singlet Oxygen Scavenger

Experiments were performed using solutions containing both the respective **BDPA** derivative (10 μM) and 1,3-diphenylisobenzofuran (**DPBF**, 30 μM) as a singlet oxygen scavenger in oxygen-saturated acetonitrile. The solutions were irradiated by a Xe lamp at room temperature (34 Watt, the distance to the light source was kept constant at 10 cm) using a 495 nm cutoff filter to ensure selective excitation of the **BDPA** derivatives (**DPBF** does not absorb above 495 nm). The decrease in the concentration of the respective **BDPA** derivative and **DPBF** during irradiation was followed by UV-Vis spectroscopy at the specified time intervals.

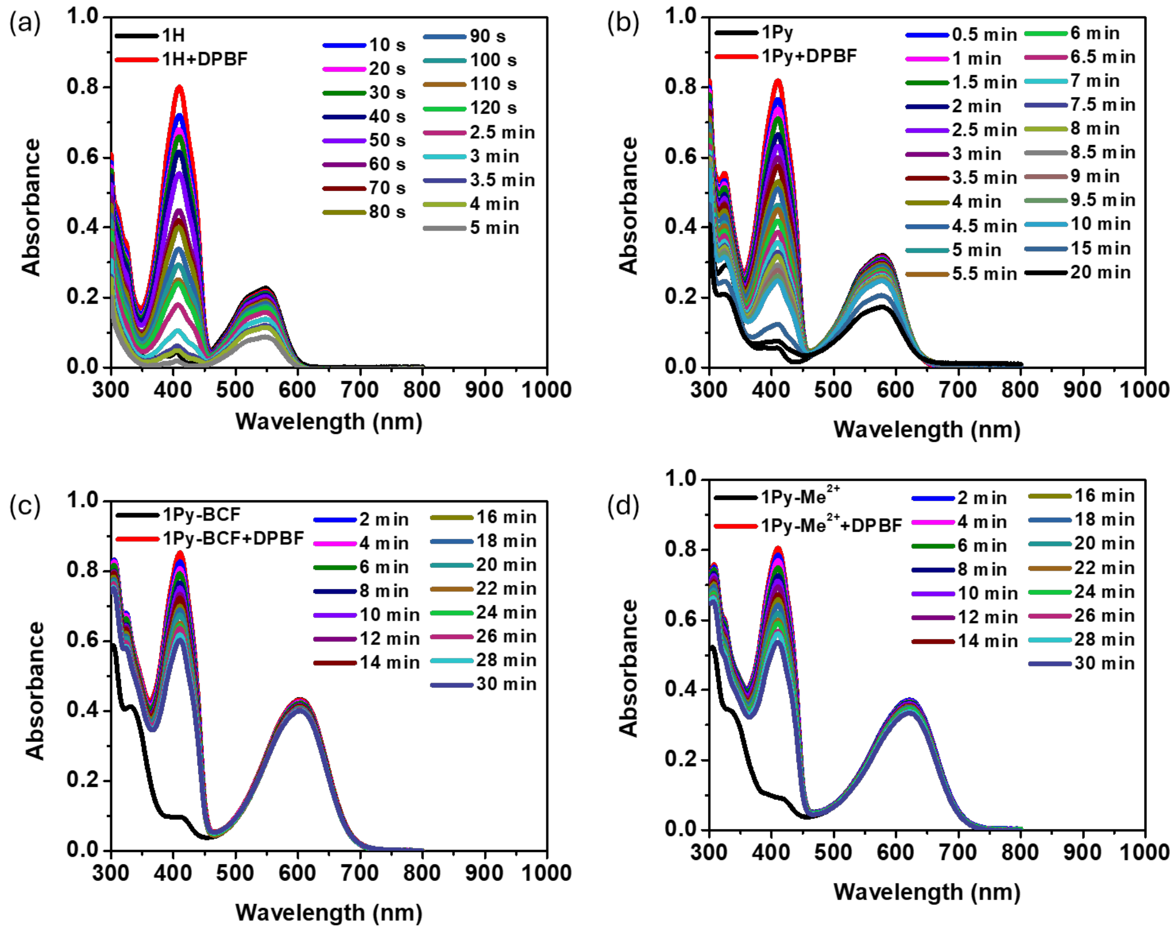

Figure S65. UV-vis spectral data taken at timed intervals upon photoirradiation of (a) **1H**, (b) **1Py**, (c) **1Py-BCF**, (d) **1Py-Me^{2+}** (10 μM in ACN) by a Xe lamp at room temperature (34 Watt, 495 nm cutoff filter) in the presence of 1,3-diphenylisobenzofuran (**DPBF**) (30 μM in ACN) as a singlet oxygen scavenger.

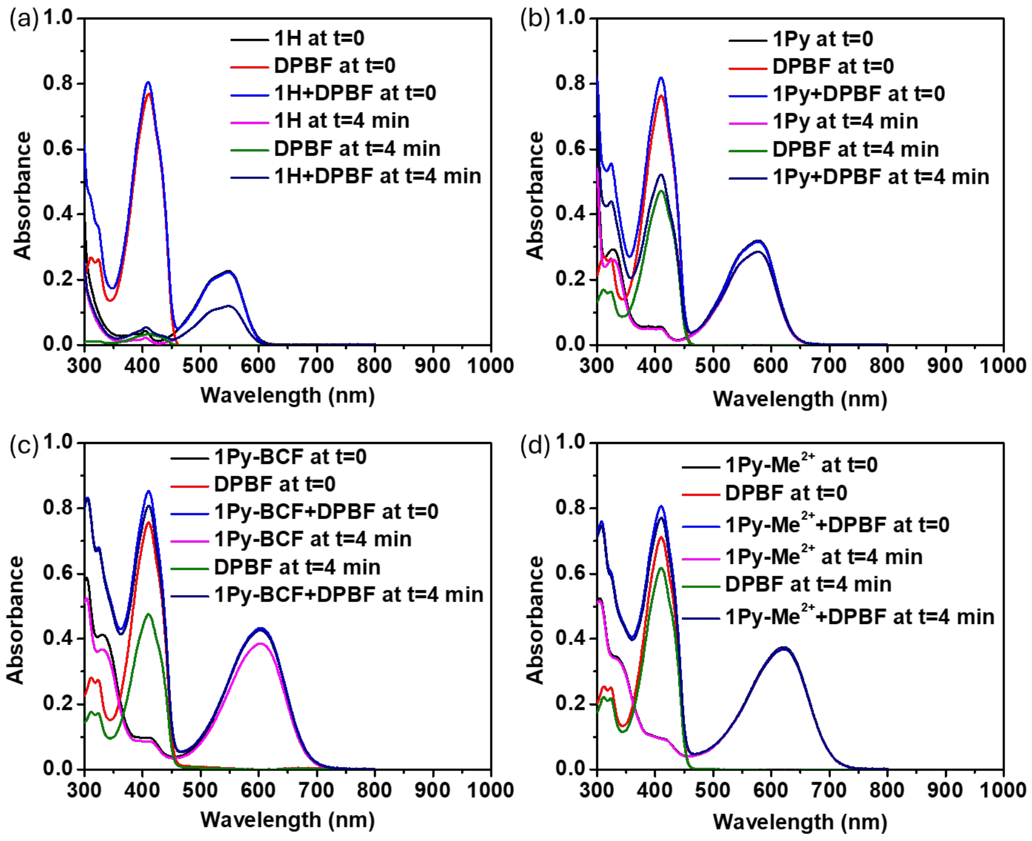

Figure S66. Comparison of UV-vis spectral data before and after photoirradiation of (a) **1H**, (b) **1Py**, (c) **1Py-BCF**, (d) **1Py-Me^{2+}** (10 μM in ACN) by a Xe lamp at room temperature (34 Watt, 4 minutes, 495 nm cutoff filter) in the presence of **DPBF** (30 μM in ACN) as a singlet oxygen scavenger. Spectra for **BDPA** at t=0, **BDPA+DPBF** at t=0 mins, and **BDPA+DPBF** at t=4 mins were acquired. Spectral data for the **BDPA** derivatives at t=4 min are calculated using the original spectra of **BDPA** at t=0 min and scaling them by the relative absorption value at λ_{max} at t=4 min. Spectral data for **DPBF** at t=0 min and of residual amounts of **DPBF** at t=4 mins were deduced by subtracting the absorptions of the BDPA derivatives (scaled based on residual absorption value at λ_{max}) from the spectra of the mixtures.

Table S5. Percentage conversion of **BDPA** derivatives and **DPBF** into endoperoxide cycloaddition products upon 4 mins irradiation.

	Absorbance at λ_{max} for **BDPA** and at 410 nm		Absorbance at λ_{max} (410 nm) for **DPBF**		% conv. **BDPA**	% conv. **DPBF**
	t=0 mins	t=4 mins	t=0 mins	t=4 mins		
**1H**	0.227, 0.036	0.119, 0.019	0.770	0.033	47.6	95.7
**1Py**	0.321, 0.056	0.286, 0.050	0.764	0.472	10.9	38.2
**1Py-BCF**	0.434, 0.097	0.428, 0.095	0.757	0.713	1.4	5.8
**1Py-Me^{2+}**	0.373, 0.094	0.367, 0.091	0.714	0.620	1.6	13.2

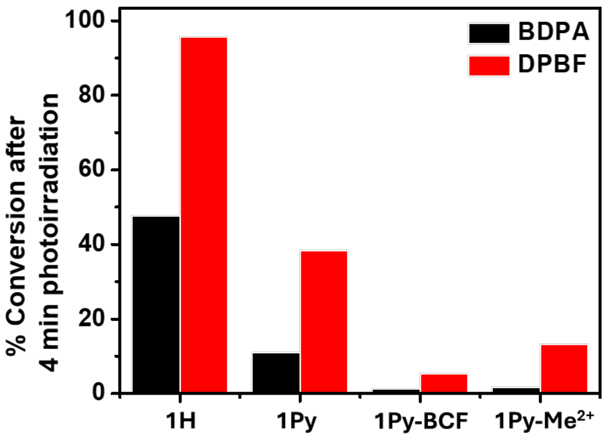

Figure S67. Illustration of % oxidation of **BDPA** and **DPDF** derived from UV-vis spectral data taken before and after photoirradiation of **1H**, **1Py**, **1Py-BCF**, and **1Py-Me^{2+}** (10 μM in ACN) by a Xe lamp for 4 minutes at room temperature (34 Watt, 495 nm cutoff filter) in the presence of **DPBF** (30 μM in ACN) as a singlet oxygen scavenger.

Electrochemical Studies

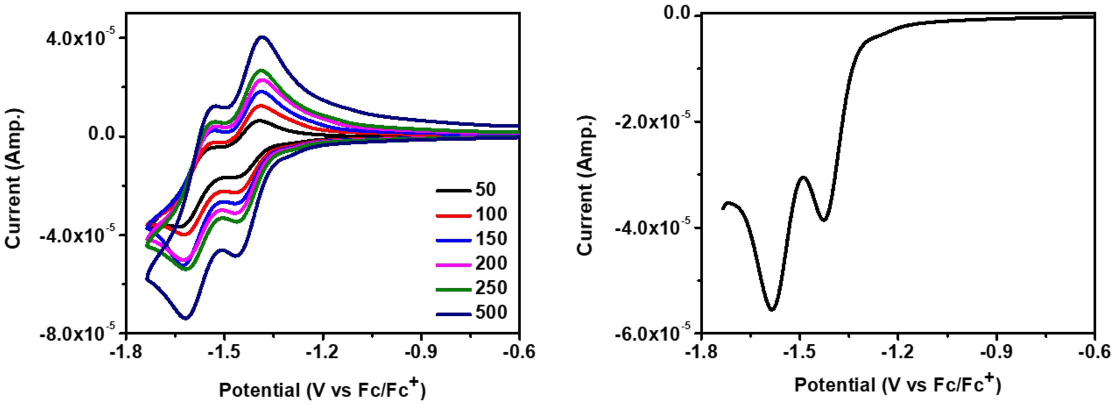

Figure S68. (a) Cyclic voltammetry data of **1Py** in acetonitrile at different scan rates ranging from 50 – 500 mV/s. (b) Square wave voltammogram of **1Py** in acetonitrile (0.1 M $Bu_4N[PF_6]$; referenced to Fc/Fc^+ couple).

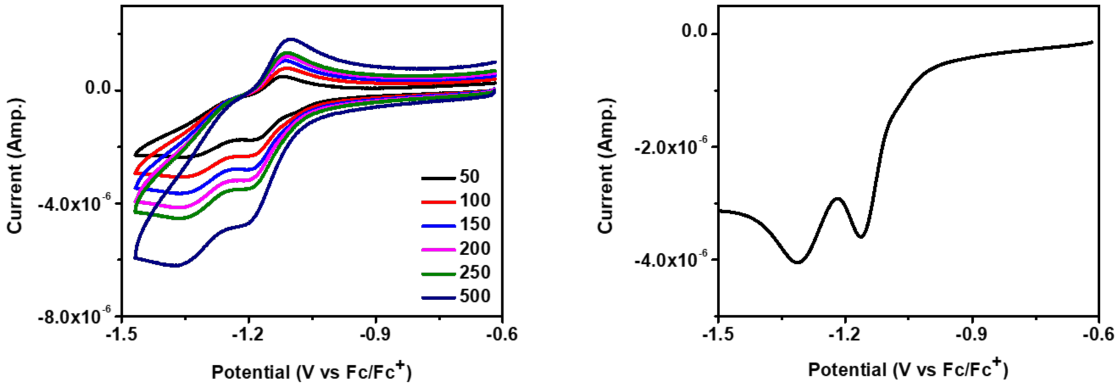

Figure S69. (a) Cyclic voltammetry data of **1Py-BCF** in acetonitrile at different scan rates ranging from 50 – 500 mV/s. (b) Square wave voltammogram of **1Py-BCF** in acetonitrile (0.1 M $Bu_4N[PF_6]$; referenced to Fc/Fc^+ couple).

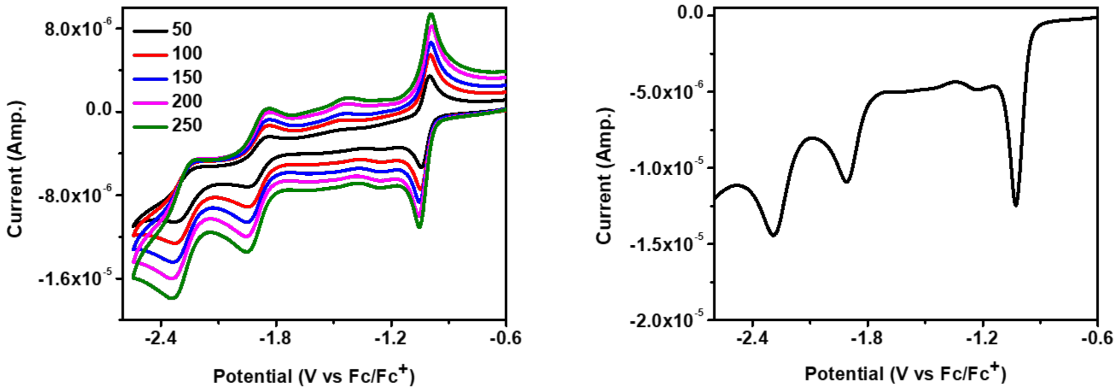

Figure S70. (a) Cyclic voltammetry data of **1Py-Me^{2+}** in acetonitrile at different scan rates ranging from 50 – 500 mV/s. (b) Square wave voltammogram of **1Py-Me^{2+}** in acetonitrile (0.1 M $Bu_4N[PF_6]$; referenced to Fc/Fc^+ couple).

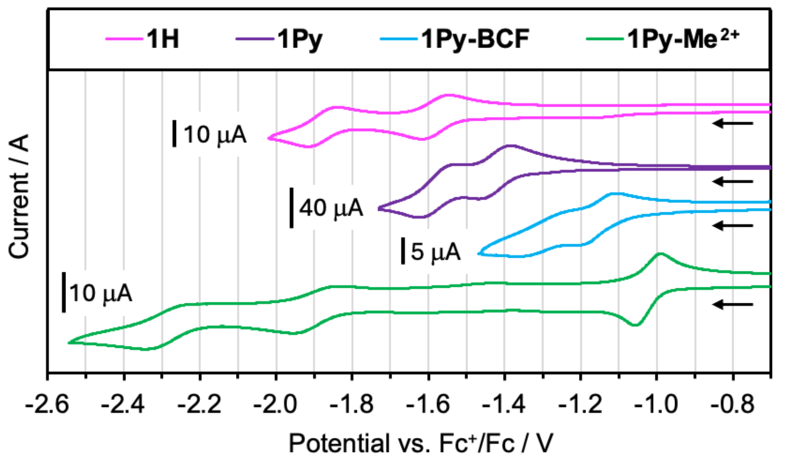

Figure S71. Comparison of cyclic voltammograms of **1Py**, **1Py-BCF**, and **1Py-Me^{2+}** in acetonitrile at a scan rate of 200 mV/s (0.1 M $Bu_4N[PF_6]$; referenced to Fc/Fc$^+$ couple).

Table S6. Comparison of estimated energy levels and band gaps for **1Py**, **1Py-BCF**, and **1Py-Me^{2+}** from electrochemical and UV-visible data with computational results.

Compound	$E_{red1,2,3}$ [V] [a]	$\Delta E_{red1\text{-}2}$ [V] [a]	E_{LUMO} [eV] [b]	$E^{DFT}{}_{LUMO}$ [eV] [c]	$E^{DFT}{}_{HOMO}$ [eV] [c]	ΔE^{DFT} [eV] [c]	$\Delta E_{exp,}{}^{UV}$ [eV] [d]
**1H**	-1.58, -1.88	0.30	-3.22	-2.61	-5.01	2.40	1.99
**1Py**	-1.43, -1.58	0.15	-3.37	-2.81	-5.06	2.25	1.85
**1Py-BCF**	-1.16, -1.32	0.16	-3.65	-3.14	-5.18	2.04	1.69
**1Py-Me^{2+}**	-1.02, -1.90, -2.29	n.o., 0.39 ($\Delta E_{red2\text{-}3}$)	-3.78	-3.29	-5.20	1.91	1.61

[a] Derived from CV data at 200 mV/s in acetonitrile relative to Fc/Fc$^+$ couple, $E_{red} = 0.5\ (E_{pc} + E_{pa})$. [b] $E_{LUMO} = -(4.8 + E_{red})$. [c] From DFT calculations at B3LYP/6-31G(d,p) (SCRF=acetonitrile) level of theory. [d] Estimated from absorption maxima in acetonitrile.

Spectroelectrochemistry Studies

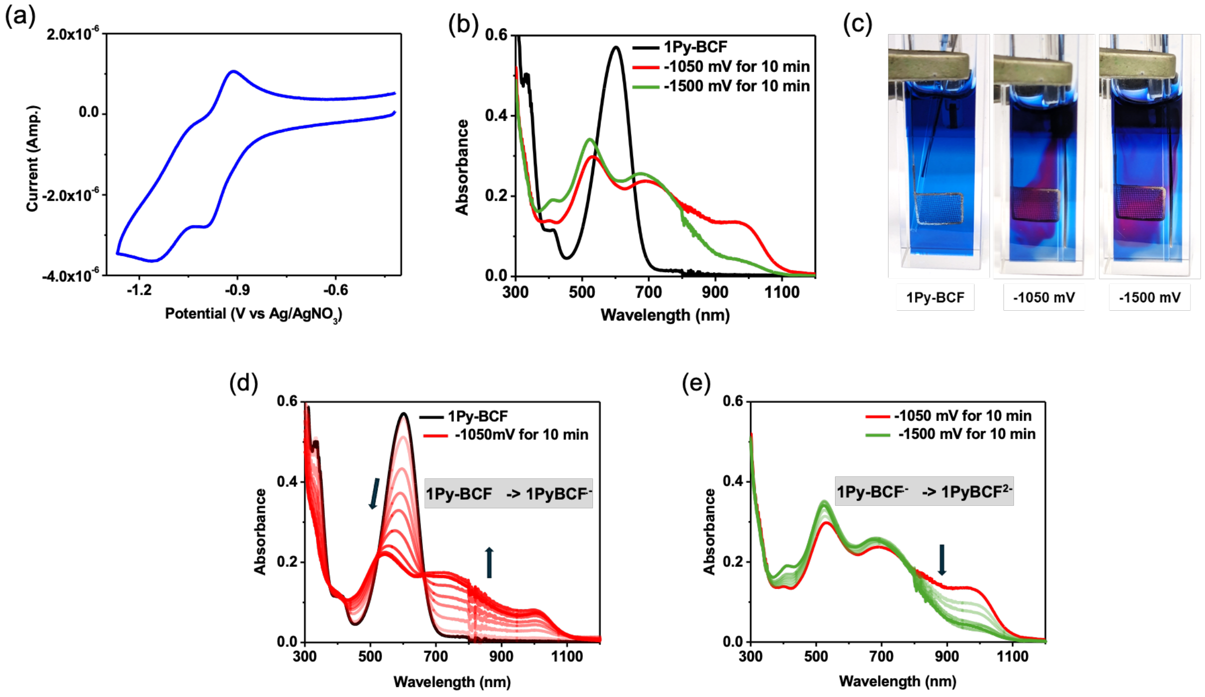

Figure S72. (a) Cyclic voltammogram of **1Py-BCF** in acetonitrile containing 0.1 M $Bu_4N[PF_6]$ (scan rate 200 mV/s). Electrochemical measurements were performed using an Au working electrode, a Pt wire counter electrode, and an $Ag/AgNO_3$ reference electrode. (b) Spectroelectrochemical data: absorption spectra of **1Py-BCF** in acetonitrile upon applying -1050 mV and -1500 mV for 10 min each. (c) Photographs of acetonitrile solution of **1Py-BCF** upon applying -1050 mV and -1500 mV. (d,e) Spectroelectrochemical data: Monitoring the absorption spectra of **1Py-BCF** in acetonitrile (with 0.1 M $Bu_4N[PF_6]$) upon applying a voltage of -1050 mV over the period of 10 min (d) and -1500 mV over the period of 10 min (e). Reference electrode: $Ag/AgNO_3$.

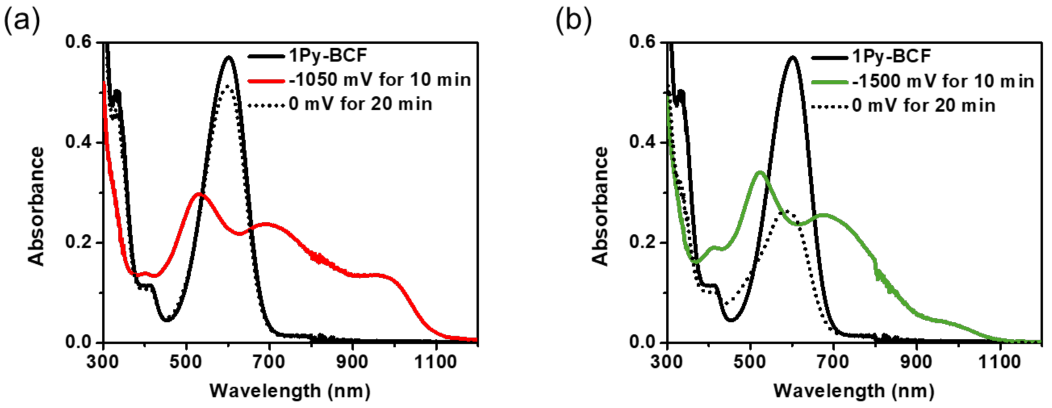

Figure S73. Spectroelectrochemical data, reversibility check: absorption spectra of **1Py-BCF** in acetonitrile (with 0.1 M $Bu_4N[PF_6]$) upon applying a voltage of 0 mV for 20 min to the solutions of the reduced species obtained by applying a voltage of (a) -1050 mV and (b) -1500 mV. Reference electrode: $Ag/AgNO_3$.

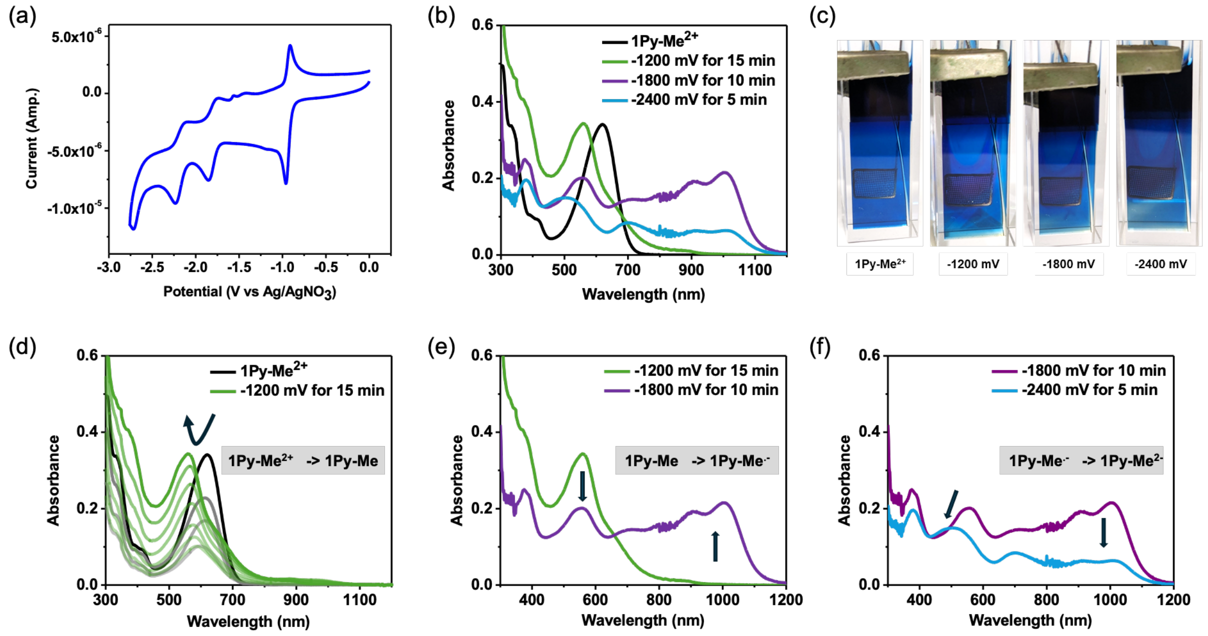

Figure S74. (a) Cyclic voltammogram of **1Py-Me^{2+}** in acetonitrile containing 0.1 M $Bu_4N[PF_6]$ (scan rate 200 mV/s). Electrochemical measurements were performed using an Au working electrode, a Pt wire counter electrode, and an $Ag/AgNO_3$ reference electrode. (b) Spectroelectrochemical data: absorption spectra of **1Py-Me^{2+}** in acetonitrile upon applying -1200 mV, -1800 mV and -2400 mV. (c) Photographs of acetonitrile solution of **1Py-Me^{2+}** upon applying -1200 mV, -1800 mV and -2400 mV. (d) Spectroelectrochemical data: monitoring the absorption spectra of **1Py-Me^{2+}** in acetonitrile (with 0.1 M $Bu_4N[PF_6]$) upon applying a voltage of -1050 mV over the period of 15 min. (e,f) Spectroelectrochemical data: absorption spectra of **1Py-Me^{2+}** in acetonitrile upon applying -1800 mV and -2400 mV. Reference electrode: $Ag/AgNO_3$.

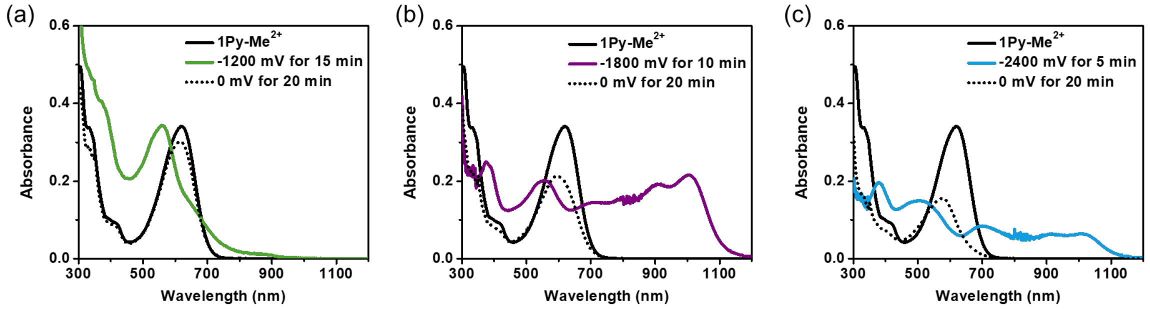

Figure S75. Spectroelectrochemical data, reversibility check: absorption spectra of **1Py-Me^{2+}** in acetonitrile (with 0.1 M $Bu_4N[PF_6]$) upon applying a voltage of 0 mV for 20 min to solutions of the reduced species obtained by applying a voltage of (a) -1200 mV, (b) -1800 mV and (b) -2400 mV. Reference electrode: $Ag/AgNO_3$.

Computational Studies

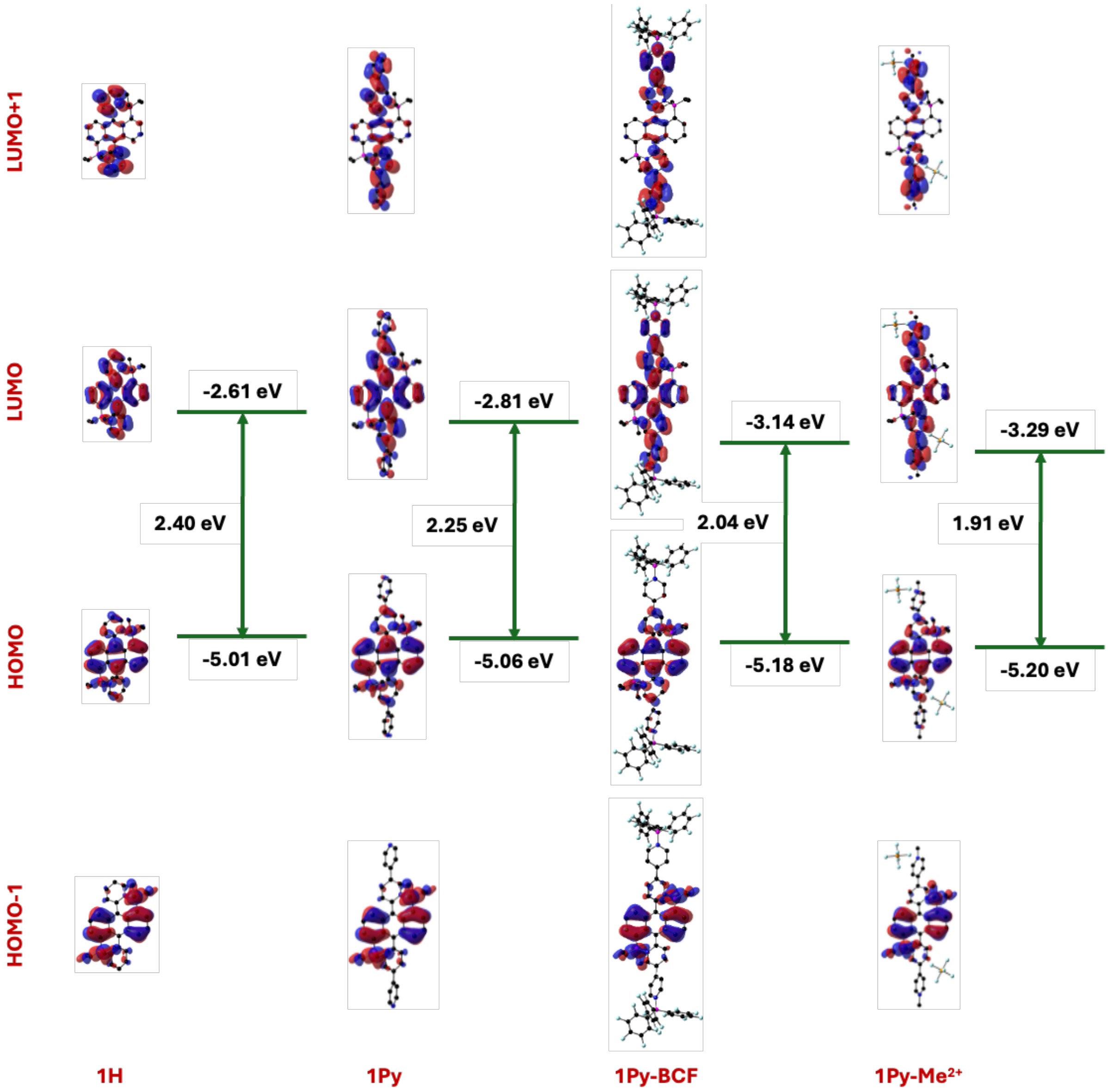

Figure S76. Frontier molecular orbital diagrams of **1H**, **1Py**, **1Py-BCF**, and **1Py-Me^{2+}** along with the HOMO and LUMO energies obtained from calculations at the B3LYP/6-31G(d,p) level of theory (SCRF=acetonitrile).

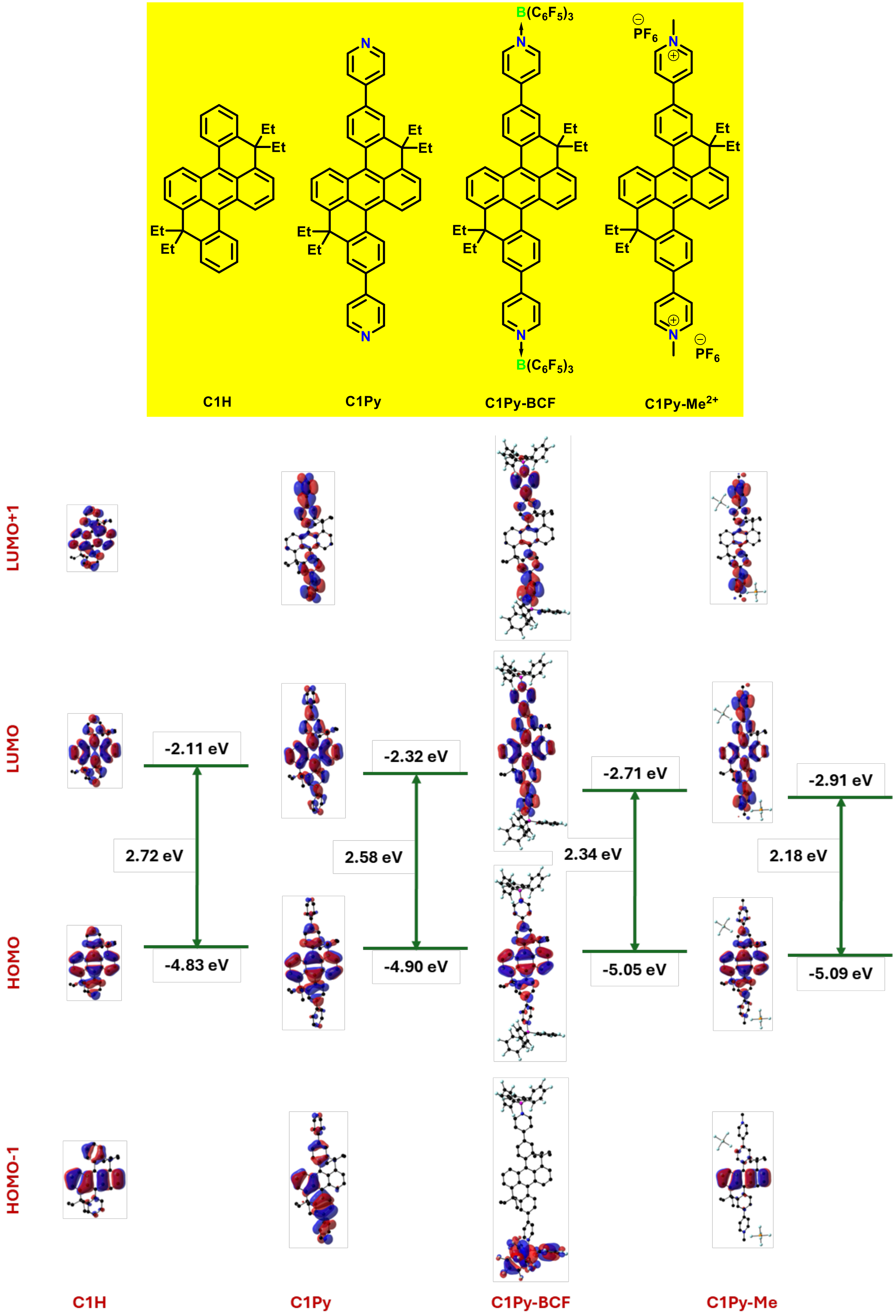

Figure S77. Frontier molecular orbital diagrams of all-carbon analogues **C1H**, **C1Py**, **C1Py-BCF**, and **C1Py-Me^{2+}** along with the HOMO and LUMO energies obtained from calculations at the B3LYP/6-31G(d,p) level of theory (SCRF=acetonitrile).

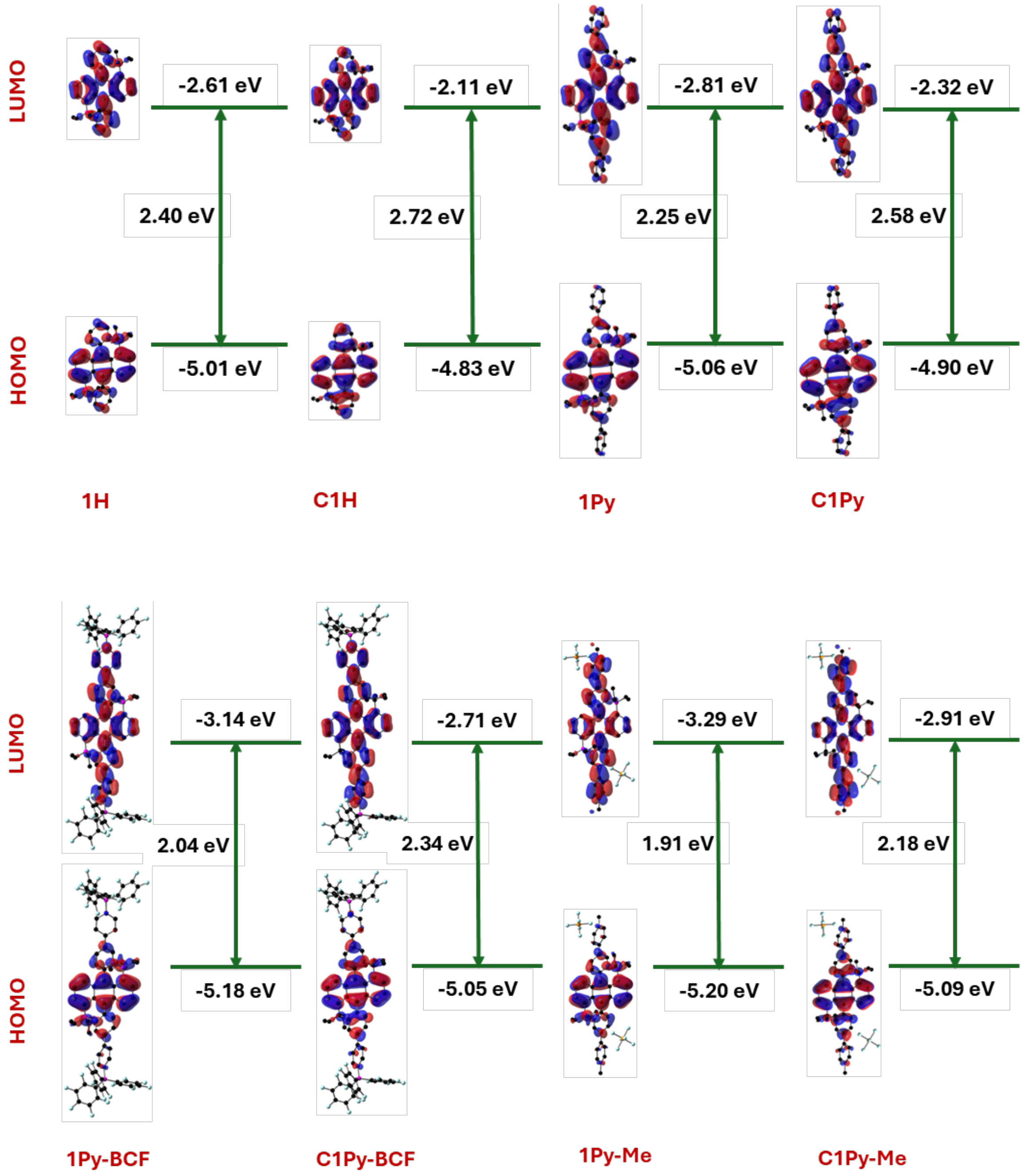

Figure S78. Comparison of frontier molecular orbital diagrams (HOMO and LUMO) of **1H**, **1Py**, **1Py-BCF**, and **1Py-Me^{2+}** with their corresponding all-carbon analogues **C1H**, **C1Py**, **C1Py-BCF**, and **C1Py-Me^{2+}** along with the orbital energies obtained from calculations at the B3LYP/6-31G(d,p) level of theory (SCRF=acetonitrile).

Table S7. Geometric parameters (distances in Å, angles in °) for **1Py-BCF** and **1Py-Me** in different redox states obtained from DFT calculations at the R/U-B3LYP/6-31G(d,p) (SCRF=acetonitrile) level.

	$B\text{-}N_{PyB}$	$B\text{-}N_{Pyt}$	$B\text{-}C_{An}$	$C_{An}\text{-}C_{PyB}$	$C_{PyB}\text{-}C_{Pyt}$	α[a]	β[b]	γ[c]	δ[d]	ε[e]	ϕ[f]
**1Py-BCF**	1.645	**1.651**	1.614	**1.466**	**1.474**	106.0	**171.3**	165.3	**22.2**	**31.9**	32.9
	1.648	**1.651**	1.615	**1.466**	**1.475**	106.3	**174.0**	166.5		**32.1**	35.1
**1Py-BCF$^{-\cdot}$**	1.637	1.623	1.616	1.432	1.456	105.9	173.5	164.6	25.8	17.2	30.4
	1.640	1.623	1.618	1.433	1.457	106.7	176.9	166.3		18.2	34.4
**1Py-BCF^{2-}**	1.634	1.605	1.619	1.400	1.435	106.0	176.1	163.9	30.2	6.0	28.7
	1.638	1.605	1.620	1.401	1.436	107.3	179.4	166.2		8.7	34.1
**1Py-Me^{2+}** [g]	1.643		1.615	**1.467**	**1.473**	105.4	**171.1**	165.3	**22.2**	**28.1**	32.1
	1.645		1.618	**1.468**	**1.473**	106.3	**173.9**	166.9		**30.6**	37.6
**1Py-Me$^{+\cdot}$**	1.638		1.616	1.434	1.450	105.6	173.3	164.8	25.7	13.9	30.5
	1.642		1.618	1.435	1.450	106.6	176.5	166.4		15.4	34.0
**1Py-Me^{0}**	1.636		1.617	1.402	1.427	106.0	176.0	164.0	30.1	4.8	29.2
	1.640		1.618	1.402	1.428	107.4	179.2	165.0		6.7	34.0
**1Py-Me$^{-\cdot}$**	1.631		1.622	1.424	1.427	106.4	173.9	165.2	26.8	3.0	28.5
	1.636		1.622	1.424	1.428	107.5	177.7	167.3		3.8	28.5
**1Py-Me^{2-}** [h]	1.629		1.622	1.440	1.409	107.0	172.3	166.5	25.1	3.0	27.5
	1.633		1.624	1.440	1.410	108.3	176.8	168.9		4.2	32.6

[a] α = C_{An}-B-N angle; [b] β = Cent1-C_{An}-B; [c] γ = Cent2-C_{An}-C_{Py}; [d] δ = $Ph_{An,out}$ // $Ph_{An,out}$, dihedral angle between outer anthracene rings; [e] ε = Py_B // Py_t , dihedral angle between pyridyl rings; [f] ϕ = $Ph_{An,in}$ // Py_B, interplanar angle between inner anthracene ring and pendent pyridyl ring; [g] counter anions included in computations; [h] closed shell configuration.

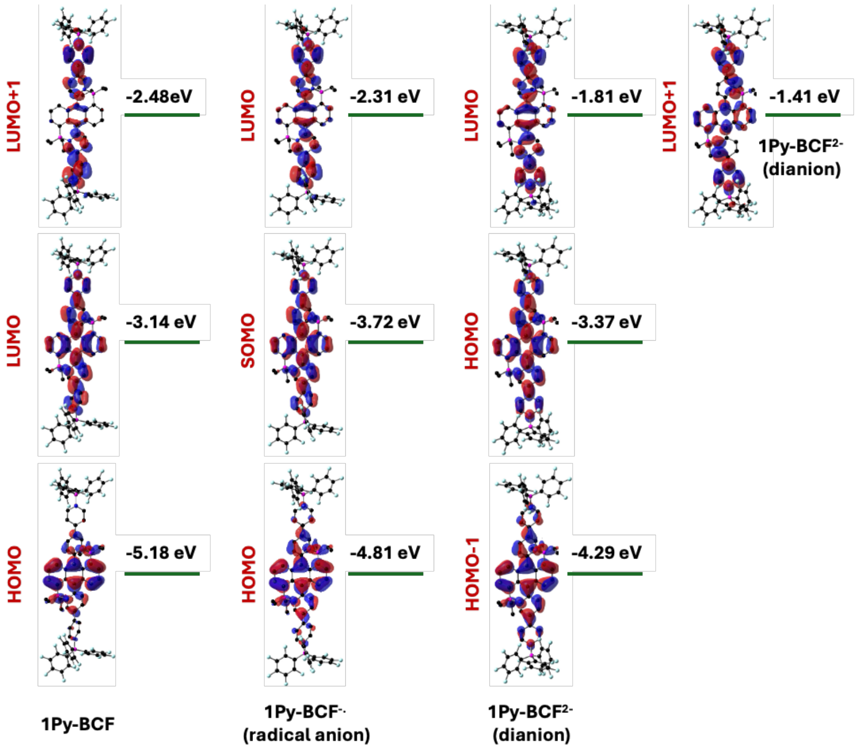

Figure S79. Frontier molecular orbital diagrams (iso=0.02) for different redox states of **1Py-BCF**. Calculations were performed at the R/U-B3LYP/6-31G(d,p) (SCRF=acetonitrile) level.

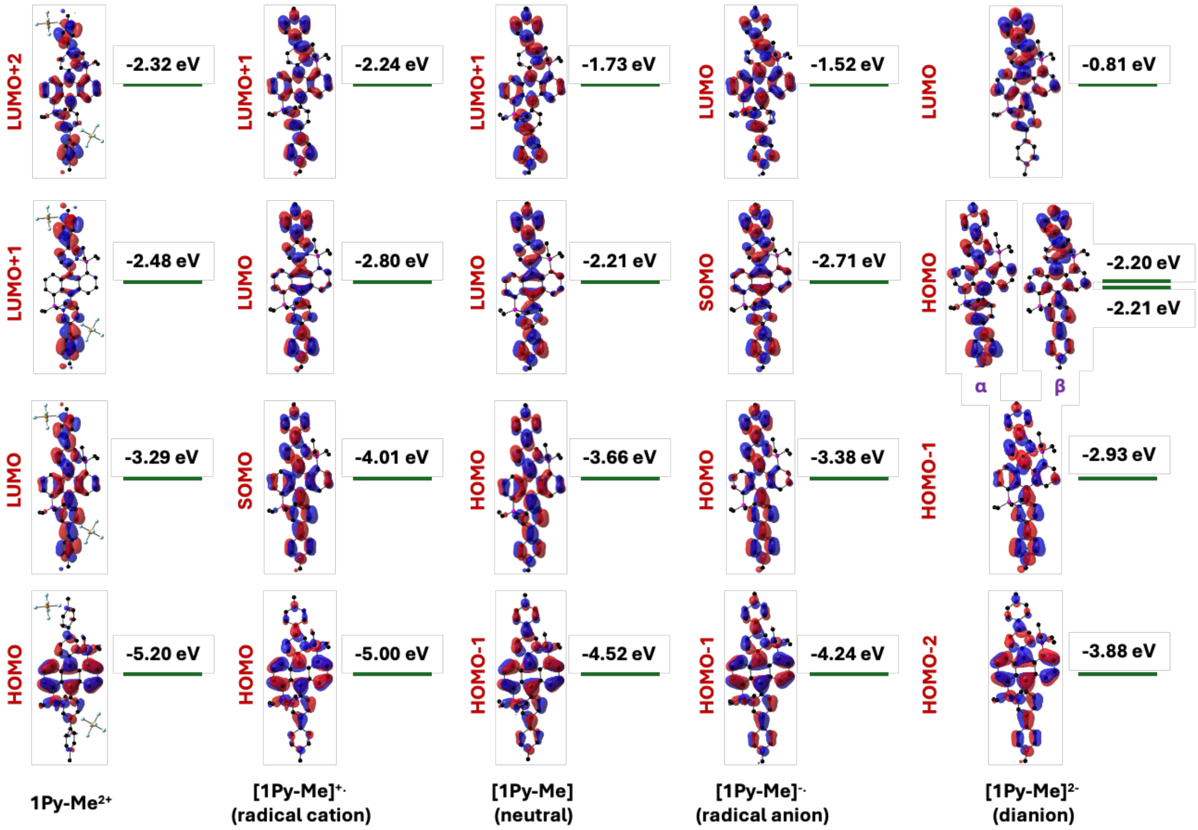

Figure S80. Frontier molecular orbital diagrams (iso=0.02) for different redox states of **1Py-Me**. Calculations were performed at the R/U-B3LYP/6-31G(d,p) (SCRF=acetonitrile) level; dianion in closed shell configuration.

TD-DFT Computations of Parent Compounds at B3LYP/6-31G(d,p) // B3LYP/6-31G(d,p) (SCRF=acetonitrile) Level of Theory

Table S8. TD-DFT results for **1H** at the B3LYP/6-31G(d,p) // B3LYP/6-31G(d,p) (SCRF=acetonitrile) level of theory.

Transition[a]	E_{exc}, eV	λ, nm	Oscillator Strength, *f*	Assignment (coefficients, *c*)[b]
**$S_0 \rightarrow S_1$**	**2.03**	**610.5**	**0.334**	**H → L = 0.70**
$S_0 \rightarrow S_5$	3.29	377.4	0.064	H-2 → L = 0.64<br>H → L+2 = 0.14<br>H → L+5 = 0.23
$S_0 \rightarrow S_6$	3.64	340.7	0.074	H-3 → L = 0.69

[a] Only transitions with oscillator strengths >0.05 are presented. [b] % contribution = 2 c^2 * 100%, H = HOMO, L = LUMO).

Table S9. TD-DFT results for **1Py** at the B3LYP/6-31G(d,p) // B3LYP/6-31G(d,p) (SCRF=acetonitrile) level of theory.

Transition[a]	E_{exc}, eV	λ, nm	Oscillator Strength, *f*	Assignment (coefficients, *c*)[b]
**$S_0 \rightarrow S_1$**	**1.88**	**660.8**	**0.528**	**H → L = 0.71**
$S_0 \rightarrow S_6$	3.18	389.7	0.071	H-2 → L = 0.67<br>H → L+7 = 0.18<br>H → L+8 = 0.10
**$S_0 \rightarrow S_8$**	**3.47**	**357.4**	**0.298**	**H-3 → L = 0.68**<br>**H → L+4 = 0.16**
$S_0 \rightarrow S_{10}$	3.69	336.4	0.067	H-10 → L = 0.11<br>H-8 → L = -0.12<br>H-7 → L = 0.21<br>H-6 → L = -0.18<br>H-5 → L = -0.21<br>H-4 → L = 0.57

[a] Only transitions with oscillator strengths >0.05 are presented. [b] % contribution = 2 c^2 * 100%, H = HOMO, L = LUMO).

Table S10. TD-DFT results for **1Py-BCF** at the B3LYP/6-31G(d,p) // B3LYP/6-31G(d,p) (SCRF=acetonitrile) level of theory.

Transition[a]	E_{exc}, eV	λ, nm	Oscillator Strength, *f*	Assignment (coefficients, *c*)[b]
**$S_0 \rightarrow S_1$**	**1.68**	**738.6**	**0.658**	**H → L = 0.71**
$S_0 \rightarrow S_6$	3.01	412.6	0.069	H-4 → L = 0.68<br>H → L+13 = 0.14
**$S_0 \rightarrow S_{10}$**	**3.28**	**378.5**	**0.253**	H-13 → L = -0.15<br>**H-11 → L = 0.49**<br>H-7 → L = 0.14<br>H-6 → L = 0.14<br>**H-5 → L = -0.33**<br>H-1 → L+1 = -0.14<br>H → L+4 = -0.21

[a] Only transitions with oscillator strengths >0.05 are presented. [b] % contribution = 2 c^2 * 100%, H = HOMO, L = LUMO).

Table S11. TD-DFT results for **1Py-Me^{2+}** at the B3LYP/6-31G(d,p) // B3LYP/6-31G(d,p) (SCRF=acetonitrile) level of theory.

Transition[a]	E_{exc}, eV	λ, nm	Oscillator Strength, *f*	Assignment (coefficients, *c*)[b]
**$S_0 \rightarrow S_1$**	**1.57**	**789.7**	**0.599**	**H → L = 0.71**
$S_0 \rightarrow S_6$	2.90	427.6	0.062	H-2 → L = 0.69<br>H → L+9 = 0.12
$S_0 \rightarrow S_7$	3.07	403.4	0.056	H-3 → L = -0.34<br>H-1 → L+1 = -0.20<br>H → L+4 = 0.53<br>H-2 → L+6 = -0.23
$S_0 \rightarrow S_8$	3.14	394.6	0.067	H-3 → L = 0.35<br>H-1 → L+1 = 0.48<br>H → L+4 = 0.36<br>H-2 → L+6 = -0.10
$S_0 \rightarrow S_9$	3.23	383.9	0.111	H-3 → L = 0.50<br>H-1 → L+1 = -0.47<br>H → L+4 = 0.14

[a] Only transitions with oscillator strengths >0.05 are presented. [b] % contribution = 2 c^2 * 100%, H = HOMO, L = LUMO).

TD-DFT Computations of Parent Compounds and all-Carbon Analogues

Table S12a. TD-DFT results for **1H** at the B3LYP/6-31G(d,p) // cam-B3LYP/6-31G(d,p) (SCRF=acetonitrile) level of theory.

Transition[a]	E_{exc}, eV	λ, nm	Oscillator Strength, *f*	Assignment (coefficients, *c*)[b]
**$S_0 \rightarrow S_1$**	**2.37**	**522.8**	**0.443**	**H → L = 0.70**
$S_0 \rightarrow S_7$	4.43	280.2	0.162	H-7 → L = -0.17<br>H-6 → L = -0.14<br>H-3 → L = 0.59<br>H-1 → L+1 = -0.12<br>H → L+2 = 0.15<br>H → L+3 = 0.17
$S_0 \rightarrow S_{10}$	4.61	268.9	0.261	H-6 → L = 0.39<br>H-4 → L = -0.20<br>H-3 → L = -0.16<br>H-2 → L = -0.21<br>H-1 → L+1 = 0.11<br>H → L+5 = 0.41

[a] Only transitions with oscillator strengths >0.1 are presented. [b] % contribution = 2 c^2 * 100%, H = HOMO, L = LUMO).

Table S12b. TD-DFT results for **C1H** at the B3LYP/6-31G(d,p) // cam-B3LYP/6-31G(d,p) (SCRF=acetonitrile) level of theory.

Transition[a]	E_{exc}, eV	λ, nm	Oscillator Strength, *f*	Assignment (coefficients, *c*)[b]
**$S_0 \rightarrow S_1$**	**2.68**	**462.9**	**0.514**	**H → L = 0.70**
$S_0 \rightarrow S_3$	4.38	282.8	0.135	H → L+1 = 0.42<br>H → L+2 = 0.50<br>H-1 → L = –0.18<br>H-3 → L = –0.11
$S_0 \rightarrow S_5$	4.57	271.1	0.246	H-5 → L = –0.26<br>H-3 → L = –0.26<br>H-2 → L = –0.26<br>H-1 → L = 0.34<br>H → L+1 = –0.22<br>H → L+2 = 0.26<br>H → L+4 = –0.15
**$S_0 \rightarrow S_9$**	**4.79**	**259.0**	**0.398**	**H-5 → L = 0.56**<br>**H-1 → L = 0.23**<br>H → L+1 = –0.17<br>H → L+2 = 0.2<br>H → L+5 = –0.12

[a] Only transitions with oscillator strengths >0.12 are presented. [b] % contribution = 2 c^2 * 100%, H = HOMO, L = LUMO).

Table S13a. TD-DFT results for **1Py** at the B3LYP/6-31G(d,p) // cam-B3LYP/6-31G(d,p) (SCRF=acetonitrile) level of theory.

Transition[a]	E_{exc}, eV	λ, nm	Oscillator Strength, *f*	Assignment (coefficients, *c*)[b]
**$S_0 \rightarrow S_1$**	**2.24**	**554.0**	**0.665**	**H → L = 0.70**
$S_0 \rightarrow S_2$	3.52	352.4	0.077	H-2 → L = 0.61<br>H-2 → L+4 = 0.12<br>H-1 → L = 0.10<br>H → L+5 = 0.27
$S_0 \rightarrow S_6$	4.06	305.3	0.222	H-3 → L = -0.25<br>H → L+2 = 0.43<br>H → L+3 = 0.39<br>H → L+8 = 0.21
$S_0 \rightarrow S_8$	4.25	291.6	0.355	H-5 → L = -0.10<br>H-3 → L = 0.56<br>H → L+4 = 0.33

[a] Only transitions with oscillator strengths >0.05 are presented. [b] % contribution = 2 c^2 * 100%, H = HOMO, L = LUMO).

Table S13b. TD-DFT results for **C1Py** at the B3LYP/6-31G(d,p) // cam-B3LYP/6-31G(d,p) (SCRF=acetonitrile) level of theory.

Transition[a]	E_{exc}, eV	λ, nm	Oscillator Strength, *f*	Assignment (coefficients, *c*)[b]
**$S_0 \rightarrow S_1$**	**2.56**	**484.9**	**0.852**	**H → L = 0.70**
**$S_0 \rightarrow S_3$**	**4.07**	**304.6**	**0.255**	**H → L+2 = 0.58**<br>H → L+7 = 0.12<br>H → L+9 = –0.19<br>H-3 → L = –0.15
$S_0 \rightarrow S_6$	4.44	279.4	0.177	H-4 → L = 0.38<br>H-3 → L = –0.31<br>H-2 → L = 0.15<br>H-1 → L = –0.30<br>H → L+3 = –0.22<br>H → L+6 = 0.12
**$S_0 \rightarrow S_{10}$**	**4.67**	**265.6**	**0.587**	H-6 → L = 0.10<br>**H-5 → L = –0.31**<br>H-4 → L+1 = 0.10<br>**H-3 → L = –0.35**<br>H-2 → L = –0.22<br>H-1 → L = 0.14<br>**H → L+3 = 0.36**<br>H → L+5 = 0.12<br>H → L+7 = –0.12

[a] Only transitions with oscillator strengths >0.1 are presented. [b] % contribution = 2 c^2 * 100%, H = HOMO, L = LUMO).

Table S14a. TD-DFT results for **1Py-BCF** at the B3LYP/6-31G(d,p) // cam-B3LYP/6-31G(d,p) (SCRF=acetonitrile) level of theory.

Transition[a]	E_{exc}, eV	λ, nm	Oscillator Strength, *f*	Assignment (coefficients, *c*)[b]
**$S_0 \rightarrow S_1$**	**2.11**	**588.5**	**0.806**	**H → L = 0.69**<br>H → L+2 = -0.12
$S_0 \rightarrow S_5$	3.68	337.0	0.113	H → L+2 = 0.64<br>H → L+9 = 0.16
$S_0 \rightarrow S_7$	3.94	315.0	0.211	H–18 → L = 0.11<br>H–13 → L = –0.13<br>H–11 → L = 0.27<br>H → L+4 = 0.56<br>H → L+6 = 0.15
$S_0 \rightarrow S_8$	4.14	299.4	0.519	H–18 → L = 0.15<br>H–13 → L = –0.23<br>H–11 → L = 0.47<br>H–1 → L+1 = 0.10<br>H → L+4 = –0.33

[a] Only transitions with oscillator strengths >0.1 are presented. [b] % contribution = 2 c^2 * 100%, H = HOMO, L = LUMO).

Table S14b. TD-DFT results for **C1Py-BCF** at the B3LYP/6-31G(d,p) // cam-B3LYP/6-31G(d,p) (SCRF=acetonitrile) level of theory.

Transition[a]	E_{exc}, eV	λ, nm	Oscillator Strength, *f*	Assignment (coefficients, *c*)[b]
**$S_0 \rightarrow S_1$**	**2.43**	**509.2**	**1.166**	**H → L = 0.68**<br>H → L+2 = –0.16
**$S_0 \rightarrow S_6$**	**4.33**	**286.2**	**0.494**	H–15 → L = –0.13<br>H–13 → L = –0.13<br>**H–10 → L = 0.33**<br>H–9 → L = 0.10<br>H–8 → L = –0.14<br>H–7 → L = 0.18<br>H–5 → L = 0.18<br>**H–4 → L = 0.24**<br>H–4 → L+1 = –0.15<br>**H–1 → L = –0.21**<br>H → L+7 = –0.19
**$S_0 \rightarrow S_{10}$**	**4.57**	**271.0**	**0.588**	H–15 → L = –0.24<br>H–10 → L = 0.17<br>H–7 → L = 0.10<br>**H–1 → L = 0.33**<br>**H → L+7 = 0.43**<br>H → L+10 = –0.12

[a] Only transitions with oscillator strengths >0.2 are presented. [b] % contribution = 2 c^2 * 100%, H = HOMO, L = LUMO).

Table S15a. TD-DFT results for **1Py-Me^{2+}** (with counterions) at the B3LYP/6-31G(d,p) // cam-B3LYP/6-31G(d,p) (SCRF=acetonitrile) level of theory.

Transition[a]	E_{exc}, eV	λ, nm	Oscillator Strength, *f*	Assignment (coefficients, *c*)[b]
**$S_0 \rightarrow S_1$**	**2.05**	**604.7**	**0.751**	**H → L = 0.68**<br>H → L+2 = 0.16
$S_0 \rightarrow S_7$	3.91	316.7	0.203	H–6 → L = –0.14<br>H–3 → L = –0.33<br>H → L+4 = 0.43<br>H → L+6 = –0.34
$S_0 \rightarrow S_8$	4.10	302.7	0.392	H–8 → L+1 = 0.10<br>H–6 → L = 0.16<br>H–3 → L = 0.50<br>H–1 → L+1 = –0.12<br>H → L+4 = 0.31<br>H → L+6 = –0.23

[a] Only transitions with oscillator strengths >0.1 are presented. [b] % contribution = 2 c^2 * 100%, H = HOMO, L = LUMO).

Table S15b. TD-DFT results for **C1Py-Me^{2+}** (with counterions) at the B3LYP/6-31G(d,p) // cam-B3LYP/6-31G(d,p) (SCRF=acetonitrile) level of theory.

Transition[a]	E_{exc}, eV	λ, nm	Oscillator Strength, *f*	Assignment (coefficients, *c*)[b]
**$S_0 \rightarrow S_1$**	**2.37**	**524.1**	**1.111**	**H → L = 0.67**<br>H → L+2 = 0.19
$S_0 \rightarrow S_3$	3.53	350.9	0.138	H–1 → L = –0.13<br>H → L = –0.15<br>H → L+2 = 0.62<br>H → L+5 = 0.17
**$S_0 \rightarrow S_6$**	**4.27**	**290.5**	**0.476**	H–6 → L = 0.11<br>H–5 → L = 0.11<br>**H–4 → L = 0.52**<br>H–3 → L = –0.17<br>H → L+5 = –0.15
$S_0 \rightarrow S_7$	4.29	289.0	0.128	H–4 → L = 0.27<br>H–3 → L = 0.47<br>H–2 → L = –0.22
**$S_0 \rightarrow S_{10}$**	**4.52**	**274.3**	**0.487**	H–5 → L = 0.21<br>**H–1 → L = 0.33**<br>**H → L+5 = 0.46**

[a] Only transitions with oscillator strengths >0.1 are presented. [b] % contribution = 2 c^2 * 100%, H = HOMO, L = LUMO).

TD-DFT Computations of Reduced Species

Table S16. TD-DFT results for **1Py-BCF**$^{-\cdot}$ (radical anion, counter cation not included) at the UB3LYP/6-31G(d,p) // cam-UB3LYP/6-31G(d,p) (SCRF=acetonitrile) level of theory.

Transition[a]	E_{exc}, eV	λ, nm	Oscillator Strength, *f*	Assignment (coefficients, *c*)[b]
**$S_0 \rightarrow S_1$**	**1.24**	**997.1**	**0.677**	α: H → L+1 = –0.187<br>**α: SOMO → L = 0.903**<br>α: SOMO → L+2 = –0.190<br>β: H → SOMO = 0.263
**$S_0 \rightarrow S_2$**	**1.30**	**950.8**	**0.844**	α: H → L+1 = 0.159<br>α: SOMO → L = –0.201<br>**β: H → SOMO = 0.883**<br>β: H → L+1 = –0.307
**$S_0 \rightarrow S_7$**	**2.83**	**438.2**	**0.307**	α: H–2 → L+22 = –0.128<br>α: H → L+1 = –0.312<br>α: H → L+7 = 0.101<br>α: H → L+10 = –0.124<br>α: H → L+12 = –0.112<br>α: SOMO → L = –0.235<br>α: SOMO → L+2 = –0.107<br>β: H–12 → SOMO = –0.128<br>β: H–5 → L = –0.193<br>β: H–2 → SOMO = 0.240<br>β: H–1 → SOMO = –0.130<br>β: H → L+22 = 0.134<br>β: H → SOMO = 0.255<br>**β: H → L+1 = 0.575**<br>β: H → L+12 = 0.215<br>β: H → L+16 = 0.141
$S_0 \rightarrow S_8$	2.98	416.7	0.108	α: H → L = 0.676<br>α: H → L+2 = –0.150<br>α: H → L+13 = –0.105<br>β: H–5 → SOMO = –0.117<br>β: H → L = 0.616

[a] Only transitions with oscillator strengths >0.1 are presented. [b] % contribution = c^2 * 100%, H = HOMO, L = LUMO).

Table S17. TD-DFT results for **1Py-BCF**$^{2-}$ (counter cations not included) at the B3LYP/6-31G(d,p) // cam-B3LYP/6-31G(d,p) (SCRF=acetonitrile) level of theory.

Transition[a]	E_{exc}, eV	λ, nm	Oscillator Strength, *f*	Assignment (coefficients, *c*)[b]
**$S_0 \rightarrow S_1$**	**1.77**	**700.6**	**2.51**	**H–1 → L+2 = –0.15**<br>**H → L = 0.68**
**$S_0 \rightarrow S_2$**	**2.22**	**559.2**	**0.22**	H–1 → L+1 = –0.13<br>H–1 → L+5 = 0.10<br>**H → L+1 = 0.65**

				H → L+5 = 0.13<br>H → L+18 = 0.11
$S_0 \rightarrow S_4$	2.86	433.0	0.12	H–1 → L+1 = 0.12<br>H–1 → L+3 = 0.13<br>H–1 → L+5 = 0.19<br>H → L+1 = –0.18<br>H → L+5 = 0.46<br>H → L+7 = 0.30<br>H → L+11 = 0.22<br>H → L+15 = 0.14
$S_0 \rightarrow S_5$	2.89	429.4	0.11	H–1 → L+1 = 0.66<br>H → L+1 = 0.14
$S_0 \rightarrow S_6$	3.45	359.8	0.16	H–1 → L+2 = 0.65<br>H–1 → L+19 = 0.11<br>H → L+2 = 0.16

[a] Only transitions with oscillator strengths >0.1 are presented. [b] % contribution = 2 c^2 * 100%, H = HOMO, L = LUMO).

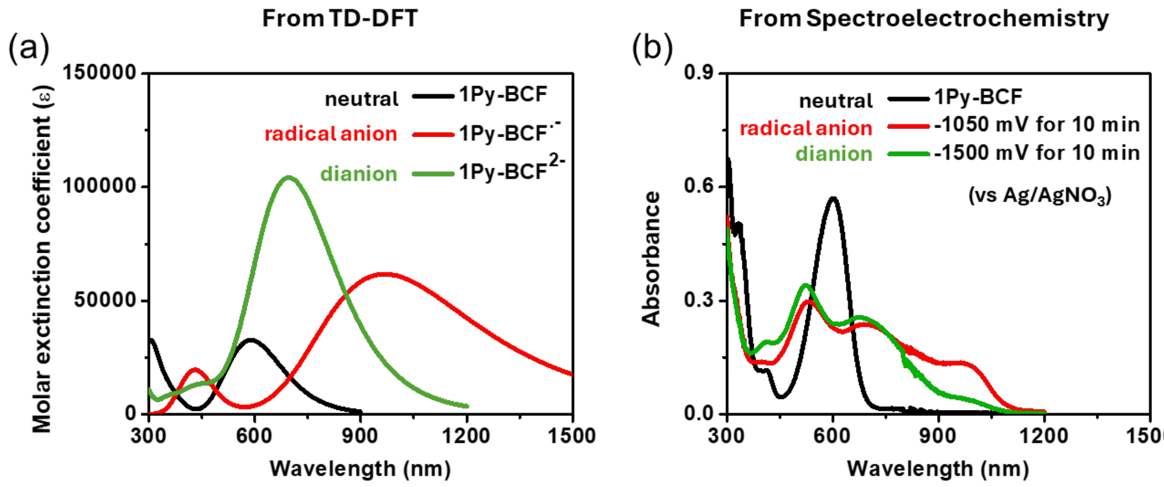

Figure S81. Comparison of calculated (TD-DFT) (a) and experimentally observed (spectroelectrochemistry) (b) UV-Vis absorption spectra for different redox states of **1Py-BCF**. TD-DFT calculations were performed at the R/U-B3LYP/6-31G(d,p) // cam-R/U-B3LYP/6-31G(d,p) (SCRF=acetonitrile) level.

Table S18. Comparison of calculated (TD-DFT) and experimentally observed (spectroelectrochemistry) UV-Vis absorption maxima for different redox states of **1Py-BCF**. TD-DFT calculations were performed at the R/U-B3LYP/6-31G(d,p) // cam-R/U-B3LYP/6-31G(d,p) (SCRF=acetonitrile) level.

**Species**	**λ (nm) from TD-DFT**	**λ (nm) from Spectroelectrochemistry**
**1Py-BCF**	589	609
**[1Py-BCF]$^{\cdot-}$** (radical anion)	997, 951, 438	1000 (shoulder), 688, 531, 401
**[1Py-BCF]$^{2-}$** (dianion, closed shell)	701, 559, 433	674, 522, 413

Table S19. TD-DFT results for **[1Py-Me]$^{+\cdot}$** (radical cation, counter anion not included) at the UB3LYP/6-31G(d,p) // cam-UB3LYP/6-31G(d,p) (SCRF=acetonitrile) level of theory.

Transition[a]	E_{exc}, eV	λ, nm	Oscillator Strength, *f*	Assignment (coefficients, *c*)[b]
**$S_0 \rightarrow S_1$**	**0.98**	**1263.2**	**0.950**	α: H–1 → L = –0.24<br>**α: SOMO → L = 0.95**<br>α: SOMO → L+1 = 0.10<br>α: SOMO ← L = 0.12
**$S_0 \rightarrow S_2$**	**1.27**	**973.5**	**0.485**	α: H–1 → L = 0.15<br>**β: H–1 → SOMO = 0.90**<br>β: H–1 → L = –0.35
$S_0 \rightarrow S_7$	2.71	457.3	0.231	α: H–1 → L = –0.43<br>α: SOMO → L+1 = 0.11<br>α: SOMO → L+2 = –0.18<br>α: SOMO → L = 0.13<br>α: SOMO → L+1 = –0.25<br>α: SOMO → L+2 = 0.13<br>β: H–1 → SOMO = –0.14<br>β: H–1 → L = 0.18<br>β: H–1 → SOMO+1 = 0.18<br>β: H–1 → L+6 = 0.12<br>β: SOMO → L = 0.32<br>β: SOMO → L = 0.55<br>β: SOMO → L+6 = –0.26
$S_0 \rightarrow S_8$	2.77	447.3	0.139	α: H–1 → L = –0.48<br>β: H–1 → SOMO = 0.80<br>β: H–1 → L+6 = 0.15

[a] Only transitions with oscillator strengths >0.1 are presented. [b] % contribution = c^2 * 100%, SOMO = Singly occupied molecular orbital, H = HOMO, L = LUMO.

Table S20. TD-DFT results for **[1Py-Me]^0** (neutral) at the B3LYP/6-31G(d,p) // cam-B3LYP/6-31G(d,p) (SCRF=acetonitrile) level of theory.

Transition[a]	E_{exc}, eV	λ, nm	Oscillator Strength, *f*	Assignment (coefficients, *c*)[b]
**$S_0 \rightarrow S_1$**	**1.68**	**735.8**	**2.256**	H–1 → L+1 = –0.14<br>**H → L = 0.69**
**$S_0 \rightarrow S_2$**	**2.18**	**568.7**	**0.219**	H–1 → L = –0.10<br>H–1 → L+2 = 0.12<br>**H → L+1 = 0.66**<br>H → L+3 = 0.12<br>H → L+6 = 0.10
$S_0 \rightarrow S_4$	2.70	458.7	0.132	H–1 → L = 0.67<br>H → L+1 = 0.12
$S_0 \rightarrow S_6$	3.31	374.1	0.126	H–1 → L+1 = 0.65<br>H → L = 0.14<br>H → L+2 = –0.17

[a] Only transitions with oscillator strengths >0.11 are presented. [b] % contribution = 2 c^2 * 100%, H = HOMO, L = LUMO).

Table S21. TD-DFT results for **[1Py-Me]**$^{\cdot-}$ (radical anion, counter cation not included) at the UB3LYP/6-31G(d,p) // cam-UB3LYP/6-31G(d,p) (SCRF=acetonitrile) level of theory.

Transition[a]	E_{exc}, eV	λ, nm	Oscillator Strength, *f*	Assignment (coefficients, *c*)[b]
**$S_0 \rightarrow S_1$**	**1.021**	**1214.9**	**0.805**	α: H–1 → L = –0.23<br>α: H → L+1 = 0.19<br>**α: SOMO → L = 0.94**<br>α: SOMO ← L = 0.12
**$S_0 \rightarrow S_2$**	**1.620**	**765.3**	**0.809**	β: H–1 → L = –0.22<br>β: H–1 → L+2 = 0.10<br>β: H–1 → L+6 = 0.10<br>**β: H → L = 0.90**<br>β: H → L+1 = 0.20<br>β: H → L+7 = –0.12
**$S_0 \rightarrow S_{11}$**	**2.634**	**470.7**	**0.330**	α: H–1 → L = 0.36<br>α: H → L = –0.18<br>α: SOMO → L+1 = 0.36<br>α: SOMO → L+2 = 0.12<br>α: SOMO → L+3 = 0.40<br>α: SOMO → L+4 = 0.16<br>α: SOMO → L+5 = –0.38<br>β: H–1 → L+2 = –0.16<br>β: H → L = 0.19<br>β: H → L+1 = –0.39<br>β: H → L+3 = –0.22

[a] Only transitions with oscillator strengths >0.1 are presented. [b] % contribution = c^2 * 100%, SOMO = Singly occupied molecular orbital, H = HOMO, L = LUMO.

Table S22. TD-DFT results for **[1Py-Me]**$^{2-}$ (dianion, open shell, counter cations not included) at the UB3LYP/6-31G(d,p) // cam-UB3LYP/6-31G(d,p) (SCRF=acetonitrile) level of theory.

Transition[a]	E_{exc}, eV	λ, nm	Oscillator Strength, *f*	Assignment (coefficients, *c*)[b]
**$S_0 \rightarrow S_3$**	**1.01**	**1231.9**	**1.535**	α: H–1 → L+1 = 0.12<br>**α: H → L = 0.70**<br>β: H–1 → L+1 = 0.12<br>**β: H → L = 0.70**<br>α: H → L+1 = –0.12<br>β: H → L+1 = –0.12
**$S_0 \rightarrow S_{10}$**	**1.88**	**661.2**	**0.225**	α: H–1 → L+1 = –0.27<br>α: H–1 → L+4 = 0.18<br>α: H → L = 0.11<br>**α: H → L+2 = 0.58**<br>α: H → L+5 = –0.12<br>β: H–1 → L+1 = –0.27<br>β: H–1 → L+4 = 0.18<br>β: H → L = 0.11<br>**β: H → L+2 = 0.58**<br>β: H → L+5 = –0.12

**$S_0 \rightarrow S_{18}$**	**2.74**	**452.0**	**0.677**	**α: H → L+3 = 0.67**<br>**β: H → L+3 = 0.67**

[a] Only transitions with oscillator strengths >0.1 are presented. [b] % contribution = $2c^2$ * 100%, H = HOMO, L = LUMO.

Table S23. TD-DFT results for **[1Py-Me]$^{2-}$** (dianion, closed shell, counter cations not included) at the RB3LYP/6-31G(d,p) // cam-RB3LYP/6-31G(d,p) (SCRF=acetonitrile)).

Transition[a]	E_{exc}, eV	λ, nm	Oscillator Strength, *f*	Assignment (coefficients, *c*)[b]
**$S_0 \rightarrow S_1$**	**1.18**	**1046.9**	**1.65**	H → L+1 = 0.14<br>**H → L = 0.69**
**$S_0 \rightarrow S_3$**	**2.01**	**615.6**	**0.25**	H → L+1 = –0.31<br>H → L+5 = 0.19<br>**H → L+2 = 0.56**<br>H → L+4 = –0.12
**$S_0 \rightarrow S_7$**	**2.88**	**430.8**	**0.68**	H–1 → L+3 = –0.11<br>**H → L+3 = 0.68**
$S_0 \rightarrow S_8$	3.00	412.9	0.13	H–1 → L = 0.34<br>H → L+1 = 0.16<br>H → L+2 = 0.24<br>H → L+4 = 0.47<br>H → L+6 = –0.14
$S_0 \rightarrow S_9$	3.08	402.2	0.21	H–1 → L = –0.11<br>H–1 → L+1 = –0.11<br>H → L = 0.11<br>H → L+2 = 0.24<br>H → L+3 = –0.11<br>H → L+6 = 0.16<br>H → L+1 = 0.24<br>H → L+5 = 0.41<br>H → L+7 = –0.35
$S_0 \rightarrow S_{10}$	3.14	395.4	0.28	H–1 → L = 0.57<br>H → L+4 = –0.33<br>H → L+5 = 0.13<br>H → L+6 = 0.16
$S_0 \rightarrow S_{16}$	4.14	299.3	0.18	H–1 → L+3 = –0.12<br>H–1 → L+4 = –0.13<br>H → L+5 = 0.54<br>H → L+7 = –0.24<br>H → L+2 = –0.10<br>H → L+9 = –0.21

[a] Only transitions with oscillator strengths >0.1 are presented. [b] % contribution = 2 c^2 * 100%, H = HOMO, L = LUMO).

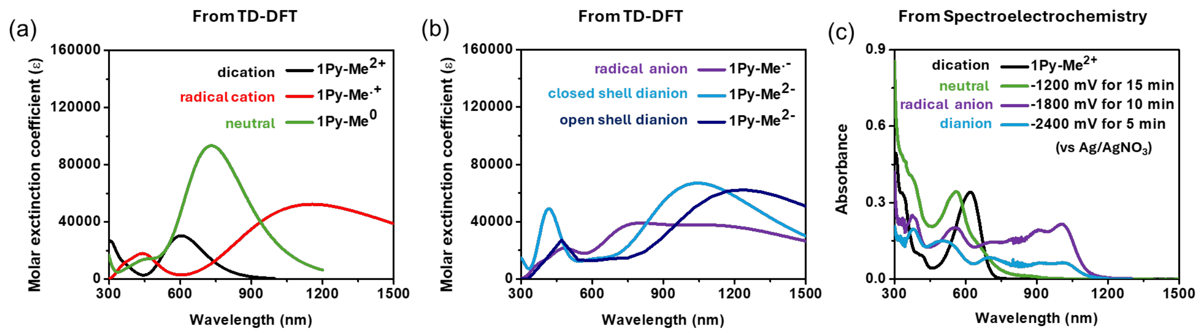

Figure S82. Comparison of calculated (TD-DFT) (a,b) and experimentally observed (spectroelectrochemistry) (c) UV-Vis absorption spectra for different redox states of **1Py-Me**. TD-DFT calculations were performed at the R/U-B3LYP/6-31G(d,p) // cam-R/U-B3LYP/6-31G(d,p) (SCRF=acetonitrile) level.

Table S24. Comparison of calculated (TD-DFT) and experimentally observed (spectroelectrochemistry) UV-Vis absorption maxima for different redox states of **1Py-Me** (counterions are omitted except for **1Py-Me^{2+}**). TD-DFT calculations were performed at the R/U-B3LYP/6-31G(d,p) // cam-R/U-B3LYP/6-31G(d,p) (SCRF=acetonitrile) level.

**Species**	**λ (nm) from TD-DFT**	**λ (nm) from Spectroelectrochemistry**
**[1Py-Me]$^{2+}$**$(PF_6)_2$	605	619
**[1Py-Me]$^{+\cdot}$** (radical cation)	1263, 974	Not observed
**[1Py-Me]** (neutral)	736, 569	654 (shoulder), 558, 375 (sh)
**[1Py-Me]$^{-\cdot}$** (radical anion)	1215, 765, 471	1002, 906, 696, 557, 375
**[1Py-Me]$^{2-}$** (dianion)	1047, 616, 431 (closed shell)<br>1232, 661, 452 (open shell)	1014, 909, 700, 502, 378

Table S25a. Comparison of LUMO energies obtained from cyclic voltammetry with those calculated from geometry optimizations using different functionals with the 6-31G(d,p) basis set, with and without solvation (SCRF=acetonitrile).

	$E_{LUMO, CV}$ (eV)	$E_{LUMO, DFT}$ (eV)				
		B3LYP (Gas phase)	B3LYP (ACN)	cam-B3LYP (ACN)	wb97xd (ACN)	wb97xd (Gas phase)
**1H**	-3.22	-2.63	-2.61	-1.53	-1.03	-1.03
**1Py**	-3.38	-2.93	-2.81	-1.74	-1.24	-1.33
**1Py-BCF**	-3.65	-3.80	-3.14	-2.03	-1.52	-2.16
**[1Py-Me]$^{2+}$$(PF_6)_2$**	-3.78	-3.88	-3.29	-2.16	-1.63	-1.76
**[1Py-Me]$^{2+}$**		-7.70	-3.44	-2.31	-1.78	-6.05

Note: B3LYP/6-31G(d,p) (SCRF=acetonitrile) provides the best agreement, and for **1Py-Me^{2+}** inclusion of counter anions further improves the accuracy.

Table S25b. Comparison of the absorption maxima obtained from UV-Vis absorption spectra with the values calculated using TD-DFT (B3LYP or CAM-B3LYP/6-31G(d,p), with SCRF=acetonitrile). The geometries used for the calculations were optimized at the B3LYP/6-31G(d,p) level of theory in acetonitrile.

Compound	λ_{max} (nm) (ACN)	λ (nm) from TD-DFT	
		B3LYP (ACN)	CAM-B3LYP (ACN)
**1H**	547	610	523
**1Py**	577	661	554
**1Py-BCF**	609	739	589
**[1Py-Me]$^{2+}$(PF$_6$)$_2$**	619	790	605

Note: cam-B3LYP/6-31G(d,p) (SCRF=acetonitrile) provides the best agreement.

Table S26. Comparison of computed singlet-triplet gaps (B3LYP/6-31G(d,p), with SCRF=acetonitrile).

	**S_0 (Hartree)**	**T_1 (Hartree)[a]**	**$\Delta(S_0\text{-}T_1)$ (Hartree)**	**$\Delta(S_0\text{-}T_1)$ (kJ/mol)**
**1H**	-1399.250207	-1399.213188	0.037019	97.2
**1Py**	-1893.451264	-1893.416278	0.034986	91.9
**1Py-BCF**	-6309.989043	-6309.956971	0.032072	84.2
**1Py-Me^{2+}(PF$_6$)$_2$**	-3854.465220	-3854.434518	0.030702	80.6

[a] Geometry optimized for triplet state.

Table S27. Coordinates for Optimized Structure of **1H** in the ground state (S_0).

E(RB3LYP) = -1399.250207 Hartree.

opt freq RB3LYP/6-31G(d,p) scrf=(solvent=acetonitrile)

Center Number	Atomic Number	Atomic Type	Coordinates (Angstroms) X	Y	Z
1	7	0	3.802339	0.597819	-0.207593
2	7	0	-3.658807	-0.574085	-0.380293
3	6	0	-1.124251	0.794080	0.061917
4	6	0	1.248875	-0.784043	0.124309
5	6	0	0.178343	1.423627	0.062359
6	6	0	-2.466195	-1.192071	-0.630908
7	6	0	1.341956	0.615444	-0.096755
8	6	0	-0.055145	-1.412489	0.098415
9	6	0	-1.210442	-0.609494	-0.125272
10	6	0	-2.447101	-2.316199	-1.478556
11	1	0	-1.494572	-2.750280	-1.747953
12	6	0	2.621869	1.199635	-0.535611
13	6	0	-4.833135	-2.246322	-1.652838
14	6	0	0.229878	2.831165	0.296432
15	1	0	1.189228	3.324930	0.389785
16	6	0	-3.621438	-2.850188	-1.986236
17	1	0	-3.589570	-3.716068	-2.639002
18	6	0	2.648001	2.329126	-1.378252
19	1	0	1.711810	2.743080	-1.724908
20	6	0	2.438172	-1.546984	0.388551
21	6	0	-2.325785	1.555639	0.268539
22	6	0	-4.800364	-1.107684	-0.867097
23	1	0	-5.706999	-0.579547	-0.611555
24	6	0	-2.187555	2.918640	0.458185
25	1	0	-3.069173	3.525348	0.644782
26	6	0	-0.125890	-2.811215	0.367515
27	1	0	-1.092611	-3.295791	0.431845
28	6	0	5.041053	2.326865	-1.335123
29	6	0	-0.922110	3.553089	0.486093
30	1	0	-0.859196	4.617938	0.694061
31	6	0	2.280888	-2.902105	0.625038
32	1	0	3.152031	-3.507487	0.859061
33	6	0	4.968060	1.173626	-0.576160
34	1	0	5.861594	0.660870	-0.249793
35	6	0	-5.032161	1.692797	0.086916
36	1	0	-5.978882	1.190061	0.334633
37	1	0	-5.031325	2.569364	0.751735
38	6	0	3.847003	2.897243	-1.777316
39	1	0	3.849739	3.766564	-2.426270
40	6	0	1.013774	-3.530942	0.628146
41	1	0	0.938751	-4.588557	0.866641
42	6	0	5.095718	-1.737323	-0.051928
43	1	0	6.080954	-1.308160	0.187912
44	1	0	5.093185	-2.691101	0.495221
45	6	0	-3.800805	0.310401	2.097999
46	1	0	-2.894974	-0.260788	2.352214
47	1	0	-3.746314	1.221686	2.712622
48	6	0	4.199881	-0.500369	2.165422
49	1	0	4.324981	-1.467047	2.677032
50	1	0	5.186905	-0.013382	2.227724
51	6	0	3.185720	0.343355	2.951622
52	1	0	2.201451	-0.137465	2.982286

53	1	0	3.043889	1.333218	2.500153
54	1	0	3.499604	0.509282	3.990304
55	6	0	-5.100131	2.190335	-1.367883
56	1	0	-5.971142	2.832306	-1.550347
57	1	0	-5.161296	1.360774	-2.083307
58	1	0	-4.206536	2.766923	-1.630588
59	5	0	-3.732749	0.793483	0.518529
60	6	0	5.034466	-2.043733	-1.557246
61	1	0	4.091294	-2.535997	-1.820267
62	1	0	5.850167	-2.700367	-1.885159
63	1	0	5.097162	-1.131334	-2.163445
64	6	0	-5.029866	-0.503985	2.532013
65	1	0	-5.962023	0.052004	2.378925
66	1	0	-4.989687	-0.777698	3.594255
67	1	0	-5.118817	-1.440818	1.967657
68	5	0	3.877311	-0.835921	0.585864
69	1	0	6.008113	2.739025	-1.596362
70	1	0	-5.782962	-2.624968	-2.010406

Table S28. Coordinates for Optimized Structure of **1H** in the first triplet state (T_1).

E(UB3LYP) = -1399.213188 Hartree.

opt freq UB3LYP/6-31G(d,p) scrf=(solvent=acetonitrile)

Center Number	Atomic Number	Atomic Type	Coordinates (Angstroms) X	Y	Z
1	7	0	3.752836	0.574341	-0.362248
2	7	0	-3.601009	-0.560002	-0.486076
3	6	0	-1.145228	0.802139	0.204688
4	6	0	1.266154	-0.783780	0.265453
5	6	0	0.137515	1.435100	0.233984
6	6	0	-2.392517	-1.220698	-0.587104
7	6	0	1.334966	0.635244	-0.026244
8	6	0	-0.019070	-1.413583	0.297678
9	6	0	-1.206453	-0.627371	-0.028328
10	6	0	-2.335461	-2.423353	-1.337465
11	1	0	-1.375882	-2.902652	-1.472213
12	6	0	2.546404	1.219825	-0.538653
13	6	0	-4.682357	-2.274914	-1.790381
14	6	0	0.195230	2.789683	0.593860
15	1	0	1.154871	3.282166	0.700770
16	6	0	-3.463585	-2.950175	-1.933696
17	1	0	-3.397640	-3.862220	-2.517541
18	6	0	2.525962	2.417915	-1.301463
19	1	0	1.570878	2.875997	-1.516582
20	6	0	2.450138	-1.504902	0.534544
21	6	0	-2.338978	1.523983	0.423683
22	6	0	-4.695126	-1.092839	-1.072522
23	1	0	-5.609348	-0.531486	-0.946312
24	6	0	-2.215067	2.891669	0.748863
25	1	0	-3.111201	3.474291	0.941170
26	6	0	-0.095181	-2.749354	0.715362
27	1	0	-1.061591	-3.230903	0.813914
28	6	0	4.907049	2.330747	-1.543302
29	6	0	-0.975626	3.511840	0.850673
30	1	0	-0.906239	4.558830	1.131742
31	6	0	2.307818	-2.852439	0.934772

32	1	0	3.196695	-3.428255	1.175835
33	6	0	4.882729	1.142171	-0.840321
34	1	0	5.794137	0.596328	-0.640053
35	6	0	-4.982842	1.719948	-0.128786
36	1	0	-5.963036	1.230621	-0.031187
37	1	0	-5.058031	2.575699	0.558216
38	6	0	3.686680	2.971049	-1.800557
39	1	0	3.648540	3.879360	-2.392657
40	6	0	1.064617	-3.461905	1.041869
41	1	0	0.983673	-4.492426	1.375507
42	6	0	4.981538	-1.798310	-0.329215
43	1	0	6.003589	-1.394329	-0.264770
44	1	0	5.037669	-2.736584	0.240895
45	6	0	-4.055224	0.242254	1.982214
46	1	0	-3.204421	-0.369710	2.318706
47	1	0	-4.048040	1.128662	2.633873
48	6	0	4.489607	-0.454240	1.958203
49	1	0	4.654848	-1.404691	2.487478
50	1	0	5.491008	-0.008951	1.844689
51	6	0	3.634539	0.463825	2.842568
52	1	0	2.644519	0.032327	3.029940
53	1	0	3.473591	1.442518	2.374005
54	1	0	4.098787	0.650282	3.819548
55	6	0	-4.837653	2.259358	-1.562610
56	1	0	-5.667147	2.918245	-1.848631
57	1	0	-4.803335	1.449982	-2.302415
58	1	0	-3.909987	2.831262	-1.678036
59	5	0	-3.769062	0.774450	0.437048
60	6	0	4.668405	-2.137404	-1.795547
61	1	0	3.683979	-2.610361	-1.890335
62	1	0	5.404689	-2.822958	-2.233673
63	1	0	4.652428	-1.239994	-2.426330
64	6	0	-5.354438	-0.539416	2.227146
65	1	0	-6.240888	0.056796	1.982253
66	1	0	-5.454986	-0.849935	3.275213
67	1	0	-5.403001	-1.452267	1.620692
68	5	0	3.908918	-0.827035	0.457305
69	1	0	5.850684	2.726595	-1.898246
70	1	0	-5.599158	-2.642115	-2.235159

Table S29. Coordinates for Optimized Structure of **1Py** in the ground state (S_0).

E(RB3LYP) = -1893.451264 Hartree.

opt freq RB3LYP/6-31G(d,p) scrf=(solvent=acetonitrile)

Center Number	Atomic Number	Atomic Type	Coordinates (Angstroms) X	Y	Z
1	7	0	3.772213	-0.592890	0.289208
2	7	0	-3.687292	0.569815	0.247287
3	7	0	-9.451213	-1.086511	-2.245933
4	6	0	-0.849417	1.113880	0.620951
5	6	0	0.930182	-1.113836	0.722662
6	6	0	0.582499	1.313852	0.584698
7	6	0	-2.743560	-0.392854	0.022847
8	6	0	1.440063	0.182655	0.443805
9	6	0	-0.503205	-1.312973	0.739528
10	6	0	-1.363238	-0.203135	0.493044

11	6	0	-3.092081	-1.501001	-0.773022
12	1	0	-2.329568	-2.223410	-1.027291
13	6	0	2.821987	0.331949	-0.036086
14	6	0	-5.362586	-0.716452	-0.935840
15	6	0	1.067700	2.643736	0.764363
16	1	0	2.133716	2.822078	0.832891
17	6	0	-4.380489	-1.669002	-1.245771
18	1	0	-4.624466	-2.540759	-1.843245
19	6	0	3.176530	1.371031	-0.920077
20	1	0	2.409309	2.048743	-1.266071
21	6	0	1.835179	-2.195130	0.998328
22	6	0	-6.767412	-0.841959	-1.384675
23	6	0	-1.755678	2.214027	0.801545
24	6	0	-4.944083	0.392395	-0.203277
25	1	0	-5.629245	1.197379	0.017234
26	6	0	-1.202853	3.475024	0.936037
27	1	0	-1.852835	4.329473	1.101586
28	7	0	9.609903	1.024538	-2.053663
29	6	0	-0.992114	-2.610391	1.070312
30	1	0	-2.058634	-2.772043	1.169942
31	6	0	5.470931	0.640669	-0.916857
32	6	0	0.195920	3.690938	0.933082
33	1	0	0.585836	4.691576	1.099145
34	6	0	6.892713	0.771437	-1.306911
35	6	0	1.276791	-3.427254	1.295131
36	1	0	1.926831	-4.262417	1.539904
37	6	0	5.044687	-0.415454	-0.116396
38	1	0	5.736122	-1.184108	0.197719
39	6	0	-4.289451	3.169614	0.607202
40	1	0	-5.342296	2.996046	0.875187
41	1	0	-4.010889	4.031496	1.231394
42	6	0	4.477897	1.530130	-1.356635
43	1	0	4.724370	2.349811	-2.022737
44	6	0	-7.091035	-1.435871	-2.613871
45	1	0	-6.317906	-1.798947	-3.282416
46	6	0	-0.120958	-3.635552	1.345565
47	1	0	-0.509837	-4.609333	1.630808
48	6	0	4.289281	-3.209225	0.507760
49	1	0	5.366169	-3.098617	0.709496
50	1	0	4.011520	-4.098231	1.091979
51	6	0	-3.521440	1.562005	2.681467
52	1	0	-2.830132	0.753659	2.964063
53	1	0	-3.185390	2.439391	3.254377
54	6	0	-7.833838	-0.377175	-0.600288
55	1	0	-7.661269	0.066806	0.374206
56	6	0	3.891519	-1.686686	2.693092
57	1	0	3.733633	-2.628971	3.239757
58	1	0	4.981575	-1.523498	2.713024
59	6	0	-8.428551	-1.532332	-2.990832
60	1	0	-8.688534	-1.987310	-3.944295
61	6	0	-9.137101	-0.520063	-1.071489
62	1	0	-9.970343	-0.166039	-0.468122
63	6	0	7.263453	1.278429	-2.561446
64	1	0	6.517101	1.567780	-3.293309
65	6	0	7.929271	0.398223	-0.438086
66	1	0	7.718859	0.024674	0.558272
67	6	0	8.615581	1.383130	-2.879561
68	1	0	8.912122	1.770944	-3.851850
69	6	0	3.210002	-0.549489	3.468190
70	1	0	2.127633	-0.705041	3.540129
71	1	0	3.361938	0.422352	2.982198

72	1	0	3.594396	-0.456724	4.492045
73	6	0	-4.216346	3.598504	-0.869088
74	1	0	-4.848841	4.469076	-1.083142
75	1	0	-4.538115	2.797685	-1.546573
76	1	0	-3.191316	3.858780	-1.154541
77	6	0	9.250772	0.541022	-0.855310
78	1	0	10.060793	0.257539	-0.186612
79	5	0	-3.325072	1.934732	1.083458
80	6	0	4.087144	-3.527661	-0.982640
81	1	0	3.030468	-3.714714	-1.204912
82	1	0	4.651753	-4.412519	-1.302038
83	1	0	4.405678	-2.697777	-1.625906
84	6	0	-4.935602	1.181395	3.147461
85	1	0	-5.655981	1.987040	2.964529
86	1	0	-4.969597	0.957543	4.221407
87	1	0	-5.310713	0.290311	2.628130
88	5	0	3.428354	-1.958271	1.136916

Table S30. Coordinates for Optimized Structure of **1Py** in the first triplet state (T_1).

E(UB3LYP) = -1893.416278 Hartree.

opt freq UB3LYP/6-31G(d,p) scrf=(solvent=acetonitrile)

Center Number	Atomic Number	Atomic Type	Coordinates (Angstroms) X	Y	Z
1	7	0	3.712929	-0.606381	0.256059
2	7	0	-3.635301	0.583314	0.228479
3	7	0	-9.202832	-1.150604	-2.639147
4	6	0	-0.864325	1.149591	0.835579
5	6	0	0.939836	-1.100363	0.962281
6	6	0	0.551694	1.353764	0.827346
7	6	0	-2.690740	-0.423766	0.165718
8	6	0	1.438731	0.212398	0.603617
9	6	0	-0.476063	-1.297357	1.037860
10	6	0	-1.370267	-0.199201	0.681497
11	6	0	-3.033066	-1.620293	-0.516118
12	1	0	-2.279787	-2.387518	-0.626610
13	6	0	2.758378	0.371840	0.061114
14	6	0	-5.245797	-0.778590	-0.968366
15	6	0	1.033548	2.639179	1.117898
16	1	0	2.100100	2.813214	1.200154
17	6	0	-4.277942	-1.799165	-1.074816
18	1	0	-4.507324	-2.725904	-1.589410
19	6	0	3.102354	1.482646	-0.755362
20	1	0	2.337403	2.208996	-0.990358
21	6	0	1.847470	-2.143659	1.251473
22	6	0	-6.602908	-0.904132	-1.537645
23	6	0	-1.770943	2.217877	1.016309
24	6	0	-4.850157	0.387508	-0.315502
25	1	0	-5.527692	1.224644	-0.234240
26	6	0	-1.222827	3.492447	1.270788
27	1	0	-1.890004	4.333505	1.434323
28	7	0	9.399812	0.987360	-2.454404
29	6	0	-0.952682	-2.522563	1.524965
30	1	0	-2.017637	-2.673249	1.660838
31	6	0	5.357299	0.665553	-0.992306
32	6	0	0.149824	3.700217	1.340168

33	1	0	0.545515	4.685477	1.569128
34	6	0	6.743276	0.774719	-1.490991
35	6	0	1.304030	-3.359561	1.721284
36	1	0	1.975773	-4.172629	1.980733
37	6	0	4.952998	-0.432443	-0.238704
38	1	0	5.642208	-1.239212	-0.032135
39	6	0	-4.228477	3.199185	0.444450
40	1	0	-5.310878	3.048461	0.568994
41	1	0	-4.021468	4.073541	1.078648
42	6	0	4.366248	1.629959	-1.276514
43	1	0	4.595885	2.491742	-1.893641
44	6	0	-6.844611	-1.667259	-2.691681
45	1	0	-6.035585	-2.167921	-3.212328
46	6	0	-0.063936	-3.544693	1.875473
47	1	0	-0.452030	-4.481548	2.264392
48	6	0	4.124027	-3.245068	0.338835
49	1	0	5.223078	-3.186557	0.366464
50	1	0	3.897067	-4.139274	0.936453
51	6	0	-3.775912	1.627985	2.642529
52	1	0	-3.150976	0.804001	3.018949
53	1	0	-3.485086	2.505911	3.238128
54	6	0	-7.713322	-0.272329	-0.953544
55	1	0	-7.614049	0.308581	-0.042999
56	6	0	4.156799	-1.757744	2.593287
57	1	0	4.025014	-2.695691	3.153002
58	1	0	5.244149	-1.661102	2.443984
59	6	0	-8.141023	-1.756507	-3.192040
60	1	0	-8.333024	-2.342446	-4.088588
61	6	0	-8.969047	-0.423261	-1.536140
62	1	0	-9.833112	0.060984	-1.085744
63	6	0	7.035018	1.402049	-2.713131
64	1	0	6.246304	1.804720	-3.339309
65	6	0	7.832289	0.262263	-0.766889
66	1	0	7.691125	-0.207131	0.200715
67	6	0	8.357843	1.480427	-3.141048
68	1	0	8.589434	1.960857	-4.089478
69	6	0	3.667009	-0.589061	3.459556
70	1	0	2.596681	-0.673953	3.679927
71	1	0	3.815649	0.375946	2.959633
72	1	0	4.194218	-0.535176	4.420598
73	6	0	-3.944213	3.581458	-1.018756
74	1	0	-4.532480	4.447511	-1.346659
75	1	0	-4.173746	2.760000	-1.708796
76	1	0	-2.886693	3.828968	-1.164362
77	6	0	9.117927	0.390236	-1.286386
78	1	0	9.965705	-0.001898	-0.728321
79	5	0	-3.362182	1.956680	1.069836
80	6	0	3.671686	-3.497461	-1.108444
81	1	0	2.585401	-3.633088	-1.165285
82	1	0	4.136844	-4.391514	-1.542075
83	1	0	3.922415	-2.655989	-1.766097
84	6	0	-5.249095	1.303227	2.929647
85	1	0	-5.912053	2.127218	2.642084
86	1	0	-5.424356	1.106068	3.995062
87	1	0	-5.585817	0.412209	2.385102
88	5	0	3.441378	-1.966061	1.119854

Table S31. Coordinates for Optimized Structure of **1Py-BCF** in the ground state (S_0).

E(RB3LYP) = -6309.989043 Hartree.

opt freq RB3LYP/6-31G(d,p) scrf=(solvent=acetonitrile)

Center Number	Atomic Number	Atomic Type	Coordinates (Angstroms) X	Y	Z
1	7	0	3.647628	-2.044050	0.834404
2	7	0	-3.638550	-1.979867	-1.101075
3	7	0	9.537212	0.505729	-0.034031
4	7	0	-9.530185	0.408493	0.071758
5	6	0	1.416994	-2.185538	-0.195103
6	6	0	-0.760932	-2.322579	-1.368325
7	6	0	0.683151	-2.268619	-1.416321
8	6	0	-4.908523	-1.543177	-1.029574
9	1	0	-5.503154	-1.715685	-1.914298
10	6	0	-1.408120	-2.269532	-0.105142
11	6	0	0.780075	-2.541576	1.024993
12	6	0	-5.442016	-0.875373	0.071515
13	6	0	2.796883	-1.684579	-0.172634
14	6	0	4.920035	-1.606356	0.822848
15	1	0	5.538965	-1.987116	1.622516
16	6	0	-1.546422	-2.444175	-2.564016
17	6	0	-0.665510	-2.576319	1.073222
18	6	0	5.439015	-0.742342	-0.137197
19	6	0	-2.801038	-1.811402	-0.032845
20	6	0	-6.850677	-0.437123	0.074334
21	6	0	3.245817	-0.746305	-1.125832
22	1	0	2.547074	-0.368996	-1.858519
23	6	0	1.305669	-2.372685	-2.695097
24	1	0	2.384953	-2.427809	-2.765545
25	6	0	4.545660	-0.282299	-1.119526
26	1	0	4.856030	0.455468	-1.851159
27	6	0	6.851772	-0.318458	-0.093626
28	6	0	-3.266988	-1.080546	1.078160
29	1	0	-2.584845	-0.867821	1.888749
30	6	0	-1.282986	-2.983876	2.290465
31	1	0	-2.359314	-3.097966	2.334838
32	6	0	-0.864493	-2.507583	-3.766446
33	1	0	-1.420957	-2.627960	-4.691414
34	6	0	1.570998	-2.873852	2.176338
35	6	0	-3.970396	-2.201857	-3.763672
36	1	0	-3.601806	-2.779111	-4.624310
37	1	0	-5.033619	-2.480552	-3.713083
38	6	0	-4.566863	-0.617056	1.138439
39	1	0	-4.900734	-0.071414	2.014013
40	6	0	3.902233	-2.439162	3.468821
41	1	0	3.544729	-3.085715	4.282852
42	1	0	4.987505	-2.625677	3.455977
43	6	0	0.548408	-2.487607	-3.835184
44	1	0	1.041428	-2.596864	-4.797247
45	6	0	7.558226	-0.199194	1.112084
46	1	0	7.086598	-0.393943	2.067240
47	6	0	-3.358189	-4.357582	-2.195802
48	1	0	-2.933422	-4.883207	-3.064099
49	1	0	-2.749549	-4.682183	-1.338386
50	6	0	8.882637	0.203735	1.105536
51	1	0	9.446114	0.281717	2.023629
52	6	0	7.555436	-0.017768	-1.273167

53	1	0	7.092152	-0.115878	-2.247122
54	6	0	-9.161881	-0.718413	-0.570537
55	1	0	-9.948389	-1.281438	-1.051214
56	6	0	0.891259	-3.245074	3.324829
57	1	0	1.453993	-3.534297	4.207643
58	6	0	-3.863258	-0.704648	-4.103900
59	1	0	-4.406524	-0.445507	-5.020997
60	1	0	-2.819060	-0.405134	-4.244333
61	1	0	-4.267654	-0.070145	-3.305235
62	6	0	-0.519370	-3.314228	3.383417
63	1	0	-1.003834	-3.656997	4.293539
64	6	0	-7.256564	0.723007	0.757285
65	1	0	-6.546103	1.344916	1.287415
66	6	0	-7.850798	-1.162163	-0.590140
67	1	0	-7.630492	-2.090723	-1.102066
68	6	0	8.874809	0.380354	-1.207662
69	1	0	9.443048	0.592879	-2.103850
70	6	0	3.646973	-0.975974	3.865883
71	1	0	2.573733	-0.775348	3.959076
72	1	0	4.037306	-0.273087	3.118872
73	1	0	4.114740	-0.714832	4.823157
74	6	0	-8.581562	1.106764	0.738821
75	1	0	-8.918471	1.989683	1.266261
76	6	0	3.691459	-4.517772	1.769875
77	1	0	4.792465	-4.518039	1.717270
78	1	0	3.447610	-5.126790	2.653658
79	6	0	-4.800948	-4.842067	-1.983518
80	1	0	-5.438105	-4.617533	-2.846614
81	1	0	-5.265911	-4.370974	-1.107989
82	1	0	-4.851086	-5.926107	-1.820367
83	6	0	3.139054	-5.220711	0.521288
84	1	0	2.047845	-5.312546	0.561287
85	1	0	3.546795	-6.232566	0.401997
86	1	0	3.380705	-4.670757	-0.396821
87	5	0	3.181042	-2.988109	2.098381
88	5	0	-3.133341	-2.743048	-2.465450
89	5	0	11.138970	0.845077	-0.081965
90	6	0	11.695667	1.295916	1.402733
91	6	0	11.727912	-0.585242	-0.657247
92	6	0	11.437535	2.162533	-1.026099
93	6	0	11.058463	2.313027	2.123875
94	6	0	12.905373	0.874835	1.965493
95	6	0	12.093189	-0.835700	-1.981438
96	6	0	11.754314	-1.717147	0.163922
97	6	0	12.758979	2.425043	-1.406137
98	6	0	10.537328	3.175912	-1.363406
99	6	0	11.527048	2.839147	3.323276
100	9	0	9.904818	2.844153	1.654450
101	6	0	13.416964	1.378945	3.160068
102	9	0	13.669893	-0.058259	1.363538
103	6	0	12.509572	-2.082017	-2.448036
104	9	0	12.030556	0.138966	-2.917334
105	6	0	12.161769	-2.978384	-0.254545
106	9	0	11.358832	-1.619456	1.454583
107	6	0	13.163362	3.559862	-2.097623
108	9	0	13.731441	1.541829	-1.087690
109	6	0	10.897222	4.330115	-2.060010
110	9	0	9.229433	3.091799	-1.028240
111	6	0	12.723871	2.366525	3.848350
112	9	0	10.845644	3.802182	3.962710
113	9	0	14.581202	0.920692	3.646222

114	6	0	12.549157	-3.162954	-1.577546
115	9	0	12.855851	-2.246032	-3.734765
116	9	0	12.174278	-4.015013	0.598311
117	6	0	12.219616	4.526074	-2.431796
118	9	0	14.450513	3.735686	-2.436591
119	9	0	9.972315	5.255421	-2.362806
120	9	0	13.202954	2.858107	4.997549
121	9	0	12.942141	-4.368583	-2.007542
122	9	0	12.584291	5.629670	-3.097515
123	5	0	-11.091716	0.875046	0.233397
124	6	0	-12.049757	0.144464	-0.891778
125	6	0	-11.372780	0.434741	1.798856
126	6	0	-11.266173	2.481221	-0.091568
127	6	0	-11.719459	0.196401	-2.251472
128	6	0	-13.316707	-0.391152	-0.636376
129	6	0	-11.362668	1.297779	2.896408
130	6	0	-11.496815	-0.916310	2.138661
131	6	0	-12.465922	3.107800	0.265539
132	6	0	-10.412722	3.280786	-0.855384
133	6	0	-12.527991	-0.284567	-3.276131
134	9	0	-10.541083	0.743149	-2.633385
135	6	0	-14.163363	-0.877134	-1.631277
136	9	0	-13.807556	-0.459211	0.617400
137	6	0	-11.522213	0.875527	4.215782
138	9	0	-11.160398	2.625545	2.736238
139	6	0	-11.658557	-1.385185	3.437040
140	9	0	-11.455254	-1.859114	1.168526
141	6	0	-12.788959	4.419948	-0.056000
142	9	0	-13.396717	2.416982	0.960951
143	6	0	-10.693570	4.603717	-1.198826
144	9	0	-9.232309	2.806322	-1.315069
145	6	0	-13.768072	-0.827730	-2.961975
146	9	0	-12.129290	-0.213689	-4.555426
147	9	0	-15.363415	-1.386511	-1.311597
148	6	0	-11.675855	-0.476820	4.490144
149	9	0	-11.511847	1.765259	5.221150
150	9	0	-11.786421	-2.698718	3.682844
151	6	0	-11.889910	5.180520	-0.797061
152	9	0	-13.957818	4.954647	0.331899
153	9	0	-9.816933	5.316609	-1.924472
154	9	0	-14.570403	-1.292722	-3.927321
155	9	0	-11.826080	-0.902358	5.750805
156	9	0	-12.178359	6.447799	-1.121015

Table S32. Coordinates for Optimized Structure of **1Py-BCF** in the first triplet state (T_1).

E(UB3LYP) = -6309.956971 Hartree.

opt freq UB3LYP/6-31G(d,p) scrf=(solvent=acetonitrile)

Center Number	Atomic Number	Atomic Type	Coordinates (Angstroms) X	Y	Z
1	7	0	3.579436	-2.277037	0.862269
2	7	0	-3.593279	-2.272484	-1.129853
3	7	0	9.344486	0.542001	-0.039964
4	7	0	-9.337830	0.443488	0.079779
5	6	0	1.417382	-2.621285	-0.218362
6	6	0	-0.768132	-2.829467	-1.407857

7	6	0	0.662198	-2.808215	-1.454908
8	6	0	-4.829881	-1.757732	-1.046656
9	1	0	-5.408276	-1.814295	-1.956910
10	6	0	-1.420554	-2.730310	-0.120726
11	6	0	0.780311	-3.024333	1.017104
12	6	0	-5.360164	-1.154352	0.095253
13	6	0	2.739134	-2.065945	-0.215481
14	6	0	4.817903	-1.758513	0.850060
15	1	0	5.424630	-2.020140	1.705294
16	6	0	-1.550158	-2.976313	-2.576420
17	6	0	-0.647950	-3.115859	1.054817
18	6	0	5.334980	-0.967878	-0.175156
19	6	0	-2.764037	-2.238073	-0.022820
20	6	0	-6.726078	-0.613071	0.090868
21	6	0	3.198498	-1.219182	-1.261192
22	1	0	2.514124	-0.959592	-2.056154
23	6	0	1.284950	-3.053117	-2.688559
24	1	0	2.364319	-3.128910	-2.746539
25	6	0	4.462219	-0.682027	-1.250521
26	1	0	4.763651	-0.009357	-2.045589
27	6	0	6.710662	-0.456313	-0.118311
28	6	0	-3.243648	-1.604640	1.153696
29	1	0	-2.578810	-1.510907	2.000485
30	6	0	-1.250595	-3.650525	2.202897
31	1	0	-2.323584	-3.801465	2.231291
32	6	0	-0.862637	-3.186599	-3.789518
33	1	0	-1.431297	-3.326139	-4.703816
34	6	0	1.571844	-3.346786	2.143133
35	6	0	-3.889172	-2.379319	-3.801398
36	1	0	-3.578860	-2.973710	-4.672874
37	1	0	-4.979864	-2.519146	-3.777952
38	6	0	-4.507926	-1.068282	1.218003
39	1	0	-4.839550	-0.592006	2.133735
40	6	0	3.716200	-2.491113	3.522828
41	1	0	3.402537	-3.134798	4.356518
42	1	0	4.815488	-2.516801	3.576879
43	6	0	0.525097	-3.244091	-3.846494
44	1	0	1.028298	-3.440158	-4.788628
45	6	0	7.390609	-0.250181	1.094779
46	1	0	6.916758	-0.439166	2.049749
47	6	0	-3.585501	-4.640292	-2.282871
48	1	0	-3.189394	-5.193866	-3.146640
49	1	0	-3.052435	-5.045821	-1.409852
50	6	0	8.685367	0.234688	1.097459
51	1	0	9.223671	0.378579	2.022746
52	6	0	7.423192	-0.148638	-1.294420
53	1	0	6.990441	-0.309276	-2.273840
54	6	0	-9.008929	-0.599659	-0.711380
55	1	0	-9.801059	-1.017143	-1.315499
56	6	0	0.903802	-3.866664	3.272932
57	1	0	1.484909	-4.154985	4.143701
58	6	0	-3.582111	-0.898754	-4.087141
59	1	0	-4.076200	-0.538704	-4.997848
60	1	0	-2.505771	-0.734639	-4.210775
61	1	0	-3.908550	-0.246214	-3.267840
62	6	0	-0.474912	-4.034681	3.301313
63	1	0	-0.958784	-4.461857	4.174535
64	6	0	-7.096041	0.459180	0.926686
65	1	0	-6.380544	0.933918	1.586099
66	6	0	-7.736997	-1.141275	-0.731065
67	1	0	-7.558064	-1.997870	-1.368825

68	6	0	8.711922	0.334125	-1.219719
69	1	0	9.282440	0.551758	-2.113303
70	6	0	3.232164	-1.059083	3.802364
71	1	0	2.137581	-1.010430	3.833353
72	1	0	3.559759	-0.357809	3.024848
73	1	0	3.604914	-0.672481	4.758845
74	6	0	-8.384071	0.948964	0.898483
75	1	0	-8.688405	1.765601	1.540152
76	6	0	3.912917	-4.668898	1.937970
77	1	0	5.003154	-4.513479	1.963930
78	1	0	3.685538	-5.271736	2.829410
79	6	0	-5.082079	-4.952104	-2.140333
80	1	0	-5.650807	-4.635334	-3.021865
81	1	0	-5.525234	-4.450478	-1.270901
82	1	0	-5.263354	-6.026454	-2.009134
83	6	0	3.553399	-5.477810	0.684336
84	1	0	2.481898	-5.704888	0.645673
85	1	0	4.090542	-6.433719	0.641770
86	1	0	3.799684	-4.932885	-0.235193
87	5	0	3.173208	-3.206150	2.143451
88	5	0	-3.158186	-3.051173	-2.498626
89	5	0	10.918618	0.978464	-0.072152
90	6	0	11.419644	1.512969	1.405283
91	6	0	11.609132	-0.429329	-0.589344
92	6	0	11.155125	2.281001	-1.055139
93	6	0	10.707294	2.512028	2.079867
94	6	0	12.642141	1.187675	2.002684
95	6	0	12.010824	-0.699406	-1.898945
96	6	0	11.691991	-1.530270	0.269228
97	6	0	12.465119	2.613372	-1.419681
98	6	0	10.200916	3.224411	-1.443377
99	6	0	11.119763	3.106391	3.268018
100	9	0	9.532683	2.955121	1.572852
101	6	0	13.098772	1.762184	3.187765
102	9	0	13.474757	0.284999	1.446241
103	6	0	12.509949	-1.932124	-2.318261
104	9	0	11.904370	0.239017	-2.867644
105	6	0	12.182673	-2.776783	-0.101407
106	9	0	11.270710	-1.416027	1.550386
107	6	0	12.812723	3.748331	-2.141211
108	9	0	13.483944	1.803481	-1.054303
109	6	0	10.503049	4.375879	-2.171384
110	9	0	8.893990	3.070080	-1.130486
111	6	0	12.333180	2.727237	3.829502
112	9	0	10.368253	4.046245	3.862108
113	9	0	14.279652	1.393808	3.709313
114	6	0	12.601509	-2.979970	-1.412034
115	9	0	12.886420	-2.115845	-3.593957
116	9	0	12.245297	-3.782651	0.785664
117	6	0	11.818059	4.642270	-2.525012
118	9	0	14.093324	3.993158	-2.462229
119	9	0	9.529016	5.231410	-2.522143
120	9	0	12.759001	3.286126	4.969026
121	9	0	13.074354	-4.172532	-1.796183
122	9	0	12.127180	5.744569	-3.220618
123	5	0	-10.865994	1.000685	0.233209
124	6	0	-11.800313	0.535758	-1.043758
125	6	0	-11.279890	0.345036	1.690861
126	6	0	-10.910075	2.647360	0.163780
127	6	0	-11.379686	0.773908	-2.357862
128	6	0	-13.115960	0.070352	-0.945789

129	6	0	-11.276456	1.021863	2.912043
130	6	0	-11.522708	-1.027820	1.802408
131	6	0	-12.085281	3.300767	0.553005
132	6	0	-9.956635	3.489255	-0.413561
133	6	0	-12.152088	0.526700	-3.488081
134	9	0	-10.143586	1.277795	-2.584303
135	6	0	-13.928393	-0.184036	-2.049497
136	9	0	-13.691371	-0.153905	0.252698
137	6	0	-11.549153	0.413094	4.136601
138	9	0	-10.968653	2.337519	2.978216
139	6	0	-11.799881	-1.679091	2.998627
140	9	0	-11.488175	-1.808002	0.696868
141	6	0	-12.297742	4.668048	0.430746
142	9	0	-13.104124	2.583254	1.077135
143	6	0	-10.124970	4.867383	-0.553550
144	9	0	-8.783038	3.004319	-0.879488
145	6	0	-13.445243	0.042160	-3.331971
146	9	0	-11.667095	0.765970	-4.716301
147	9	0	-15.179876	-0.639309	-1.880085
148	6	0	-11.817914	-0.948269	4.181855
149	9	0	-11.537469	1.130916	5.271267
150	9	0	-12.038198	-3.000134	3.023259
151	6	0	-11.303228	5.464301	-0.128493
152	9	0	-13.450736	5.223422	0.837093
153	9	0	-9.156628	5.616131	-1.106118
154	9	0	-14.214326	-0.199251	-4.400824
155	9	0	-12.078206	-1.552055	5.348511
156	9	0	-11.483683	6.785327	-0.258131

Table S33. Coordinates for Optimized Structure of **1Py-BCF**$^{\cdot-}$ (radical anion, no counter ion) in doublet state (D_1).

E(UB3LYP) = -6310.114919 Hartree.

opt freq UB3LYP/6-31G(d,p) scrf=(solvent=acetonitrile)

Center Number	Atomic Number	Atomic Type	Coordinates (Angstroms) X	Y	Z
1	7	0	3.600386	-2.225770	0.916324
2	7	0	-3.615831	-2.263816	-1.058826
3	7	0	9.403249	0.536446	-0.028783
4	7	0	-9.393426	0.453275	0.068423
5	6	0	1.419551	-2.559923	-0.152111
6	6	0	-0.775481	-2.770897	-1.321784
7	6	0	0.660233	-2.723709	-1.373434
8	6	0	-4.854589	-1.759069	-0.988332
9	1	0	-5.427934	-1.854379	-1.898389
10	6	0	-1.427018	-2.676119	-0.041882
11	6	0	0.783774	-2.928029	1.087716
12	6	0	-5.400908	-1.124454	0.131173
13	6	0	2.744923	-2.016941	-0.156476
14	6	0	4.839190	-1.716203	0.895550
15	1	0	5.442403	-1.986587	1.750392
16	6	0	-1.558508	-2.905445	-2.508658
17	6	0	-0.651351	-3.001222	1.134580
18	6	0	5.368583	-0.928070	-0.128752
19	6	0	-2.775714	-2.199557	0.045161

20	6	0	-6.760275	-0.599921	0.110363
21	6	0	3.211549	-1.157802	-1.196221
22	1	0	2.522856	-0.885126	-1.983357
23	6	0	1.280079	-2.919876	-2.635577
24	1	0	2.359614	-2.995726	-2.695203
25	6	0	4.474065	-0.629488	-1.194379
26	1	0	4.762671	0.053388	-1.985674
27	6	0	6.740095	-0.439853	-0.083165
28	6	0	-3.270214	-1.525970	1.199594
29	1	0	-2.606851	-1.399160	2.043445
30	6	0	-1.255508	-3.467162	2.330199
31	1	0	-2.328180	-3.621809	2.360684
32	6	0	-0.879596	-3.039363	-3.715396
33	1	0	-1.444766	-3.164726	-4.635185
34	6	0	1.575591	-3.211595	2.241998
35	6	0	-3.952717	-2.505432	-3.721974
36	1	0	-3.623640	-3.121555	-4.572050
37	1	0	-5.034819	-2.702037	-3.673730
38	6	0	-4.532864	-0.996830	1.249473
39	1	0	-4.856294	-0.487769	2.150306
40	6	0	3.809040	-2.491324	3.572856
41	1	0	3.487852	-3.131662	4.407136
42	1	0	4.908007	-2.569093	3.600750
43	6	0	0.525878	-3.074664	-3.781252
44	1	0	1.022062	-3.245345	-4.733371
45	6	0	7.513313	-0.414767	1.099833
46	1	0	7.109479	-0.745447	2.048108
47	6	0	-3.510575	-4.672212	-2.115280
48	1	0	-3.119750	-5.243490	-2.971473
49	1	0	-2.932965	-5.027147	-1.248103
50	6	0	8.807190	0.059813	1.090305
51	1	0	9.404019	0.060242	1.990641
52	6	0	7.393261	0.042901	-1.243207
53	1	0	6.903657	0.034194	-2.208571
54	6	0	-9.007204	-0.504688	-0.807418
55	1	0	-9.761429	-0.861769	-1.493494
56	6	0	0.908763	-3.630934	3.390211
57	1	0	1.483506	-3.881145	4.278087
58	6	0	-3.734158	-1.026020	-4.085724
59	1	0	-4.252391	-0.738270	-5.009475
60	1	0	-2.669486	-0.808398	-4.224450
61	1	0	-4.092114	-0.352263	-3.297036
62	6	0	-0.488838	-3.783667	3.433558
63	1	0	-0.967415	-4.165118	4.332032
64	6	0	-7.198022	0.378919	1.034985
65	1	0	-6.530753	0.784358	1.784636
66	6	0	-7.735200	-1.035626	-0.815527
67	1	0	-7.523314	-1.818508	-1.532615
68	6	0	8.686813	0.504623	-1.182142
69	1	0	9.199374	0.851169	-2.070479
70	6	0	3.403129	-1.043112	3.891362
71	1	0	2.312795	-0.944045	3.943653
72	1	0	3.748389	-0.343539	3.119556
73	1	0	3.811229	-0.690228	4.847226
74	6	0	-8.482623	0.866587	0.987346
75	1	0	-8.825007	1.610547	1.695248
76	6	0	3.863803	-4.643387	1.955146
77	1	0	4.961627	-4.537914	1.937307
78	1	0	3.650382	-5.245948	2.852120
79	6	0	-4.985853	-5.048459	-1.906364
80	1	0	-5.598955	-4.794144	-2.779057

81	1	0	-5.421920	-4.525582	-1.045519
82	1	0	-5.119342	-6.122693	-1.722668
83	6	0	3.427984	-5.439923	0.716348
84	1	0	2.346768	-5.619881	0.717290
85	1	0	3.921492	-6.418751	0.652318
86	1	0	3.660521	-4.903639	-0.211875
87	5	0	3.191267	-3.159243	2.200654
88	5	0	-3.163742	-3.080055	-2.403827
89	5	0	10.968587	0.957178	-0.100513
90	6	0	11.580905	1.255073	1.403939
91	6	0	11.608792	-0.356145	-0.874423
92	6	0	11.171671	2.396409	-0.883639
93	6	0	10.934886	2.145582	2.270229
94	6	0	12.832869	0.830053	1.860871
95	6	0	11.924173	-0.422088	-2.232680
96	6	0	11.725412	-1.578557	-0.204996
97	6	0	12.462662	2.772208	-1.272805
98	6	0	10.212109	3.395818	-1.062163
99	6	0	11.436101	2.545118	3.505032
100	9	0	9.740410	2.676330	1.918135
101	6	0	13.377555	1.209889	3.086535
102	9	0	13.610102	0.015557	1.118249
103	6	0	12.374335	-1.578085	-2.869917
104	9	0	11.776142	0.657158	-3.035822
105	6	0	12.170052	-2.755709	-0.795376
106	9	0	11.388512	-1.663165	1.103347
107	6	0	12.785991	4.002573	-1.830761
108	9	0	13.488840	1.908429	-1.100733
109	6	0	10.489969	4.644118	-1.621031
110	9	0	8.924061	3.206826	-0.696461
111	6	0	12.675369	2.071798	3.918984
112	9	0	10.744564	3.390607	4.285461
113	9	0	14.582724	0.753641	3.464428
114	6	0	12.503261	-2.755347	-2.145704
115	9	0	12.668056	-1.563154	-4.180566
116	9	0	12.270821	-3.888518	-0.080873
117	6	0	11.785583	4.952683	-2.009845
118	9	0	14.049533	4.284820	-2.188717
119	9	0	9.511313	5.551596	-1.774523
120	9	0	13.186089	2.444154	5.099642
121	9	0	12.930681	-3.877362	-2.740076
122	9	0	12.071196	6.147652	-2.544853
123	5	0	-10.921635	0.982432	0.200792
124	6	0	-11.793861	0.650367	-1.161440
125	6	0	-11.410336	0.183962	1.563512
126	6	0	-10.982070	2.629465	0.297570
127	6	0	-11.311908	1.020117	-2.423105
128	6	0	-13.109685	0.175626	-1.176121
129	6	0	-11.465308	0.732734	2.845960
130	6	0	-11.652754	-1.192848	1.524906
131	6	0	-12.179490	3.236004	0.694458
132	6	0	-10.009038	3.529895	-0.142919
133	6	0	-12.026548	0.884312	-3.609054
134	9	0	-10.071637	1.549957	-2.538896
135	6	0	-13.865369	0.029497	-2.338313
136	9	0	-13.743290	-0.167475	-0.035995
137	6	0	-11.790486	0.003619	3.989423
138	9	0	-11.165913	2.035274	3.059752
139	6	0	-11.981634	-1.961625	2.635329
140	9	0	-11.565831	-1.858346	0.349332
141	6	0	-12.396939	4.608000	0.705036

142	9	0	-13.217360	2.465778	1.092321
143	6	0	-10.181690	4.914772	-0.146844
144	9	0	-8.810997	3.101434	-0.600585
145	6	0	-13.321872	0.382891	-3.566588
146	9	0	-11.484279	1.246900	-4.782334
147	9	0	-15.120761	-0.443368	-2.276749
148	6	0	-12.055741	-1.355002	3.884662
149	9	0	-11.832847	0.603125	5.190677
150	9	0	-12.216711	-3.278508	2.515201
151	6	0	-11.383328	5.461149	0.280455
152	9	0	-13.572825	5.114871	1.110800
153	9	0	-9.194085	5.720193	-0.571915
154	9	0	-14.035882	0.246855	-4.691362
155	9	0	-12.366451	-2.074146	4.971471
156	9	0	-11.567923	6.788413	0.279872

Table S34. Coordinates for Optimized Structure of **1Py-BCF^{2-}** (dianion, no counter ions) in singlet state (S_1). Both open shell and closed shell optimization resulted same closed shell state.

E(UB3LYP) = -6310.228650 Hartree.

opt freq UB3LYP/6-31G(d,p) scrf=(solvent=acetonitrile) nosymm guess=(mix,always)

Center Number	Atomic Number	Atomic Type	Coordinates (Angstroms) X	Y	Z
1	7	0	3.548623	-2.444052	1.067320
2	7	0	-3.582460	-2.631333	-0.916528
3	7	0	9.264872	0.460134	-0.059903
4	7	0	-9.223327	0.458330	0.067540
5	6	0	1.415029	-2.982705	0.003684
6	6	0	-0.793734	-3.319517	-1.142376
7	6	0	0.633797	-3.282415	-1.202286
8	6	0	-4.774159	-2.038282	-0.884886
9	1	0	-5.339384	-2.139816	-1.799996
10	6	0	-1.442864	-3.114352	0.147235
11	6	0	0.787040	-3.307615	1.281104
12	6	0	-5.335366	-1.371170	0.217546
13	6	0	2.694005	-2.414246	-0.053656
14	6	0	4.755309	-1.883508	1.005927
15	1	0	5.337748	-2.006410	1.908454
16	6	0	-1.579773	-3.536367	-2.305415
17	6	0	-0.637924	-3.412646	1.335529
18	6	0	5.301424	-1.207991	-0.097489
19	6	0	-2.748316	-2.613128	0.222728
20	6	0	-6.638961	-0.773982	0.154494
21	6	0	3.185983	-1.680281	-1.195237
22	1	0	2.514640	-1.544049	-2.032010
23	6	0	1.246868	-3.622109	-2.429852
24	1	0	2.326817	-3.709421	-2.480503
25	6	0	4.414035	-1.099663	-1.226135
26	1	0	4.694675	-0.514578	-2.095079
27	6	0	6.629471	-0.664458	-0.072050
28	6	0	-3.283034	-1.948241	1.385237
29	1	0	-2.648506	-1.869919	2.257457
30	6	0	-1.224417	-3.877035	2.533072
31	1	0	-2.293306	-4.060687	2.565231
32	6	0	-0.907981	-3.800702	-3.503179

33	1	0	-1.482255	-3.984393	-4.408304
34	6	0	1.581883	-3.501284	2.441964
35	6	0	-3.942370	-3.068620	-3.554579
36	1	0	-3.659611	-3.775492	-4.349499
37	1	0	-5.036438	-3.179924	-3.495069
38	6	0	-4.503404	-1.349321	1.392013
39	1	0	-4.817749	-0.811773	2.279934
40	6	0	3.690151	-2.443051	3.742153
41	1	0	3.399106	-3.024558	4.629446
42	1	0	4.790529	-2.409095	3.805626
43	6	0	0.489695	-3.878458	-3.564139
44	1	0	0.985171	-4.148143	-4.494192
45	6	0	7.466828	-0.654559	1.082876
46	1	0	7.133375	-1.068950	2.025737
47	6	0	-3.658571	-5.122269	-1.776823
48	1	0	-3.312570	-5.790753	-2.581240
49	1	0	-3.108553	-5.447903	-0.880612
50	6	0	8.726442	-0.112234	1.052673
51	1	0	9.355899	-0.132109	1.930846
52	6	0	7.234436	-0.085866	-1.229278
53	1	0	6.715346	-0.062480	-2.178763
54	6	0	-8.635455	-0.049050	-1.051418
55	1	0	-9.205795	0.027854	-1.966079
56	6	0	0.932190	-3.915533	3.610669
57	1	0	1.518383	-4.095278	4.509132
58	6	0	-3.618338	-1.644870	-4.040769
59	1	0	-4.115908	-1.395881	-4.987537
60	1	0	-2.540717	-1.518295	-4.192691
61	1	0	-3.923506	-0.884199	-3.311079
62	6	0	-0.449890	-4.135858	3.655078
63	1	0	-0.918567	-4.510611	4.562264
64	6	0	-7.294905	-0.259313	1.313838
65	1	0	-6.838216	-0.332134	2.292452
66	6	0	-7.396987	-0.639142	-1.046203
67	1	0	-7.012802	-0.985471	-1.997245
68	6	0	8.498951	0.438369	-1.188803
69	1	0	8.956154	0.856063	-2.077301
70	6	0	3.142024	-1.015554	3.899425
71	1	0	2.045805	-1.016714	3.913945
72	1	0	3.447159	-0.370842	3.065557
73	1	0	3.481767	-0.527253	4.822514
74	6	0	-8.534123	0.318611	1.236592
75	1	0	-9.033066	0.684865	2.125327
76	6	0	4.006083	-4.721557	2.342494
77	1	0	5.090407	-4.516358	2.351660
78	1	0	3.811617	-5.263267	3.282427
79	6	0	-5.157512	-5.371124	-1.544923
80	1	0	-5.751095	-5.146702	-2.439427
81	1	0	-5.552301	-4.743336	-0.735983
82	1	0	-5.373288	-6.412745	-1.270641
83	6	0	3.696913	-5.660929	1.167124
84	1	0	2.634493	-5.930476	1.143981
85	1	0	4.270314	-6.597085	1.209637
86	1	0	3.925329	-5.188120	0.204137
87	5	0	3.186663	-3.292819	2.420550
88	5	0	-3.194560	-3.585489	-2.185346
89	5	0	10.791030	0.947165	-0.167946
90	6	0	11.454653	1.205719	1.325623
91	6	0	11.471894	-0.292786	-1.031187
92	6	0	10.914755	2.433044	-0.886468
93	6	0	10.803280	2.021882	2.258763

94	6	0	12.741475	0.820387	1.715526
95	6	0	11.725516	-0.279699	-2.403602
96	6	0	11.677158	-1.539045	-0.430755
97	6	0	12.174450	2.881425	-1.300180
98	6	0	9.910324	3.399187	-0.985439
99	6	0	11.333752	2.384413	3.493051
100	9	0	9.574743	2.515748	1.979316
101	6	0	13.315676	1.165111	2.938252
102	9	0	13.527803	0.080607	0.906106
103	6	0	12.196027	-1.380840	-3.118817
104	9	0	11.492868	0.829293	-3.145497
105	6	0	12.146476	-2.663865	-1.099210
106	9	0	11.409459	-1.704511	0.886121
107	6	0	12.428909	4.148797	-1.809044
108	9	0	13.241681	2.055248	-1.205818
109	6	0	10.118523	4.682819	-1.493057
110	9	0	8.644332	3.142998	-0.587337
111	6	0	12.607959	1.950877	3.838262
112	9	0	10.635367	3.158467	4.339857
113	9	0	14.554727	0.748305	3.248652
114	6	0	12.413534	-2.583924	-2.461800
115	9	0	12.425750	-1.288502	-4.439873
116	9	0	12.334856	-3.823780	-0.447106
117	6	0	11.385882	5.063680	-1.909431
118	9	0	13.667439	4.500006	-2.195121
119	9	0	9.099144	5.555460	-1.570689
120	9	0	13.146472	2.289500	5.017523
121	9	0	12.862797	-3.655267	-3.130869
122	9	0	11.604507	6.293871	-2.395673
123	5	0	-10.733412	1.001687	0.117117
124	6	0	-11.282537	1.415806	-1.387969
125	6	0	-11.519087	-0.276191	0.821260
126	6	0	-10.848744	2.425470	0.953097
127	6	0	-10.535206	2.271979	-2.206244
128	6	0	-12.557617	1.130356	-1.887323
129	6	0	-11.860510	-0.371240	2.171233
130	6	0	-11.735420	-1.454393	0.099579
131	6	0	-12.114679	2.888604	1.329950
132	6	0	-9.818586	3.338754	1.191930
133	6	0	-10.967271	2.762014	-3.434867
134	9	0	-9.306427	2.678678	-1.811343
135	6	0	-13.034935	1.604352	-3.108370
136	9	0	-13.427895	0.367265	-1.193643
137	6	0	-12.421037	-1.507598	2.754218
138	9	0	-11.632179	0.657180	3.022474
139	6	0	-12.292183	-2.610524	0.634187
140	9	0	-11.390053	-1.516448	-1.208169
141	6	0	-12.354544	4.118389	1.930043
142	9	0	-13.202899	2.117511	1.102811
143	6	0	-10.011764	4.583065	1.794504
144	9	0	-8.540422	3.065603	0.847489
145	6	0	-12.234951	2.424688	-3.892865
146	9	0	-10.180537	3.566940	-4.167740
147	9	0	-14.269301	1.279286	-3.527865
148	6	0	-12.643847	-2.637476	1.979499
149	9	0	-12.732638	-1.520438	4.061575
150	9	0	-12.484358	-3.699282	-0.129846
151	6	0	-11.287852	4.978889	2.168098
152	9	0	-13.601082	4.486083	2.272496
153	9	0	-8.968822	5.404393	2.005026
154	9	0	-12.679737	2.887390	-5.069054

155	9	0	-13.178771	-3.741219	2.520708
156	9	0	-11.491883	6.172141	2.744369

Table S35. Coordinates for Optimized Structure of **1Py-Me^{2+}** (no counter ions) in the ground state (S_0).

E(RB3LYP) = -1972.978260 Hartree.

opt freq RB3LYP/6-31G(d,p) scrf=(solvent=acetonitrile)

Center Number	Atomic Number	Atomic Type	Coordinates (Angstroms) X	Y	Z
1	7	0	3.747451	-0.677383	0.423210
2	7	0	-3.669849	0.672100	0.370446
3	7	0	-9.426955	-0.896882	-2.050390
4	6	0	-0.824425	1.153029	0.745625
5	6	0	0.900203	-1.120852	0.874656
6	6	0	0.611444	1.316882	0.707505
7	6	0	-2.747574	-0.316540	0.158573
8	6	0	1.440084	0.161262	0.578828
9	6	0	-0.537054	-1.284089	0.892266
10	6	0	-1.368412	-0.154448	0.630714
11	6	0	-3.118966	-1.427326	-0.626030
12	1	0	-2.371803	-2.167757	-0.872872
13	6	0	2.819172	0.270214	0.091789
14	6	0	-5.369280	-0.597251	-0.787762
15	6	0	1.130848	2.633799	0.873735
16	1	0	2.200783	2.786337	0.943119
17	6	0	-4.408247	-1.573635	-1.097635
18	1	0	-4.667652	-2.447505	-1.684742
19	6	0	3.195465	1.291658	-0.806279
20	1	0	2.443975	1.983836	-1.157363
21	6	0	1.776995	-2.220207	1.161527
22	6	0	-6.771818	-0.699331	-1.224017
23	6	0	-1.702484	2.276189	0.910675
24	6	0	-4.929938	0.514131	-0.068711
25	1	0	-5.594735	1.339067	0.141862
26	6	0	-1.117686	3.524753	1.032575
27	1	0	-1.746078	4.397017	1.186831
28	7	0	9.565930	0.752314	-1.937172
29	6	0	-1.059527	-2.562430	1.239073
30	1	0	-2.129563	-2.696332	1.341248
31	6	0	5.467061	0.503044	-0.798812
32	6	0	0.285065	3.704971	1.029956
33	1	0	0.699962	4.696974	1.185318
34	6	0	6.883560	0.590285	-1.191198
35	6	0	1.187409	-3.434534	1.473897
36	1	0	1.816204	-4.282683	1.728352
37	6	0	5.020375	-0.535484	0.016090
38	1	0	5.690004	-1.321484	0.335081
39	6	0	-4.210618	3.289454	0.683740
40	1	0	-5.268847	3.147331	0.949190
41	1	0	-3.915165	4.155777	1.293445
42	6	0	4.495757	1.412999	-1.251399
43	1	0	4.754974	2.220248	-1.926963
44	6	0	-7.120985	-1.347363	-2.423526
45	1	0	-6.369203	-1.771728	-3.076556
46	6	0	-0.214143	-3.606410	1.527223
47	1	0	-0.627611	-4.566231	1.824024
48	6	0	4.206161	-3.300454	0.684236

49	1	0	5.285631	-3.211492	0.883427
50	1	0	3.910257	-4.172947	1.283855
51	6	0	-3.494010	1.696512	2.790037
52	1	0	-2.827416	0.873202	3.087763
53	1	0	-3.135503	2.572636	3.350636
54	6	0	-7.821267	-0.162391	-0.456935
55	1	0	-7.636889	0.316972	0.495843
56	6	0	3.846821	-1.732127	2.844914
57	1	0	3.666666	-2.660856	3.407245
58	1	0	4.940594	-1.596111	2.859921
59	6	0	-8.441432	-1.431226	-2.810562
60	1	0	-8.747880	-1.913227	-3.729599
61	6	0	-9.126974	-0.271101	-0.888705
62	1	0	-9.959188	0.123940	-0.321718
63	6	0	7.270986	1.147964	-2.423980
64	1	0	6.541519	1.513354	-3.135108
65	6	0	7.910226	0.126039	-0.348579
66	1	0	7.697650	-0.281317	0.631339
67	6	0	8.603798	1.215967	-2.769769
68	1	0	8.938600	1.629529	-3.711926
69	6	0	3.195212	-0.565330	3.601357
70	1	0	2.109280	-0.691149	3.676400
71	1	0	3.372992	0.395098	3.101575
72	1	0	3.582944	-0.467853	4.623253
73	6	0	-4.117870	3.687392	-0.800103
74	1	0	-4.729989	4.566851	-1.034707
75	1	0	-4.451920	2.881214	-1.465396
76	1	0	-3.085615	3.920094	-1.083039
77	6	0	9.229322	0.214149	-0.741861
78	1	0	10.043984	-0.127676	-0.117579
79	5	0	-3.278430	2.043065	1.189462
80	6	0	3.993381	-3.638607	-0.800392
81	1	0	2.932400	-3.805460	-1.017596
82	1	0	4.537324	-4.541166	-1.104869
83	1	0	4.328937	-2.827750	-1.459271
84	6	0	-4.920903	1.364762	3.253803
85	1	0	-5.615756	2.189303	3.057512
86	1	0	-4.964940	1.156372	4.330219
87	1	0	-5.320945	0.477599	2.746152
88	5	0	3.374263	-2.023049	1.295810
89	6	0	-10.829738	-0.964640	-2.519530
90	1	0	-11.001559	-0.166599	-3.243488
91	1	0	-11.001369	-1.934505	-2.983505
92	1	0	-11.494181	-0.843051	-1.666717
93	6	0	10.984260	0.796590	-2.359935
94	1	0	11.198407	-0.075737	-2.979513
95	1	0	11.156508	1.709662	-2.927218
96	1	0	11.617499	0.789481	-1.475321

Table S36. Coordinates for Optimized Structure of **1Py-Me^{2+}** (with PF_6^-) in the ground state (S_0).

E(RB3LYP) = -3854.465220 Hartree.

opt freq RB3LYP/6-31G(d,p) scrf=(solvent=acetonitrile)

Center Number	Atomic Number	Atomic Type	Coordinates (Angstroms) X	Y	Z
1	7	0	4.279928	-1.731183	-1.439032
2	7	0	-2.624885	-1.968103	1.572648

3	7	0	-8.769339	-0.102994	1.102126
4	6	0	0.277370	-2.085390	1.425965
5	6	0	1.466240	-2.445243	-1.145866
6	6	0	1.702554	-1.923430	1.245913
7	6	0	-1.969591	-1.860870	0.375964
8	6	0	2.240227	-1.917091	-0.076393
9	6	0	0.037864	-2.589460	-0.967483
10	6	0	-0.549045	-2.212368	0.277122
11	6	0	-2.652878	-1.288236	-0.716107
12	1	0	-2.117535	-1.120235	-1.639490
13	6	0	3.552174	-1.322314	-0.356497
14	6	0	-4.664158	-1.118253	0.591084
15	6	0	2.512454	-1.847314	2.416726
16	1	0	3.590956	-1.815161	2.324155
17	6	0	-3.979992	-0.919924	-0.619541
18	1	0	-4.481475	-0.497010	-1.482710
19	6	0	4.045819	-0.251771	0.418170
20	1	0	3.429162	0.147954	1.210215
21	6	0	2.106505	-2.836967	-2.369397
22	6	0	-6.085246	-0.771993	0.765622
23	6	0	-0.311287	-2.133548	2.734023
24	6	0	-3.922255	-1.627483	1.656230
25	1	0	-4.362482	-1.741681	2.635749
26	6	0	0.542438	-2.022089	3.817557
27	1	0	0.139436	-2.082073	4.824351
28	7	0	9.980343	1.279027	-1.675309
29	6	0	-0.712313	-3.168084	-2.030430
30	1	0	-1.769156	-3.365260	-1.897246
31	6	0	6.076239	-0.219023	-0.865263
32	6	0	1.943066	-1.895249	3.665745
33	1	0	2.578959	-1.866119	4.546176
34	6	0	7.425815	0.292208	-1.159671
35	6	0	1.301426	-3.376082	-3.359784
36	1	0	1.752331	-3.714062	-4.288293
37	6	0	5.503512	-1.216123	-1.651372
38	1	0	6.015968	-1.605950	-2.519158
39	6	0	-2.544691	-1.926145	4.263537
40	1	0	-3.578097	-2.277033	4.403556
41	1	0	-2.009608	-2.383644	5.108588
42	6	0	5.286722	0.302468	0.176111
43	1	0	5.643208	1.116388	0.797609
44	6	0	-6.679524	0.271993	0.037174
45	1	0	-6.109545	0.871123	-0.661481
46	6	0	-0.090373	-3.555121	-3.192820
47	1	0	-0.670551	-4.026667	-3.981146
48	6	0	4.173888	-2.355044	-4.038404
49	1	0	5.261480	-2.457954	-4.177977
50	1	0	3.758729	-3.101179	-4.730810
51	6	0	-1.995270	-4.191771	2.832621
52	1	0	-1.497554	-4.564372	1.924679
53	1	0	-1.403360	-4.592722	3.668937
54	6	0	-6.908429	-1.472292	1.668264
55	1	0	-6.534631	-2.309913	2.243170
56	6	0	4.399305	-4.271402	-2.159014
57	1	0	4.084500	-4.980021	-2.940079
58	1	0	5.491900	-4.183484	-2.275859
59	6	0	-8.012143	0.586762	0.218030
60	1	0	-8.498909	1.384923	-0.323711
61	6	0	-8.232057	-1.119514	1.817746
62	1	0	-8.897199	-1.636024	2.497424
63	6	0	7.783272	1.609347	-0.825567

64	1	0	7.083322	2.294808	-0.371546
65	6	0	8.412855	-0.508972	-1.764914
66	1	0	8.227076	-1.544036	-2.021898
67	6	0	9.055361	2.072376	-1.086840
68	1	0	9.357606	3.078726	-0.830596
69	6	0	4.099223	-4.888664	-0.785242
70	1	0	3.026238	-5.065171	-0.649715
71	1	0	4.422465	-4.235164	0.034639
72	1	0	4.607774	-5.850791	-0.644006
73	6	0	-2.509189	-0.397451	4.438980
74	1	0	-2.934058	-0.078192	5.398580
75	1	0	-3.073049	0.121539	3.653666
76	1	0	-1.482942	-0.017805	4.391068
77	6	0	9.667726	0.004900	-2.014095
78	1	0	10.454365	-0.578947	-2.473829
79	5	0	-1.864929	-2.546750	2.910373
80	6	0	3.734615	-0.959292	-4.510353
81	1	0	2.645822	-0.849832	-4.453340
82	1	0	4.031766	-0.755983	-5.546564
83	1	0	4.166916	-0.161639	-3.893169
84	6	0	-3.408991	-4.790556	2.897502
85	1	0	-3.924376	-4.520161	3.826213
86	1	0	-3.394027	-5.886588	2.845555
87	1	0	-4.038440	-4.445762	2.067032
88	5	0	3.713579	-2.822895	-2.532448
89	6	0	-10.204552	0.227116	1.268303
90	1	0	-10.448945	0.197103	2.329397
91	1	0	-10.383401	1.219547	0.863957
92	1	0	-10.801048	-0.508574	0.726568
93	6	0	11.325815	1.809356	-1.987429
94	1	0	11.355829	2.113719	-3.034944
95	1	0	11.525605	2.665159	-1.346098
96	1	0	12.065737	1.031980	-1.803325
97	15	0	-10.285793	3.048715	-1.865345
98	9	0	-11.903588	3.196232	-1.750660
99	9	0	-10.421158	1.419624	-1.702774
100	9	0	-10.390063	2.901772	-3.483107
101	9	0	-8.652069	2.879891	-1.949388
102	9	0	-10.159848	3.171630	-0.225385
103	9	0	-10.127307	4.663921	-1.996340
104	15	0	6.491708	4.258519	1.702964
105	9	0	7.407021	4.760051	2.952211
106	9	0	5.181650	4.177646	2.667327
107	9	0	5.589528	3.724081	0.433417
108	9	0	7.806679	4.315417	0.715502
109	9	0	6.852240	2.694356	2.063202
110	9	0	6.134242	5.798350	1.313032

Table S37. Coordinates for Optimized Structure of **1Py-Me^{2+}** (with PF_6^-) in the first triplet state (T_1).

E(UB3LYP) = -3854.434518 Hartree.

opt freq UB3LYP/6-31G(d,p) scrf=(solvent=acetonitrile)

Center Number	Atomic Number	Atomic Type	Coordinates (Angstroms) X	Y	Z
1	7	0	4.095940	-1.718769	-1.565563
2	7	0	-2.538456	-1.719320	1.826262

3	7	0	-8.600676	0.431444	1.394024
4	6	0	0.323982	-2.073278	1.598424
5	6	0	1.381510	-2.599933	-1.030740
6	6	0	1.729544	-1.972443	1.345481
7	6	0	-1.946396	-1.835484	0.580328
8	6	0	2.212269	-1.965049	-0.032067
9	6	0	-0.014100	-2.770076	-0.757507
10	6	0	-0.572493	-2.231573	0.476248
11	6	0	-2.699060	-1.441499	-0.557325
12	1	0	-2.222639	-1.458679	-1.527017
13	6	0	3.461401	-1.358320	-0.391170
14	6	0	-4.601250	-0.927946	0.829509
15	6	0	2.598904	-1.969144	2.447722
16	1	0	3.671307	-1.979314	2.293817
17	6	0	-3.992839	-0.992763	-0.444705
18	1	0	-4.533635	-0.701619	-1.337651
19	6	0	4.041506	-0.319396	0.386276
20	1	0	3.504523	0.040588	1.252088
21	6	0	1.959540	-3.063938	-2.235224
22	6	0	-5.977431	-0.465684	1.028076
23	6	0	-0.200237	-2.055039	2.912027
24	6	0	-3.806140	-1.294577	1.919353
25	1	0	-4.185115	-1.221224	2.927679
26	6	0	0.725702	-2.019896	3.974554
27	1	0	0.360158	-2.028832	4.996733
28	7	0	9.696090	1.413451	-2.265335
29	6	0	-0.782846	-3.521256	-1.659120
30	1	0	-1.823188	-3.731540	-1.439797
31	6	0	5.922729	-0.193614	-1.109469
32	6	0	2.097832	-1.996762	3.752188
33	1	0	2.789588	-2.002416	4.589141
34	6	0	7.217622	0.353107	-1.522504
35	6	0	1.129052	-3.799759	-3.107539
36	1	0	1.550202	-4.198981	-4.025294
37	6	0	5.279415	-1.168889	-1.873415
38	1	0	5.707978	-1.516458	-2.802691
39	6	0	-2.295568	-1.369309	4.481903
40	1	0	-3.353534	-1.566899	4.708790
41	1	0	-1.770676	-1.784471	5.354248
42	6	0	5.237754	0.262000	0.042831
43	1	0	5.647338	1.050819	0.662936
44	6	0	-6.612401	0.377036	0.093955
45	1	0	-6.097817	0.731684	-0.789471
46	6	0	-0.208525	-4.043523	-2.821833
47	1	0	-0.815867	-4.637175	-3.498314
48	6	0	3.696350	-2.305033	-4.140054
49	1	0	4.759610	-2.280798	-4.424229
50	1	0	3.270416	-3.076178	-4.797061
51	6	0	-2.104737	-3.844203	3.319293
52	1	0	-1.719911	-4.369329	2.432330
53	1	0	-1.502802	-4.216619	4.160771
54	6	0	-6.739086	-0.840949	2.156868
55	1	0	-6.346898	-1.512428	2.909563
56	6	0	4.390086	-4.220406	-2.365056
57	1	0	4.035362	-4.956942	-3.100737
58	1	0	5.435874	-4.014308	-2.643271
59	6	0	-7.906962	0.807998	0.293080
60	1	0	-8.416232	1.456230	-0.405249
61	6	0	-8.027278	-0.383828	2.313774
62	1	0	-8.640471	-0.657543	3.162486
63	6	0	7.638545	1.625256	-1.087774

64	1	0	7.017893	2.252375	-0.466084
65	6	0	8.111276	-0.361679	-2.350772
66	1	0	7.886579	-1.360176	-2.702949
67	6	0	8.865544	2.124243	-1.465238
68	1	0	9.206579	3.096815	-1.136962
69	6	0	4.360187	-4.856041	-0.968393
70	1	0	3.341304	-5.132903	-0.674458
71	1	0	4.740748	-4.170258	-0.201554
72	1	0	4.972656	-5.764885	-0.915477
73	6	0	-2.066456	0.152064	4.452923
74	1	0	-2.401491	0.641191	5.375681
75	1	0	-2.601788	0.632509	3.624547
76	1	0	-1.004939	0.389889	4.321826
77	6	0	9.323235	0.184465	-2.705355
78	1	0	10.034020	-0.336932	-3.333346
79	5	0	-1.779200	-2.222028	3.182422
80	6	0	3.051468	-0.953865	-4.488205
81	1	0	1.973650	-0.966703	-4.289375
82	1	0	3.187182	-0.687634	-5.543608
83	1	0	3.475127	-0.134284	-3.894784
84	6	0	-3.568899	-4.248765	3.540806
85	1	0	-3.979242	-3.811244	4.457859
86	1	0	-3.681472	-5.337087	3.624679
87	1	0	-4.213217	-3.929630	2.712016
88	5	0	3.508925	-2.842910	-2.595568
89	6	0	-10.000634	0.873445	1.580813
90	1	0	-10.147081	1.139537	2.627051
91	1	0	-10.184726	1.732070	0.940692
92	1	0	-10.671277	0.057127	1.306966
93	6	0	11.001898	1.966122	-2.681842
94	1	0	10.976279	2.183266	-3.750767
95	1	0	11.192165	2.880606	-2.124711
96	1	0	11.784035	1.237396	-2.468522
97	15	0	-10.293341	2.706845	-2.218556
98	9	0	-11.886491	2.983064	-2.022003
99	9	0	-10.473387	1.204295	-1.580973
100	9	0	-10.557321	2.111401	-3.710801
101	9	0	-8.682655	2.419238	-2.382808
102	9	0	-10.006968	3.284824	-0.700876
103	9	0	-10.088651	4.204281	-2.825295
104	15	0	6.700846	4.044405	1.823475
105	9	0	7.799168	4.459024	2.951845
106	9	0	5.561194	3.856007	2.972412
107	9	0	5.611694	3.601556	0.670657
108	9	0	7.841877	4.213533	0.649645
109	9	0	7.124553	2.465020	1.985208
110	9	0	6.274528	5.604287	1.629764

Table S38. Coordinates for Optimized Structure of **1Py-Me⁺** (radical cation, no counter ion) in doublet state (D_1).

E(UB3LYP) = -1973.114921 Hartree.

opt freq UB3LYP/6-31G(d,p) scrf=(solvent=acetonitrile)

Center Number	Atomic Number	Atomic Type	Coordinates (Angstroms) X	Y	Z

1	7	0	3.711803	-0.712177	0.389078
2	7	0	-3.639902	0.679726	0.397005
3	7	0	-9.285952	-0.839715	-2.362783
4	6	0	-0.835900	1.140119	0.951586
5	6	0	0.906534	-1.141481	1.026318
6	6	0	0.591624	1.307327	0.926806
7	6	0	-2.716805	-0.352839	0.297759
8	6	0	1.444973	0.158503	0.724746
9	6	0	-0.521651	-1.305196	1.075929
10	6	0	-1.382251	-0.180400	0.793469
11	6	0	-3.114588	-1.514374	-0.424257
12	1	0	-2.382450	-2.291657	-0.591534
13	6	0	2.774640	0.293178	0.204456
14	6	0	-5.315626	-0.592740	-0.816958
15	6	0	1.108233	2.604986	1.183010
16	1	0	2.178490	2.747089	1.275053
17	6	0	-4.366557	-1.642131	-0.964233
18	1	0	-4.600719	-2.526485	-1.545559
19	6	0	3.163184	1.399063	-0.607244
20	1	0	2.415054	2.135269	-0.864717
21	6	0	1.785399	-2.240394	1.271633
22	6	0	-6.665891	-0.664929	-1.339823
23	6	0	-1.712832	2.253302	1.130892
24	6	0	-4.859118	0.541771	-0.131246
25	1	0	-5.513559	1.383376	0.038338
26	6	0	-1.132902	3.501297	1.328831
27	1	0	-1.769910	4.366230	1.492303
28	7	0	9.444841	0.814973	-2.189582
29	6	0	-1.034430	-2.565769	1.475692
30	1	0	-2.102340	-2.688982	1.614039
31	6	0	5.407853	0.528216	-0.832073
32	6	0	0.263049	3.677232	1.380630
33	1	0	0.679428	4.657184	1.598929
34	6	0	6.780533	0.613371	-1.290771
35	6	0	1.206938	-3.454875	1.628891
36	1	0	1.847116	-4.304155	1.851607
37	6	0	4.950591	-0.569612	-0.091619
38	1	0	5.615471	-1.383444	0.158279
39	6	0	-4.196055	3.289234	0.776082
40	1	0	-5.270999	3.143900	0.963753
41	1	0	-3.938376	4.129174	1.438139
42	6	0	4.429548	1.523868	-1.110906
43	1	0	4.657108	2.362781	-1.758275
44	6	0	-7.240452	-1.898015	-1.748588
45	1	0	-6.698256	-2.829369	-1.653890
46	6	0	-0.184837	-3.616409	1.755223
47	1	0	-0.593837	-4.566617	2.088528
48	6	0	4.134912	-3.351676	0.549272
49	1	0	5.230643	-3.291203	0.650105
50	1	0	3.873912	-4.235654	1.148751
51	6	0	-3.631268	1.612581	2.859542
52	1	0	-2.981295	0.782657	3.175867
53	1	0	-3.320054	2.469819	3.475966
54	6	0	-7.500650	0.474886	-1.484930
55	1	0	-7.154867	1.467615	-1.229481
56	6	0	4.009942	-1.828510	2.767255
57	1	0	3.856175	-2.764097	3.327299
58	1	0	5.104523	-1.719209	2.688565
59	6	0	-8.518546	-1.957123	-2.243952
60	1	0	-8.977177	-2.887968	-2.551940
61	6	0	-8.772757	0.363802	-1.985862

62	1	0	-9.420380	1.221417	-2.112665
63	6	0	7.324034	1.826574	-1.789968
64	1	0	6.738605	2.735760	-1.818494
65	6	0	7.671126	-0.494020	-1.279165
66	1	0	7.356146	-1.472851	-0.943172
67	6	0	8.624751	1.899130	-2.222397
68	1	0	9.057748	2.817168	-2.597693
69	6	0	3.459131	-0.662829	3.601888
70	1	0	2.378362	-0.757710	3.757533
71	1	0	3.626975	0.303007	3.109607
72	1	0	3.926988	-0.601569	4.593149
73	6	0	-4.004326	3.747244	-0.680690
74	1	0	-4.596733	4.638842	-0.922255
75	1	0	-4.293767	2.968989	-1.398353
76	1	0	-2.954477	3.985158	-0.883989
77	6	0	8.962812	-0.370571	-1.721452
78	1	0	9.653612	-1.203828	-1.728363
79	5	0	-3.304547	2.012721	1.288343
80	6	0	3.782753	-3.641831	-0.918846
81	1	0	2.702792	-3.781653	-1.042780
82	1	0	4.276891	-4.544315	-1.300460
83	1	0	4.073423	-2.815444	-1.579827
84	6	0	-5.084428	1.256192	3.210097
85	1	0	-5.771347	2.082066	2.991128
86	1	0	-5.205934	1.010838	4.273184
87	1	0	-5.439166	0.385670	2.643762
88	5	0	3.391881	-2.066940	1.259213
89	6	0	-10.635109	-0.924322	-2.949931
90	1	0	-10.570047	-0.856285	-4.038140
91	1	0	-11.089164	-1.873210	-2.667006
92	1	0	-11.242121	-0.105717	-2.566329
93	6	0	10.825982	0.895779	-2.697450
94	1	0	10.879482	0.451854	-3.693896
95	1	0	11.126264	1.940885	-2.745833
96	1	0	11.490174	0.360472	-2.018919

Table S39. Coordinates for Optimized Structure of **1Py-Me** (neutral) in singlet state (S_1). Both open shell and closed shell optimization resulted the same closed shell state.

E(UB3LYP) = -1973.239492 Hartree.

opt freq UB3LYP/6-31G(d,p) scrf=(solvent=acetonitrile) nosymm guess=(mix,always)

Center Number	Atomic Number	Atomic Type	Coordinates (Angstroms) X	Y	Z
1	7	0	3.672340	-0.600600	0.499836
2	7	0	-3.614275	0.712455	0.272758
3	7	0	-9.097844	-1.404423	-2.447169
4	6	0	-0.865198	1.331693	0.881344
5	6	0	0.922352	-0.884369	1.382184
6	6	0	0.551727	1.511577	0.833384
7	6	0	-2.680752	-0.322469	0.488400
8	6	0	1.440641	0.346735	0.797775
9	6	0	-0.493817	-1.044817	1.496191
10	6	0	-1.393776	-0.023494	0.956898
11	6	0	-3.093786	-1.641552	0.082019
12	1	0	-2.378967	-2.446920	0.179729

13	6	0	2.720503	0.400933	0.227764
14	6	0	-5.242556	-0.820476	-0.702497
15	6	0	1.049059	2.833014	0.902954
16	1	0	2.118228	2.998026	0.977477
17	6	0	-4.301186	-1.889076	-0.490870
18	1	0	-4.522592	-2.895751	-0.826779
19	6	0	3.116031	1.397810	-0.736330
20	1	0	2.372089	2.116701	-1.050839
21	6	0	1.814400	-1.900531	1.816611
22	6	0	-6.533204	-1.000871	-1.285223
23	6	0	-1.754939	2.438412	0.846396
24	6	0	-4.790351	0.448661	-0.284429
25	1	0	-5.436183	1.309627	-0.373177
26	6	0	-1.195873	3.719420	0.854858
27	1	0	-1.850428	4.587538	0.845865
28	7	0	9.302286	0.418578	-2.542593
29	6	0	-0.981213	-2.168351	2.199338
30	1	0	-2.045840	-2.259051	2.386267
31	6	0	5.335204	0.434551	-0.957274
32	6	0	0.189533	3.921231	0.916640
33	1	0	0.594954	4.928011	0.984598
34	6	0	6.663983	0.416409	-1.478447
35	6	0	1.263055	-3.010049	2.466573
36	1	0	1.922592	-3.792706	2.833949
37	6	0	4.877794	-0.547574	-0.055551
38	1	0	5.537146	-1.343864	0.259744
39	6	0	-4.204601	3.339510	0.112926
40	1	0	-5.291504	3.212409	0.236154
41	1	0	-4.003230	4.293200	0.623666
42	6	0	4.347003	1.420721	-1.310447
43	1	0	4.555649	2.166691	-2.068901
44	6	0	-7.051678	-2.299559	-1.599672
45	1	0	-6.483704	-3.194521	-1.384055
46	6	0	-0.114138	-3.133819	2.688865
47	1	0	-0.506671	-3.981433	3.245596
48	6	0	4.061259	-3.187484	1.078526
49	1	0	5.163033	-3.149091	1.064698
50	1	0	3.840732	-3.951463	1.838387
51	6	0	-3.807562	2.092500	2.510803
52	1	0	-3.183446	1.349348	3.030109
53	1	0	-3.550498	3.056263	2.977841
54	6	0	-7.419026	0.078565	-1.611533
55	1	0	-7.141095	1.109571	-1.436927
56	6	0	4.182244	-1.304081	3.000691
57	1	0	4.064751	-2.120432	3.731352
58	1	0	5.267091	-1.249278	2.806713
59	6	0	-8.286843	-2.466203	-2.159530
60	1	0	-8.685868	-3.444975	-2.394183
61	6	0	-8.644808	-0.141618	-2.170220
62	1	0	-9.316472	0.668631	-2.424603
63	6	0	7.159477	1.454251	-2.334292
64	1	0	6.542211	2.302526	-2.598291
65	6	0	7.616222	-0.619520	-1.202282
66	1	0	7.367444	-1.461581	-0.570104
67	6	0	8.430247	1.430125	-2.835134
68	1	0	8.809700	2.212278	-3.480618
69	6	0	3.736253	0.008897	3.661376
70	1	0	2.669848	-0.015896	3.914400
71	1	0	3.884373	0.866132	2.993367
72	1	0	4.286529	0.224165	4.587287
73	6	0	-3.895450	3.520747	-1.383760

74	1	0	-4.468984	4.338933	-1.838793
75	1	0	-4.120048	2.614756	-1.960775
76	1	0	-2.832971	3.736858	-1.541326
77	6	0	8.875750	-0.596768	-1.727971
78	1	0	9.597903	-1.378250	-1.527904
79	5	0	-3.352768	2.191911	0.922096
80	6	0	3.566131	-3.702280	-0.282659
81	1	0	2.476613	-3.823459	-0.282793
82	1	0	4.004147	-4.671310	-0.555819
83	1	0	3.807342	-3.002556	-1.092814
84	6	0	-5.283264	1.782552	2.807705
85	1	0	-5.952388	2.541126	2.384155
86	1	0	-5.492581	1.736180	3.884847
87	1	0	-5.588763	0.816781	2.385426
88	5	0	3.414128	-1.771521	1.619574
89	6	0	-10.394216	-1.598343	-3.107061
90	1	0	-10.305861	-1.424640	-4.183136
91	1	0	-10.734346	-2.618689	-2.933355
92	1	0	-11.123646	-0.904719	-2.686863
93	6	0	10.637412	0.374973	-3.150208
94	1	0	10.618948	-0.207158	-4.075929
95	1	0	10.966111	1.390773	-3.368983
96	1	0	11.338040	-0.081047	-2.450283

Table S40. Coordinates for Optimized Structure of **1Py-Me**$^{\cdot -}$ (radical anion, no counter ion) in doublet state (D_1).

E(UB3LYP) = -1973.329998 Hartree.

opt freq UB3LYP/6-31G(d,p) scrf=(solvent=acetonitrile)

Center Number	Atomic Number	Atomic Type	Coordinates (Angstroms) X	Y	Z
1	7	0	3.715446	-0.723350	0.353260
2	7	0	-3.651366	0.679510	0.400633
3	7	0	-9.356407	-0.831799	-2.362598
4	6	0	-0.841475	1.136916	0.970940
5	6	0	0.907858	-1.146562	1.035653
6	6	0	0.585995	1.301916	0.942144
7	6	0	-2.728018	-0.365549	0.337200
8	6	0	1.453256	0.155905	0.720691
9	6	0	-0.519273	-1.306589	1.106694
10	6	0	-1.397806	-0.187445	0.812732
11	6	0	-3.161772	-1.545094	-0.352826
12	1	0	-2.444259	-2.343417	-0.489261
13	6	0	2.775089	0.295337	0.209452
14	6	0	-5.366131	-0.601777	-0.791399
15	6	0	1.098356	2.597114	1.218442
16	1	0	2.170043	2.733869	1.312939
17	6	0	-4.406744	-1.669291	-0.896808
18	1	0	-4.647500	-2.573619	-1.445270
19	6	0	3.193946	1.422349	-0.574593
20	1	0	2.453335	2.174118	-0.813221
21	6	0	1.783371	-2.250354	1.267283
22	6	0	-6.693649	-0.666567	-1.312559
23	6	0	-1.714448	2.252798	1.149422
24	6	0	-4.871368	0.544577	-0.129876

25	1	0	-5.504202	1.408754	0.002164
26	6	0	-1.136566	3.504953	1.349409
27	1	0	-1.776191	4.371041	1.503244
28	7	0	9.528086	0.854860	-2.143226
29	6	0	-1.023360	-2.558526	1.543031
30	1	0	-2.090606	-2.671957	1.701585
31	6	0	5.453913	0.545152	-0.820625
32	6	0	0.258083	3.677233	1.414705
33	1	0	0.678944	4.654713	1.641156
34	6	0	6.810052	0.636902	-1.257094
35	6	0	1.210778	-3.463850	1.648390
36	1	0	1.855493	-4.315584	1.853986
37	6	0	4.958054	-0.576234	-0.120825
38	1	0	5.602846	-1.419090	0.081361
39	6	0	-4.191108	3.300175	0.765854
40	1	0	-5.269432	3.162728	0.942538
41	1	0	-3.931374	4.140294	1.427909
42	6	0	4.456241	1.551563	-1.073792
43	1	0	4.685185	2.410177	-1.695989
44	6	0	-7.228678	-1.856545	-1.928283
45	1	0	-6.634250	-2.759436	-1.997209
46	6	0	-0.176540	-3.616570	1.816494
47	1	0	-0.585071	-4.559881	2.172815
48	6	0	4.068343	-3.379109	0.386954
49	1	0	5.169277	-3.354681	0.440993
50	1	0	3.801922	-4.284108	0.952952
51	6	0	-3.658534	1.630194	2.858391
52	1	0	-3.012244	0.802093	3.187815
53	1	0	-3.354053	2.491280	3.474471
54	6	0	-7.625459	0.436191	-1.282366
55	1	0	-7.349292	1.390270	-0.849941
56	6	0	4.087384	-1.949795	2.669210
57	1	0	3.926498	-2.900584	3.202796
58	1	0	5.181702	-1.870758	2.551392
59	6	0	-8.493483	-1.912763	-2.427189
60	1	0	-8.896922	-2.807321	-2.886177
61	6	0	-8.881676	0.339271	-1.794187
62	1	0	-9.579725	1.167502	-1.777254
63	6	0	7.326817	1.794080	-1.946362
64	1	0	6.694142	2.651794	-2.139706
65	6	0	7.792837	-0.402230	-1.056892
66	1	0	7.537772	-1.322306	-0.545114
67	6	0	8.618627	1.875193	-2.366322
68	1	0	9.007573	2.744693	-2.882345
69	6	0	3.609661	-0.799846	3.568854
70	1	0	2.531977	-0.865308	3.759204
71	1	0	3.789592	0.176105	3.101650
72	1	0	4.113263	-0.786861	4.545387
73	6	0	-3.985542	3.755792	-0.689608
74	1	0	-4.564171	4.655209	-0.939945
75	1	0	-4.279961	2.977706	-1.404985
76	1	0	-2.930761	3.979241	-0.884504
77	6	0	9.075125	-0.282183	-1.493470
78	1	0	9.812482	-1.062242	-1.347270
79	5	0	-3.311930	2.010416	1.282475
80	6	0	3.650953	-3.586177	-1.077766
81	1	0	2.562206	-3.681740	-1.164449
82	1	0	4.095731	-4.484803	-1.526207
83	1	0	3.946428	-2.735741	-1.705005
84	6	0	-5.114729	1.275520	3.199450
85	1	0	-5.799565	2.100963	2.969839

86	1	0	-5.250947	1.033005	4.262369
87	1	0	-5.463698	0.405911	2.628428
88	5	0	3.393719	-2.097655	1.179226
89	6	0	-10.648217	-0.864810	-3.036572
90	1	0	-10.567424	-0.555504	-4.086097
91	1	0	-11.053330	-1.877994	-2.999556
92	1	0	-11.343511	-0.195462	-2.526160
93	6	0	10.860259	0.898388	-2.732421
94	1	0	10.868223	0.490695	-3.750995
95	1	0	11.211315	1.931768	-2.765567
96	1	0	11.550739	0.318178	-2.117117

Table S41. Coordinates for Optimized Structure of **1Py-Me**$^{2-}$ (dianion, no counter ions) in closed shell singlet state (S_1).

E(RB3LYP) = -1973.394289 Hartree.

opt freq RB3LYP/6-31G(d,p) scrf=(solvent=acetonitrile)

Center Number	Atomic Number	Atomic Type	Coordinates (Angstroms) X	Y	Z
1	7	0	3.751612	-0.745929	0.297170
2	7	0	-3.688137	0.687662	0.377409
3	7	0	-9.593990	-0.854754	-2.003793
4	6	0	-0.829553	1.126883	0.822482
5	6	0	0.897690	-1.158472	0.882883
6	6	0	0.606504	1.277939	0.784119
7	6	0	-2.770685	-0.352387	0.266446
8	6	0	1.461651	0.129725	0.582214
9	6	0	-0.537466	-1.304215	0.964211
10	6	0	-1.405911	-0.182218	0.692669
11	6	0	-3.229099	-1.509534	-0.438581
12	1	0	-2.511812	-2.297753	-0.632377
13	6	0	2.814686	0.261978	0.105779
14	6	0	-5.479541	-0.588185	-0.735622
15	6	0	1.127881	2.580847	1.029485
16	1	0	2.201293	2.710034	1.114300
17	6	0	-4.499630	-1.637711	-0.923422
18	1	0	-4.755050	-2.529895	-1.486446
19	6	0	3.256508	1.363943	-0.694651
20	1	0	2.515893	2.093979	-0.998002
21	6	0	1.763129	-2.278817	1.108142
22	6	0	-6.822261	-0.659159	-1.160236
23	6	0	-1.691955	2.258127	1.000605
24	6	0	-4.933973	0.561691	-0.089435
25	1	0	-5.552315	1.431656	0.069211
26	6	0	-1.102081	3.506167	1.166320
27	1	0	-1.730790	4.380842	1.318570
28	7	0	9.744346	0.945664	-1.746284
29	6	0	-1.048967	-2.562358	1.387974
30	1	0	-2.116661	-2.665182	1.550922
31	6	0	5.560135	0.536989	-0.780727
32	6	0	0.299527	3.668592	1.210135
33	1	0	0.729563	4.647218	1.415812
34	6	0	6.927534	0.656602	-1.099937
35	6	0	1.179504	-3.487300	1.476868
36	1	0	1.815834	-4.347073	1.677592

37	6	0	5.016563	-0.598154	-0.108790
38	1	0	5.647395	-1.447775	0.109530
39	6	0	-4.172876	3.326738	0.706936
40	1	0	-5.243470	3.206951	0.935146
41	1	0	-3.871552	4.176382	1.338900
42	6	0	4.542019	1.505353	-1.133242
43	1	0	4.784213	2.343455	-1.779429
44	6	0	-7.388991	-1.833139	-1.806254
45	1	0	-6.780614	-2.717229	-1.964916
46	6	0	-0.213518	-3.629024	1.646095
47	1	0	-0.629093	-4.571869	1.996884
48	6	0	4.035072	-3.405675	0.195928
49	1	0	5.135184	-3.409263	0.266401
50	1	0	3.736098	-4.334051	0.705636
51	6	0	-3.559930	1.698908	2.809113
52	1	0	-2.916989	0.862343	3.123571
53	1	0	-3.203798	2.565176	3.389792
54	6	0	-7.788106	0.420699	-1.015803
55	1	0	-7.504199	1.357805	-0.548645
56	6	0	4.048903	-2.100568	2.553268
57	1	0	3.850380	-3.070259	3.038103
58	1	0	5.147106	-2.046541	2.461377
59	6	0	-8.679541	-1.899609	-2.212348
60	1	0	-9.093646	-2.782883	-2.686822
61	6	0	-9.071224	0.318993	-1.436161
62	1	0	-9.783492	1.129247	-1.323229
63	6	0	7.480653	1.802417	-1.805650
64	1	0	6.840144	2.631523	-2.087619
65	6	0	7.937378	-0.339859	-0.771870
66	1	0	7.669783	-1.237632	-0.224480
67	6	0	8.793985	1.910977	-2.118310
68	1	0	9.197159	2.773505	-2.638386
69	6	0	3.584109	-0.980375	3.495882
70	1	0	2.502023	-1.028709	3.665936
71	1	0	3.793557	0.009520	3.072212
72	1	0	4.071172	-1.019356	4.480388
73	6	0	-4.030180	3.750828	-0.765293
74	1	0	-4.603322	4.656542	-1.007137
75	1	0	-4.373816	2.963175	-1.447177
76	1	0	-2.981837	3.949614	-1.014694
77	6	0	9.242816	-0.197784	-1.101411
78	1	0	9.988702	-0.943446	-0.847470
79	5	0	-3.287136	2.032474	1.206166
80	6	0	3.638191	-3.519866	-1.284414
81	1	0	2.549006	-3.584288	-1.393947
82	1	0	4.069370	-4.401788	-1.777969
83	1	0	3.964718	-2.641269	-1.854728
84	6	0	-5.003732	1.386471	3.234343
85	1	0	-5.680009	2.224418	3.024809
86	1	0	-5.089870	1.166897	4.307822
87	1	0	-5.402823	0.516707	2.697674
88	5	0	3.378781	-2.148696	1.044940
89	6	0	-10.764892	-0.761042	-2.860700
90	1	0	-10.533469	-0.367228	-3.863564
91	1	0	-11.213258	-1.751535	-2.976920
92	1	0	-11.505775	-0.103705	-2.397354
93	6	0	10.974361	0.836753	-2.513965
94	1	0	10.832421	0.336665	-3.485768
95	1	0	11.379743	1.835348	-2.699054
96	1	0	11.713729	0.269311	-1.941951

Table S42. Coordinates for Optimized Structure of **1Py-Me**$^{2-}$ (dianion, no counter ions) in open shell singlet state (S_1).

E(UB3LYP) = -1973.397710 Hartree.

opt freq UB3LYP/6-31G(d,p) scrf=(solvent=acetonitrile) nosymm guess=(mix,always)

Center Number	Atomic Number	Atomic Type	Coordinates (Angstroms) X	Y	Z
1	7	0	3.713643	-0.690098	0.405561
2	7	0	-3.659295	0.672319	0.401768
3	7	0	-9.354847	-0.953946	-2.409044
4	6	0	-0.859974	1.163694	0.987573
5	6	0	0.913178	-1.109336	1.116854
6	6	0	0.567144	1.340057	0.956137
7	6	0	-2.729449	-0.369872	0.380555
8	6	0	1.446718	0.192537	0.757278
9	6	0	-0.513778	-1.279212	1.191628
10	6	0	-1.404025	-0.174506	0.856035
11	6	0	-3.159900	-1.573487	-0.276522
12	1	0	-2.441661	-2.378206	-0.370978
13	6	0	2.767370	0.322533	0.247175
14	6	0	-5.361540	-0.643373	-0.790926
15	6	0	1.064560	2.644434	1.204698
16	1	0	2.135644	2.792916	1.296277
17	6	0	-4.393702	-1.714888	-0.838960
18	1	0	-4.624700	-2.637946	-1.361712
19	6	0	3.186895	1.435314	-0.563112
20	1	0	2.444566	2.184367	-0.808238
21	6	0	1.796203	-2.197790	1.378753
22	6	0	-6.677459	-0.736495	-1.341492
23	6	0	-1.741764	2.274295	1.135715
24	6	0	-4.879001	0.517617	-0.152247
25	1	0	-5.516373	1.382739	-0.050188
26	6	0	-1.175950	3.542370	1.298332
27	1	0	-1.826164	4.406271	1.421652
28	7	0	9.539721	0.870287	-2.175140
29	6	0	-1.004691	-2.519252	1.668226
30	1	0	-2.072044	-2.636207	1.828571
31	6	0	5.451268	0.551767	-0.818880
32	6	0	0.214088	3.728646	1.363804
33	1	0	0.628264	4.715977	1.561533
34	6	0	6.801075	0.646556	-1.276673
35	6	0	1.233406	-3.410910	1.790903
36	1	0	1.886918	-4.252651	2.012591
37	6	0	4.960715	-0.550672	-0.090196
38	1	0	5.607679	-1.388302	0.126960
39	6	0	-4.228332	3.294033	0.723777
40	1	0	-5.306753	3.148038	0.894930
41	1	0	-3.980026	4.146495	1.375226
42	6	0	4.442566	1.553931	-1.078227
43	1	0	4.663784	2.400425	-1.721094
44	6	0	-7.205985	-1.948697	-1.924719
45	1	0	-6.606839	-2.852449	-1.950500
46	6	0	-0.149638	-3.569820	1.966317
47	1	0	-0.553000	-4.507138	2.345768
48	6	0	4.085323	-3.342857	0.520089

49	1	0	5.186784	-3.311419	0.566340
50	1	0	3.825949	-4.232099	1.114467
51	6	0	-3.694654	1.669049	2.847008
52	1	0	-3.041371	0.854605	3.197063
53	1	0	-3.404816	2.544426	3.451289
54	6	0	-7.613160	0.370842	-1.385653
55	1	0	-7.336480	1.345300	-0.998268
56	6	0	4.113533	-1.847939	2.756499
57	1	0	3.971337	-2.786890	3.317217
58	1	0	5.206196	-1.755763	2.629108
59	6	0	-8.461131	-2.033238	-2.439440
60	1	0	-8.854928	-2.947683	-2.868230
61	6	0	-8.859401	0.256109	-1.909629
62	1	0	-9.552602	1.088966	-1.946707
63	6	0	7.311077	1.788891	-2.000985
64	1	0	6.669739	2.638492	-2.208999
65	6	0	7.796813	-0.389044	-1.074087
66	1	0	7.546262	-1.302392	-0.545673
67	6	0	8.596361	1.873828	-2.434504
68	1	0	8.974527	2.736136	-2.971737
69	6	0	3.626457	-0.682676	3.630925
70	1	0	2.551291	-0.760973	3.831406
71	1	0	3.783600	0.281796	3.132948
72	1	0	4.137226	-0.631730	4.603247
73	6	0	-4.020262	3.733023	-0.736389
74	1	0	-4.601140	4.627230	-1.002651
75	1	0	-4.308848	2.943091	-1.440960
76	1	0	-2.965386	3.957844	-0.929796
77	6	0	9.072693	-0.275436	-1.520873
78	1	0	9.812266	-1.053746	-1.369378
79	5	0	-3.340362	2.014410	1.261748
80	6	0	3.662815	-3.597080	-0.935513
81	1	0	2.573867	-3.699430	-1.013390
82	1	0	4.108533	-4.506747	-1.361846
83	1	0	3.951105	-2.762645	-1.586999
84	6	0	-5.148620	1.302190	3.186170
85	1	0	-5.842278	2.114864	2.936712
86	1	0	-5.293126	1.075251	4.252160
87	1	0	-5.481576	0.420521	2.624343
88	5	0	3.407969	-2.033922	1.272379
89	6	0	-10.508062	-0.949795	-3.293630
90	1	0	-10.249517	-0.674788	-4.327788
91	1	0	-10.963510	-1.943495	-3.305986
92	1	0	-11.250564	-0.238086	-2.923645
93	6	0	10.755953	0.803191	-2.967822
94	1	0	10.592210	0.357111	-3.960909
95	1	0	11.159209	1.809913	-3.105709
96	1	0	11.503700	0.204774	-2.440950

Table S43. Coordinates for Optimized Structure of **C1H** in the ground state (S_0).

E(RB3LYP) = --1392.40902040 Hartree.

opt RB3LYP/6-31G(d,p) scrf=(solvent=acetonitrile)

Center Number	Atomic Number	Atomic Type	Coordinates (Angstroms) X	Y	Z
1	6	0	-1.140835	0.760288	0.028651
2	6	0	1.258039	-0.747116	0.100343
3	6	0	0.145840	1.421636	0.031694
4	6	0	-2.483667	-1.225392	-0.591730
5	6	0	1.332174	0.649651	-0.131296
6	6	0	-0.030915	-1.405682	0.100332
7	6	0	-1.207702	-0.642923	-0.139038
8	6	0	-2.508080	-2.399380	-1.373520
9	1	0	-1.574264	-2.883559	-1.632782
10	6	0	2.632959	1.231477	-0.508031
11	6	0	-4.901759	-2.284027	-1.570082
12	6	0	0.168236	2.829727	0.272467
13	1	0	1.118834	3.336590	0.373166
14	6	0	-3.700202	-2.931451	-1.852769
15	1	0	-3.686605	-3.836914	-2.451992
16	6	0	2.712213	2.424386	-1.261346
17	1	0	1.800631	2.885863	-1.620096
18	6	0	2.448200	-1.506943	0.368344
19	6	0	-2.348347	1.517797	0.224709
20	6	0	-4.887569	-1.093026	-0.843365
21	1	0	-5.831338	-0.593855	-0.660451
22	6	0	-2.248664	2.878915	0.421436
23	1	0	-3.135230	3.472419	0.609742
24	6	0	-0.081258	-2.796298	0.414207
25	1	0	-1.043350	-3.287332	0.488164
26	6	0	5.114031	2.404630	-1.152010
27	6	0	-0.993172	3.532647	0.455679
28	1	0	-0.955100	4.596963	0.668058
29	6	0	2.321665	-2.848081	0.672320
30	1	0	3.197799	-3.432732	0.929073
31	6	0	5.053138	1.185300	-0.480916
32	1	0	5.984127	0.708733	-0.194317
33	6	0	-4.897831	1.680737	0.098968
34	1	0	-5.817275	1.138787	0.334472
35	1	0	-4.899164	2.529505	0.791032
36	6	0	3.931620	3.009104	-1.579746
37	1	0	3.956685	3.927270	-2.158870
38	6	0	1.063170	-3.491865	0.704514
39	1	0	1.007827	-4.539333	0.985461
40	6	0	4.890838	-1.740377	-0.259442
41	1	0	5.880989	-1.298843	-0.112438
42	1	0	4.933087	-2.700730	0.263177
43	6	0	-3.720851	0.426546	1.977772
44	1	0	-2.818295	-0.148197	2.213504
45	1	0	-3.633880	1.374135	2.523268
46	6	0	4.258723	-0.720007	1.955343
47	1	0	4.331638	-1.736731	2.360414
48	1	0	5.273184	-0.302831	1.979422
49	6	0	3.347216	0.118247	2.854242
50	1	0	2.336726	-0.298287	2.904438
51	1	0	3.268788	1.150207	2.498291
52	1	0	3.746150	0.147496	3.873314

53	6	0	-4.977142	2.194835	-1.343461
54	1	0	-5.870228	2.814065	-1.476383
55	1	0	-5.033124	1.371321	-2.061153
56	1	0	-4.106838	2.802597	-1.605399
57	6	0	4.658965	-1.986043	-1.752866
58	1	0	3.697545	-2.476517	-1.932500
59	1	0	5.444363	-2.632208	-2.158105
60	1	0	4.670701	-1.050825	-2.320632
61	6	0	-4.945687	-0.341201	2.485116
62	1	0	-5.872061	0.229530	2.369869
63	1	0	-4.827250	-0.555625	3.552397
64	1	0	-5.071281	-1.296394	1.967184
65	1	0	6.077114	2.858680	-1.364929
66	1	0	-5.844574	-2.686489	-1.928200
67	6	0	3.829461	-0.836589	0.452371
68	6	0	-3.680890	0.775234	0.438951
69	6	0	3.833828	0.555748	-0.189279
70	6	0	-3.696800	-0.531631	-0.362928

Table S44. Coordinates for Optimized Structure of **C1Py** in the ground state (S_0).

E(RB3LYP) = -1886.61494219 Hartree.

opt RB3LYP/6-31G(d,p) scrf=(solvent=acetonitrile)

Center Number	Atomic Number	Forces (Hartrees/Bohr) X	Y	Z
1	7	0.000000353	-0.000000646	-0.000001239
2	6	-0.000003830	0.000002134	0.000000281
3	6	-0.000000282	0.000001602	-0.000000002
4	6	-0.000000288	0.000005939	0.000001219
5	6	-0.000013268	0.000013879	0.000001168
6	6	-0.000001061	-0.000007180	0.000001703
7	6	-0.000002988	0.000001928	-0.000000936
8	6	0.000005418	0.000000635	0.000000850
9	6	-0.000005891	-0.000008353	-0.000008670
10	1	0.000000789	0.000003172	0.000001501
11	6	0.000008313	-0.000007750	0.000000444
12	6	0.000002563	0.000011254	0.000006127
13	6	-0.000000223	-0.000000527	-0.000000721
14	1	0.000001954	-0.000001238	-0.000003345
15	6	0.000004490	-0.000006172	0.000000245
16	1	-0.000001067	0.000001830	-0.000001891
17	6	0.000000282	0.000006644	-0.000001965
18	1	-0.000003140	0.000000432	0.000004686
19	6	0.000006574	-0.000001646	0.000001523
20	6	-0.000001371	-0.000002443	-0.000002608
21	6	-0.000004818	-0.000008817	0.000002475
22	6	-0.000007249	-0.000008635	-0.000007793
23	1	-0.000000434	0.000002551	0.000001600
24	6	0.000001939	0.000006845	0.000002072
25	1	0.000001338	-0.000000768	-0.000002108
26	7	0.000000188	0.000000946	-0.000000647
27	6	0.000003365	-0.000005560	0.000004177
28	1	0.000001592	-0.000000767	-0.000000380
29	6	-0.000002572	-0.000003997	0.000003652
30	6	-0.000002564	-0.000000528	-0.000001114
31	1	0.000000038	-0.000000150	0.000000922

32	6	0.000000157	0.000001263	-0.000000411
33	6	0.000002822	-0.000001400	0.000000170
34	1	-0.000001844	0.000000721	-0.000001019
35	6	0.000001501	0.000006218	-0.000003099
36	1	0.000000297	-0.000000619	0.000001501
37	6	-0.000002758	-0.000001629	0.000004160
38	1	0.000001944	-0.000000407	-0.000001232
39	1	0.000000950	-0.000003586	0.000000352
40	6	-0.000000256	0.000003514	-0.000001187
41	1	0.000001436	-0.000001070	0.000000242
42	6	0.000001009	0.000000662	0.000000076
43	1	0.000000182	-0.000000176	-0.000000442
44	6	-0.000002794	0.000002624	-0.000001697
45	1	0.000000521	-0.000000209	0.000000273
46	6	0.000002459	0.000002360	0.000007416
47	1	-0.000003076	-0.000001445	-0.000001200
48	1	0.000000037	0.000003378	0.000000610
49	6	0.000003883	0.000003134	-0.000005306
50	1	-0.000002536	-0.000000007	-0.000000812
51	1	-0.000000124	-0.000001213	-0.000002384
52	6	-0.000000428	-0.000000073	0.000000266
53	1	-0.000000018	-0.000000405	-0.000000773
54	6	0.000001639	-0.000006153	-0.000000707
55	1	-0.000001277	0.000000850	-0.000002146
56	1	-0.000001429	0.000001909	-0.000000747
57	6	-0.000000690	-0.000000145	-0.000000247
58	1	0.000000270	-0.000000094	-0.000000712
59	6	0.000000554	-0.000000113	-0.000000125
60	1	-0.000000131	-0.000000392	-0.000000563
61	6	-0.000000756	0.000000204	0.000000887
62	1	0.000000402	0.000000451	0.000000403
63	6	0.000000887	0.000000221	0.000000481
64	1	0.000000069	0.000000385	0.000000207
65	6	0.000001149	0.000000458	0.000001014
66	1	0.000000091	0.000000579	0.000000460
67	6	-0.000001323	0.000001532	0.000001645
68	1	0.000000982	-0.000000654	0.000000062
69	1	0.000000901	-0.000001659	0.000000491
70	1	-0.000000804	0.000000665	0.000000027
71	6	0.000002349	-0.000001961	-0.000001085
72	1	0.000000168	-0.000000450	-0.000001529
73	1	-0.000000833	-0.000000968	0.000001556
74	1	-0.000000452	0.000000147	0.000001696
75	6	-0.000000966	0.000000029	0.000001631
76	1	0.000000160	0.000000390	0.000000496
77	6	-0.000002045	-0.000001018	-0.000000343
78	1	0.000001294	-0.000000658	-0.000000205
79	1	-0.000000061	0.000000802	-0.000001187
80	1	0.000001299	0.000001285	0.000002065
81	6	-0.000001595	-0.000001581	0.000001771
82	1	-0.000000521	-0.000001285	-0.000000011
83	1	-0.000000716	0.000000568	-0.000001026
84	1	0.000001630	0.000000476	-0.000001166
85	6	-0.000004943	-0.000007715	0.000003863
86	6	0.000017733	0.000003702	0.000006834
87	6	-0.000003532	0.000009541	-0.000008026
88	6	-0.000001020	-0.000005594	-0.000002498

Table S45. Coordinates for Optimized Structure of **C1Py-BCF** in the ground state (S_0).

E(RB3LYP) = -6303.15843176 Hartree.

opt RB3LYP/6-31G(d,p) scrf=(solvent=acetonitrile)

Center Number	Atomic Number	Atomic Type	Coordinates (Angstroms) X	Y	Z
1	7	0	9.678715	0.482676	0.027512
2	7	0	-9.670754	0.372514	0.071983
3	6	0	1.414272	-1.862254	-0.305067
4	6	0	-0.769680	-1.927376	-1.475351
5	6	0	0.672377	-1.841130	-1.522224
6	6	0	-4.979861	-1.310691	-1.236282
7	1	0	-5.602895	-1.350656	-2.120069
8	6	0	-1.423286	-2.028613	-0.223331
9	6	0	0.778504	-2.353583	0.864474
10	6	0	-5.545374	-0.812291	-0.049866
11	6	0	2.808863	-1.411933	-0.203037
12	6	0	4.978515	-1.491616	0.862738
13	1	0	5.633026	-1.908830	1.618248
14	6	0	-1.537521	-1.935232	-2.690507
15	6	0	-0.664138	-2.442589	0.907468
16	6	0	5.526674	-0.606485	-0.078017
17	6	0	-2.842082	-1.652893	-0.158701
18	6	0	-6.962410	-0.408294	-0.004705
19	6	0	3.339134	-0.449540	-1.092506
20	1	0	2.687439	0.019081	-1.818567
21	6	0	1.295906	-1.809181	-2.805918
22	1	0	2.375088	-1.841399	-2.876129
23	6	0	4.665512	-0.057874	-1.043487
24	1	0	5.021141	0.700382	-1.732987
25	6	0	6.953282	-0.237818	-0.035809
26	6	0	-3.396939	-1.103883	1.016840
27	1	0	-2.771859	-0.976342	1.891644
28	6	0	-1.274939	-3.013163	2.062044
29	1	0	-2.348080	-3.155233	2.079992
30	6	0	-0.866826	-1.871795	-3.893952
31	1	0	-1.412044	-1.914649	-4.828936
32	6	0	1.554945	-2.789846	1.990742
33	6	0	-3.786426	-1.685411	-3.881732
34	1	0	-3.397666	-2.200230	-4.766287
35	1	0	-4.835556	-1.988470	-3.834973
36	6	0	-4.718258	-0.691565	1.077633
37	1	0	-5.107233	-0.295784	2.009729
38	6	0	3.638778	-2.295821	3.345113
39	1	0	3.310226	-3.003857	4.111499
40	1	0	4.729667	-2.380928	3.339820
41	6	0	0.546060	-1.819690	-3.952776
42	1	0	1.036875	-1.825560	-4.921202
43	6	0	7.693832	-0.233086	1.158489
44	1	0	7.233912	-0.482662	2.106393
45	6	0	-3.155422	-3.814774	-2.629578
46	1	0	-2.655471	-4.169176	-3.538845
47	1	0	-2.564365	-4.190568	-1.787092
48	6	0	9.030800	0.120530	1.154765
49	1	0	9.613149	0.109373	2.064430
50	6	0	7.654426	0.123588	-1.203202

51	1	0	7.171486	0.111865	-2.172310
52	6	0	-9.258599	-0.620892	-0.743318
53	1	0	-10.020750	-1.110722	-1.331614
54	6	0	0.896408	-3.340193	3.072907
55	1	0	1.460376	-3.725805	3.914422
56	6	0	-3.711486	-0.168884	-4.095551
57	1	0	-4.268166	0.113623	-4.994701
58	1	0	-2.680207	0.172202	-4.219549
59	1	0	-4.141034	0.380197	-3.252552
60	6	0	-0.511309	-3.460724	3.108619
61	1	0	-0.983517	-3.933521	3.964267
62	6	0	-7.418227	0.610986	0.854358
63	1	0	-6.734505	1.149331	1.498556
64	6	0	-7.937642	-1.025249	-0.806789
65	1	0	-7.684021	-1.846296	-1.465675
66	6	0	8.987824	0.466734	-1.137734
67	1	0	9.547326	0.722104	-2.028442
68	6	0	3.238180	-0.877235	3.759472
69	1	0	2.151464	-0.773817	3.831858
70	1	0	3.599651	-0.130683	3.045816
71	1	0	3.662695	-0.634723	4.738807
72	6	0	-8.750186	0.965211	0.870014
73	1	0	-9.118541	1.739493	1.530529
74	6	0	3.616125	-4.241178	1.742594
75	1	0	4.711556	-4.212429	1.796006
76	1	0	3.279857	-4.842737	2.595610
77	6	0	-4.562958	-4.414758	-2.556772
78	1	0	-5.174045	-4.151312	-3.425179
79	1	0	-5.097665	-4.096384	-1.657078
80	1	0	-4.492374	-5.506984	-2.529101
81	6	0	3.191968	-4.926209	0.441765
82	1	0	2.104794	-5.028497	0.374780
83	1	0	3.624124	-5.930485	0.388907
84	1	0	3.533695	-4.371959	-0.437936
85	5	0	11.286599	0.760310	-0.029646
86	6	0	11.891587	1.079078	1.471582
87	6	0	11.810487	-0.644795	-0.721178
88	6	0	11.625731	2.131677	-0.880470
89	6	0	11.310730	2.065356	2.278178
90	6	0	13.093146	0.570458	1.976291
91	6	0	12.137301	-0.810428	-2.068400
92	6	0	11.808438	-1.834846	0.013525
93	6	0	12.950124	2.368356	-1.267042
94	6	0	10.763076	3.203066	-1.123884
95	6	0	11.823373	2.483345	3.501957
96	9	0	10.172064	2.676128	1.874068
97	6	0	13.647497	0.964724	3.192961
98	9	0	13.807564	-0.345914	1.292253
99	6	0	12.492161	-2.033965	-2.635563
100	9	0	12.095222	0.232935	-2.928731
101	6	0	12.155479	-3.076393	-0.506120
102	9	0	11.444833	-1.818795	1.317193
103	6	0	13.390012	3.534411	-1.880550
104	9	0	13.890590	1.425203	-1.035133
105	6	0	11.159277	4.390664	-1.740240
106	9	0	9.459032	3.148427	-0.769335
107	6	0	13.009140	1.926323	3.965639
108	9	0	11.194699	3.423926	4.223976
109	9	0	14.800792	0.425834	3.619502
110	6	0	12.506525	-3.177334	-1.848115
111	9	0	12.803930	-2.115349	-3.939131

112	9	0	12.144441	-4.173394	0.268051
113	6	0	12.482100	4.560410	-2.123055
114	9	0	14.677606	3.682853	-2.231646
115	9	0	10.268926	5.373053	-1.954893
116	9	0	13.529693	2.313277	5.136855
117	9	0	12.840979	-4.362374	-2.374886
118	9	0	12.881294	5.695307	-2.712429
119	5	0	-11.241149	0.769029	0.276652
120	6	0	-12.164810	0.215720	-0.973156
121	6	0	-11.540647	0.069634	1.742299
122	6	0	-11.455495	2.403048	0.220146
123	6	0	-11.815716	0.504164	-2.298145
124	6	0	-13.419944	-0.386998	-0.837013
125	6	0	-11.570263	0.738529	2.967433
126	6	0	-11.634955	-1.321490	1.851865
127	6	0	-12.678157	2.931967	0.649879
128	6	0	-10.610446	3.340159	-0.378995
129	6	0	-12.594147	0.181342	-3.404934
130	9	0	-10.648734	1.138493	-2.560416
131	6	0	-14.236496	-0.721540	-1.916072
132	9	0	-13.929372	-0.676497	0.377365
133	6	0	-11.740056	0.100124	4.195567
134	9	0	-11.399013	2.078943	3.033542
135	6	0	-11.805513	-2.002554	3.051410
136	9	0	-11.553391	-2.089747	0.740443
137	6	0	-13.031433	4.271338	0.545907
138	9	0	-13.602392	2.112102	1.198911
139	6	0	-10.921559	4.694903	-0.502124
140	9	0	-9.409117	2.979340	-0.884851
141	6	0	-13.822472	-0.439283	-3.211506
142	9	0	-12.177949	0.476769	-4.646215
143	9	0	-15.426420	-1.308136	-1.710185
144	6	0	-11.863376	-1.282019	4.239643
145	9	0	-11.768469	0.811100	5.334351
146	9	0	-11.903532	-3.341486	3.074289
147	6	0	-12.140305	5.167207	-0.036417
148	9	0	-14.221548	4.704951	0.991825
149	9	0	-10.051646	5.540781	-1.078003
150	9	0	-14.596108	-0.756401	-4.257153
151	9	0	-12.023228	-1.914172	5.409484
152	9	0	-12.457533	6.463854	-0.149644
153	6	0	3.633811	-1.872481	0.850755
154	6	0	-3.643852	-1.705545	-1.325963
155	6	0	-3.045783	-2.239716	-2.632453
156	6	0	3.091709	-2.779756	1.961087

Table S46. Coordinates for Optimized Structure of **C1Py-Me^{2+}** (with PF_6^-) in the ground state (S_0).

E(RB3LYP) = -3847.63583086 Hartree.

opt RB3LYP/6-31G(d,p) scrf=(solvent=acetonitrile)

Center Number	Atomic Number	Atomic Type	Coordinates (Angstroms) X	Y	Z
1	7	0	-8.910035	0.141313	1.297400
2	6	0	0.190374	-1.733510	1.571321

3	6	0	1.346827	-2.385355	-0.938115
4	6	0	1.614003	-1.600858	1.362015
5	6	0	-2.080625	-1.671421	0.603243
6	6	0	2.143435	-1.742873	0.045518
7	6	0	-0.076641	-2.520744	-0.724259
8	6	0	-0.656095	-1.999155	0.467183
9	6	0	-2.847418	-1.280249	-0.515485
10	1	0	-2.382990	-1.241652	-1.492560
11	6	0	3.478588	-1.269736	-0.339820
12	6	0	-4.798633	-0.917147	0.862275
13	6	0	2.440149	-1.402026	2.508712
14	1	0	3.516224	-1.393745	2.396982
15	6	0	-4.176598	-0.911067	-0.396904
16	1	0	-4.731239	-0.640471	-1.288730
17	6	0	4.099510	-0.189966	0.329500
18	1	0	3.556749	0.337533	1.103077
19	6	0	1.944476	-2.924341	-2.126598
20	6	0	-6.216221	-0.553989	1.011918
21	6	0	-0.363762	-1.620320	2.892387
22	6	0	-4.021919	-1.258057	1.984269
23	1	0	-4.483617	-1.206733	2.961274
24	6	0	0.493808	-1.398496	3.949568
25	1	0	0.113888	-1.346921	4.962599
26	7	0	10.130623	0.688899	-1.857656
27	6	0	-0.842710	-3.239576	-1.687069
28	1	0	-1.895488	-3.415069	-1.505497
29	6	0	6.103072	-0.425408	-0.995413
30	6	0	1.892584	-1.300254	3.760744
31	1	0	2.537135	-1.178523	4.625804
32	6	0	7.488071	-0.041918	-1.305845
33	6	0	1.142438	-3.615637	-3.013740
34	1	0	1.576588	-4.078274	-3.892705
35	6	0	5.454721	-1.438508	-1.720745
36	1	0	5.980067	-1.895936	-2.550241
37	6	0	-2.392809	-1.294476	4.414214
38	1	0	-3.420013	-1.629435	4.580315
39	1	0	-1.839345	-1.693085	5.270547
40	6	0	5.378483	0.233975	0.014460
41	1	0	5.819180	1.053083	0.571440
42	6	0	-6.856768	0.323737	0.116096
43	1	0	-6.318062	0.783781	-0.702172
44	6	0	-0.243307	-3.782467	-2.794003
45	1	0	-0.827948	-4.365147	-3.499141
46	6	0	3.735611	-2.489186	-3.862344
47	1	0	4.813484	-2.539919	-4.044452
48	1	0	3.309896	-3.273509	-4.494974
49	6	0	-1.882099	-3.536892	3.313071
50	1	0	-1.425270	-3.991614	2.427174
51	1	0	-1.220276	-3.771095	4.155289
52	6	0	-7.008196	-1.067783	2.061086
53	1	0	-6.603859	-1.770433	2.778158
54	6	0	4.084930	-4.277201	-2.116884
55	1	0	3.637150	-4.968618	-2.840625
56	1	0	5.152253	-4.218785	-2.363794
57	6	0	-8.187058	0.653352	0.273088
58	1	0	-8.701943	1.326251	-0.397391
59	6	0	-8.331937	-0.709523	2.180082
60	1	0	-8.969231	-1.091303	2.967331

61	6	0	7.970976	1.252755	-1.030925
62	1	0	7.338463	2.020007	-0.609762
63	6	0	8.405752	-0.950536	-1.874582
64	1	0	8.127057	-1.974595	-2.086921
65	6	0	9.277699	1.587546	-1.308772
66	1	0	9.667987	2.574124	-1.098366
67	6	0	3.922821	-4.845545	-0.705454
68	1	0	2.869038	-4.972799	-0.440242
69	1	0	4.384538	-4.198344	0.046824
70	1	0	4.403283	-5.826697	-0.638211
71	6	0	-2.352882	0.238078	4.445111
72	1	0	-2.764232	0.607268	5.389879
73	1	0	-2.941314	0.675200	3.633109
74	1	0	-1.332131	0.618533	4.353241
75	6	0	9.701081	-0.565749	-2.139731
76	1	0	10.431655	-1.239738	-2.567625
77	6	0	3.203797	-1.126698	-4.315321
78	1	0	2.117942	-1.062537	-4.198303
79	1	0	3.436078	-0.962031	-5.372189
80	1	0	3.652104	-0.307874	-3.744525
81	6	0	-3.253086	-4.177566	3.550746
82	1	0	-3.718800	-3.833488	4.478980
83	1	0	-3.138771	-5.263622	3.628461
84	1	0	-3.946869	-3.977784	2.728822
85	6	0	-10.344191	0.478628	1.440581
86	1	0	-10.555716	0.671073	2.491867
87	1	0	-10.557390	1.361045	0.843410
88	1	0	-10.944346	-0.362086	1.088361
89	6	0	11.515573	1.083063	-2.190344
90	1	0	11.561500	1.395233	-3.235240
91	1	0	11.814216	1.906364	-1.544349
92	1	0	12.176207	0.232975	-2.026943
93	15	0	-10.549259	2.674205	-2.180673
94	9	0	-12.163027	2.855112	-2.052305
95	9	0	-10.685815	1.106038	-1.713013
96	9	0	-10.700624	2.228947	-3.739546
97	9	0	-8.919515	2.479454	-2.277983
98	9	0	-10.376734	3.100592	-0.597327
99	9	0	-10.389710	4.235379	-2.616318
100	15	0	7.106571	4.215650	1.360161
101	9	0	8.167614	4.695203	2.499748
102	9	0	5.879813	4.329890	2.426901
103	9	0	6.055115	3.707424	0.201639
104	9	0	8.335505	4.082896	0.273038
105	9	0	7.341445	2.650321	1.795750
106	9	0	6.871472	5.760534	0.898668
107	6	0	3.460801	-2.861313	-2.366886
108	6	0	-1.844888	-1.969550	3.125631
109	6	0	-2.674256	-1.606121	1.888963
110	6	0	4.142937	-1.835922	-1.454501

References

[1] J. Zuo, K. Liu, J. Harrell, L. Fang, P. Piotrowiak, D. Shimoyama, R. A. Lalancette, F. Jäkle, "Near-IR Emissive B-N Lewis Pair-Functionalized Anthracenes via Selective LUMO Extension in Conjugated Dimer and Polymer" *Angew. Chem. Int. Ed.* **2024**, *63*, e202411855.

[2] C. Wolf, B. T. Ghebremariam, "Synthesis of Atropisomeric 1,8-Bis(4',4'-dipyridyl)naphthalenes from 4-Trimethylstannylpyridines" *Synthesis* **2002**, *2002*, 749–752.

[3] *SHELXTL-NT, Version 6.10; Bruker AXS*, Madison, Wisconsin, USA, **2000**.

[4] (a) G. M. Sheldrick, *Acta Crystallogr A Found Adv* **2015**, *71*, 3-8; (b) O. V. Dolomanov, A. J. Blake, N. R. Champness, M. Schröder, *J. Appl. Crystallogr.* **2003**, *36*, 1283-1284; (c) O. V. Dolomanov, L. J. Bourhis, R. J. Gildea, J. A. K. Howard, H. Puschmann, *J. Appl. Crystallogr.* **2009**, *42*, 339-341.

[5] M. J. Frisch, G. W. Trucks, H. B. Schlegel, G. E. Scuseria, M. A. Robb, J. R. Cheeseman, G. Scalmani, V. Barone, G. A. Petersson, H. Nakatsuji, X. Li, M. Caricato, A. V. Marenich, J. Bloino, B. G. Janesko, R. Gomperts, B. Mennucci, H. P. Hratchian, J. V. Ortiz, A. F. Izmaylov, J. L. Sonnenberg, Williams, F. Ding, F. Lipparini, F. Egidi, J. Goings, B. Peng, A. Petrone, T. Henderson, D. Ranasinghe, V. G. Zakrzewski, J. Gao, N. Rega, G. Zheng, W. Liang, M. Hada, M. Ehara, K. Toyota, R. Fukuda, J. Hasegawa, M. Ishida, T. Nakajima, Y. Honda, O. Kitao, H. Nakai, T. Vreven, K. Throssell, J. A. Montgomery Jr., J. E. Peralta, F. Ogliaro, M. J. Bearpark, J. J. Heyd, E. N. Brothers, K. N. Kudin, V. N. Staroverov, T. A. Keith, R. Kobayashi, J. Normand, K. Raghavachari, A. P. Rendell, J. C. Burant, S. S. Iyengar, J. Tomasi, M. Cossi, J. M. Millam, M. Klene, C. Adamo, R. Cammi, J. W. Ochterski, R. L. Martin, K. Morokuma, O. Farkas, J. B. Foresman, D. J. Fox, Wallingford, CT, **2016**.

[6] T. A. K. Roy Dennington, and John M. Millam, Semichem Inc., Shawnee Mission, KS, **2016**.

[7] J. Zuo, R. A. Lalancette, D. E. Prokopchuk, F. Jäkle, "Regioselective access to B-N Lewis pair-functionalized anthracenes: mechanistic studies and optoelectronic properties" *Chem. Sci.* **2025**, *16*, 8114–8124.
